# Supplementary material for: A novel histopathological classification of implant periapical lesion: A systematic review and treatment decision tree
Source: PLoS One. 2022 Dec 22;17(12):e0277387. doi: 10.1371/journal.pone.0277387 (PMC9778521; doi:10.1371/journal.pone.0277387)
Supplement: S1 File — (ZIP) [file pone.0277387.s001.zip › support files/Included study/Balshi 2007.pdf]

A Retrospective Evaluation of a Treatment Protocol for Dental Implant Periapical Lesions: Long-term Results of 39 Implant Apicoectomies

Stephen F. Balshi, MBE[1]/Glenn J. Wolfinger, DMD[2]/Thomas J. Balshi, DDS[3]

Purpose: *The goal of this study was to evaluate retrospectively the efficacy of a treatment modality for a lesion at the apical portion of a nonmobile dental implant.* ***Materials and Methods:*** *All patients were treated with an intraoral treatment approach. A flap was elevated facial to the effected implant site, exposing the bone. A carbide bur was used to open a window in the bone. The bur was then used to cut the implant and completely remove the affected portion of the implant. The surgical site was then closed with interrupted vicryl sutures, and patients were prescribed.* ***Results:*** *Thirty-nine implants in 35 patients with a mean age of 58.3 years were identified clinically and radiographically with the presence of a periapical lesion. These 39 implants, which constituted 9.9% of implants (39 of 395) placed in these 35 patients, were consecutively treated using the intraoral apicoectomy procedure. Thirty-eight of the 39 implants (97.4%) treated with this technique remained stable and continued in function with no further complication. Follow-up time averaged 4.54 years; the longest follow-up time exceeded 15 years.* ***Conclusion:*** *Based upon the results of this retrospective study, lesions in the apical region of implants can be treated successfully using an intraoral apicoectomy procedure.* INT J ORAL MAXILLOFAC IMPLANTS 2007;22:267–272

Key words: *complications, machined implants, osseointegration, overheating, periapical abscesses*

The use of dental implants for the treatment of edentulous and partially edentulous patients with fixed prostheses has increased in recent years.[1] Despite the most scrupulous attempts to maintain a sterile surgical environment[2] and precautionary surgical techniques, dental implant patients have developed a variety of complications associated with implant placement.[3,4] One such complication is pathology at the apex of an implant, which may be characterized by suppuration, fistula formation, and loss of alveolar bone.[5] An infection that forms at the implant apex possesses the ability to spread coronally, proximally, lingually, and facially. This transmission of infection can not only destroy the bony interface of the infected implant but also result in the loss of support of the adjacent teeth and implants.[6] Poor patient compliance with follow-up maintenance or unfamiliarity on the part of the clinician in this clinical scenario can result in implant failure, prosthesis failure, and even failure of an adjacent tooth or implant.

According to Meffert,[7] problematic implants can be placed in 1 of 3 categories: ailing, failing, or failed. Ailing implants exhibit bone loss with pocket formation; however, they are static at maintenance checks. Failing implants exhibit bone loss, pocket formation, bleeding upon probing, and purulent exudates. Failed implants have mobility, a dull sound on percussion, and peri-implant radiolucency. Etiologic factors such as surgical trauma and the role of microorganisms have been reported by Mombelli et al.[8] Becker et al[9] suggested overheating of the bone and insufficient bone volume as possible factors for implant failure.

Ailing and failing implants may be treated in an attempt to preserve the implant; failed implants must be removed, since they are nonfunctional and bone loss will continue.[10] The apical lesion described in this study creates a fourth category, since pocket pathology is not related to this condition.

[1]Director of Research and Biomedical Engineering, Prosthodontics Intermedica, Institute for Facial Esthetics, Fort Washington, Pennsylvania.

[2]Prosthodontist, Prosthodontics Intermedica, Institute for Facial Esthetics, Fort Washington, Pennsylvania; Staff Prosthodontist, VA Medical Center, Philadelphia, Pennsylvania.

[3]Founder, Prosthodontics Intermedica, Institute for Facial Esthetics, Fort Washington, Pennsylvania.

Correspondence to: Mr Stephen F. Balshi, Prosthodontics Intermedica, 467 Pennsylvania Ave, Suite 201, Fort Washington, PA 19034. Fax: +215 643 1149. E-mail: balshi2@aol.com

COPYRIGHT © 2007 BY QUINTESSENCE PUBLISHING CO, INC. PRINTING OF THIS DOCUMENT IS RESTRICTED TO PERSONAL USE ONLY. NO PART OF THIS ARTICLE MAY BE REPRODUCED OR TRANSMITTED IN ANY FORM WITHOUT WRITTEN PERMISSION FROM THE PUBLISHER

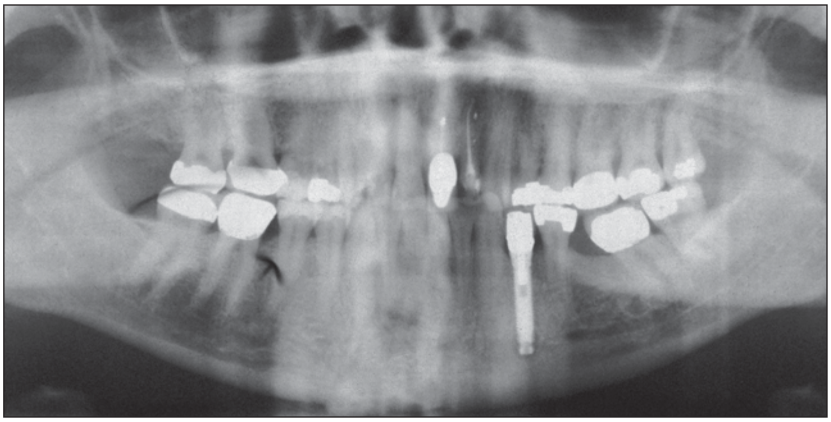

Fig 1 Panoramic radiograph with a radiolucent lesion at the apex of the implant in the area of the right first premolar.

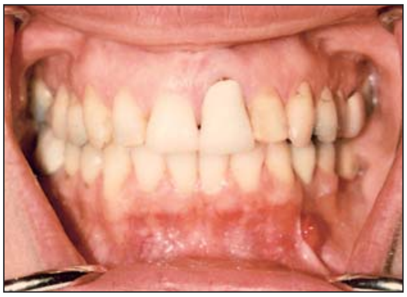

Fig 2 Buccal fistula draining from the implant apex.

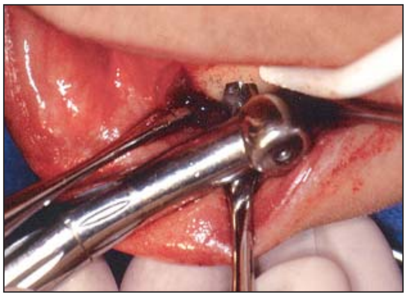

Fig 3 Carbide bur being used to cut a window in bone buccal to the implant to provide access to the affected area.

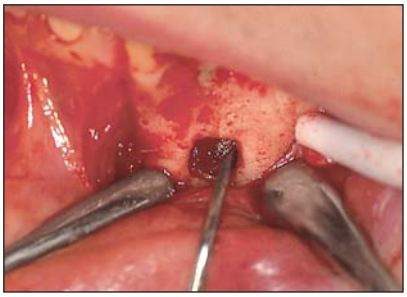

Fig 4 Vigorous debridement of bone.

This report describes an intraoral approach for treating implants with periapical lesions. The intention of the study is to describe a treatment protocol and to present the retrospective results of such treatment.

MATERIALS AND METHODS

A retrospective chart review was conducted of all patients with known bone lesions in the apical portion of implants. The lesions were identified either radiographically (radiolucency; Fig 1), by clinical observation (swelling, suppuration, fistula; Fig 2), or by a combination of these. If the lesion formed subsequent to functional loading of the implant, screw-retained prostheses were removed to enable individual implant testing. For single-tooth prostheses, mobility testing was conducted with the crown attached.

All patients were treated with an intraoral treatment approach. The area of the implant periapical lesion was locally anesthetized with a combination of bupivacaine hydrochloride and epinephrine (Marcaine 0.5%; Novoco Pharmaceuticals/Eastman Kodak, Cambridge, ON, Canada) and lidocaine hydrochloride (Lignospan Forte; Septodont, New Castle, DE), which provided hemostasis at the surgical site. A flap was elevated facial to the implant site, exposing the bone. A periapical film in the area of the implant apical lesion was used to measure the abscess. A carbide bur (SSW FG-557; SS White Burs, Lakewood, NJ) in a high-speed drill (Star Dental div. of Den-tal-ez, Lancaster, PA) was used to open a window in the bone that was slightly larger than the lesion itself (Fig 3). A curette was used to debride the bony defect (Fig 4). Biopsy samples of excised tissue were sent for microscopic analysis. A carbide bur was used to remove the affected portion of the implant (average, 3.6 mm; range, 2 to 6 mm; Fig 5). The area was thoroughly debrided and irrigated (1 capsule of 250 mg tetracycline/saline solution, Ivax Pharmaceuticals, Miami, FL) to remove any additional soft tissue or titanium debris (Fig 6). In most cases (72.0%), Bio-Oss bovine bone (Osteohealth, Shirley, NY) was used to graft the bony defect (Fig 7). In the case of larger openings (38%), a Bio-Gide membrane (Osteohealth) was used to cover the surgical area. A minority (28.0%) of the patient treatments were performed without bone grafting or membranes. The flap was closed using interrupted Vicryl sutures (Johnson & Johnson/Ethicon, Somerville, NJ). Pain medication (Motrin 600 mg, 1 tablet every 4 to 6 hours; McNeil, Fort Washington, PA) and antibiotics (amoxicillin 500 mg, 1 tablet 4 times/d) were prescribed. Panoramic and periapical radiographs were obtained following treatment (Fig 8).

COPYRIGHT © 2007 BY QUINTESSENCE PUBLISHING CO, INC. PRINTING OF THIS DOCUMENT IS RESTRICTED TO PERSONAL USE ONLY. NO PART OF THIS ARTICLE MAY BE REPRODUCED OR TRANSMITTED IN ANY FORM WITHOUT WRITTEN PERMISSION FROM THE PUBLISHER

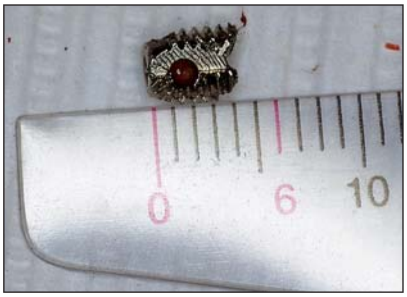

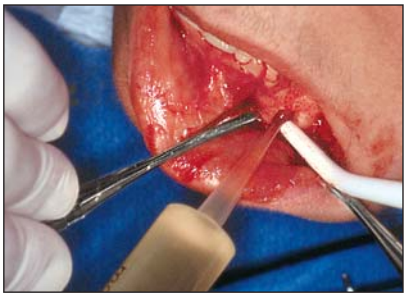

Fig 5 *(Left)* Apical portion of an implant after removal.

Fig 6 *(Right)* Antibiotic irrigation of bone crypt.

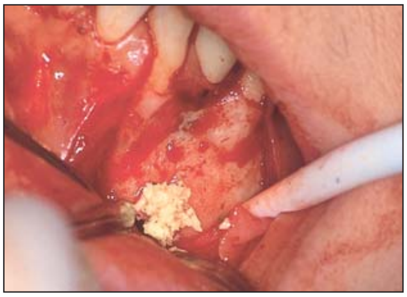

Fig 7 Bone graft (Bio-Oss).

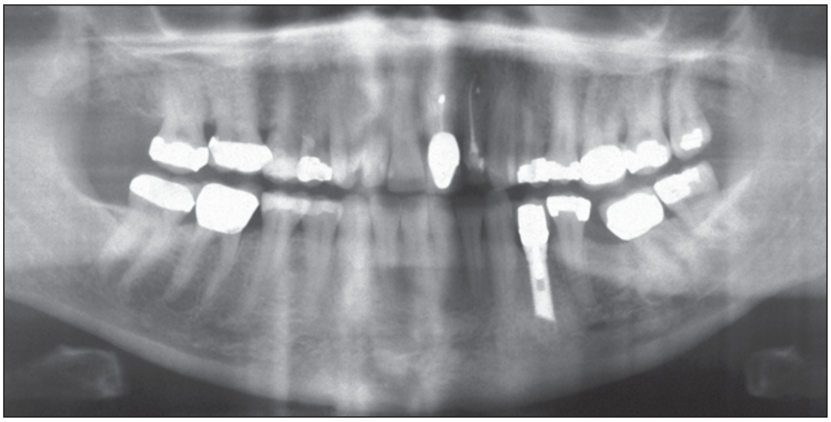

Fig 8 Postsurgical panoramic radiograph.

RESULTS

Thirty-five dental implant patients (22 women, 13 men) with a mean age of 58.3 years (range, 21 to 82 years) with an implant periapical lesion were consecutively treated using the intraoral dissection approach described. This procedure was offered to all patients with an osseous lesion in the apical portion of an implant. Patients were excluded if the lesion had spread coronally to the crest of the alveolar ridge, creating oral communication with the lesion, or had caused implant mobility or failure. All patients were treated at Prosthodontics Intermedica (The Institute for Facial Esthetics, Fort Washington, PA).

Brånemark System implants (Nobel Biocare) with an implant apical lesion constituted 9.9% (39 of 395) of the implants in this patient population. All 39 lesions were identified radiographically; however, only 66.7% (26 of 39) demonstrated clinical evidence of infection (eg, swelling, suppuration, fistula formation). Thirty-eight of 39 implants (97.4%) treated with the described intraoral resection technique remained stable and in clinical function, with no signs of reoccurrence after clinical and radiographic examination (Figs 9a to 9c).

Microscopic pathologic analysis from 37 of 39 sites showed a stroma of delicate bundles of immature collagen fibers interspersed by active fibrocytes and numerous dilated capillaries. Throughout the stroma an infiltrate of inflammatory cells, predominantly lymphocytes and plasma cells, was reported. None of the 37 biopsies demonstrated malignant features.

Seventeen of the treated implants in this study were in the maxilla (9 anterior, 8 posterior). The remaining 22 treated implants were in the mandible (11 anterior, 11 posterior). A majority (51.28%) of the implants were placed in type 3 bone; the remainder were placed in type 1 (2.56%), type 2 (33.33%), or type 4 bone (12.82%). The average length of the implant population was 15.5 mm.

For the 39 implant apicoectomy procedures that represent this study, there was an average of 1.64 years between the date of implant placement and the date of the apicoectomy procedure. However, the majority of apical lesions appeared within the first 2 years after initial implant placement. Follow-up time averaged 4.54 years (range, 0.84 years to 15.02 years); the cumulative survival rate (CSR) was 97.40% (Table 1).

DISCUSSION

The diagnosis and treatment of peri-implantitis, peri-implant lesions with involvement of the coronal por-

COPYRIGHT © 2007 BY QUINTESSENCE PUBLISHING CO, INC. PRINTING OF THIS DOCUMENT IS RESTRICTED TO PERSONAL USE ONLY. NO PART OF THIS ARTICLE MAY BE REPRODUCED OR TRANSMITTED IN ANY FORM WITHOUT WRITTEN PERMISSION FROM THE PUBLISHER

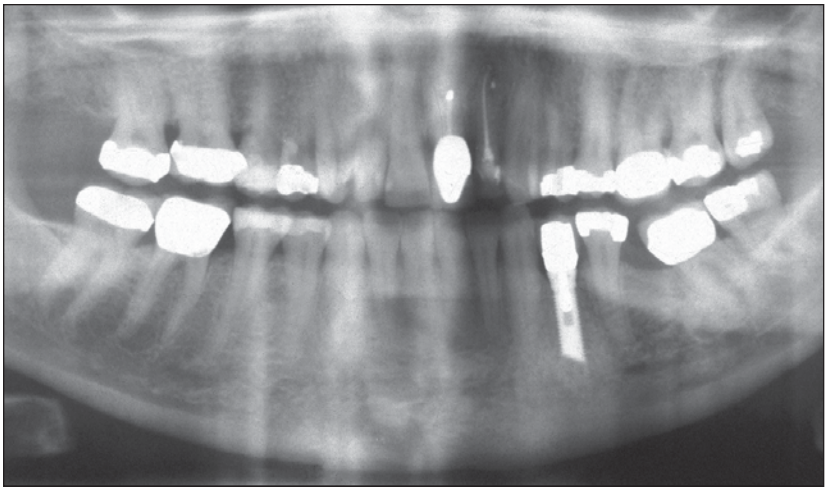

Fig 9a Panoramic radiograph 6 months after implant apicoectomy.

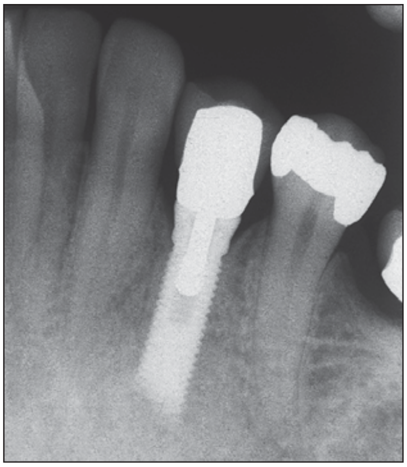

Fig 9b Periapical radiograph 6 months after implant apicoectomy.

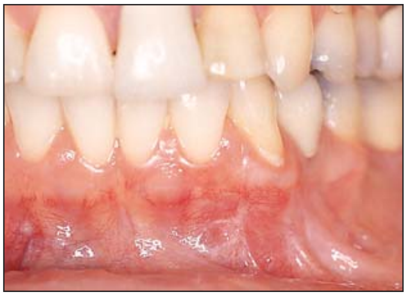

Fig 9c Healed surgical site.

Table 1 Life Table Analysis

Years	No. of implants	No. of failures in period	Survival rate for period (%)	CSR (%)
0 to 1	39	1	97.40	97.40
1 to 2	37	0	100.00	97.40
2 to 3	30	0	100.00	97.40
3 to 4	22	0	100.00	97.40
4 to 5	17	0	100.00	97.40
5+	15	0	100.00	97.40

tion of the implant, has been described in the literature.[11,12] Other reports have discussed etiology, prevention, and treatment of implant periapical lesions.[5,6,13–20] These reports vary from treatment with antibiotics,[11,14,17,18] treatment by detoxification of the implant,[15–18] and treatment by implant removal.[6,14,19,20] The current study illustrates treatment by removal of only the involved portion of the implant, thereby maintaining the osseointegrated portion of the implant and maintaining the prosthesis.

It has been proposed that the most likely causes of periapical lesions are *(1)* bacterial infection from either remnants of extracted natural teeth or a seeding mechanism from the remaining natural teeth[21–23] *(2)* overheating of the bone during the creation of the osteotomy site by placement of long implants with external irrigation[24,25]; *(3)* microfractures in the bone caused from micromotion (overload)[26]; and *(4)* residual bone cavities created by the placement of implants that are shorter than the prepared osteotomy site.[13] The results of the present study neither confirmed nor contradicted any of these hypotheses; rather, the present data suggest that the etiology of such lesions is multifactorial.

This study demonstrates that the implant apicoectomy procedure described is an effective treatment method to maintain an implant with an apical lesion providing a stable state of osseointegration without further complication. Follow-up time for this study averaged 4.54 years, with the longest follow-up time exceeding 15 years. These results provide some long-term data for this surgical procedure.

COPYRIGHT © 2007 BY QUINTESSENCE PUBLISHING CO, INC. PRINTING OF THIS DOCUMENT IS RESTRICTED TO PERSONAL USE ONLY. NO PART OF THIS ARTICLE MAY BE REPRODUCED OR TRANSMITTED IN ANY FORM WITHOUT WRITTEN PERMISSION FROM THE PUBLISHER

Thirty-one of the 39 implants with apical lesions in this study were treated within 2 years of implant placement. The remaining 8 implants were treated between 2 and 11 years after implant placement. However, careful review of the radiographs of these 8 patients obtained in the first 2 years following implant placement revealed signs of radiolucencies in the areas where the apicoectomies were eventually performed. The apicoectomies were not performed on these eight patients at that time due to lack of clinical symptoms, pain, and/or patient compliance.

The 1 implant (4.0 × 18-mm TiUnite Mk III) with an apical lesion in this study that failed despite undergoing the implant apicoectomy procedure was placed in the anterior mandible in type 4 bone in a 53-year-old man who reported smoking at least 2 packs of cigarettes per day. This implant was 1 of 6 implants that supported an acrylic resin screw-retained temporary prosthesis. The implant was immediately loaded using the same protocol as the other 5 mandibular implants. The implant was placed into an immediate extraction site where a periodontally hopeless tooth had previously existed. The implant was eventually removed 7 months after implant placement and 6 months after the implant apicoectomy procedure. The other 5 implants in the mandible remain osseointegrated.

Aggressive management of the affected site is required if the pathologic process is to be resolved and the implant salvaged. Patient noncompliance with the recommended treatment has led to further bone destruction and implant failure. Surgical intervention is aimed at the removal of any inflamed granulation tissue and the involved portion of the implant. Resection of the implant facilitates complete removal of the lesion while leaving enough integrated implant length to support the restoration. It is crucial to treat the implant before the lesion spreads coronally. Once the lesion reaches the portion of the implant that has an internal screw thread, an implant apicoectomy is no longer possible, since a channel would then exist between the oral cavity and the osseous environment for bacterial migration. Other treatment options, such as antibiotics[11,14,17,18] and detoxification,[15–18] are recommended prior to implant removal.

CONCLUSION

An intraoral dissection approach was performed in 35 patients that exhibited radiolucent lesions at the apex of 39 osseointegrated implants. This procedure resulted in resolution of the osseous lesion in 38 of 39 implants, with follow-up for as long as 15 years (average, 4.54 years).

ACKNOWLEDGMENTS

The authors would like to thank the staff at Prosthodontics Intermedica for their kind and very gentle treatment of the patients; Mrs Joann Coughlan and J. Neil Della Croce for data recovery/entry; Dr Sow-Yeh Chen and Temple Oral Pathology Laboratory for microscopic diagnoses of the biopsies; and Mr Ron Dove and Dr Neil Park of Nobel Biocare for years of technical support.

REFERENCES

1. Brånemark P-I, Zarb GA, Albrektsson T (eds). Tissue-Integrated Prostheses: Osseointegration in Clinical Dentistry. Chicago: Quintessence, 1985.
2. van Steenberghe D, Yoshida K, Papaioannou W, Bollen CM, Reybrouck G, Quirynen M. Complete nose coverage to prevent airborne contamination via nostrils is unnecessary. Clin Oral Implants Res 1997;8:512–516.
3. Balshi TJ. Preventing and resolving complications with osseointegrated patients. Dent Clin North Am 1989;33:821–868.
4. Friberg B, Jemt T, Lekholm U. Early failures in 4,641 consecutively placed Brånemark dental implants: A study from stage 1 surgery to the connection of the completed prosthesis. Int J Oral Maxillofac Implants 1991;6:142–146.
5. Balshi TJ, Pappas CE, Wolfinger GJ, Hernandez RE. Management of an abscess around the apex if a mandibular root form implant: Clinical report. Implant Dent 1994;3:81–85.
6. Sussman HI, Moss SS. Localized osteomyelitis secondary to endodontic implant pathosis. A case report. J Periodontol 1993;4:306–310.
7. Meffert RM. How to treat ailing and failing implants. Implant Dent 1992;1:25–33.
8. Mombelli A, Buser D, Lang NP. Colonization of osseointegrated titanium implants in edentulous patients. Early results. Oral Microbiol Immunol 1988;3:113–120.
9. Becker W, Becker BE, Newman MG, Nyman S. Clinical and microbiologic findings that may contribute to dental implant failure. Int J Oral Maxillofac Implants 1990;5:31–38.
10. Meffert RM, Langer B, Fritz ME. Dental implants: A review. J Periodontol 1992;63:859–870.
11. Mombelli A, Lang NP. Antimicrobial treatment of peri-implant infections. Clin Oral Implants Res 1992;3:162–168.
12. Lozada JL, James, RA, Boskovic M, Cordova C, Emanuelli S. Surgical repair of peri-implant defects. J Oral Implantol 1990; 16:42–46.
13. Riser GM, Nevins M. The implant periapical lesion: Etiology, prevention, and treatment. Compend Contin Educ Dent 1995;16:768–777.
14. Piattelli A, Scarano A, Piattelli M. Abscess formation around the apex of a maxillary root form implant: Clinical and microscopical aspects. A case report. J Periodontol 1995;66:899–903.
15. Dennison DK, Huerzeler MB, Quinones C, Caffesse RG. Contaminated implant surfaces: An in vitro comparison of implant surface coating and treatment modalities for decontamination. J Periodontol 1994:65;942–948.
16. Zablotsky MH, Diedrich DL, Meffert RM. Detoxification of endotoxin-contaminated titanium and hydroxyapatite-coated surfaces utilizing various chemotherapeutic and mechanical modalities. Implant Dent 1992;1:154–158.
17. Ayangco L, Sheridan PJ. Development and treatment of retrograde peri-implantitis involving a site with a history of failed endodontic and apicoectomy procedures: A series of reports. Int J Oral Maxillofac Implants 2001;16:412–417.

COPYRIGHT © 2007 BY QUINTESSENCE PUBLISHING CO, INC. PRINTING OF THIS DOCUMENT IS RESTRICTED TO PERSONAL USE ONLY. NO PART OF THIS ARTICLE MAY BE REPRODUCED OR TRANSMITTED IN ANY FORM WITHOUT WRITTEN PERMISSION FROM THE PUBLISHER

18. Park SH, Sorensen WP, Wang HL. Management and prevention of retrograde peri-implant infection from retained root tips: Two case reports. Int J Periodontics Restorative Dent 2004; 24:422–433.
19. Oh TJ, Yoon J, Wang HL. Management of the implant periapical lesion: a case report. Implant Dent 2003;12:41–46.
20. Scarano A, Di Domizio P, Petrone G, Iezzi G, Piattelli A. Implant periapical lesion: A clinical and histologic case report. J Oral Implantol 2000;26:109–113.
21. Shaffer MD, Juruaz DA, Haggerty PC. The effect of peri-radicular endodontic pathosis on the apical region of adjacent implants. Oral Surg Oral Med Oral Pathol Oral Radiol Endod 1998;86:578–581.
22. Mombelli A. Etiology, diagnosis and treatment considerations in peri-implantitis. Curr Opin Periodontol 1997;4:127–136.
23. McAllister BS, Masters D, Meffert RM. Treatment of implants demonstrating periapical radiolucencies. Pract Periodontics Aesthet Dent 1992;4:37-41.
24. Eriksson RA, Adell R. Temperatures during drilling for the placement of implants using the osseointegration technique. J Oral Maxillofac Surg 1986;44:4–7.
25. Watanabe F, Tawada Y, Komatsu S, Hata Y. Heat distribution in bone during preparation of implant sites: Heat analysis by real-time thermography. Int J Oral Maxillofac Implants 1992;7:212–219.
26. Meffert RM. Periodontics and peri-implantitis: One and the same. Prac Periodontics Aesthet Dent 1993;5(9):79–80, 82.

COPYRIGHT © 2007 BY QUINTESSENCE PUBLISHING CO, INC. PRINTING OF THIS DOCUMENT IS RESTRICTED TO PERSONAL USE ONLY. NO PART OF THIS ARTICLE MAY BE REPRODUCED OR TRANSMITTED IN ANY FORM WITHOUT WRITTEN PERMISSION FROM THE PUBLISHER
