# Supplementary material for: A novel histopathological classification of implant periapical lesion: A systematic review and treatment decision tree
Source: PLoS One. 2022 Dec 22;17(12):e0277387. doi: 10.1371/journal.pone.0277387 (PMC9778521; doi:10.1371/journal.pone.0277387)
Supplement: S1 File — (ZIP) [file pone.0277387.s001.zip › support files/Included study/Casado 2008.pdf]

Immediate Dental Implant Failure Associated With Nasopalatine Duct Cyst

Priscila L. Casado, MScD,* Marcelo Donner, DDS,† Bernardo Pascarelli, BSc,‡ Clebio Derocy, DDS,§ Maria Eugênia L. Duarte, PhD,|| and Eliane P. Barboza, MScD, DScD¶

This case report presents an analysis of the clinical, radiographic, and histological features of a peri-implant lesion around an implant placed immediately after extraction of a tooth with a periapical lesion. A 52-year-old man received an immediate implant (3.75 × 11.5 mm²) placed in the anterior region of the maxilla. Three years after implant placement, the patient presented with swelling in the anterior portion of the maxilla. Radiographic examination showed a well-circumscribed radiolucency around the implant. The implant and the lesion were removed and fixed in 10% buffered formalin and processed. Histological analysis showed 3 types of epithelium: respiratory, cuboidal, and non-keratinized stratified squamous. In the cyst wall peripheral nerves, arteries, veins, and chronic inflammation were present. The diagnosis was nasopalatine duct cyst. We concluded that the nasopalatine duct cyst can develop in association with dental implants. Clinically, the lesion is similar to the classical nasopalatine duct cyst. Histological analysis should be mandatory in all cases of peri-implant lesions and in all dental periapical lesions before immediate implant placement. (Implant Dent 2008;17:169–175)

Key Words: dental implant failure, peri-implant disease, histological analysis, immediate implant

Immediate postextraction implant placement is a well-accepted protocol due to potential for the preservation of aesthetics, shorter total treatment time, maintenance of socket walls, reduced surgical time, and better actual implant placement.[1] This technique achieves osseointegration with bone healing around dental implants and allows direct anchorage of the implant without the growth of fibrous tissue at the bone–implant interface.[2] Although a high dental implant success rate has been reported,[3–5] several studies demonstrated dental implant failures.[6–9]

The etiology and mechanism of implant failure are multifactorial, and the periapical implant lesion has been reported as one possible cause for dental implant failures.[8,10,11] According to Tözüm *et al*,[9] the periapical implant lesion often occurs because of overloading and excessive tightening of the dental implant, bone overheating during surgical procedure, fenestration of the vestibular alveolar bone, presence of preexisting bone pathology, contamination of the dental implant surface, preexisting microbial pathology, or poor bone quality. Novaes and Novaes[12] reported that, in immediate implant placement for replacement of teeth with periapical lesions, success can be achieved if certain preoperative and postoperative measures are followed. These include antibiotic administration, meticulous cleaning, and alveolar debridement, before surgical procedure.

This case report presents an analysis of the clinical, radiographic, and histological features of a peri-implant lesion around an implant placed immediately after extraction of a tooth that had a periapical lesion.

Case Description

A 52-year-old man underwent the extraction of the right maxillary central incisor because of root resorption and a periapical lesion (Fig. 1). The patient received amoxicillin 500 mg 24 hours before surgery, 3 times daily for 7 days. After extraction, careful curettage and alveolar debridement was performed to remove any trace of infected or inflamed tissue. The patient underwent the placement of one 3.75 × 11.5 mm² immediate implant (Titanium Fix Implants A.S.; São José dos Campos, São Paulo, Brazil). During the surgery the implant presented primary stability and a satisfactory position. The patient was asked to rinse with an antimicrobial mouth rinse (chlorhexidine 0.12%) for plaque control. Seven days after surgery the sutures were removed and healing was uneventful. The patient was not reliable for the follow-up appointments.

Three years after immediate implant placement, the patient returned for implant exposure and prosthetic treatment. However, a swelling in the anterior portion of the maxilla was observed. No pain or purulence was related. Radiographic evaluation showed a large well-circumscribed periapical radiolu-

*Master of Science in Dentistry, Rio de Janeiro Federal University, Rio de Janeiro, Brazil; Brazilian Institute of Periodontology, Rio de Janeiro, Brazil.
†Oral Implantologist, Brazilian Institute of Periodontology, Rio de Janeiro, Brazil.
‡Graduate Student, Rio de Janeiro Federal University, Rio de Janeiro, Brazil.
§Graduate Student, Federal Fluminense University, Rio de Janeiro, Brazil.
||Pathology, Rio de Janeiro Federal University, Rio de Janeiro, Brazil.
¶Doctor of Science in Dentistry, Periodontology, AAP-Diplomate, Brazilian Institute of Periodontology, Rio de Janeiro, Brazil; Federal Fluminense University, Rio de Janeiro, Brazil.

ISSN 1056-6163/08/01702-169
Implant Dentistry
Volume 17 • Number 2
Copyright © 2008 by Lippincott Williams & Wilkins
DOI: 10.1097/ID.0b013e3181776c52

Copyright © Lippincott Williams & Wilkins. Unauthorized reproduction of this article is prohibited.

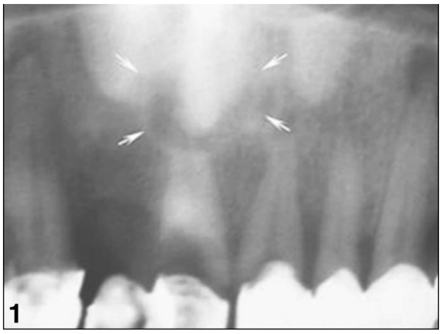

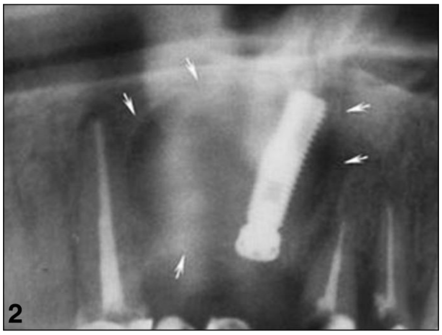

Fig. 1. Periapical radiolucent lesion and root resorption (arrows) were observed at the apical region of the central incisor.

Fig. 2. Well-circumscribed radiolucency around the implant (arrows). Note that the implant was lodged inside the lesion. The lesion growth led the implant to its original position.

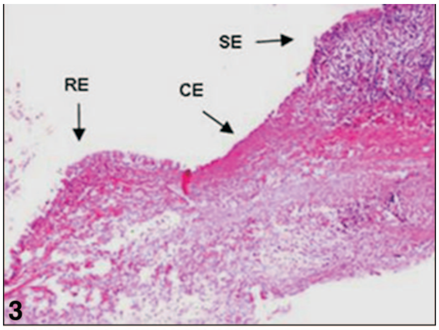

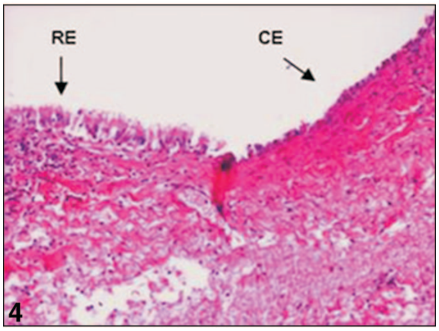

Fig. 3. Histopathological aspect with three epithelium types: respiratory (RE), cuboidal (CE), and non-keratinized stratified squamous (SE). Hematoxylin and eosin 4×.

Fig. 4. Respiratory (RE) and cuboidal (CE) epithelium lining the cyst wall. Hematoxylin and eosin 10×.

cency of 1.5 cm width surrounding the dental implant. The implant position was not compatible with its original position (Fig. 2).

The first step of the treatment plan was to remove the peri-implant lesion for histological analysis. An inverse bevel incision was made along the crest of the ridge. Buccal releasing incisions were made extending into the alveolar mucosa at the distal of the tooth 8 and the mesial at the tooth 11. Mucoperiosteal flap was elevated. Complete loss of a labial cortical plate and a large bone defect were observed. The lesion (1.5 cm width) was removed with the dental implant lodged inside it. Debridement of the tissues at the defect site was followed by the irrigation with sterile saline solution. The bone defect was filled with an organic bovine matrix graft (Genius/Baumer S.A., São Paulo, Brazil) and the wound closure was obtained with 3-0 silk sutures. The patient was prescribed 0.2% chlorhexidine gluconate and instructed to rinse gently twice daily for 3 weeks. Patient was also prescribed antibiotics (amoxicillin 500 mg, 3 times daily) for 7 days. Seven days after surgery, sutures were removed and healing was uneventful. The follow-up visits included routine intraoral examinations and professional plaque control.

The lesion was fixed in 10% buffered formalin and sections (5 μm) of paraffin embedded tissue were cut and stained with hematoxylin and eosin. Microscopic examination revealed a cyst lined by 3 types of epithelium: respiratory, cuboidal, and non-keratinized stratified squamous (Figs. 3 and 4). In the cyst wall a dense lymphocytic infiltrate, veins, and peripheral nerves were present. These histological features associated with the site of the lesion and the radiographic aspects diagnosed the lesion as a nasopalatine duct cyst.

Discussion

Immediate implantation is indicated for replacing teeth with pathologies such as caries or fractures.[13] This procedure according to Peñarrocha *et al*[13] can be carried out on extraction teeth sites with chronic apical lesions that are not likely to improve with endodontic treatment and apical surgery. These authors stated that only the existence of an acute periapical inflammatory process constitutes an absolute contraindication to immediate implantation.[12,14] As stated by Novaes *et al*,[15] the placement of immediate implants in chronically infected sites may not necessarily be contraindicated if appropriate clinical procedures like antibiotic administration, meticulous cleaning, and alveolar debridement are performed before an implant surgical procedure. Lindeboom *et al*[16] performed the first controlled comparison between immediate and delayed placement of implants for replacement of teeth with periapical lesions. This last study supported the feasibility of the immediate placement of implants in infected sites.

Although the protocol related in the literature for immediate placement in areas with dental periapical lesions was followed, in this reported case, success has not been achieved. This case report has demonstrated that this procedure can present some risks to the implant survival due to the possibility of implantation in a supposed infected chronic lesion that could be a development lesion or more aggressive lesions such as a tumor or recidivate cysts. This is the first description of a peri-implant cyst diagnosed as a nasopalatine duct cyst.

The nasopalatine duct cyst (NPDC) is considered to be the most common nonodontogenic cyst of the oral cavity. A review of 295 cases involving nonodontogenic tumors performed by Daley *et al*[17] found 73% to be NPDC. The NPDC, first described by Meyer in 1914,[18] can form within the incisive canals behind the alveolar process of the central incisors of the palatine bone or in the soft tissue of the palate where the canal opens.[19,20]

The cause or origin of the NPDC has been debated in the literature. Initially, it was thought to be originated from the trapping of epithelium during fusion of the embryological process.[21] This concept has been rejected, and today it is thought to develop from oronasal ducts present within the incisive canals.[19,22] Precipitating factors in the development of the NPDC have been reported to be trauma, infection, and spontaneous occurrence.[21] Nasopalatine duct cysts are most often detected in patients between the fourth to sixth decades of life.[22] The cysts often present as asymptomatic swellings of the palate but

Copyright © Lippincott Williams & Wilkins. Unauthorized reproduction of this article is prohibited.

they can present with painful swelling or drainage.[23] Radiological findings include a well-demarcated cystic structure in a round, ovoid, or heart shape presenting in the midline of the maxilla.[20,24]

The diagnosis of the NPDC relies only on histological analysis; and, as such, it depends on the clinicians to submit all surgically excised peri-implant tissue lesions for examination. Therefore, despite this study being the first description (to the best of our knowledge) of NPDC forming a peri-implant lesion, other lesions diagnosed as chronic pathologies, but not submitted to histological analysis, could be NPDC.

Tözüm *et al*[9] related that the inactive form of peri-implant lesion should be periodically monitored and does not need any surgical treatment. However, if the lesion is active (pain and/or pus formation) they indicate debridement and an antibiotic therapy to achieve a successful eradication of bacterial contamination. This reported case showed an implant placement in an area of an inactive lesion. Although primary stability, at the time of implant insertion, was achieved, the development of the lesion influenced the implant osseointegration. In addition, the implantation in the preexisting lesion could facilitate the secondary infection into the bone allowing the osteomyelitis development.

Surgical procedures including the removal or the resection of the contaminated dental implant was previously suggested to avoid a possible osteomyelitis[10,25] and surgical removal of the dental implants should be performed whether the implants were mobile or not.[10,26] On the contrary, if the dental implant has a stable osseointegration and the periapical lesion does not risk the adjacent regions, it was recently suggested that the removal of the implant should be avoided. However, a complete debridement as well as histological analysis should be performed.

Conclusion

The case presented in this article demonstrates that a nasopalatine duct cyst can develop in association with dental implants. Clinically, the lesion is similar to the classical NPDC. A detailed diagnosis, systematic treatment plan, and appropriate treatment procedures would minimize the occurrence of dental implant periapical lesions in the immediate implant. Histological analysis should be mandatory in all cases of peri-implant lesions and in all dental periapical lesions before immediate implant placement. Future studies are necessary to achieve better knowledge about the peri-implant lesion and its consequences.

Disclosure

All co-authors claim to have no financial interest in any of the products or companies mentioned in this article.

References

1. Lazzara RJ. Immediate implant placement into extraction sites: Surgical and restorative advantages. *Int J Periodontics Restorative Dent*. 1989;9:332-343.
2. Branemark PI, Hansson BO, Adell R, et al. Osseointegrated implants in the treatment of the edentulous jaws: Experience from a 10-year period. *Scand J Plast Reconstr Surg*. 1977;16:1-132.
3. Adell R, Lekholm U, Rockler B. A 15-year study of osseointegrated implants in the treatment of the edentulous jaw. *Int J Oral Surg*. 1981;10:387-416.
4. Albrektsson T. A multicenter report on osseointegrated oral implants. *J Prosthet Dent*. 1988;60:75-84.
5. Misch CE, Steignga J, Barboza E, et al. Short dental implants in posterior partial edentulism: A multicenter retrospective 6-year case series study. *J Periodontol*. 2006;77:1340-1347.
6. Brisman DL, Brisman AS, Moses MS. Implant failures associated with asymptomatic endodontically treated teeth. *J Am Dent Assoc*. 2001;132:191-195.
7. Esposito M, Hirsh J, Lekholm U, Thomsen P. Differential diagnosis and treatment strategies for biologic complication and failing oral implants. *Int J Oral Maxillofac Implants*. 1999;14:473-490.
8. Ross-Jansaker AM, Renvert S, Egelberg J. Treatment of peri-implant infections: A literature review. *J Clin Periodontol*. 2003;30:467-485.
9. Tözüm TF, Sençimen M, Ortaglu K, et al. Diagnosis and treatment of a large periapical implant lesion associated with adjacent natural tooth: A case report. *Oral Surg Oral Med Oral Pathol Oral Radiol Endod*. 2006;101:e132-e138.
10. Piattelli A, Scarano A, Piattelli M, et al. Implant periapical lesion: Clinical, histological and histochemical aspects. *Int J Periodontics Restorative Dent*. 1998;18:181-187.
11. Sussman HI. Periapical implant pathology. *J Oral Implantol*. 1998;24:133-138.
12. Novaes-Junior AB, Novaes AB. Immediate implants placed into infected sites: A clinical report. *Int J Oral Maxillofac Implants*. 1995;10:609-613.
13. Peñarrocha M, Uribe R, Balaguer J. Immediate Implants after extraction: A review of the current situation. *Med Oral*. 2004;9:234-242.
14. Novaes-Junior AB, Novaes AB. Soft tissue management for primary closure in guided bone regeneration: Surgical technique and case report. *Int J Oral Maxillofac Implants*. 1997;12:84-87.
15. Novaes-Junior AB, Vidigal GM, Novaes AB, et al. Immediate implants placed into infected sites: A histomorphometric study in dogs. *Int J Oral Maxillofac Implants*. 1998;13:422–427.
16. Lindeboom JA, Tjiook Y, Kroon HM. Immediate placement of implants in periapical infected sites: A prospective randomized study in 50 patients. *Oral Surg Oral Med Oral Pathol Oral Radiol Endod*. 2006;101:705-710.
17. Daley TD, Wysocki GP, Pringle GA. Relative incidence of odontogenic tumors and oral and jaw cysts in a Canadian population. *Oral Surg Oral Med Oral Pathol*. 1994;77:276-280.
18. Meyer AW. A unique supernumerary paranasal sinus directly above the superior incisors. *J Anatomy*. 1914;48:118-129.
19. Gnanasekhar JD, Walvekar SV, Al-Kandari AM, et al. Misdiagnosis and mismanagement of a nasopalatine duct cyst and its corrective therapy: A case report. *Oral Surg Oral Med Oral Pathol Oral Radiol Endod*. 1995;80:465-470.
20. Elliott KA, Franzese CB, Pitman KT. Diagnosis and Surgical Management of Nasopalatine Duct Cysts. *Laryngoscope*. 2004;114:1336-1340.
21. Albayram MS, Sciubba J, Zinreich J. Radiology quiz case: Nasopalatine duct cyst. *Arch Otolaryngol Head Neck Surg*. 2001;127:1283-1285.
22. Swanson KS, Kaugars GE, Gunsolley JC. Nasopalatine duct cyst: An analysis of 334 cases. *J Oral Maxillofac Surg*. 1991;49:268-271.
23. Vasconcelos RF, Ferreira de Aguiar MC, Castro WH, et al. Retrospective analysis of 31 cases of nasopalatine duct cyst. *Oral Dis*. 1999;5:325-328.
24. Staretz LR, Brada BJ, Schott TR. Well-defined radiolucent lesion in the maxillary anterior region. *J Am Dent Assoc*. 1990;120:335-336.
25. Reiser GM, Nevins M. The periapical lesion: Etiology, prevention and treatment. *Compendium Contin Educ Dent*. 1995;16:768-777.
26. Oh TJ, Yoon J, Wang HL. Management of the implant periapical lesion: A case report. *Implant Dent*. 2003;12:41-46.

Reprint requests and correspondence to:
Priscila Ladeira Casado, MScD
Av. Presidente Wilson 165 – 810 CEP.:20030-020
Rio de Janeiro, Brazil
Phone: 55-21-22206940
Fax: 55-21-22206706
E-mail: priscilalcasado@terra.com.br

Copyright © Lippincott Williams & Wilkins. Unauthorized reproduction of this article is prohibited.

Abstract Translations

GERMAN / DEUTSCH

AUTOR(EN): Priscila L. Casado, MScD, Marcelo Donner, DDS, Bernardo Pascarelli, BSc, Clebio Derocy, DDS, Maria Eugênia L. Duarte, PhD, Eliane P. Barboza, MScD, DScD. *Korrespondenz an: Priscila Ladeira Casado, MScD, Av. Presidente Wilson 165-810 CEP. 20030-020, Rio de Janeiro. Telefon: 55-21-22206940, Fax: 55-21-22206706, e-mail: priscilalcasado@terra.com.br*

Unmittelbares Versagen eines Zahnimplantats in Verbindung mit dem Auftreten einer Nasopalatinalzyste

ZUSAMMENFASSUNG: Die vorliegende Fallstudie unternimmt eine Analyse der klinischen, radiographischen und histologischen Eigenheiten einer im Implantatumfeld auftretenden Läsion bei einem Implantat, das unmittelbar nach Extraktion eines Zahns mit einer periapikalen Läsion eingepflanzt wurde. Einem 52 Jahre alten Mann wurde im vorderen Bereich des Oberkiefers ein sofortiges Implantat (3,75 mm x 11,5 mm) eingepflanzt. Drei Jahre nach Implantierung trat bei dem Patienten eine Schwellung des vorderen Bereichs des Oberkiefers auf. Der Bereich um das Implantat herum wies bei röntgenographischer Untersuchung eine klar umrissene Aufhellung auf. Implantat und Läsion wurden entfernt, in 10%-gepuffertes Formalin eingelegt und weiter verarbeitet. Die histologische Analyse weist drei verschiedene Epitheltypen aus: Atmungsbedingte, würfelförmige und nicht keratinisierte geschichtete schuppenartige Epithel. In der Zystenwand fanden sich periphere Nerven, Arterien, Venen und chronische Entzündungsherde. Die zu stellende Diagnose lautete auf Nasopalatinalzyste. Wir schlossen, dass sich eine Nasopalatinalzyste in Verbindung mit Zahnimplantierungen entwickeln kann. Aus klinischer Sicht ähnelt die Läsion sehr stark der klassischen Nasopalatinalzyste. Eine histologische Analyse sollte zwingend für all jene Fälle durchgeführt werden, bei denen eine sofortige Implantatsetzung vorgesehen ist und Läsionen im das Implantat umgebenden Bereich oder periapikale Zahnläsionen festgestellt werden.

SCHLÜSSELWÖRTER: Versagen des Zahnimplantats; Erkrankung des Gewebes um das Implantat herum; histologische Analyse; sofortiges Implantat.

SPANISH / ESPAÑOL

AUTOR(ES): Priscila L. Casado, MScD, Marcelo Donner, DDS, Bernardo Pascarelli, BSc, Clebio Derocy, DDS, Maria Eugênia L. Duarte, PhD, Eliane P. Barboza, MScD, DScD. *Correspondencia a: Priscila Ladeira Casado, MScD, Av. Presidente Wilson 165-810 CEP.:20030-020, Rio de Janeiro. Teléfono: 55-21-22206940, Fax: 55-21-22206706, Correo electrónico: priscilalcasado@terra.com.br*

Falla inmediata del implante dental asociado con un quiste en el conducto nasopalatino

ABSTRACTO: Este informe de un caso presenta un análisis de las características clínicas, radiográficas e histológicas de una lesión periimplante alrededor de un implante colocado inmediatamente después de la extracción de un diente con una lesión periapical. Un hombre de 52 años recibió un implante inmediato (3,75 mm x 11,5 mm) colocado en la región anterior del maxilar. Tres años después de la colocación del implante el paciente presentó inflamación en la parte anterior del maxilar. Un examen radiográfico demostró una radiolucidez circunscripta alrededor del implante. Se sacaron el implante y la lesión y se fijaron en formalina tamponada al 10% y fueron procesados. El análisis histológico demostró tres tipos de epitelios: respiratorio, cuboides y escamoso estratificado no-queratinizado. En la pared del quiste, se observaron nervios periféricos, arterias, venas e inflamación crónica. El diagnóstico fue quiste del conducto nasopalatino. Llegamos a la conclusión de que un quiste del conducto nasopalatino (NPDC por sus siglas en inglés) puede aparecer asociado a un implante dental. Clínicamente, la lesión es similar al NPDC clásico. El análisis histológico debería ser obligatorio en todos los casos de lesiones periimplante y en todas las lesiones dentales periapicales antes de la colocación de un implante inmediato.

PALABRAS CLAVES: falla del implante dental, enfermedad periimplante, análisis histológico, implante inmediato.

PORTUGUESE / PORTUGUÊS

AUTOR(ES): Priscila L. Casado, Mestre em Odontologia Marcelo Donner, Cirurgião-Dentista, Bernardo Pascarelli, Bacharel em Ciência, Clebio Derocy, Cirurgião-Dentista, Maria Eugênia L. Duarte, PhD, Eliane P. Barboza, Mestre em Odontologia, Doutora em Odontologia. *Correspondência para: Priscila Ladeira Casado, MScD, Av. Presidente Wilson 165 – 810 CEP.:20030-020, Rio de Janeiro. Telefone: 55-21-22206940, Fax: 55-21-22206706, e-mail: priscilalcasado@terra.com.br*

Falha Imediata de Implantes Dentários Associada a Cisto do Duto Nasoplatino

RESUMO: Este relato de caso apresenta uma análise das características clínicas, radiográficas e histológicas de uma lesão de periimplante em torno de um implante colocado imediatamente após a extração de um dente com lesão periapical. Um homem de 52 anos recebeu um implante imediato (3.75 mm x 11.5 mm) colocado na região anterior da maxila. Três dias após a colocação do implante, o paciente apresentou inchaço na porção anterior da maxila. O exame radiográfico mostrou uma radiolucência bem circunscrita em torno do implante. O implante e a lesão foram removidos e fixados em formalina com acidez constante a 10% e processados. A análise histológica mostrou três tipos de epitélio: escamoso estratificado respiratório, cuboidal e não-queratinado. Na

Copyright © Lippincott Williams & Wilkins. Unauthorized reproduction of this article is prohibited.

parede do cisto estavam presentes nervos periféricos, artérias, veias e inflamação crônica. O diagnóstico foi cisto do duto nasoplatino. Concluímos que o cisto do duto nasoplatino (NPDC) pode desenvolver-se em associação com implantes dentários. Clinicamente, a lesão é semelhante ao clássico NPDC. A análise histológica deveria ser obrigatória em todos os casos de lesões de periimplante antes da colocação imediata do implante.

PALAVRAS-CHAVE: Falha no implante dentário; doença do periimplante; análise histológica; implante dentário.

RUSSIAN / РУССКИЙ

АВТОРЫ: Priscila L. Casado, магистр стоматологии, Marcelo Donner, доктор стоматологии, Bernardo Pascarelli, бакалавр наук, Clebio Derocy, доктор стоматологии, Maria Eugênia L. Duarte, доктор философии, Eliane P. Barboza, магистр стоматологии, доктор стоматологии. *Адрес для корреспонденции: Priscila Ladeira Casado, MScD, Av. Presidente Wilson 165 – 810, CEP.:20030–020 - Rio de Janeiro. Факс: 55–21-22206706 Телефон: 55–21-22206940 Адрес электронной почты: priscilalcasado@terra.com.br*

Неудачный исход немедленной имплантации в связи с образованием кисты резцового (носонебного) канала

РЕЗЮМЕ. Описание данного случая представляет собой анализ клинических, рентгенографических и гистологических характеристик новообразования вокруг имплантата, установленного сразу после удаления зуба с периапикальным новообразованием. Мужчине в возрасте 52 лет немедленно после удаления зуба установили имплантат (3,75 мм x 11,5 мм) в передней области верхней челюсти. Спустя три года после установки имплантата пациент пришел на прием с опухолью в передней области верхней челюсти. Рентгенографическое обследование показало контурированное просветление вокруг имплантата. Имплантат и новообразование были удалены, помещены в 10-процентный буферный формалин и исследованы. Гистологический анализ показал наличие трех типов эпителия: респираторного, кубовидного и некератинизированного многослойного плоского. В стенке кисты присутствовали периферийные нервы, артерии, вены, а также наблюдался хронический воспалительный процесс. Диагноз – киста резцового (носонебного) канала. Мы пришли к выводу, что киста резцового (носонебного) канала может развиться в связи с зубными имплантатами. С клинической точки зрения это новообразование подобно классической кисте резцового (носонебного) канала. Гистологический анализ должен обязательно проводиться во всех случаях околоимплантатных новообразований, а также во всех случаях зубных периапикальных новообразований до немедленной установки имплантата.

КЛЮЧЕВЫЕ СЛОВА: неудачный исход имплантации; околоимлантатное заболевание, гистологический анализ, имплантат для немедленной имплантации

TURKISH / TÜRKÇE

YAZARLAR: Priscila L. Casado, MScD, Diçs Hekimi Marcelo Donner, Bernardo Pascarelli, BSc, Diçs Hekimi Clebio Derocy, Dr. Maria Eugênia L. Duarte, Eliane P. Barboza, MScD, DScD. *Yazýþma için: Priscila Ladeira Casado, MScD, Av. Presidente Wilson 165 – 810 CEP.:20030–020, Rio de Janeiro Brezilya. Telefon: 55–21-22206940, Faks: 55–21-22206706, e-posta: priscilalcasado@terra.com.br*

Burun-Damak (Nazo-palatin) Kanalýnda Kistten Kaynaklanan Hemen Yükleme Türü Ýmplant Baþarýsýzlýðý

ÖZET: Bu olgu raporu, periapik lezyonu olan bir diþin çekilmesinden hemen sonra yüklenen bir implantýn etrafýndaki bir peri-implant lezyonun klinik, radyografik ve histolojik özelliklerinin bir analizini sunmaktadýr. 52 yaþýndaki erkek hastaya maksillanýn anterior bölgesinde hemen yükleme ile bir implant (3,75 mm x 11,5 mm) uygulandý. Ýmplant yerleþtirilmesinden üç yýl sonra hasta, maksillanýn anterior bölgesinde bir þiþlik ile bize baþvurdu. Radyografik inceleme sonucunda, implantý iyice çevreleyen bir radyo geçirgen alan gözlemlendi. Ýmplant ve lezyon çýkartýlarak %10 tamponlu formalinde sabitleþtirildi ve iþlemden geçirildi. Histolojik analiz üç tür epitelyum gösterdi: respiratuvar, küboid ve keratinize olmayan tabakalaþmýþ skuamoz. Kist duvarýnda periferik sinirler, arterler, venler ve kronik enflamasyon vardý. Olguya burun-damak (nazo-palatin) kanal kisti (BDKK) tanýsý kondu. Burun-damak kanal kistlerinin dental implantlar ile iliþkili olarak geliþebileceði kanýsýna varýldý. Klinik açýdan lezyon, klasik BDKK olgusuna benzer. Hemen implant yüklemesi yapýlmadan önce tüm peri-implant lezyon ve tüm dental peri-apik lezyon olgularýnda histolojik analiz zorunlu tutulmalýdýr.

ANAHTAR KELİMELER: dental implant baþarýsýzlýðý; peri-implant hastalýðý; histolojik analiz; hemen implant yükleme.

Copyright © Lippincott Williams & Wilkins. Unauthorized reproduction of this article is prohibited.

JAPANESE / 日本語

鼻口蓋管嚢胞と関連した即時デンタルインプラント失敗症例

共同研究者氏名: プリシラ•L•カサド（Priscila L. Casado）MScD, マルセロ•ドナー（Marcelo Donner）DDS, ベルナルド•パスカレリ（Bernardo Pascarelli） BSc, クレビオ•デロシ（Clebio Derocy） DDS, マリア•ユージニア•L•デュアルテ（Maria Eugênia L. Duarte） PhD, エリアン•P•バルボザ（Eliane P. Barboza）MScD, DScD

研究概要: 当ケースレポートは 根尖病変で抜歯直後に埋入したインプラント周辺の病変について臨床、レントゲンさらに組織学的特徴を分析し報告するものである。52歳の男性患者は上顎前部に即時インプラント治療 (3.75 mm x 11.5 mm) を受けたが、インプラント埋入3年後に上顎前部に腫脹が発生した。レントゲンではインプラント周辺をすっかり囲む放射線透過状態が確認された。インプラントならびに組織損傷部位は除去し、10%に緩和したホルマリンに固定してプロセスした。組織学上分析では次の3種類の上皮組織が検証された： 呼吸、立方、非角化重層扁平である。また嚢胞壁には周辺神経、動脈、静脈とともに慢性的炎症が見られた。診断結果は鼻口蓋管嚢胞で、鼻口蓋管嚢胞 (NPDC) はデンタルインプラントと関連して発病の可能性があるという結論に達した。臨床学的には病変は一般的 NPDC と類似する。 インプラント周辺病変ならびにすべての歯周病変ケースでは 即時インプラント埋入前に組織学分析を必須とするべきである。

キーワード: デンタルインプラント失敗症例; インプラント周辺病変; 組織学的分析; 即時インプラント

ご質問の宛先: Priscila Ladeira Casado, MScD, Av. Presidente Wilson 165 – 810 CEP.:20030-020 - Rio de Janeiro
FAX: 55-21-22206706 電話: 55-21-22206940 電子メール: priscilalcasado@terra.com.br

CHINESE / 中国語

與鼻腭管囊腫相關的立即牙科植體失敗

作者：Priscila L. Casado, MScD；Marcelo Donner, DDS；Bernardo Pascarelli, BSc；Clebio Derocy, DDS；Maria Eugênia L. Duarte, PhD；Eliane P. Barboza, MScD, DScD

摘要：本病例報告提供根尖周圍病變牙齒在拔牙後立即植入植體的植體周圍病變的臨床、X 光照相術及組織學特性分析。一名 52 歲男性接受上頜前面區域的立即植體 (3.75 mm x 11.5 mm) 植入。植體植入三年後，患者的上頜前面部分出現腫脹。X 光攝影術檢查顯示，植體周圍出現完整的外切放射線透光。取出植體和病變，並以 10% 緩衝福馬林固定與處理。組織學分析顯示三種上皮類型：呼吸系統、骰骨及非角質化分層鱗片。囊胞壁出現根尖周圍神經、動脈、靜脈及慢性發炎。診斷為鼻腭管囊腫。我們的結論是：鼻腭管囊腫 (nasopalatine duct cyst，NPDC) 的發展可能和牙科植體有關。臨床上，此病變類似典型的 NPDC。所有病例在進行立即植體植入之前，有必要對其植體周圍病變及所有植體根尖周圍病變應進行組織學分析。

關鍵字：牙科植體失敗、植體周圍疾病、組織學分析、立即植體

通訊方式：Priscila Ladeira Casado, MScD, Av. Presidente Wilson 165 – 810 CEP.:20030-020 - Rio de Janeiro
傳真：55-21-2220670 電話：55-21-22206940 電郵信箱：priscilalcasado@terra.com.br

Copyright © Lippincott Williams & Wilkins. Unauthorized reproduction of this article is prohibited.

KOREAN / 한국어

상악골 비구개관 낭종과 연관된 즉각적 치과용 임플란트 실패

저자: 프리실라 엘 카사도(Priscila L. Casado), 치의과 석사(MScD), 마르첼로 도너(Marcelo Donner), 구강외과 의사(DDS), 버나르도 파스카렐리(Bernardo Pascarelli), 이학사(BSc), 클레비오 데로시(Clebio Derocy), 구강외과 의사(DDS), 마리아 유지니아 엘 듀아르트(Maria Eugênia L. Duarte), 박사(PhD), 엘리안 피 바르보자(Eliane P. Barboza), 치의과 석사 겸 특수 케어 치의학(MScD, DScD)

초록: 본 증례 보고서에는 치근단 병변이 있는 치아 적출 후 바로 식립된 임플란트 주위의 임플란트 주변 병변의 임상, 방사선 및 조직학적 기능 분석이 나타나 있다. 52세 남성은 상악골의 앞 부위에 식립된 즉시 임플란트 (3.75 mm x 11.5 mm)를 이식 받았다. 임플란트를 이식한 지 3년 후, 상악골의 앞 부위에 부종이 나타났다. 방사선 검사에서 임플란트 주위에 방사선 투과가 잘 되는 것으로 나타났다. 임플란트와 병변은 제거되어 10% 완충 포르말린에 고정하여 처리하였다. 조직학적 분석에서는 세 가지 종류의 상피, 즉 호흡, 입방, 비각질 중층 편평을 보였다. 낭종 벽에서 말초신경과 동맥, 정맥, 만성 감염이 있었다. 상악골 비구개관 낭종으로 진단되었다. 상악골 비구개관 낭종(NPDC)은 치과용 임플란트와 관련되어 발전할 수 있는 것으로 결론 지어졌다. 임상적으로 병변은 표준 NPDC와 유사하다. 따라서 즉시 임플란트 식립 전에는 임플란트 주위 병변의 모든 증례와 모든 치근단 병변 내 조직학적 분석이 필수이다.

키워드: 치과용 임플란트 실패; 임플란트 주위 질환; 조직학적 분석; 즉시 임플란트

연락처: 프리실라 엘 카사도(Priscila L. Casado), 치의과 석사(MScD), Av. Presidente Wilson 165 – 810
CEP.:20030-020 - Rio de Janeiro
팩스: 55-21-22206706 전화: 55-21-22206940 이메일: priscilalcasado@terra.com.br

Copyright © Lippincott Williams & Wilkins. Unauthorized reproduction of this article is prohibited.
