# Supplementary material for: A novel histopathological classification of implant periapical lesion: A systematic review and treatment decision tree
Source: PLoS One. 2022 Dec 22;17(12):e0277387. doi: 10.1371/journal.pone.0277387 (PMC9778521; doi:10.1371/journal.pone.0277387)
Supplement: S1 File — (ZIP) [file pone.0277387.s001.zip › support files/Included study/Chaffee 2001.pdf]

Periapical abscess formation and resolution adjacent to dental implants: A clinical report

Nancy R. Chaffee, BS, DDS, MS,[a] Kevin Lowden, DDS,[b] John C. Tiffee, DDS,[c] and Lyndon F. Cooper, DDS, PhD[d]
School of Dentistry, University of North Carolina at Chapel Hill, Chapel Hill, N.C.

Treatment planning for dental implants typically involves consideration of bone quality and quantity for implant support.[1] In addition, implant-tooth relationships are key aspects of esthetic and biomechanical restorative outcomes.[2] In the partially edentulous situation, uncontrolled periodontal disease on adjacent teeth is an unacceptable risk factor that could represent a source of pathogenic bacteria leading to peri-implant inflammation.[3] The proximity of adjacent tooth roots is of concern when implants are placed among natural teeth. The transection of a root apex during implant placement is a reported clinical risk. Interestingly, the 10-year outcome for 1 case indicated that endodontic therapy could be performed without loss of the implant or tooth root.[4]

Although it is clear that implant therapy must not proceed in the presence of periapical radiolucency or other signs or symptoms of irreversible pulpal disease, the relative pulpal health of teeth adjacent to an implant may represent an additional risk factor in implant treatment planning. In this clinical report, devitalization and pericementitis of a tooth adjacent to a recently placed dental implant was followed from 1994 to 1999. Significant periapical osteolysis involved the bone supporting the commercially pure (cp) titanium implant. The delayed resolution of this situation and the extended treatment period are presented.

CLINICAL REPORT

In June 1996, implants were placed in the areas of the right mandibular first and second molars. One week after implant surgery, the adjacent right mandibular second premolar was positive to percussion testing. However, the tooth responded positively to thermal and electronic pulp testing. A clinical impression of hyperocclusion associated with nocturnal bruxism was made; the tooth was adjusted to remove laterotrusive contacts, and the patient was dismissed. On reevaluation 35 days later, the patient

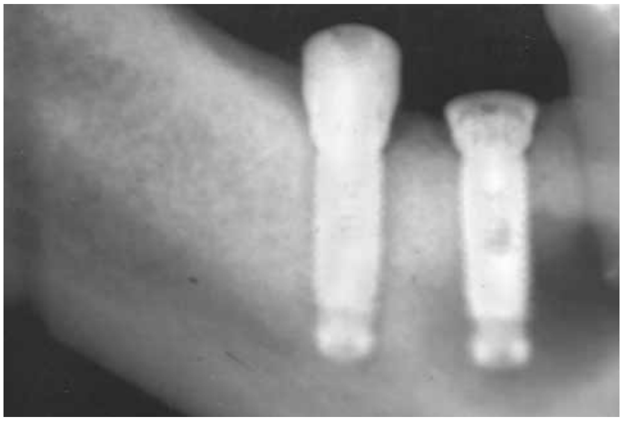

Fig. 1. Periapical radiograph taken August 1996. Radiolucency adjacent to implant was present before second stage surgery.

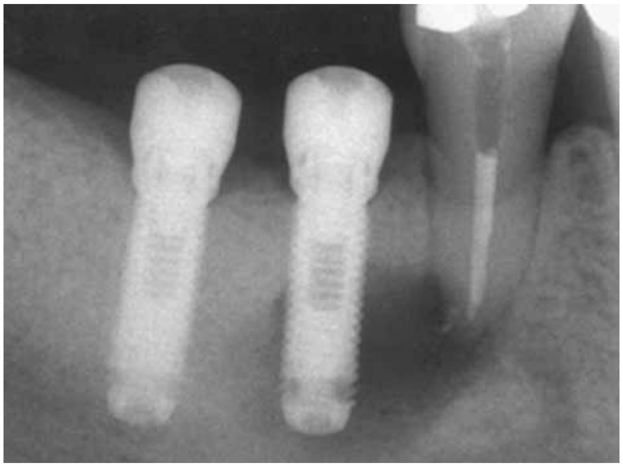

Fig. 2. Periapical radiograph taken December 1996 shows status of periapical radiolucency immediately after endodontic therapy.

again presented with a complaint of pain associated with the second premolar. Radiographs indicated a periapical radiolucency associated with the second premolar and radiolucency surrounding the dental implants (Fig. 1).

In December 1996, the patient returned to the clinic for second stage surgery. Clinical evaluation revealed purulent discharge from the gingival sulcus of

[a]Assistant Professor, Department of Prosthodontics, and Director, Graduate Prosthodontics.
[b]Graduate Prosthodontics Resident, Department of Prosthodontics.
[c]Assistant Professor, Department of Diagnostic Sciences and General Dentistry.
[d]Associate Professor, Department of Prosthodontics.
J Prosthet Dent 2001;85:109-12.

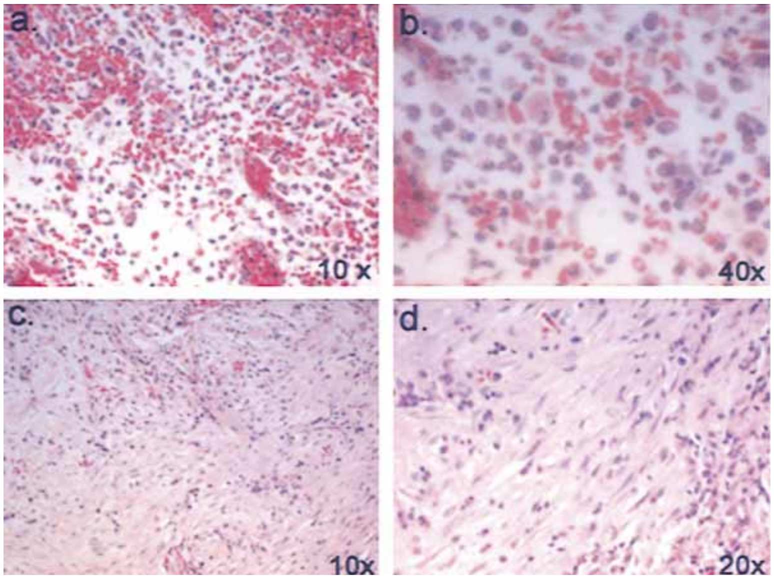

Fig. 3. Photomicrographs of biopsied soft tissue adjacent to implant and tooth. Neutrophils, macrophages, and small caliber vascular channels (**a,** ×10; **b,** ×40). Dense, fibrous tissue supporting patchy, mixed inflammatory cell infiltrate (**c,** ×10; **d,** ×20).

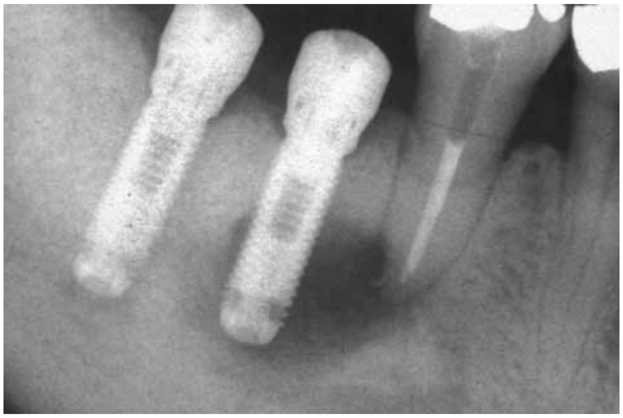

Fig. 4. Periapical radiograph taken May 1997 shows status of periapical radiolucency 6 months after endodontic therapy.

the second premolar. The second premolar was nonvital as determined by thermal and electronic pulp testing in comparison with the adjacent teeth. The dental implant in the right mandibular first molar position was immobile and asymptomatic. A periapical radiograph of the premolar region revealed significant periapical and peri-implant osteolysis (Fig. 2). A full-thickness mucoperiosteal flap was raised under local anesthetic to reveal a 5-mm facial cortical bone perforation involving the apical aspect of the fixture and the adjacent tooth root. The soft tissue lesion was curetted, the bone defect was filled with DFDBA (LifeNet, Virginia Beach, Va.), covered with a barrier membrane (Resolute, W.L. Gore & Associates, Elkton, Md.), and the flap was sutured in place. Amoxicillin (500 mg, 3 times daily) was prescribed for 10 days, and the patient was discharged.

Histologic evaluation of the biopsied tissue identified the presence of granulation tissue containing acute inflammatory cells, necrotic debris, and abundant hemorrhage bound by fibrous connective tissue infiltrated with chronic inflammatory cells (Fig. 3). Calcified debris was present in the granulation tissue in 1 area. Two weeks after surgical enucleation of the chronic abscess, endodontic therapy was initiated; it was completed 3 weeks later by conventional filling with gutta percha and AH26 cement (Dentsply DeTrey, Konstanz, Germany).

In May 1997, second stage surgery was again attempted. Healing abutments were placed, and radiographic evaluation revealed the continued presence of a radiolucency at the apical aspect of the implant and the second premolar (Fig. 4). In the absence of radiographic evidence of bone formation at the implant surface, permanent restoration of the implants was indefinitely postponed. Additional radiographs taken in August 1997 indicated the persistent absence of bone at the implant in the first molar site. On evaluation in December 1997, the second premolar again displayed pericementitis and class II mobility. A diagnosis of recurrent periapical periodontitis was made, and the tooth was retreated by mechanical and chemical debridment. No antibiotic therapy was included in this treatment. In February 1998, the canal was obturated with gutta percha and AH26 cement (Dentsply DeTrey). Four months later, the second premolar was

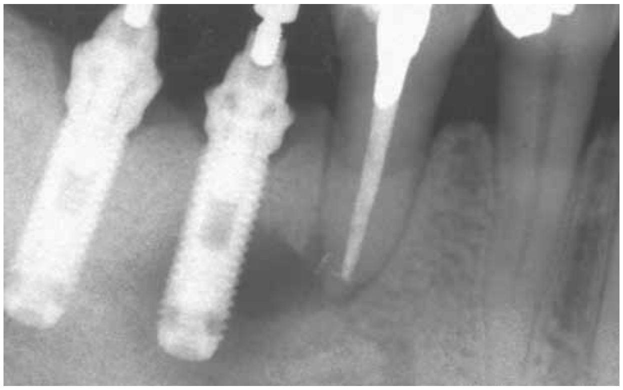

Fig. 5. Periapical radiograph taken February 1998 shows status of periapical radiolucency after endodontic retreatment and placement of provisional prosthesis.

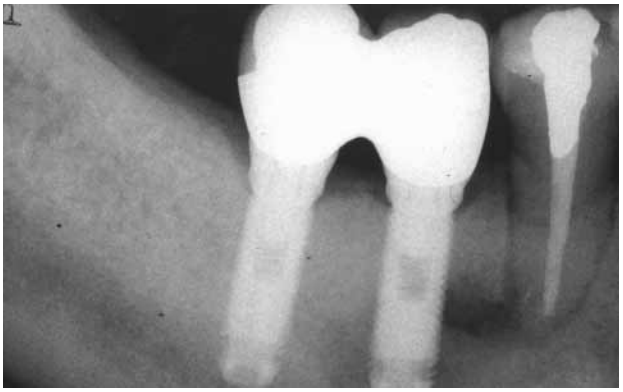

Fig. 6. Periapical radiograph taken June 1999 shows improved status of periapical radiolucency at time of final prosthesis attachment.

asymptomatic, and radiographic evaluation revealed some increase in bone density at the dental implant surface (Fig. 5). An additional 6 months for healing was suggested before implant restoration.

In February 1999, radiographic evaluation demonstrated the persistence of radiolucency. However, by May 1999, the radiolucency associated with the second premolar was obviously reduced, and the bone density at the implant had increased. Restoration of the implants was finally initiated. The final restoration of the implants that replaced the right mandibular first and second molars was delivered in June 1999 (Fig. 6). The status of the peri-implant bone in September 1999 continued to show improvement.

DISCUSSION

This report indicates that complications associated with dental implants that create concern for the integrity of osseointegration (for example, adjacent bacterial infection) require significant time for resolution. The risk of localized osteomyelitis and implant loss as previously reported was considered when the decision to maintain this fixture was made.[5] We considered the implant in this case to be at reduced risk because it was not placed immediately into an extraction socket. Although it may be expected that pericementitis can be resolved directly after endodontic treatment of necrotic teeth, this case report demonstrates that recurrent infection can lead to significant (>2 year) delays in prudent implant treatment.

The impact of endodontic abscesses on dental implant therapy has been considered only a few times in the literature. "Implant periapical lesion" is defined as implant-associated osteolysis occurring after the overheating of bone, implant contamination, or adjacent tooth-associated periapical infection.[6,7] Apical osteolysis at a dental implant has not been shown to invoke adjacent pericementitis or periapical osteolysis. However, Shaffer et al[8] suggest that periradicular infection of teeth could lead to osteolysis at the apex of an adjacent implant. In the case presented here, implant placement was temporally associated with the onset of pericementitis at the adjacent tooth. However, no cause-and-effect relationship could be established.

SUMMARY

The vitality of teeth adjacent to dental implants should be considered in the treatment planning of dental implants. Both the restorability of an endodontically treated tooth and the risk of infection of the adjacent implant are important factors in planning for success. Given the illustrated difficulties and difficulties associated with resolving periapical infections of teeth and implants, it is essential to define the vitality of teeth by careful pulp testing and to consider the integrity of existing questionable, endodontically treated teeth before implant treatment. The risk of periapical infection at teeth adjacent to implants must be minimized.

REFERENCES

1. Lekholm U. Surgical considerations and possible shortcomings of host sites. J Prosthet Dent 1998;79:43-8.
2. Gunne J, Rangert B, Glantz PO, Svensson A. Functional loads on freestanding and connected implants in three-unit mandibular prostheses opposing complete dentures: an in vivo study. Int J Oral Maxillofac Implants 1997;12:335-41.
3. Esposito M, Hirsch JM, Lekholm U, Thomsen P. Biological factors contributing to failures of osseointegrated oral implants. (II). Etiopathogenesis. Eur J Oral Sci 1998;106:721-64.
4. Rubenstein JE, Taylor TD. Apical nerve transection resulting form implant placement: a 10-year follow-up report. J Prosthet Dent 1997;78:537-41.
5. Sussman HI, Moss SS. Localized osteomyelitis secondary to endodontic-implant pathosis. A case report. J Periodontol 1993;64:306-10.
6. Reiser GM, Nevins M. The implant periapical lesion: etiology, prevention, and treatment. Compend Contin Educ Dent 1995;16:768, 770, 772 passim.
7. Piattelli A, Scarano A, Piattelli M. Abscess formation around the apex of

a maxillary root form implant: clinical and microscopical aspects. A case report. J Periodontol 1995;66:899-903.
8. Shaffer MD, Juruaz DA, Haggerty PC. The effect of periradicular endodontic pathosis on the apical region of adjacent implants. Oral Surg Oral Med Oral Pathol Oral Radiol Endod 1998;86:578-81.

Reprint requests to:
Dr Lyndon F. Cooper
Department of Prosthodontics
UNC School of Dentistry
404 Brauer Hall, CB #7450
Chapel Hill, NC 27599-7450
Fax: (919)966-3821
E-mail: lyndon_cooper@dentistry.unc.edu

Copyright © 2001 by The Editorial Council of *The Journal of Prosthetic Dentistry.*
0022-3913/2001/$35.00 + 0. **10/1/113353**

doi:10.1067/mpr.2001.113353

Noteworthy Abstracts of the Current Literature

Dentist-related factors influencing the amount of prosthodontic treatment provided
Kronstrom M, Palmqvist S, Soderfeldt B, Carlsson GE.
Community Dent Oral Epidemiol 2000;28:185-94.

Purpose. The objective of this study was to examine the association between indicators of the amount of prosthodontic treatment and dentist-related factors. A previous study of general dentists in the public dental health service in a Swedish county found that factors related to dentists rather than to patients explained approximately 20% of the variation in prosthodontic service rates.
Material and methods. Questionnaires were sent to a random sample of 2059 general dentists in Sweden with an overall response rate of 76%. Fifty percent of the respondents were in private practice and the rest in the public health service. The gender distribution was 58% male with no significant differences between respondents and nonrespondents related to gender or dental care system. Dependent variables were: (1) reported weekly working hours used for prosthodontics, and (2) reported number of prosthodontic procedures. Independent variables were related to "social and demographic attributes," "job situation," and "attitudes of dentists." Multiple regression analysis was used where a continuous dependent variable could be obtained. When the dependent variable was categorical, dichotomies were constructed for use as dependent variables in logistic regression models.
Results. The article includes charts that illustrate the multiple regression models for independent variables. "Weekly working hours used for dental care of adults" showed a strong association in all models with the dependent variable "weekly working hours used for prosthodontics." Male dentists provided more prosthodontic services than female dentists, even if the former reported less time used for prosthodontics. Private practitioners produced more fixed prosthodontics than dentists employed in the public health service. Dentists in the public health service reported a higher production of removable dentures than did private practitioners.
Conclusions. Factors other than patient status and scientific knowledge seem to have an impact on the amount of services provided. 21 References. —*ME Razzoog*
