# Supplementary material for: A novel histopathological classification of implant periapical lesion: A systematic review and treatment decision tree
Source: PLoS One. 2022 Dec 22;17(12):e0277387. doi: 10.1371/journal.pone.0277387 (PMC9778521; doi:10.1371/journal.pone.0277387)
Supplement: S1 File — (ZIP) [file pone.0277387.s001.zip › support files/Included study/Chan 2011.pdf]

Case Report

Retrograde Peri-Implantitis: A Case Report Introducing an Approach to Its Management

Hsun-Liang Chan,* Hom-Lay Wang,* Jill D. Bashutski,* Paul C. Edwards,† Jia-Hui Fu,* and Tae-Ju Oh*

Background: *Although several potential etiologic factors associated with retrograde peri-implantitis (RPI) and potential treatment options have been discussed in the literature, the etiology has not been fully investigated and the definitive management methods remain undefined. We propose a decision-making protocol for the treatment of RPI and provide new insight into the etiology of this process based on the findings from two clinical cases.*

Methods: *The medical and dental histories of two patients who developed RPI were thoroughly reviewed. Both patients were treated according to the treatment guidelines proposed in this manuscript. Fluid from the lesions was collected to examine the presence of 11 bacterial species by molecular-based microbial testing. Biopsies were also obtained for histopathologic examination.*

Results: *Patient 1, previously diagnosed with human immunodeficiency virus infection, developed RPI 3 months after implant placement. Histopathologic examination revealed a predominantly fibrous connective tissue response with minimal inflammatory infiltrate and bone formation. Patient 2 presented histopathologically with an intense acute inflammatory response.* Eikenella corrodens *was detected by microbial testing. Three months after surgical intervention, both cases healed uneventfully, and the radiodensity in the lesions significantly increased. The two implants are now functional and free of further complications.*

Conclusions: *The possible role of bacterial infection from an adjacent tooth may be a potential etiologic factor in the development of RPI. In addition, HIV infection may be associated with RPI and deserves further investigation. A decision-making flowchart was proposed after critically evaluating the currently available relevant literature. Both cases presented in this manuscript were successfully treated by following this protocol.* J Periodontol 2011;82:1080-1088.

KEY WORDS

Bacterial infections; dental implants; HIV infections; pathology, oral; periapical periodontitis; peri-implantitis.

* Graduate Periodontics, Department of Periodontics and Oral Medicine, School of Dentistry, University of Michigan, Ann Arbor, MI.
† Oral and Maxillofacial Pathology, Department of Periodontics and Oral Medicine, School of Dentistry, University of Michigan.

doi: 10.1902/jop.2010.100585

Retrograde peri-implantitis (RPI) is defined as a clinically symptomatic periapical lesion (diagnosed as a radiolucency) that develops shortly after implant insertion in which the coronal portion of the implant achieves a normal bone–implant interface.[1] This condition was first described by McAllister et al.[2] in which microbial involvement from the implant site, the extracted teeth or adjacent teeth, generation of excess bone heat during implant placement, and premature loading from inadequate relief of interim prostheses were considered the probable causes. Other etiologic possibilities are summarized in Table 1.[2-10]

The prevalence of RPI was assessed in a retrospective study of 539 implants, with 1.6% of maxillary and 2.7% of mandibular teeth exhibiting this condition before abutment connection.[4] Endodontic periapical pathology associated with either the previously extracted or neighboring teeth was present more often (three times) in cases of peri-implantitis compared to successfully integrated implants, suggesting that endodontic pathology is the most likely primary etiology. A recent article by Zhou et al.[11] supports this theory because their reported incidence of peri-implantitis on implants adjacent to an endodontically treated tooth was 7.8%, which is higher than the overall reported incidence.

Sussman[12] proposed two pathways that may lead to RPI: type 1 (implant to tooth) and type 2 (tooth to implant). Type 1 RPI occurs when the osteotomy preparation causes direct or indirect damage to the adjacent tooth, resulting in devitalization of the tooth pulp and periapical pathology. Subsequently, the periapically infected tooth inhibits osseointegration of the implant. Type 2 RPI occurs when an adjacent tooth with periapical pathology contaminates the fixture and interferes with osseointegration of the implant.

Reiser and Nevins[3] proposed an alternate RPI classification system, based on the presence or absence of symptoms, as either inactive or infected lesions. The inactive lesion may mimic a periapical scar at the root apex and not present with clinical symptoms, whereas the infected lesion is usually associated with pain, tenderness, fistula formation, or swelling.

Although there have been numerous reports that have discussed the etiology and treatment of RPI (Table 1), clinical decision-making guidelines are still lacking. The aim of this article is to propose a decision-making flowchart for the treatment of RPI from critically evaluating different surgical modalities based on evidence-based review. The histology, possible etiology, and treatment of two RPI cases are also presented.

病因汇总

Table 1.

Review of Etiology of Implants Affected by RPI

Probable etiology
Residual bacteria in implant site[2,8-10]
Adjacent endodontic lesion[4,5,7,8]
Violation of minimal distance from adjacent tooth[8]
Overheating[2,3,8,10]
Implant surface contamination[6,10]
Residual root particles or foreign bodies[3,10]
Surgical drilling beyond the length of the implant[3]
Fenestration of vestibular bone[10]
Bone compression[10]
Drainage of inflammation via marrow spaces[8]
Poor bone quality[10]
Premature loading[2]
Development of osteomyelitis[8]
Technique of the particular implant system used[8]
Bone loss caused by mucoperiosteal flap procedure[8]

MATERIALS AND METHODS

Case 1

A 45-year-old male was referred from the Department of Advanced Education in General Dentistry, School of Dentistry, University of Michigan, for implant consultation regarding tooth #25 in May 2009. Oral informed consent to be profiled was obtained from the patient prior to the commencement of treatment. Medical history was significant for human immunodeficiency virus (HIV) infection (CD4 count and viral load were $548/\mu L$ [normal CD4 count ranged from 500 to $1,000/\mu L$] and <48 copies/mL, respectively). In addition, white blood cell and red blood cell counts were below the normal range ($3.9 \times 10^3/mm^3$ and 3.49×10^6 cells/μL, respectively). Antiviral medications included the combinations of lopinavir and ritonavir[‡] and lamivudine and zidovudine.[§] Tooth #25 had been treated endodontically several years ago and had fractured 1 month prior. The tooth was asymptomatic. A periapical radiograph showed incomplete root canal treatment with apical radiolucency (Fig. 1I). Because the tooth was non-restorable,

‡ Kaletra, Abbott Laboratories, Abbott Park, IL.
§ Combivir, ViiV Healthcare, Research Triangle Park, NC.

ridge preservation with allograft[‖] covered with a collagen plug[¶] at the time of tooth extraction with delayed single implant replacement was performed (Figs. 1A through 1C and Fig. 1J). At the postoperative appointment, dislodgment of the collagen plug was noted, with some allograft exfoliation on the surface of the socket. No signs of infection were evident. Four months after the ridge-preservation procedures, a 3.25 × 13-mm implant[#] was placed in a one-stage approach. The postoperative course of healing was uneventful. Three months after implant placement, a radiolucency measuring 4 mm in diameter was identified at the implant apex. A diagnosis of RPI (Fig. 1K) was established. In subsequent follow-up visits, the lesion demonstrated an increase in size, and consequently surgical intervention was planned.

An incision was made at the mucogingival junction and a periosteal flap was raised. The buccal plate was removed to gain access to the lesion (Figs. 1D through 1F). Apical fluid was collected with paper points and processed using a commercially available DNA test kit[**] to determine the presence of 11 pathogenic microbial species. The tested bacteria included *Aggregatibacter actinomycetemcomitans* (previously *Actinobacillus actinomycetemcomitans*), *Porphyromonas gingivalis*, *Tannerella forsythia* (previously *T. forsythensis*), *Treponema denticola*, *Prevotella intermedia*, *Parvimonas micra* (previously *Peptostreptococcus micros*), *Fusobacterium nucleatum*, *Campylobacter rectus*, *Cubacterium nodatum*, *Eikenella corrodens*, and *Capnocytophaga* species. In addition, tissue was collected from the lesion for a histopathologic examination. The bony defect around the implant tip was irrigated with 0.12% chlorhexidine gluconate[††] and subsequently filled with allograft[‡‡] mixed with 250 mg tetracycline powder and 0.9% normal saline (Fig. 1G). The flap was approximated and sutured with 5-0 chromic gut.[§§] The healing was uneventful and the implant was restored 3 months after the surgery (Fig. 1H). At a 6-month postoperative appointment, significant radiographic resolution of the lesion was observed (Fig. 1L).

Case 2

A 54-year-old female presented to the University of Michigan School of Dentistry on October 8, 2009, for implant placement in the tooth #12 area. The patient gave verbal consent to be profiled prior to treatment. Her medical history included seasonal allergies and rosacea. At the time of presentation, she was taking over-the-counter ibuprofen, acetaminophen, and a nasal decongestant spray when necessary. The patient reported that tooth #12 had been extracted because of dental caries and had been missing for >3 years. On March 12, 2010, a dental implant[‖‖] (4 × 11 mm) was placed, uneventfully, in a one-stage approach. At 1-week postoperatively, moderate swelling was noted in the apical area of teeth #12 and #13. Percussion test was positive on tooth #13. However, a periapical radiograph of teeth #12 and #13 showed no abnormalities. The patient was placed on amoxicillin, 500 mg three times daily, for 7 days and referred for endodontic evaluation on tooth #13. Subsequently, endodontic therapy on tooth #13 was completed on April 16, 2010. A radiograph of the area revealed a peri-implant radiolucency localized to the apex of tooth #12, and surgical intervention to correct the lesion was initiated (Fig. 2). On clinical examination of the implant, no mobility was noted. An incision was made at the mucogingival junction and a full-thickness flap was reflected. There was no buccal plate found covering the RPI lesion. Fluid and paper point samples were obtained from the apical lesion and sent for microbial testing as described previously. In addition, soft tissue from the lesion was removed for histopathologic examination. The apical bony defect was degranulated and cortical bone allograft[¶¶] with a bioabsorbable membrane[##] was placed over the defect. The flap was repositioned and sutured with 4-0 chromic gut sutures.[***] At the 2-week postoperative appointment, it was noted that healing was within normal limits. Three months after the surgery, the implant was restored (Fig. 2F), and at 6 months periapical radiographs demonstrated resolution of the lesion (Fig. 2H).

case1 结缔组织周围炎症细胞

Microbial Test Results

The microbial test of Case 1 failed to detect any examined bacterial species. Histopathologically (Fig. 3A), the specimen consisted of fibrous connective tissue with a mild chronic mixed inflammatory cell infiltrate. Viable bone was noted at the periphery of the specimen. At higher magnification (Fig. 3B), clusters of inflammatory cells, primarily lymphocytes, were noted in a background of relatively dense connective tissue.

In Case 2, the bacterial test revealed increased numbers of *E. corrodens*. Histopathologically, numerous acute and chronic inflammatory cells could be seen in a background of immature granulation tissue containing numerous small- to medium-diameter blood vessels (Fig. 3C). At higher magnification (Fig.

细菌可见大肠杆菌，组织病理学急慢性炎症细胞

‖ Puros Cortical Particulate Allograft, Zimmer Dental, Carlsbad, CA.
¶ CollaPlug, Zimmer Dental.
\# 3i NanoTite Tapered, Biomet 3i, Warshaw, IN.
** Micro-IDent plus, Hain Diagnostics, Midland, TX.
†† Peridex, 3M, St. Paul, MN.
‡‡ Puros Cortical Particulate Allograft, Zimmer Dental.
§§ Ethicon, Johnson & Johnson, Somerville, NJ.
‖‖ AstraTech Osseospeed Straight, Molndal, Sweden.
¶¶ Puros Cortical Particulate Allograft, Zimmer Dental.
\## Biomend, Zimmer Dental.
*** Ethicon, Johnson & Johnson.

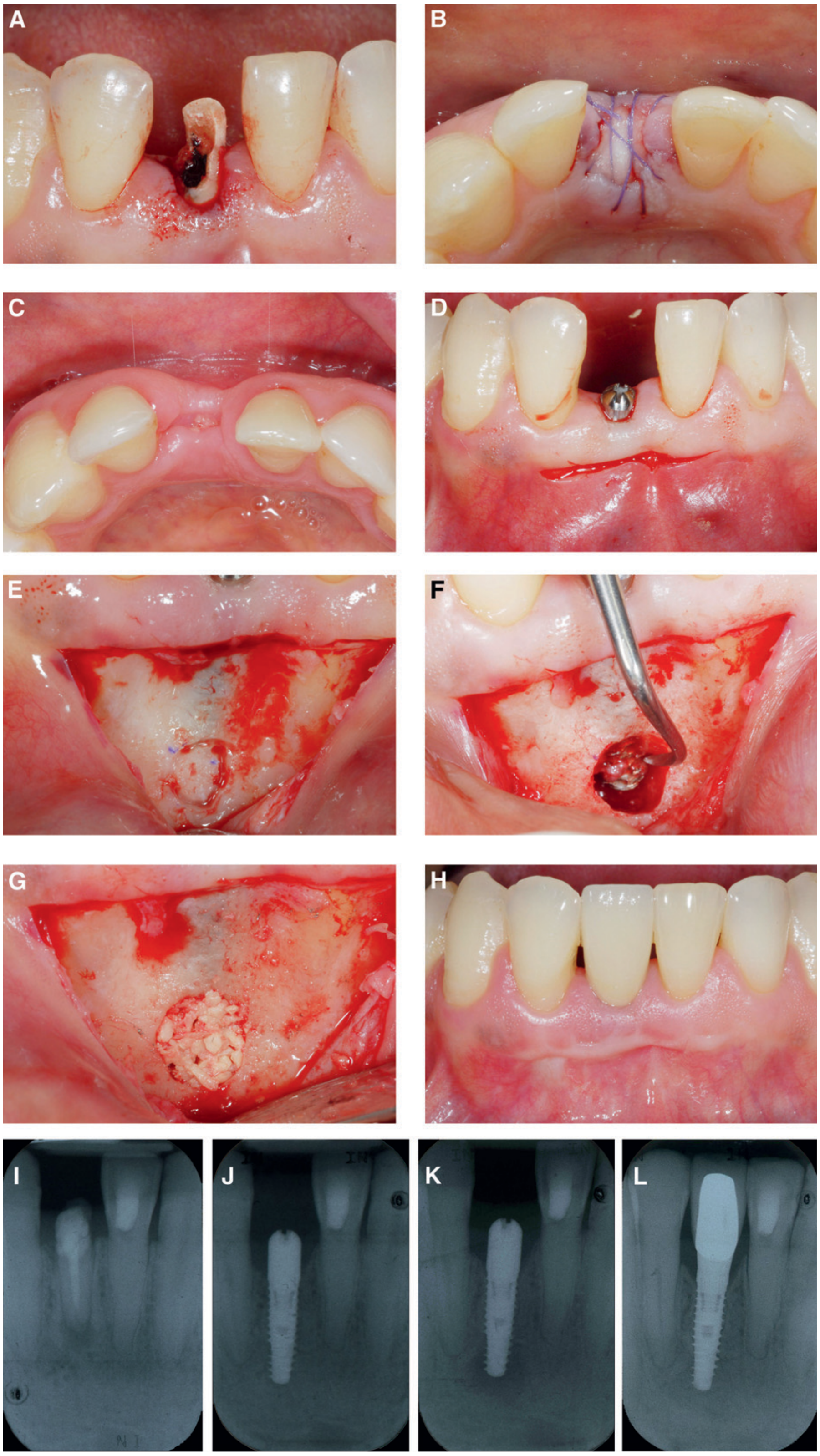

Figure 1.
*Case 1. Unrestorable tooth #25 with apical radiolucency. Extraction, socket preservation, and delayed implant placement were performed **(A through C, I,** and **J)**. Apical lesion at the implant was revealed **(K)**, and surgical debridement and bone grafting were performed **(D through G)**. There was uneventful healing **(H)**, and the x-ray showed increased density **(L)**.*

3D), collections of neutrophils, macrophages, and lymphocytes were noted within the loose vascular connective tissue.

DISCUSSION

hiv影响骨愈合

Possible Role of HIV Infection in RPI

The delayed or non-union of bone fractures in HIV patients, caused by impaired wound healing, is well recognized[13] and several mechanisms have been proposed.[14] The virus is known to alter the rate of bone resorption and formation.[14] It is speculated that the mechanism involves the modulation of the tumor necrosis factor-α superfamily, including the receptor activator nuclear factor-kappa B ligand and osteoprotegerin, by HIV.[15] Osteocalcin is reduced in the serum of patients with HIV although the precise function of osteocalcin was not fully elucidated.[16] The deregulation of various systemic cytokines that occurs in patients who are HIV-positive may disrupt bone homeostasis.[17] HIV infection may also compromise microcirculation and thus affect bone healing.[18]

There is currently a lack of evidence regarding the incidence of implant complications in patients who are HIV-positive compared to healthy patients.[19] Based on the fact that bone healing might be impaired by HIV infection, through the aforementioned mechanisms, dental implant healing might also be affected in HIV-active or AIDS patients. To the best of our knowledge, this is the first article to report the possible association between HIV infection and RPI. In this case, when the extraction socket was thoroughly debrided and socket augmentation procedure was performed, little bleeding was noted during the procedure, suggesting impaired microcirculation.

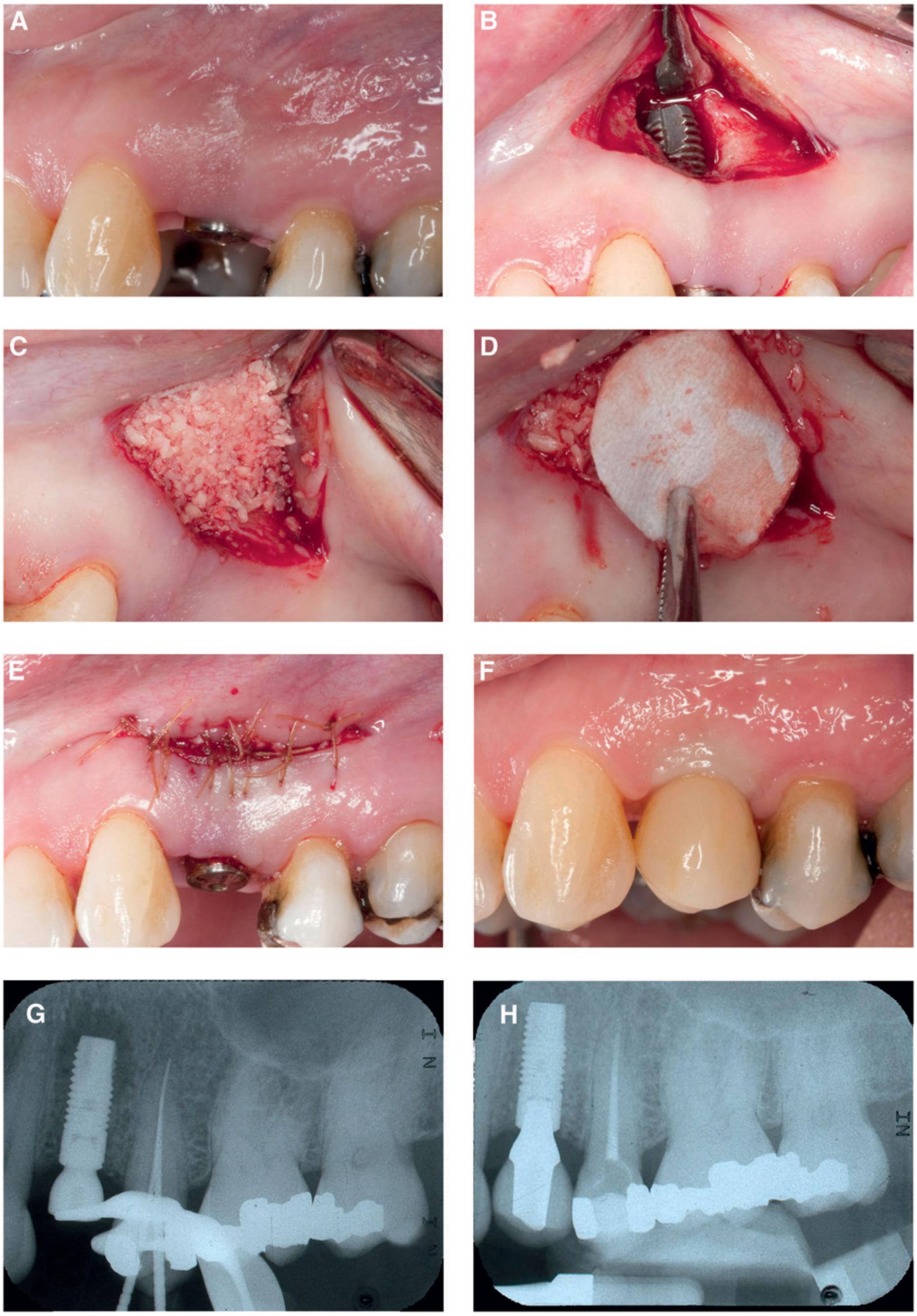

Figure 2.
Case 2. ***A)*** *Persistent swelling associated with a recently placed #12 implant.* ***B through E)*** *Surgical intervention was executed with a guided bone regeneration technique;* ***F)*** *the healing was uneventful. Radiograph revealed a well-defined radiolucent lesion at the implant apex corresponding with the swelling.* ***G)*** *Tooth #13 during endodontic therapy.* ***H)*** *Radiograph showed normal bone density after the crown was delivered.*

At the postoperative follow-up appointment, the socket was exposed without full soft-tissue coverage and the coronal part of bone graft had dislodged, indicative of unfavorable healing. Histology of the RPI lesion showed significant soft-tissue encroachment, suggestive of impaired bone formation at the implant apex. As a result, the possible role of HIV infection on the development of RPI cannot be ruled out.

Possible Association Between Endodontic Lesions and RPI

Most case reports (Table 1) suggest that residual bacteria in a radiographically healed socket or in an adjacent periapical lesion are the main cause of RPI. It was further suggested that endodontic bacteria can be reactivated during implant osteotomy, leading to implant infection.[2,10] In both of these cases, microbial examination was used to detect the presence of 11 pathogenic bacterial species in the apical lesion of the affected implants. To the best of our knowledge, this was the first time that a culture-independent microbial examination was applied for the detection of RPI-associated bacteria. This kit was primarily designed for the detection of periodontal pathogens. We used this technique to screen for the presence of microbial pathogens associated with the apical lesions in this study because some of these 11 species are also associated with periapical pathosis.[20-22] The number of *E. corrodens* was found to be elevated in Case 2, suggesting a possible association with the development of RPI. The fact that this specific species is commonly found in endodontic lesions suggests that the endodontic lesion from adjacent teeth may be a factor in the development of RPI. The evidence would have been more convincing if this species was also detected at the adjacent infected root apex.

Treatment of RPI

Table 2 provides a summary of the available literature on the management of RPI.[2,5,7,9,23-28] Various techniques were used, such as debridement only or the combination of debridement with grafting

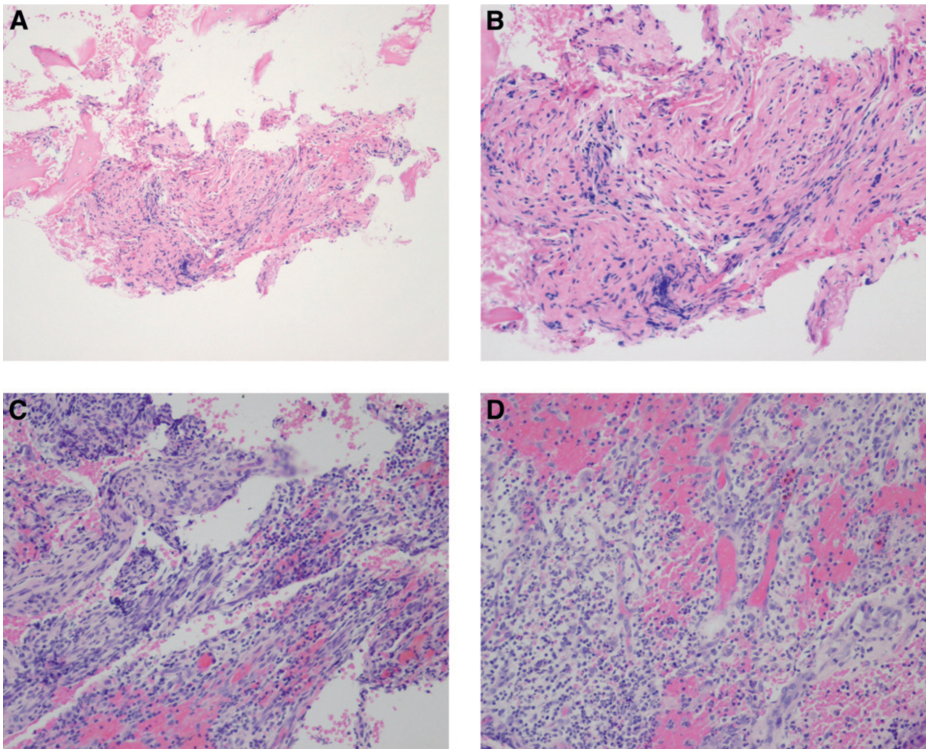

Figure 3.
Histopathology (hematoxylin and eosin stain) of the two RPI cases. Case 1: ***A)*** *At lower magnification (×10), fibrous connective tissue with a mild, chronic, mixed inflammatory cell infiltrate was noticed.* ***B)*** *At higher magnification (×20), clusters of lymphocytes were in a background of relatively dense connective tissue. Case 2:* ***C)*** *Lower magnification (×20) showed numerous acute and chronic inflammatory cells with immature granulation tissue.* ***D)*** *At higher magnification (×60), collections of neutrophils, macrophages, lymphocytes, and engorged blood vessels were noted.*

material with or without membrane, detoxification of infected implant surfaces, or apicoectomy. The treatment goals of RPI include the elimination of infection, resolution of the lesion, and ultimately implant survival. Our proposed decision-making flowchart (Fig. 4) for the treatment of RPI requires identification of the most likely etiology. Vitality testing and radiographic examination on neighboring teeth are required because in most case reports, the lesion has been linked to apical pathology from adjacent teeth. Simultaneously, the stability of the affected implant needs to be assessed. If the infected implant is mobile, it should be removed and the socket thoroughly debrided.[29] The socket can be either grafted for later implant placement[30,31] or it can receive a wider or longer implant after the removal of the mobile implant.[32] Generally, a staged approach is preferred unless elimination of the etiologic causative factors is certain. Systemic antibiotics alone are not able to abate the signs and symptoms.[23,27] Alternatively, a surgical approach combined with systemic antibiotics may resolve the lesion provided the implant is not mobile.[2,5,9,23-27]

治疗的目的

各种治疗方法的评价

BRG应用于根尖缺损更好

The literature supports a surgical approach that may include only debridement[4,9] or bone replacement grafts (BRGs) with or without the use of membrane barriers.[2,4,5,27] The use of different types of BRGs with or without occlusive membranes is not universally accepted; however, their application may provide several advantages. First, BRGs can act as a scaffold for new bone cells to grow into the bony defect. Second, they can maintain the space and prevent soft tissue from repopulating the defect. It has been demonstrated that the use of bone regeneration materials for apicoectomy surgeries improves the predictability of clinical, radiographic, and histologic healing.[33] Studies also suggest that the initial defect size adversely influences tissue healing after apicoectomy procedures, with defects ≤5 mm showing better healing.[34,35]

可借鉴

Four types of BRGs are available. The autogenous bone graft is considered the gold standard because it possesses osteogenic properties. Nevertheless, their limited availability and the potential for donor site morbidity reduce their usefulness. Allografts, such as freeze-dried bone graft or demineralized freeze-dried bone graft, overcome some of the potential limitations of autogenous bone grafting. Periodontal regeneration induced by demineralized freeze-dried bone graft, including the formation of new bone, cementum, and periodontal ligament fibers, has been documented in a human histology study.[36] Freeze-dried bone allograft has been successfully used in apical lesions associated with failed endodontic treatment.[37] Xenografts, most commonly from bovine bone, provide similar properties to allograft materials. Alloplasts, acting as fillers, result in tissue repair rather than regeneration.[38] To achieve more predictable bone regeneration, BRGs, except alloplasts, can be used for treating RPI.[38]

The concept of guided tissue regeneration based on Melcher's conceptual article[39] uses barrier membranes to differentiate cell growth. When applied in periapical surgeries, it has been demonstrated to yield good results in terms of shortening the healing

Table 2.

Review of the Treatment Approaches for Implants With RPI

Reference	Cases (n)	Grafting Material	Membrane	Detoxification	Apicoectomy
McAllister et al.[2]	2	Allograft<br>Allograft	No<br>Copolymer	Tetracycline<br>Tetracycline	No<br>No
Bretz et al.[28]	1	Allograft	No	Chlorhexidine	No
Shaffer et al.[5]	2	Allograft<br>No	Copolymer<br>No	No<br>No	Yes<br>Yes
Ayangco and Sheridan[9]	3	No<br>No<br>No	No<br>No<br>No	Tetracycline<br>Tetracycline<br>Tetracycline	No<br>No<br>No
Brisman et al.[7]	1	No	No	No	No
Jalbout and Tarnow[27]	4	Xenograft<br>Xenograft<br>Allograft<br>Alloplast	Collagen<br>Copolymer<br>No<br>No	No<br>No<br>No<br>No	Yes<br>No<br>No<br>No
Flanagan[26]	1	Calcium hydroxide paste	No	No	No
Ataullah et al.[25]	1	Xenograft	Collagen	Chlorhexidine	No
Tozum et al.[24]	1	Calcium sulfate	Collagen	No	No
Dahlin et al.[23]	2	No<br>No	No<br>No	No<br>No	Yes<br>Yes

time and increasing the amount of bone fill.[40,41] Because healing is less favorable in large periapical defects,[34,35] the application of occlusive membrane might be beneficial in these situations.

Reosseointegration requires implant surface detoxification and the application of chemotherapeutic agents, such as tetracycline, chlorhexidine gluconate, and citric acid.[29] One animal study investigated four methods: 1) air-powder abrasive unit and citric acid, 2) air-powder abrasive unit only, 3) gauze soaked in saline and citric acid, and 4) gauze soaked alternately in chlorhexidine and saline for surface treatment of peri-implantitis.[42] The degree of reosseointegration varies greatly,[43] and does not seem to differ among these methods. Because no one method has proved to be distinctly superior, chemical agents or air abrasives may be used.[44]

Implant apicoectomy has been thoroughly covered in many case reports.[5,23,27] Although Ayangco and Sheridan[9] suggested that scratching of the implant surface in such a location might not be crucial, Ataullah et al.[25] suggested that surface debridement be carried out with extreme care to prevent damage to the surface of the fixture. Practically, if the affected implant prevents thorough and complete debridement, it can be sectioned.[30]

CONCLUSIONS

A decision-making flowchart for the treatment of RPI was proposed based on review of currently available relevant literature. Bacterial contamination from endodontic lesions as an important etiologic factor was further strengthened by the presence of *E. corrodens* revealed by a DNA-based microbial test in this article. Further research should concentrate on the role of compromised systemic health on RPI and the investigation of the effectiveness of different surgical modalities for the treatment of RPI.

ACKNOWLEDGMENTS

The authors thank Dr. Kevin Cook, resident, Advanced Education in General Dentistry Program, University of Michigan School of Dentistry, for referring Case 1 and providing the restoration, and Dr. Ivan Rudek, research fellow, Graduate Periodontics, University of Michigan School of Dentistry, for restoring the implant crown for Case 2. This paper was partially supported by the University of Michigan Periodontal Graduate Student Research Fund. The authors report no conflicts of interest related to this case report.

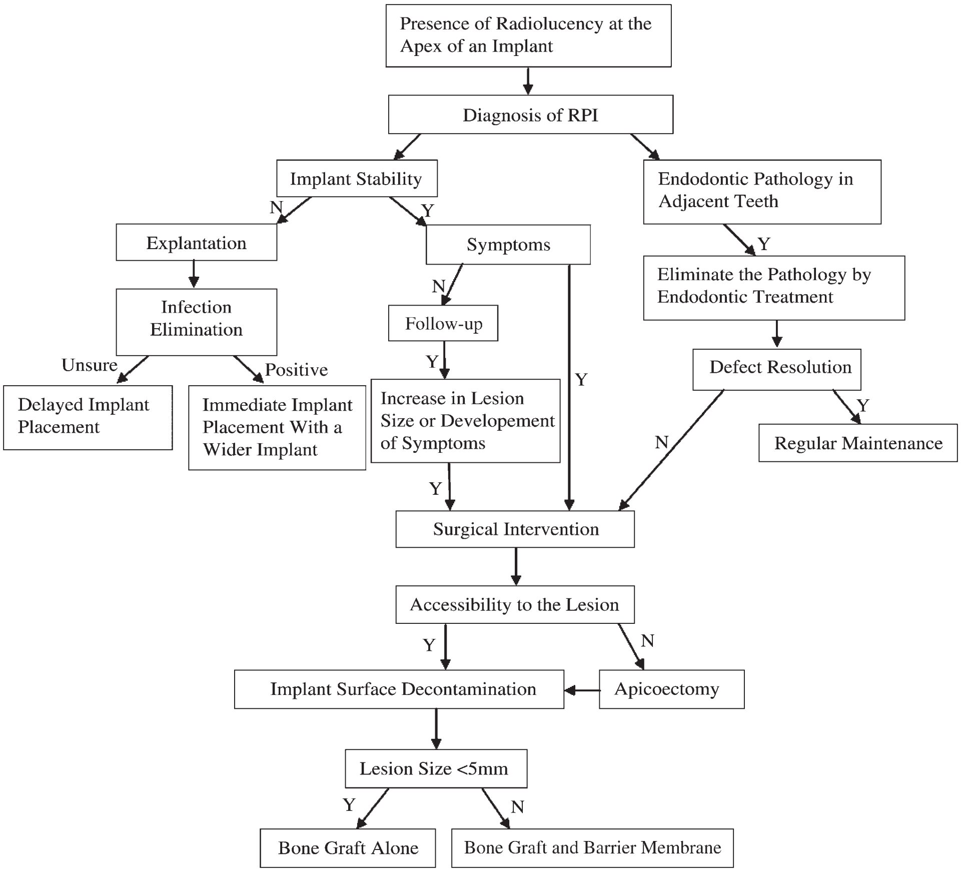

Figure 4.
Decision-making flowchart for the management of RPI. Y = yes; n = no.

REFERENCES

1. Quirynen M, Gijbels F, Jacobs R. An infected jawbone site compromising successful osseointegration. *Periodontol 2000* 2003;33:129-144.
2. McAllister BS, Masters D, Meffert RM. Treatment of implants demonstrating periapical radiolucencies. *Pract Periodontics Aesthet Dent* 1992;4:37-41.
3. Reiser GM, Nevins M. The implant periapical lesion: Etiology, prevention, and treatment. *Compend Contin Educ Dent* 1995;16:768, 770, 772 passim.
4. Quirynen M, Vogels R, Alsaadi G, Naert I, Jacobs R, van Steenberghe D. Predisposing conditions for retrograde peri-implantitis, and treatment suggestions. *Clin Oral Implants Res* 2005;16:599-608.
5. Shaffer MD, Juruaz DA, Haggerty PC. The effect of periradicular endodontic pathosis on the apical region of adjacent implants. *Oral Surg Oral Med Oral Pathol Oral Radiol Endod* 1998;86:578-581.
6. Scarano A, Di Domizio P, Petrone G, Iezzi G, Piattelli A. Implant periapical lesion: A clinical and histologic case report. *J Oral Implantol* 2000;26:109-113.
7. Brisman DL, Brisman AS, Moses MS. Implant failures associated with asymptomatic endodontically treated teeth. *J Am Dent Assoc* 2001;132:191-195.
8. Sussman HI, Moss SS. Localized osteomyelitis secondary to endodontic-implant pathosis. A case report. *J Periodontol* 1993;64:306-310.
9. Ayangco L, Sheridan PJ. Development and treatment of retrograde peri-implantitis involving a site with a history of failed endodontic and apicoectomy procedures: A series of reports. *Int J Oral Maxillofac Implants* 2001;16:412-417.
10. Piattelli A, Scarano A, Balleri P, Favero GA. Clinical and histologic evaluation of an active "implant periapical lesion": A case report. *Int J Oral Maxillofac Implants* 1998;13:713-716.
11. Zhou W, Han C, Li D, Li Y, Song Y, Zhao Y. Endodontic treatment of teeth induces retrograde peri-implantitis. *Clin Oral Implants Res* 2009;20:1326-1332.
12. Sussman HI. Periapical implant pathology. *J Oral Implantol* 1998;24:133-138.
13. Richardson J, Hill AM, Johnston CJ, et al. Fracture healing in HIV-positive populations. *J Bone Joint Surg Br* 2008;90:988-994.
14. Amiel C, Ostertag A, Slama L, et al. BMD is reduced in HIV-infected men irrespective of treatment. *J Bone Miner Res* 2004;19:402-409.
15. Ueland T, Bollerslev J, Godang K, Müller F, Frøland SS, Aukrust P. Increased serum osteoprotegerin in disorders characterized by persistent immune activation or glucocorticoid excess — Possible role in bone homeostasis. *Eur J Endocrinol* 2001;145:685-690.
16. Aukrust P, Haug CJ, Ueland T, et al. Decreased bone formative and enhanced resorptive markers in human immunodeficiency virus infection: Indication of

normalization of the bone-remodeling process during highly active antiretroviral therapy. *J Clin Endocrinol Metab* 1999;84:145-150.
17. Sachdeva N, Yoon HS, Oshima K, Garcia D, Goodkin K, Asthana D. Biochip array-based analysis of plasma cytokines in HIV patients with immunological and virological discordance. *Scand J Immunol* 2007;65:549-554.
18. Erbe M, Rickerts V, Bauersachs RM, Lindhoff-Last E. Acquired protein C and protein S deficiency in HIV-infected patients. *Clin Appl Thromb Hemost* 2003;9: 325-331.
19. Patton LL, Shugars DA, Bonito AJ. A systematic review of complication risks for HIV-positive patients undergoing invasive dental procedures. *J Am Dent Assoc* 2002;133:195-203.
20. Rupf S, Kannengiesser S, Merte K, Pfister W, Sigusch B, Eschrich K. Comparison of profiles of key periodontal pathogens in periodontium and endodontium. *Endod Dent Traumatol* 2000;16:269-275.
21. Siqueira JF Jr., Rôças IN. The microbiota of acute apical abscesses. *J Dent Res* 2009;88:61-65.
22. Siqueira JF Jr., Rôças IN, Souto R, Uzeda M, Colombo AP. Microbiological evaluation of acute periradicular abscesses by DNA-DNA hybridization. *Oral Surg Oral Med Oral Pathol Oral Radiol Endod* 2001;92:451-457.
23. Dahlin C, Nikfarid H, Alsén B, Kashani H. Apical peri-implantitis: Possible predisposing factors, case reports, and surgical treatment suggestions. *Clin Implant Dent Relat Res* 2009;11:222-227.
24. Tözüm TF, Sençimen M, Ortakoğlu K, Ozdemir A, Aydin OC, Keleş M. Diagnosis and treatment of a large periapical implant lesion associated with adjacent natural tooth: A case report. *Oral Surg Oral Med Oral Pathol Oral Radiol Endod* 2006;101:e132-e138.
25. Ataullah K, Chee LF, Peng LL, Lung HH. Management of retrograde peri-implantitis: A clinical case report. *J Oral Implantol* 2006;32:308-312.
26. Flanagan D. Apical (retrograde) peri-implantitis: A case report of an active lesion. *J Oral Implantol* 2002; 28:92-96.
27. Jalbout ZN, Tarnow DP. The implant periapical lesion: Four case reports and review of the literature. *Pract Proced Aesthet Dent* 2001;13:107-112, quiz 114.
28. Bretz WA, Matuck AN, de Oliveira G, Moretti AJ, Bretz WA. Treatment of retrograde peri-implantitis: Clinical report. *Implant Dent* 1997;6:287-290.
29. Meffert RM. How to treat ailing and failing implants. *Implant Dent* 1992;1:25-33.
30. Oh TJ, Yoon J, Wang HL. Management of the implant periapical lesion: A case report. *Implant Dent* 2003; 12:41-46.
31. Tseng CC, Chen YH, Pang IC, Weber HP. Peri-implant pathology caused by periapical lesion of an adjacent natural tooth: A case report. *Int J Oral Maxillofac Implants* 2005;20:632-635.
32. Park SH, Sorensen WP, Wang HL. Management and prevention of retrograde peri-implant infection from retained root tips: Two case reports. *Int J Periodontics Restorative Dent* 2004;24:422-433.
33. Tobón SI, Arismendi JA, Marín ML, Mesa AL, Valencia JA. Comparison between a conventional technique and two bone regeneration techniques in periradicular surgery. *Int Endod J* 2002;35:635-641.
34. Hirsch JM, Ahlström U, Henrikson PA, Heyden G, Peterson LE. Periapical surgery. *Int J Oral Surg* 1979;8:173-185.
35. Rud J, Andreasen JO, Jensen JF. A multivariate analysis of the influence of various factors upon healing after endodontic surgery. *Int J Oral Surg* 1972;1:258-271.
36. Bowers GM, Chadroff B, Carnevale R, et al. Histologic evaluation of new attachment apparatus formation in humans. Part III. *J Periodontol* 1989;60:683-693.
37. Saad AY, Abdellatief EM. Healing assessment of osseous defects of periapical lesions associated with failed endodontically treated teeth with use of freeze-dried bone allograft. *Oral Surg Oral Med Oral Pathol* 1991;71:612-617.
38. Wang HL, Greenwell H, Fiorellini J, et al.; Research, Science and Therapy Committee. Periodontal regeneration. *J Periodontol* 2005;76:1601-1622.
39. Melcher AH. On the repair potential of periodontal tissues. *J Periodontol* 1976;47:256-260.
40. Dietrich T, Zunker P, Dietrich D, Bernimoulin JP. Periapical and periodontal healing after osseous grafting and guided tissue regeneration treatment of apicomarginal defects in periradicular surgery: Results after 12 months. *Oral Surg Oral Med Oral Pathol Oral Radiol Endod* 2003;95:474-482.
41. Pecora G, Kim S, Celletti R, Davarpanah M. The guided tissue regeneration principle in endodontic surgery: One-year postoperative results of large periapical lesions. *Int Endod J* 1995;28:41-46.
42. Schou S, Holmstrup P, Jørgensen T, et al. Implant surface preparation in the surgical treatment of experimental peri-implantitis with autogenous bone graft and ePTFE membrane in cynomolgus monkeys. *Clin Oral Implants Res* 2003;14:412-422.
43. Renvert S, Polyzois I, Maguire R. Re-osseointegration on previously contaminated surfaces: A systematic review. *Clin Oral Implants Res* 2009;20(Suppl. 4): 216-227.
44. Claffey N, Clarke E, Polyzois I, Renvert S. Surgical treatment of peri-implantitis. *J Clin Periodontol* 2008; 35(Suppl. 8):316-332.

Correspondence: Dr. Tae-Ju Oh, Department of Periodontics and Oral Medicine, School of Dentistry, University of Michigan, 1011 N. University Ave., Ann Arbor, MI 48109–1078. Fax: 734/763-5503; e-mail: taejuoh@umich.edu.

Submitted September 27, 2010; accepted for publication November 15, 2010.
