# Supplementary material for: A novel histopathological classification of implant periapical lesion: A systematic review and treatment decision tree
Source: PLoS One. 2022 Dec 22;17(12):e0277387. doi: 10.1371/journal.pone.0277387 (PMC9778521; doi:10.1371/journal.pone.0277387)
Supplement: S1 File — (ZIP) [file pone.0277387.s001.zip › support files/Included study/Dahlin 2008.pdf]

Apical Peri-Implantitis: Possible Predisposing Factors, Case Reports, and Surgical Treatment Suggestions

Christer Dahlin, DDS, PhD;*†‡ Hossein Nikfarid, DDS;* Bengt Alsén, DDS;* Hossein Kashani, DDS, PhD*

ABSTRACT

Background: Apical peri-implantitis is often diagnosed by clinical findings such as pain, redness, tenderness, swelling, and sometimes the presence of a fistulous tract. There are few theories about how such a lesion occurs. Hence, the current clinical treatment protocols are scanty.

Purpose: The aim of this report was to evaluate and confer a more extended surgical protocol and to discuss possible predisposing factors for the development of retrograde peri-implantitis.

Materials and Methods: Two patients were extensively evaluated with regard to clinical signs, implant treatment, postoperative complications, and surgical treatment. The surgical protocol comprised debridement, with the additional removal of the apical portion of the affected implant. Postoperative checkup included clinical examination and radiographs. The follow-up period ranged from 1 to 3 years following surgical debridement. The possible predisposing factors are also discussed in the article.

Results: Both cases healed uneventfully with no further symptoms. Radiographs revealed complete bone fill into the resected area and continuous stable bone levels around the previously affected implants.

Conclusions: It is concluded that recommendations for treatment of apical peri-implantitis are still minimal. In the present study, a surgical approach with resection of the apical portion of the affected implants in combination with debridement is suggested. Our experience was that partially resected oral implants remain osseointegrated and also function well clinically with a follow-up period up to 3 years.

KEY WORDS: implant, implant failure, periapical lesion, peri-implantitis

Endosseous oral implants have successfully been used during the last decades for facilitating the replacement of missing teeth in totally or partially edentulous patients.[1–4] Although considered a highly successful treatment modality, concerns have been raised in the literature regarding local infectious conditions in conjunction with oral implants. A more rare site for infection, involving the apical portion of the implants, has been described and named apical peri-implantitis.[5–9]

Apical peri-implantitis is often diagnosed by clinical findings such as pain, redness, tenderness, swelling, and sometimes the presence of a fistulous tract. Also, radiological findings such as periapical radiolucency around the apical area of the implant can be noted.[10] It should be distinguished from a clinically asymptomatic, periapical radiolucency, which is usually caused by implants that are shorter than the drilled implant site or by heat-induced aseptic bone necrosis.[10–12] Inactive lesions only need observation and monitoring. The infected type of lesion can originate from a variety of reasons, such as bacterial contamination during installation, premature loading with subsequent bone microfractures, or the

*Department ENT & Oral and Maxillofacial Surgery, NÄL Medical Centre Hospital, Trollhättan, Sweden; †Department of Biomaterials Sciences, Institute for Surgical Sciences, Sahlgrenska Academy University of Göteborg, Göteborg, Sweden; ‡Nicolas & Asp Postgraduate College of Dentistry, Dubai Health Care City, Dubai, United Arab Emirates

Reprint requests: Dr. Hossein Kashani, Department of Oral and Maxillofacial Surgery, NÄL Medical Centre Hospital, 461 85 Trollhättan, Sweden; e-mail: hossein.kashani@vgregion.se

© 2008, Copyright the Authors
Journal Compilation © 2008, Wiley Periodicals, Inc.

DOI 10.1111/j.1708-8208.2008.00108.x

presence of a preexisting inflammation. Such lesions have their origin at the apex of the implant but demonstrate the capability of spreading coronally, proximally, and buccally.[13] The information regarding the incidence of the described complication is scarce and mostly collected from case reports.[6–8,12,14]

However, in a recent study, Quirynen and colleagues[10] performed a retrospective study including 539 implants. They reported an incidence of apical peri-implantitis of 1.6% for the upper jaw and 2.7% for the lower jaw. Treatment options were also suggested. The protocol included elevation of a full-thickness flap, complete removal of granulation tissue with hand instruments, and curettage of the bony cavity walls.[10] Usually, these procedures are performed in conjunction with antibiotic treatment. These proposed interventions prevented further progression of the lesions, although complete remission was not obtained.

The aim of the present paper was to describe, in two cases, a surgical treatment protocol based on the concept described earlier with the addition of an apical resection of the affected implants.

MATERIAL AND METHODS

Two cases are presented, which were treated at the Department ENT & Oral and Maxillofacial Surgery, NÄL Medical Centre Hospital, Trollhättan, Sweden.

Case 1

In November 2003, a 20-year-old male patient was referred by his dentist for consultation regarding single-tooth replacement at the right upper lateral incisor. The patient was affected by agenesis 12 and 45 (FDI). No relevant past medical history was recorded except that the patient was allergic to penicillin. Prior to implant treatment, the patient had undergone a long orthodontic treatment successfully for extrusion of upper right canine that was impacted. To ensure a suitable emergence profile, a 13-mm Brånemark System TiUnite™ RP implant (Nobel Biocare AB, Göteborg, Sweden) was placed close to the right upper central incisor according to instructions from the referring dentist. The fixture was inserted with good primary stability according to standard protocol.

After 4 months of healing, the impression was taken at implant level and the restoration was fabricated using a direct individual abutment onto which a gold-ceramic crown was cemented. Clinical checkup and radiographs after 1 year in function revealed a periapical radiolucency at the apex of the implant and the neighboring tooth, which was sensitive for electrical pulp testing. The diagnosis apical peri-implantitis was made (Figure 1A). The patient did not demonstrate any clinical symptoms. In February 2005, the patient had developed fistulae buccally. The radiographic examination showed periapical destruction around the implant and the adjacent tooth #11 (see Figure 1A). Initially, systemic antibiotics (clindamycin 300 mg 3 times daily for 1 week) failed to subdue the infection (see Figure 1B).

Surgical Procedure. A full-thickness flap was elevated buccally. A large formation of granulation tissue was noted at the apex of the implant. The granulation tissue was removed and, thereafter, the free apical portion of the implant was resected by means of a fissure bur. Thorough irrigation with saline was performed during preparation. After final rinsing of the defect, the flap was repositioned and sutured (see Figure 1C). The patient received antibiotics (clindamycin 300 mg 3 times daily for 5 days) postoperatively. A 1-month checkup demonstrated no pain or discomfort for the patient. No sign of infection was noted. At 1-year follow-up, the patient remained asymptomatic. A periapical radiograph showed new bone formation at the apical region of the implant.

A 6-month follow-up showed increased radiographic bone density, and clinical reentry revealed the formation of bone not only around the implant but also along the root of tooth #11 (see Figure 1D). One year radiographic and clinical examination showed an uneventful healing with an acceptable aesthetic outcome and no clinical symptoms. (see Figure 1, E and F).

Case 2

A 28-year-old male patient was referred to the clinic with pain, redness, and swelling around implant in position 43. The implant had been in clinical function for 2 years. The patient had a history of trauma and tooth loss and subsequent implant treatment at the 43 region. The radiographic examination showed a radioluscency around the most apical part of the implant (Figure 2A). The implant was clinically stable and the neighboring teeth were considered healthy (see Figure 2A). Initially, the patient received antibiotics (clindamycin 300 mg, 3 times daily) and the patient was followed during 8 weeks. After this period, the patient was scheduled for

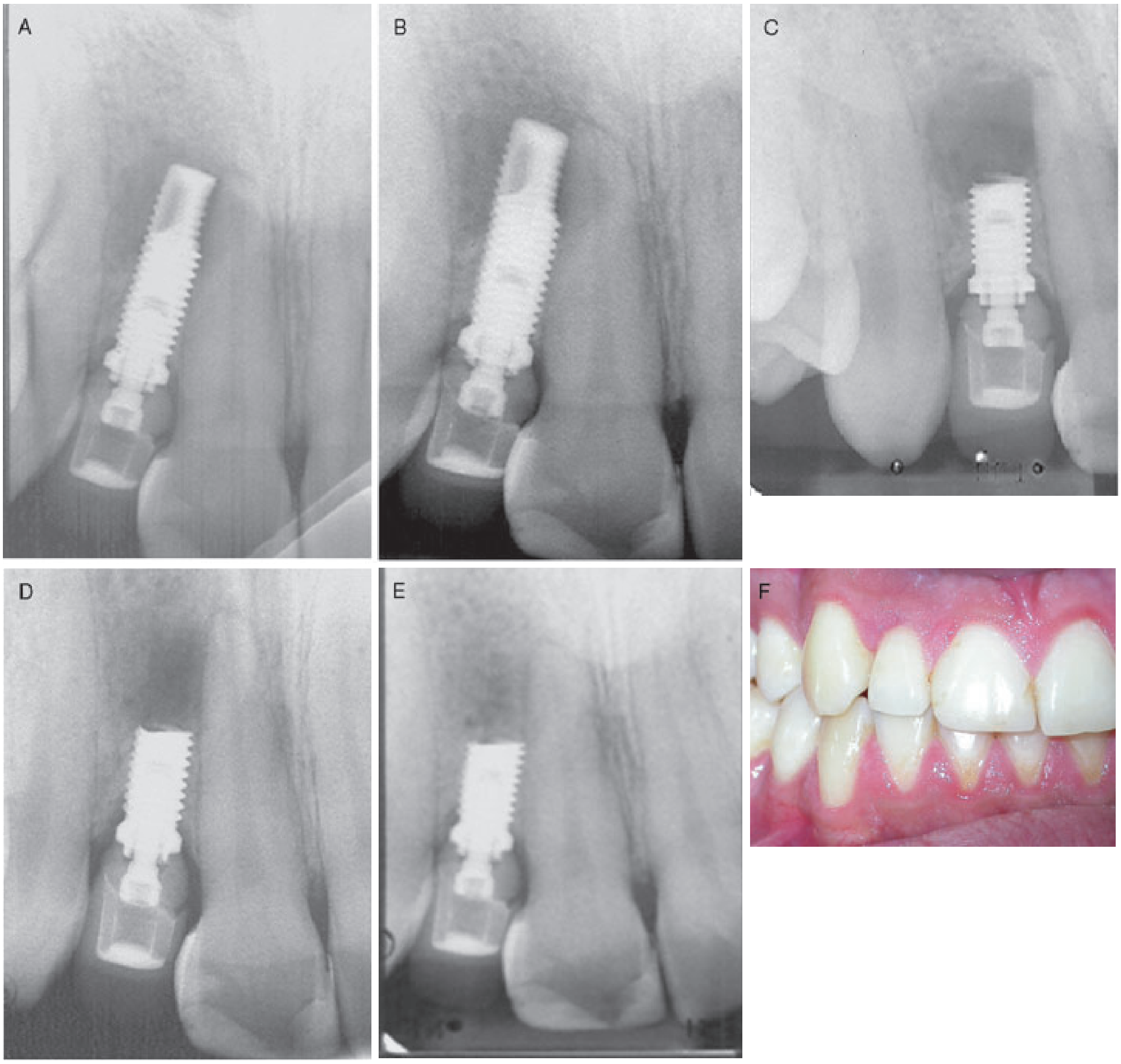

Figure 1 *A*, Implant replacing tooth #12 showing periapical destruction. The destruction is seen also around tooth #11. *B*, No healing despite antibiotic treatment. *C*, Postsurgical radiographic situation. *D*, Postoperatively 6 months, and *E*, 1-year follow-up radiographic situation. *F*, Clinical view 1 year postoperatively.

surgical exploration of the implant area (see Figure 2, B and C). Treatment comprised elevation of a full-thickness flap, curettage of the apical lesion, irrigation with saline, and removal of the apical portion of the implant (see Figure 2D). Granulation tissue was sent for histopathology analysis, which revealed a periapical inflammatory infection around the top of the implant.

At the 1-month follow-up, increased radiographic bone density could be observed, and clinical examination revealed the formation of bone not only around the implant but also along the root of tooth #11 (see Figure 2E).

The patient was followed at 3 months, 15 months, and 3 years postoperatively (see Figure 2, F–H, respectively), and further progress of bone healing was observed.

DISCUSSION

Even with improved technology with novel implant surfaces and strict guidelines for implant surgery and checkup, not all treatments are successful.[4] The periapical lesion around implants is a possible cause for implant failure. Quirynen and colleagues[10] suggest that some of the early failures may be linked with an endodontic pathology, either remaining after tooth

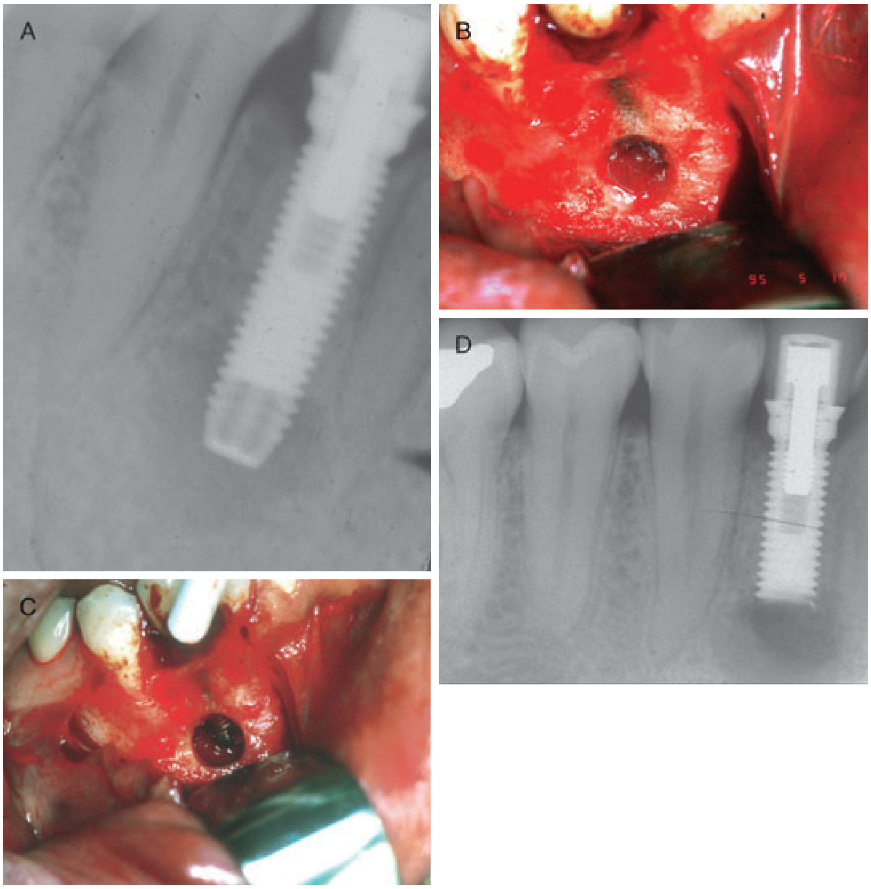

Figure 2 *A*, Implant replacing tooth in position 43. *B* and *C*, Surgical approach and apicectomy of the implant at the 43 region. *D*, Postoperative apical radiograph.

extraction or present around neighboring teeth. They also reported an almost three times higher incidence of failure for implants suffering from apical peri-implantitis when a history of endodontic pathology around previously extracted teeth were present. These findings corresponded well with our findings and have also been described in other studies.[6–9,13–18]

Implants with enhanced surface characteristics have been introduced to accelerate the osseointegration process. Several studies have confirmed a more rapid bone formation and implant surface contact as well as a high implant survival rate.[19–22] Although these types of implants clearly demonstrated a higher success rate, a higher incidence of retrograde peri-implantitis has been reported compared with the machined titanium surface.[10] The same authors also described an interesting hypothesis that makes a lot of sense. The machined implants, when coming in contact with a granuloma or endodontic pathology, will soon be surrounded by a thin layer of granulation tissue, clinically demonstrating nonosseointegration. Hence, implants with a modified surface will not have the same fate because of the rapid bone-to-implant surface response. The coronal part of such an implant still integrates before the fibrous encapsulation reaches this area. We agree in the statement that the higher incidence of implant failure at sites with a history of periapical granuloma requires a more detailed analysis of the radiographs prior to implant installation. It also raises the question of indications for immediate installation of implants into extraction sockets. A more conservative approach with delayed installation in cases with a history of apical pathology could be considered based on this theory.[23]

The recommendation for treatment of apical peri-implantitis is still scanty and mostly based on empirical data. In the study by Quirynen and colleagues,[10] it was suggested that removal of all granulation tissue was sufficient to prevent further progression of the disease. In our study, a more aggressive approach was recommended. As a result of the development with implants

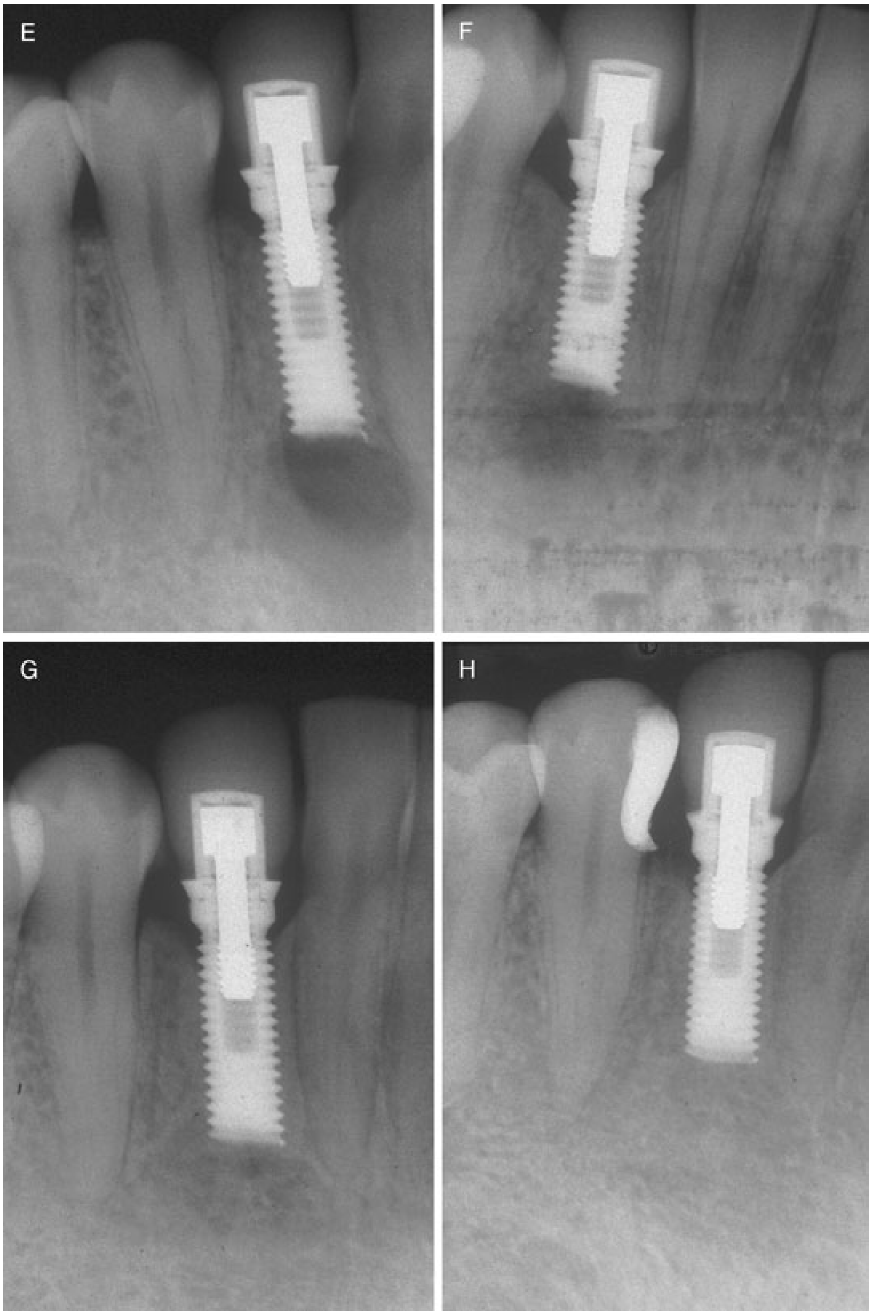

Figure 2 *E,* Radiograph checkup at 1 month. *F,* At 3 months, *G,* at 15 months, and *H,* at 3 years postoperatively. All show ongoing healing and increasing radiograph density.

with an enhanced, rough surface, this creates a *locus minoris*, with opportunities for the pathogens to remain in the area even after skillful debridement. As regeneration of the bone tissue also will not occur, further evaluation of the healing process by radiographs will be hampered. Within the limits of few cases, we propose a more extended surgical approach with surgical debridement and additional removal of the apical part of the affected implant. Our experience was that oral implants with the coronal portion intact remained osseointegrated and could successfully continue to function in the clinical setting.

REFERENCES

1. Brånemark P-I, Hansson BO, Adell R, et al. Osseointegrated implants in the treatment of the edentulous jaw. Experience from a 10-year period. Scand J Plast Reconstr Surg 1977; 16:1–132.

2. Adell R, Lekholm U, Rockler B, Brånemark P-I. A 15-year study of osseointegrated implants in the treatment of the edentulous jaw. Int J Oral Surg 1981; 10:387–416.
3. Jemt T, Lekholm U. Oral implant treatment in posterior partially edentulous jaws. A 5-year follow-up report. Int J Oral Maxillofac Implants 1993; 8:635–640.
4. van Steenberghe D, Quirynen M, Naert I. Survival and success rates with oral endosseous implants. In: Lang NP, Attstrom R, Lindhe J, eds. Proceedings of the 3rd European workshop on periodontology. Berlin: Quintessence, 1999: 242–254.
5. McAllister B, Masters D, Meffert R. Treatment of implants demonstrating periapical radiolucencies. Pract Proced Aesthet Dent 1992; 4:37–41.
6. Schaffer MD, Junuaz DA, Haggerty PC. The effect of periradicular endodontic pathosis on the apical region of adjacent implants. Oral Surg Oral Med Oral Pathol Oral Radiol Endod 1998; 86:578–581.
7. Ayangco L, Sheridan PJ. Development and treatment of retrograde peri-implantitis involving a site with a history of failed endodontic and apicectomy procedures: a series of reports. Int J Oral Maxillofac Implants 2001; 16:412–417.
8. Chaffee NR, Lowden K, Tiffee JC, Cooper LF. Periapical abscess formation and resolution adjacent to dental implants: a clinical report. J Prosthet Dent 2001; 85:109–112.
9. Quirynen M, Gijbels F, Jacobs R. An infected jawbone site compromising successful osseointegration. Periodontology 2000; 33:129–144.
10. Quirynen M, Vogels R, Alsaadi G, Naert I, Jacobs R, van Steenberghe D. Predisposing conditions for retrograde peri-implantitis, and treatment suggestion. Clin Oral Implants Res 2005; 16:599–608.
11. McAllister BS, Masters D, Meffert RM. Treatment of implants demonstrating periapical radioluscencies. Pract Proced Aesthet Dent 1992; 4:37–41.
12. Reiser GM, Nevins M. The implant periapical lesion. aetiology, prevention, and treatment. Compend Contin Educ Dent 1995; 16:768–772.
13. Green TL, Walton RE, Taylor JK, Merrell P. Radiographic and histologic periapical findings of root canal treated teeth in cadaver. Oral Surg Oral Med Oral Pathol Oral Radiol Endod 1997; 83:707–711.
14. Jalbout ZN, Tarnow DP. The implant periapical lesion: four case reports and review of the literature. Pract Proced Aesthet Dent 2001; 13:107–113.
15. Sussman HI, Moss SS. Localized osteomyelitis secondary to endodontic-implant pathosis. A case report. J Periodontol 1993; 64:306–310.
16. Piatelli A, Scarano A, Balleri P, Favero GA. Clinical and histological evaluation of an active "implant periapical lesion": a case report. Int J Oral Maxillofac Implants 1998; 13:713–716.
17. Brisman DL, Brisman AS, Moses MS. Implant failures associated with asymptomatic endodontically treated teeth. J Am Dent Assoc 2001; 132:191–195.
18. Shabang S, Bohsali K, Caplanis N, Torabinejad M. Effects of periradicular lesions on osseointegration of dental implants. J Endod 2001; 27:220–227.
19. Albrektsson T, Johansson C, Lundgren AK, Sul Y, Gottlow J. Experimental studies on oxidized implants. A histomorphometrical and biochemical analysis. Appl Osseointeg Res 2000; 1:121–124.
20. Gottlow J, Henry P, Tan A, Allan B, Johansson C, Hall J. Biomechanical and histologic evaluation of the TiUnite and Osseotite implant surfaces in dogs. Appl Osseointeg Res 2000; 1:25–27.
21. Friberg B, Billström C. Preliminary results of a prospective multicenter clinical study on TiUnite implants. Appl Osseointeg Res 2002; 3:29–31.
22. Friberg B, Dahlin C, Widmark G, Östman P-O, Billström C. One-year results of a prospective multicenter study on Brånemark System implants with TiUnite surface. Clin Implant Dent Relat Res 2005; 7:70–75.
23. Becker W, Dahlin C, Becker B, et al. The use of e-PTFE barrier membranes for bone promotion around titanium implants placed into immediate extraction sockets: A prospective multicenter study. Int J Oral Maxillofac Implants 1994; 9:31–40.
