# Supplementary material for: A novel histopathological classification of implant periapical lesion: A systematic review and treatment decision tree
Source: PLoS One. 2022 Dec 22;17(12):e0277387. doi: 10.1371/journal.pone.0277387 (PMC9778521; doi:10.1371/journal.pone.0277387)
Supplement: S1 File — (ZIP) [file pone.0277387.s001.zip › support files/Included study/Favia 2011.pdf]

Osteonecrosis of the Posterior Mandible after Implant Insertion: A Clinical and Histological Case Report

Gianfranco Favia, MD, DDS;* Adriano Piattelli, MD, DDS;† Pasquale Sportelli, DDS;*
Saverio Capodiferro, DDS;* Giovanna Iezzi, DDS, PhD†

ABSTRACT

Background: In the past few years, the occurrence of an oral lesion, called osteonecrosis of the jaw (ONJ), has been increasingly reported in patients undergoing treatment with bisphosphonates (BPs); however, few published histological studies of ONJ can be found in the literature.

Purpose: The aim of the present case was to report an occurrence of ONJ after implant insertion.

Materials and Methods: Multiple myeloma was diagnosed to a 65-year-old female. After 5 years of treatment with intravenous clodronate, two dental implants were inserted in the mandibular molar region. No preexisting bone lesions were present at a preoperative panoramic radiography. Before implant insertion, the patient had suspended the treatment with clodronate for 3 months. Four months after the implant insertion, a breakdown of the oral mucosa covering the implants occurred with a purulent discharge; periapical radiolucency was present around both implants.

An en-block resection on the alveolar bone including the two implants was performed. No signs of recurrence of the lesion were observed after a follow-up of 20 months.

Results: At the interface of one of the implants, a gap was observed between bone and implant. This bone was nonvital, and many osteocyte lacunae were empty. Moreover, this bone appeared to be partially demineralised. No newly formed bone or osteoblasts were present. Bone trabeculae were observed, on the other hand, within the apical implant threads of the other implant. A close connection was observed between this bone and the implant surface.

Discussion: The histological findings showed some areas with osseointegration in patients undergoing BP treatment for malignant disease; however, any invasive procedure can determine the onset of osteonecrosis.

Conclusion: In conclusion, there is certainly a temporal association between BP use and development of ONJ, but a correlation does not necessarily mean causation. Moreover, generalizations about this complex relationship cannot be made on the basis of a single case report. In patients undergoing intravenous treatment, clinicians must be aware of the increased risk of implant failure and, probably, implant insertion should be avoided at all until more conclusive data are available.

KEY WORDS: bisphosphonates, clodronate, dental implants, osteonecrosis of the jaw

INTRODUCTION

The primary indications for bisphosphonates (BPs) are osteoporosis and skeletal-related events in multiple myeloma, and breast and prostate cancers.[1,2] Other indications are Paget's disease of the bone, osteogenesis imperfecta, idiopathic juvenile osteoporosis, and severe steroid-induced osteoporosis.[3,4] BP can be administered either orally or intravenously.[5] There is evidence that internalization of BP in osteoclasts disrupts the cytoskeleton and vesicular trafficking, leading to cessation of resorption and induction of apoptosis.[3] BPs also have antiangiogenic effects, being able to decrease endothelial cell proliferation.[3] On a cellular level, BP may inhibit osteoclast function by:

1. inhibition of osteoclast recruitment,
2. diminution of osteoclast life span, and
3. inhibition of osteoclast activity at the bone surface.[2,6]

*Dental School, University of Bari, Italy; †Dental School, University of Chieti-Pescara, Italy

Reprint requests: Professor Adriano Piattelli, Via dei Vestini 31, 66100 Chieti, Italy; e-mail: apiattelli@unich.it

© 2009, Copyright the Authors
Journal Compilation © 2011, Wiley Periodicals, Inc.

DOI 10.1111/j.1708-8208.2009.00181.x

At a molecular level, it has been shown that BP influenced osteoclast activity through the modulation of a cell surface receptor or an intracellular enzyme.[2,3]

There is increasing evidence that BP may also positively influence the osteoblast.[7] However, high concentration of alendronate and zoledronate is cytotoxic for the osteoblasts.[8] A hypothesis of a combined effect of BP on the osteoclasts and the keratinocytes has been advanced.[9]

BPs bind avidly to the mineralized bone tissue at the sites of osteoclast lacunae and are then internalized by the osteoclast.[6,10] BPs have a selective concentration at the interface of the active osteoclast and the bone-resorption surface.[11] In the past few years, the occurrence of an oral lesion, called osteonecrosis of the jaw (ONJ), has been increasingly reported in patients undergoing treatment with BP. The precise incidence of ONJ remains unknown.[11] A cumulative hazard of 1% after 1 year, 10% after 2 years, and 20% after 3 years has been reported.[11] Respectively, 1.2 and 2.4% of patients with breast cancer and multiple myeloma, in treatment with intravenous BP, have been reported to develop ONJ.[12] A cumulative incidence of 0.8 to 12% in patients receiving intravenous BP for malignant disease has been reported[13] and, in a prospective study, 28% of patients developed ONJ.[11] Exposure with BP beyond 2.5 years may increase the risk of ONJ.[11] High cumulative doses of BP, poor oral health, and dental extractions may be considered as risk factors.[12] ONJ is much less common with lower oral doses for osteoporosis.[14] Among several million patients with osteoporosis who have received oral BP, 50 ONJ events have been reported.[14] One event per 100,000 person-years has been calculated for exposure to oral BP.[14] The hallmark of ONJ is the finding of exposed bone in the oral cavity.[15] Patients may be considered to have ONJ if all of the following three features are present:

1. Current or previous treatment with a BP
2. No history of radiation therapy to the jaws
3. Exposed necrotic bone in the maxilofacial region that has persisted for more than 8 weeks.[13]

Clinically, ONJ is characterized by the presence of ulcerated mucosa and exposed, white-yellow, devitalized bone.[16] The surrounding soft tissues are often inflamed because of a secondary mucosal infection.[13,16] Pain, oral discomfort, purulent discharge, exudates and fistula are common.[11,13,16,17]

Tooth extractions were the predominating event preceding ONJ,[18] although other causes, such as periodontal disease, dental implant procedures, and ill-fitting dentures, were also reported.[14]

Few published histological studies of ONJ can be found in the literature. These studies have shown vital cells and bone in more than half the patients,[14] pronounced inflammatory changes,[6,14] bone necrosis and infection,[2,15] minimal presence of Howship lacunae, congested venules and bacterial infiltrate within deep bone trabeculae,[19] and an absence of osteoblasts or vascularization.[16]

The aim of the present case was to report an occurrence of ONJ after implant insertion.

CASE REPORT

Multiple myeloma was diagnosed in 1996 to a 65-year-old female. At the beginning of 2002, the patient started a treatment with intravenous clodronate (300 mg twice per month). In January 2007, two dental implants were inserted in the mandibular molar region. No preexisting bone lesions were present at a preoperative panoramic radiography (Figure 1). Before implant insertion, the patient had suspended the treatment with clodronate for 3 months. Four months after the implant insertion, a breakdown of the oral mucosa covering the implants occurred with a purulent discharge; in a panoramic radiography, a periapical radiolucency was present around both implants (Figure 2).

In local anesthesia, an en-block resection on the alveolar bone including the two implants, with lateral and deep margins in an apparently healthy bone, was performed (Figure 3), and the flaps were accurately sutured. The patient underwent a strict clinical and radiological follow-up, and after 20 months, no signs of recurrence of osteonecrosis were detectable.

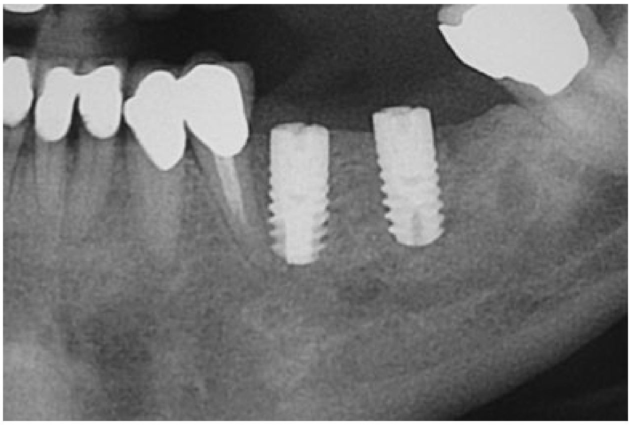

Figure 1 Preoperative panoramic radiograph showing no signs of osteonecrosis.

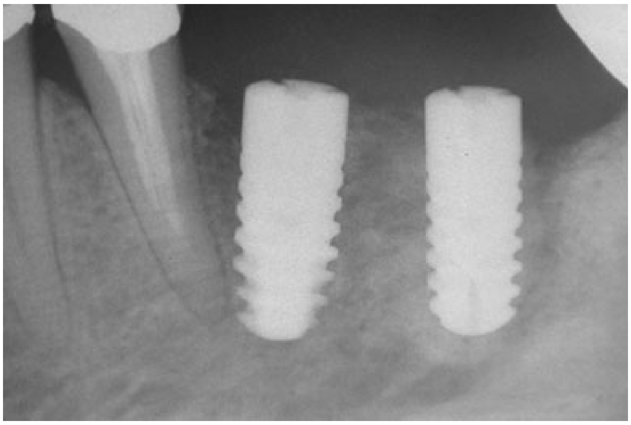

Figure 2 Periapical radiograph 4 months after implant insertion. A peri-implant radiolucency was present around both implants.

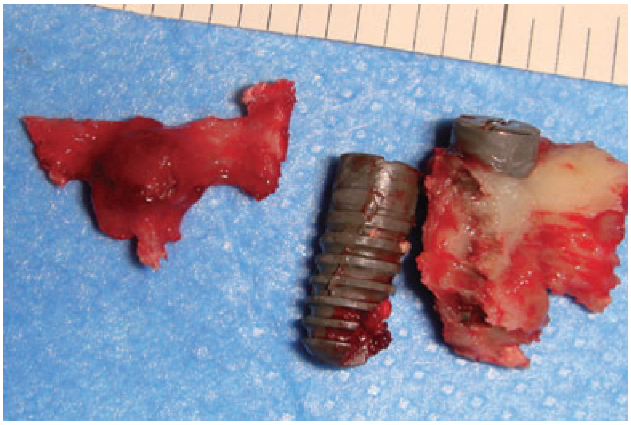

Figure 4 The surgical specimen: an implant presented bone only in the apical portion while the other implant seemed to be still surrounded by bone.

The surgical specimen was sent for microscopical evaluation (Figure 4). The implants and the surrounding tissues were stored immediately after removal in 10% buffered formalin and processed to obtain thin ground sections with the Precise 1 Automated System (Assing, Rome, Italy).[20] The specimens were dehydrated in an ascending series of alcohol rinses and embedded in a glycolmethacrylate resin (Technovit® 7200 VLC, Heraeus Kulzer GmbH & Co., Wehrheim, Germany). After polymerization, the specimens were sectioned longitudinally along the major axis of the implants with a high-precision diamond disk at about 150 μm and ground down to about 30 μm. Three slides were obtained. The slides were stained with acid fuchsin and toluidine blue.

Histomorphometry was carried out using a light microscope (Laborlux S, Ernst Leitz GmbH, Wetzlar, Germany) connected to a high-resolution video camera (3CCD, JVC KY-F55B, JVC, Yokohama, Japan) and interfaced to a monitor and PC (Intel® Pentium® III 1200 MMX, Intel Corporation, Santa Clara, CA, USA). This optical system was associated with a digitizing pad (Matrix Vision GmbH, Oppenweiler, Germany) and a histometry software package with image-capturing capabilities (Image-Pro® Plus Version 4.5, Media Cybernetics Inc., Immagini & Computer Snc, Milan, Italy).

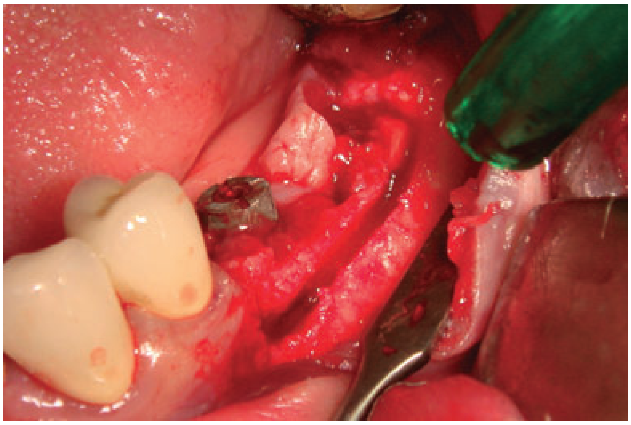

Figure 3 Intraoperative view of the affected alveolar bone. An en-block resection was performed including the two implants, with lateral and deep margins in an apparently healthy bone.

RESULTS

Implant A

At low power modification, bone was present around the implant (Figure 5). At a higher magnification, a gap was observed between bone and implant. This bone was nonvital, and many osteocyte lacunae were empty. Moreover, this bone appeared to be partially demineralized. No newly formed bone or osteoblasts were present. No osteoclasts or Howship lacunae were observed. In some areas of the interface, it was possible to see a connective tissue with an inflammatory cell infiltrate (Figure 6).

Implant B

At low power magnification, bone tissue was found around the last three apical threads of the implant (Figure 7). At a higher magnification, bone trabeculae were observed within the apical implant threads. A close connection was observed between this bone and the implant surface (Figure 8). Osteoid matrix was present in some portions of the interface; osteoblasts were

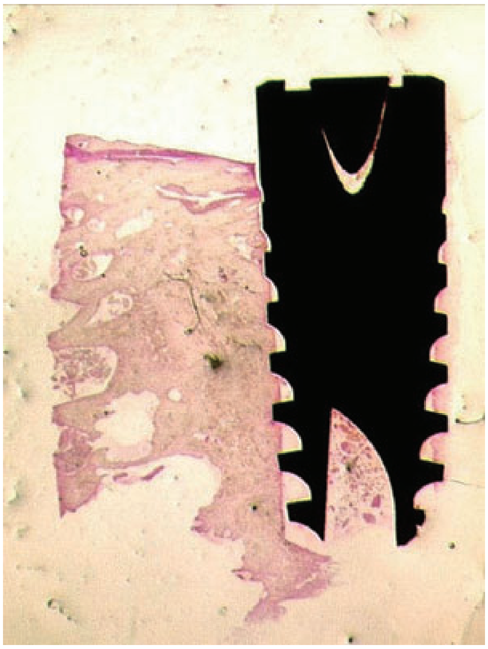

Figure 5 At low power magnification, around one implant, it is possible to observe demineralized peri-implant bone tissue. A gap is present between the bone and the implant surface, and in some fields there is a connective tissue between these two structures (acid fuchsin and toluidine blue; 10×).

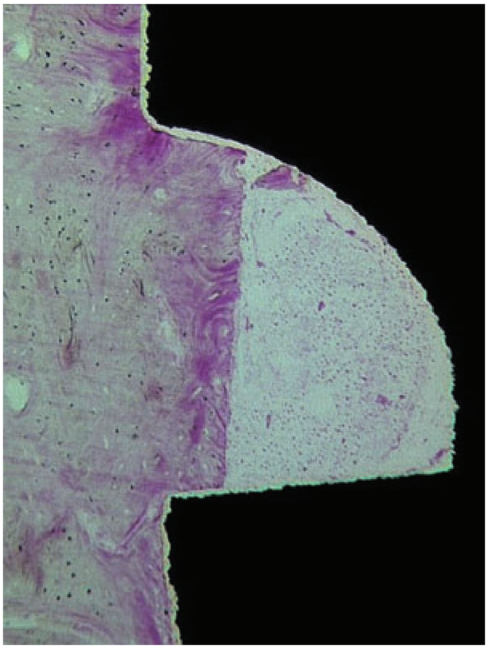

Figure 6 At higher magnification, in some areas of the interface, a gap was present between implant and bone. The bone appeared nonvital, partially demineralized, and with many empty osteocyte lacunae. In some areas of the interface, a connective tissue with an inflammatory cell infiltrate was observed (acid fuchsin and toluidine blue; 40×).

absent. It was also possible to observe bone detached and at a distance from the implant surface. Newly formed bone trabeculae undergoing remodeling were present in this area. Osteoid matrix was present in many portions; no osteoblasts were present. These bone trabeculae were lined by a loose connective tissue with many spindle cells, plasma cells, and many inflammatory cells. Some small vessels were present; their wall was composed by many endothelial cells.

DISCUSSION

The pathogenesis of ONJ is still unclear.[11] In most cases, the pathogenesis seems to be consistent with a defect in jawbone physiologic remodeling or wound healing.[13]

The profound inhibition of osteoclast function can also inhibit normal bone turnover to an extent that local microdamage from normal mechanical loading or injury (tooth extractions) cannot be repaired.[13] There is also evidence of an inhibitory effect of BP on the keratinocyte cell cycle, which might promote a mucosal breakdown.[6] Moreover, BPs diminish levels of vascular endothelial growth factor, and in rat decreased rate of capillary formation was found.[6,21] The thin oral mucosa can be easily traumatized during surgical procedures, allowing oral bacteria to track into the necrotic bone.[21]

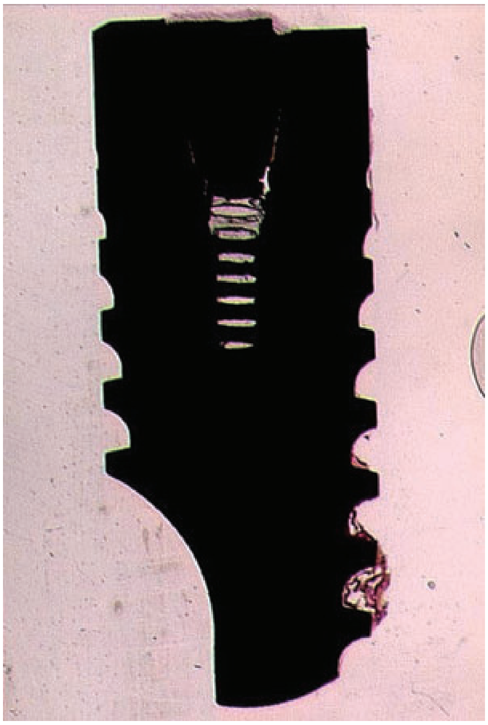

Figure 7 At low power magnification, bone is present only inside the three most apical threads of the other implant (acid fuchsin and toluidine blue; 10×).

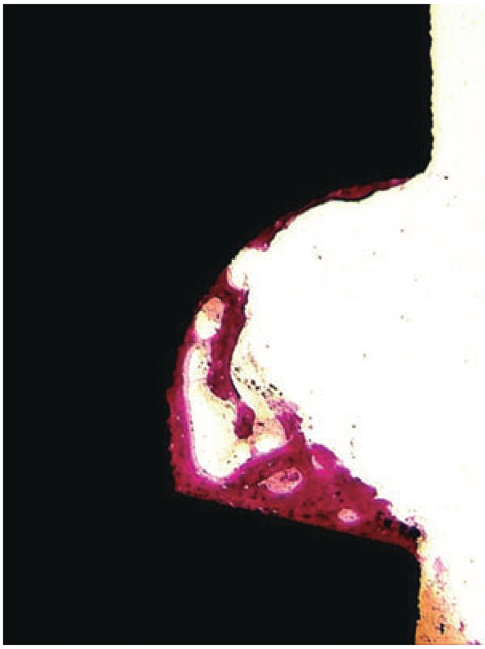

Figure 8 At higher magnification, bone trabeculae were observed within the apical implant threads. A close connection was observed between this bone and the implant surface. Osteoid matrix was present in some portions of the interface; osteoblasts were absent. Newly formed bone trabeculae undergoing remodeling were present in this area. Osteoid matrix was present in many portions; no osteoblasts were present (acid fuchsin and toluidine blue; 40×).

Furthermore, BP causes cessation of bone remodeling and bone turnover.[21] The inability of osteoclasts to resorb old bone causes the osteoblast and the osteocyte to die, leaving an acellular bone matrix. This is followed by degeneration of the small capillaries, avascularity, and high susceptibility to microfractures.[21] Mandible bone marrow stromal cells were found to be more susceptible to pamidronate than iliac crest cells based on decreased cell survival, lower alkaline phosphatase production, and structurally less-organized in vivo bone regeneration.[10]

Moreover, BPs tend to be highly concentrated in the jaws rather than in other skeletal sites because of their high vascularity and bone turnover.[13,21] ONJ has never been found in bones outside the craniofacial skeleton.[13]

The few published histological studies of ONJ show vital cells and bone in more than half the patients, which suggests a lack of necrosis.[14] The histological studies all show pronounced inflammatory changes, represented by a mixed cellular infiltration (neutrophils, lymphocytes, plasma cells),[6,14,16] infection with bacterial debris,[2,14] minimal presence of Howship lacunae, congested venules and bacterial infiltrate within the deep trabeculae,[19] scarce quantity of osteoblasts and vascularization,[16] and fibrosis of marrow spaces.[6]

There is evidence that BP is a contraindication to oral implants[22] like all oral surgical procedures. It is best to avoid all elective oral surgery in patients on BP, including endosseous implant placement[22] above all in patients taking intravenous BP.

In contrast, other researchers did not find evidence of ONJ in patients undergoing oral BP treatment and who had received dental implants.[6,23] Moreover, no causal relationship has been well established between BP treatment and implant failure.[6,23]

However, it is important that clinicians be aware of the potential risk in treating patients under BP treatment, with a hypothetical increased risk for implant failure and delayed wound healing[2,19,24] both for intravenous and oral BP therapy.

The present histological findings, that is, the presence both of well-osseointegrated portions of the implant with a close connection with the surrounding bone and of a gap between bone and implant, with connective tissue and inflammatory cells at the interface, and with the presence of nonvital bone, demonstrate that implant osseointegration can occur in patients undergoing BP treatment for malignant disease and that, on the other hand, any invasive procedure can determine the onset of osteonecrosis.

BPs are probably involved in the development of ONJ.[25] Found in the literature are mainly reported cases of ONJ treated with amino-BP, that is, zoledronic acid, pamidronate, and ibandronate.[26] Reports of ONJ with clodronate have been sporadic, and the risk for ONJ with clodronate use appears to be low.[26] Moreover, ONJ may be caused by combined, and environmental and genetic risk factors.[25] For example, BP-related ONJ has been found to be associated with polymorphisms of the cytochrome P450 in multiple myeloma.[25] Cytochrome P450 plays a role in a key biologic metabolic cascade, which apparently plays a relevant role in osteoblastic differentiation.[25]

In conclusion, there is certainly a temporal association between BP use and development of ONJ, but a correlation does not necessarily mean causation.[26] Moreover, generalizations about this complex relationship cannot be made on the basis of a single case report.

In patients undergoing intravenous treatment, clinicians must be aware of the increased risk of implant

failure and, probably, implant insertion should be avoided at all, until more conclusive data are available.

ACKNOWLEDGMENTS

This work was partially supported by the National Research Council, Rome, Italy, and by the Ministry of Education, University Research, Rome, Italy.

REFERENCES

1. Kimmel DB. Mechanism of action, pharmacokinetic and pharmacodynamic profile, and clinical applications of nitrogen-containing bisphosphonates. J Dent Res 2007; 86:1022–1033.
2. Ruggiero SL, Mehrotra B, Rosenberg TJ, Engroff SL. Osteonecrosis of the jaws associated with the use of bisphosphonates: a review of 63 cases. J Oral Maxillofac Surg 2004; 62:527–534.
3. Ficarra G, Beninati F, Rubino I, et al. Osteonecrosis of the jaws in periodontal patients with a history of bisphosphonate treatment. J Clin Periodontol 2005; 32:1123–1128.
4. Landesberg R, Cozin M, Cremers S, et al. Inhibition of oral mucosal cell wound healing by bisphosphonates. J Oral Maxillofac Surg 2008; 66:839–847.
5. Bell BM, Bell RE. Oral bisphosphonates and dental implants: a retrospective study. J Oral Maxillofac Surg 2008; 66:1022–1024.
6. Hansen T, Kunke M, Weber A, Kirkpatrick CJ. Osteonecrosis of the jaws in patients treated with bisphosphonates – histomorphologic analysis in comparison with infected osteoradionecrosis. J Oral Pathol Med 2006; 35:155–160.
7. Von Knoch F, Eckardt C, Alabre CI, Schneider E, Rubash HE, Shanbag AS. Anabolic effects of bisphosphonates on peri-implant bone stock. Biomaterials 2007; 28:3549–3559.
8. Naidu A, Dechow PC, Spears R, Wright JM, Kessler HP, Oppermann LA. The effect of bisphosphonates on osteoblasts in vitro. Oral Surg Oral Med Oral Pathol Oral Radiol Endod 2008; 106:829–837.
9. Magopoulos C, Karakinaris G, Telioudis Z, et al. Osteonecrosis of the jaws due to bisphosphonate use. A review of 60 cases and treatment proposals. Am J Otolaryngol 2007; 28:158–163.
10. Stefanik D, Sarin J, Lam T, Levin L, Leboy PS, Akintoye SO. Disparate osteogenic response of mandible and iliac crest bone marrow stromal cells to pamidronate. Oral Dis 2008; 14:465–471.
11. Boonyapakorn T, Schirmer I, Reichart PA, Sturdy I, Massenkeil G. Bisphosphonate-induced osteonecrosis of the jaws: prospective study of 80 patients with multiple myeloma and other malignancies. Oral Oncol 2008; 44:857–869.
12. Hoff AO, Toth BB, Altundag K, et al. Frequency and risk factors associated with osteonecrosis of the jaw in cancer patients treated with intravenous bisphosphonates. J Bone Miner Res 2008; 23:826–836.
13. Ruggiero SL, Drew SJ. Osteonecrosis of the jaws and bisphosphonate therapy. J Dent Res 2007; 86:1013–1021.
14. Rizzoli R, Burlet N, Cahall D, et al. Osteonecrosis of the jaw and bisphosphonate treatment for osteoporosis. Bone 2008; 42:841–847.
15. Reid IR, Bolland MJ, Grey AB. Is bisphosphonate-associated osteonecrosis of the jaw caused by soft tissue toxicity? Bone 2007; 41:318–320.
16. Migliorati CA, Schubert MM, Peterson DE, Seneda LM. Bisphosphonate-associated osteonecrosis of mandibular and maxillary bone: an emerging oral complication of supportive cancer therapy. Cancer 2005; 104:83–93.
17. Bedogni A, Blandamura S, Lokomic Z, et al. Bisphosphonate-associated jawbone osteonecrosis: a correlation between imaging techniques and histopathology. Oral Surg Oral Med Oral Pathol Oral Radiol Endod 2008; 105:358–364.
18. Bagan JV, Murillo J, Jimenez Y, et al. Avascular jaw osteonecrosis in association with cancer chemotherapy: series of 10 cases. J Oral Pathol Med 2005; 34:120–123.
19. Wang HL, Weber D, McCauley LK. Effect of long-term oral bisphosphonates on implant wound healing: literature review and a case report. J Periodontol 2007; 78:584–594.
20. Piattelli A, Scarano A, Quaranta M. High-precision, cost-effective system for producing thin sections of oral tissues containing dental implants. Biomaterials 1997; 18:577–579.
21. Sarin J, De Rossi SS, Akintoye SO. Updates on bisphosphonates and potential pathobiology of bisphosphonate-induced jaw osteonecrosis. Oral Dis 2008; 14:277–285.
22. Scully C, Madrid C, Bagan J. Dental endosseous implants in patients on bisphosphonate therapy. Implant Dent 2006; 15:212–218.
23. Grant BT, Amenedo C, Freeman K, Kraut RA. Outcomes of placing dental implants in patients taking oral bisphosphonates: a review of 115 cases. J Oral Maxillofac Surg 2008; 66:223–230.
24. Otomo-Corgel J. Implants and oral bisphosphonates: risky business. J Periodontol 2007; 74:373–376.
25. Sarasquete ME, Garcia-Sanz R, Marin L, et al. Bisphosphonate-related osteonecrosis of the jaws is associated with polymorphisms of the cytochrome P450 CYP2C8 in multiple myeloma: a genome-wide single nucleotide polymorphism analysis. Blood 2008; 112:2709–2712.
26. Diehl IJ, Fogelman I, Al-Nawas B, et al. Pathophysiology, risk factors and management of bisphophonate-associated osteonecrosis of the jaw: is there a diverse relationship of amino- and non-aminobisphophonates? Crit Rev Oncol Hematol 2007; 64:198–207.
