# Supplementary material for: A novel histopathological classification of implant periapical lesion: A systematic review and treatment decision tree
Source: PLoS One. 2022 Dec 22;17(12):e0277387. doi: 10.1371/journal.pone.0277387 (PMC9778521; doi:10.1371/journal.pone.0277387)
Supplement: S1 File — (ZIP) [file pone.0277387.s001.zip › support files/Included study/Galzignato 2010.pdf]

Dental implant failure associated with a residual maxillary cyst

P-F. Galzignato,[1] S. Sivolella,[2] G. Cavallin[3] and G. Ferronato[4]

VERIFIABLE CPD PAPER

IN BRIEF

- Implant success and survival depend on various factors including pre-surgical diagnosis and evaluation of general dental conditions.
- Anatomical variations must be known and recognised in order to plan implant surgery.
- Recent scientific data suggest the use of shorter implants in certain conditions rather than performing advanced implant procedures.

PRACTICE

A 53-year-old male underwent oral implantation including bone grafting to substitute tooth 12. Three months post-operatively, the implant migrated apically into a cyst cavity. The implant, cyst and bone graft were removed. This report regards the migration of an implant into a residual cyst; some anatomical, clinical and pathological considerations arise from this case.

CASE REPORT

A 53-year-old male patient came to our attention because, three months following insertion of an implant in position 12 with a small amount of heterologous bone implant (osteotome technique) (Fig. 1), the referring dentist attempted a second surgery phase (healing screw insertion), but, because of the lack of implant osteointegration, the implant migrated apically into a cavity. A dental implant in the maxillary sinus was then suspected. A CT scan was prescribed for the following reasons: (1) to check the diagnosis; (2) to determine the seriousness of the infection and the anatomical structures involved; (3) to plan the surgical procedure for recovery of the foreign body; (4) for legal-medical reasons. An endoral radiography would not have revealed whether the implant was in the maxillary sinus or in another cavity.

The computerised tomography showed the presence of a radiolucent area of approximately 2 cm in diameter periapically to the missing 12 tooth, involving the nasopalatine duct, and an implant inside the area. The bony wall of the maxillary sinus was maintained, without signs of sinusitis (Fig. 2).

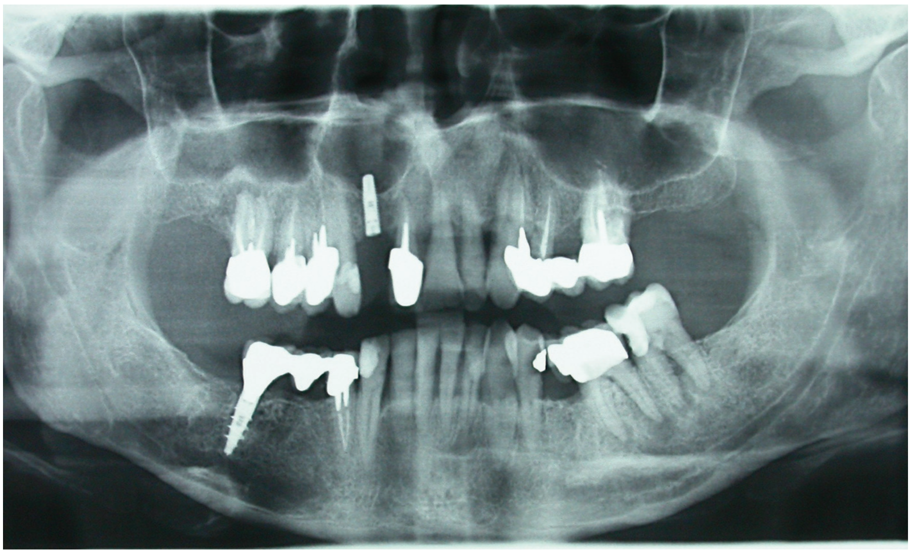

Fig. 1 Panoramic radiography at the time of implant surgery illustrating the implant in place in position 12 and a radiolucent area apically to the implant. The apical portion of the implant penetrates the inferior cortical layer of the radio-transparent area. The transparent area may be misdiagnosed as an extremely anterior recess of the sinus cavity

TREATMENT

The implant and the upper right maxillary cyst were removed under local anaesthesia through a transoral approach. Bone graft material particles were also found in the cystic wall. The histological report gave evidence for a residual odontogenic inflammatory cyst, characterised by a thick, irregular, often incomplete, squamous epithelium, with granulation tissue forming the cyst wall in the denuded areas. The fibrous capsule was infiltrated by chronic inflammatory cells. Graft particles were visible, surrounded by collagen fibres in the cystic wall.

The post-operative course was satisfactory and without complications.

DIFFERENTIAL DIAGNOSIS

The general dental conditions observed in the panoramic film could suggest a diagnosis of residual inflammatory cyst.

The differential diagnosis was therefore the following: migration of the dental implant in (i) residual inflammatory cyst or (ii) upper recess of the maxillary sinus or (iii) nasopalatine duct cyst or (iv) globullomaxillary cyst.

In the case reported, the implant did not migrate into the maxillary sinus, as in the case described by Iida *et al.*,[1] but into a residual inflammatory cyst cavity deriving from the previously extracted tooth 12.

[1,3]Department of Maxillofacial Surgery; [2*]DDS, Clinical Assistant, Department of Oral Surgery; [4]Head of the Department of Maxillofacial Surgery, University of Padova, Institute of Clinical Dentistry, Padova, Italy, Via Venezia, 90, 35100 Padova, Italy
*Correspondence to: Stefano Sivolella
Email: stefano.sivolella@libero.it

Refereed Paper
Accepted 8 June 2009
DOI: 10.1038/sj.bdj.2010.156
©British Dental Journal 2010; 208: 153–154

© 2010 Macmillan Publishers Limited. All rights reserved.

The reported case suggests some considerations. The presence of a cyst mistaken for an anterior recess of the maxillary sinus represents a diagnostic error. Despite this, due to anatomical variability, the anterior recess of the maxillary sinus can be advanced up until the periapical area of tooth 12, as described by Cuenin *et al.*[2] Trimarchi *et al.*[3] describe the *Pneumosinus dilatans* as a rare condition involving one or more of the paranasal sinuses and define it as an abnormal focal or generalised dilatation of the sinus, which is air-filled and does not present functional alterations or bony wall defects.

A dental implant positioned in the anterior maxilla may also be dislocated in another type of pre-existing cyst, such as a nasopalatine duct cyst.[4] The choice of risking damage to the sinus wall using the osteotome technique with a bone graft in a site with a residual alveolar process sufficient for inserting an implant of a slightly shorter length, with a prognosis often equal to a longer implant, can be criticised.[5]

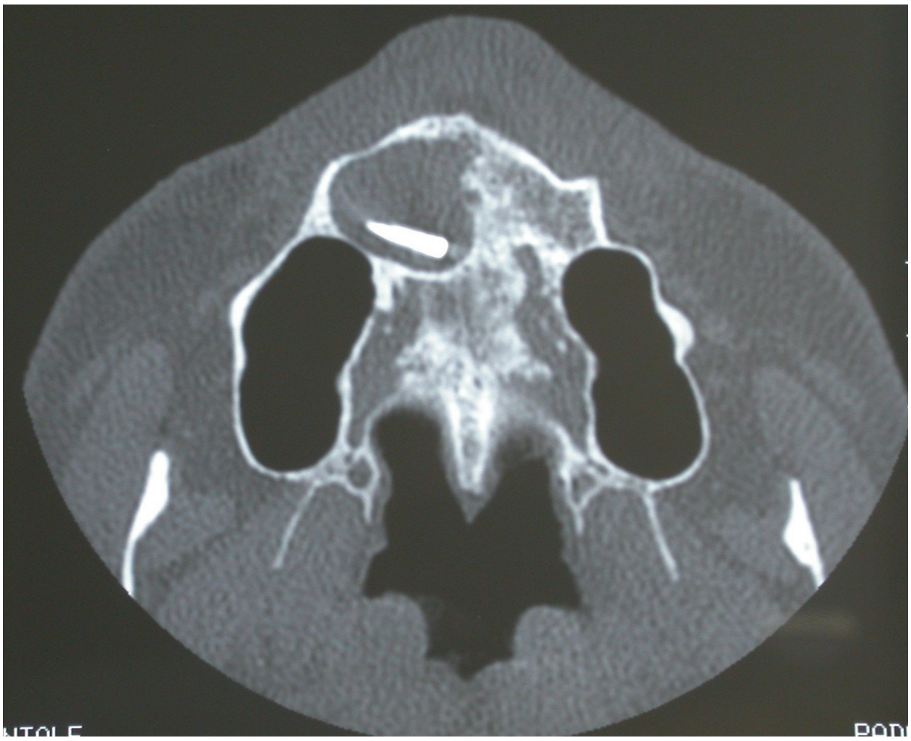

Fig. 2 Axial CT scan three months after implant surgery. It demonstrates the presence of an upper right maxillary cyst and a dental implant migrated into it. The cyst involves the naso-palatine duct: a differential diagnosis between a residual cyst and a cyst arising from the duct cells could be suspected. No pathologies affect the sinus cavities

COMMENT

A correct clinical and radiological preoperative diagnosis, even in apparently simple cases, must be made taking anatomical variability and general dental conditions into consideration. The possibility of onset of post-implant cystic lesions in the upper front maxillary sector is also to be remembered. Finally, the evaluation of the correct implant dimensions must be taken into account, so as to avoid performing unnecessary operations.

1. Iida S, Tanaka N, Kogo M, Matsuya T. Migration of a dental implant into the maxillary sinus. A case report. *Int J Oral Maxillofac Surg* 2000; **29:** 358-359.
2. Cuenin M F, Pollard B K, Elrod C W. Maxillary sinus morphology in differential dental diagnosis. *Gen Dent* 1996; **44:** 328-331.
3. Trimarchi M, Lombardi D, Tomenzoli D, Farina D, Nicolai P. Pneumosinus dilatans of the maxillary sinus: a case report and review of the literature. *Eur Arch Otorhinolaryngol* 2003; **260:** 386-389.
4. Casado P L, Donner M, Pascarelli B, Derocy C *et al.* Immediate dental implant failure associated with nasopalatine duct cyst. *Implant Dent* 2008; **17:** 169–175.
5. Nedir R, Bischof M, Briaux J M, Beyer S *et al.* A 7-year life table analysis from a prospective study on ITI implants with special emphasis on the use of short implants. Results from a private practice. *Clin Oral Implants Res* 2004; **15:** 150-157.

© 2010 Macmillan Publishers Limited. All rights reserved.
