# Supplementary material for: A novel histopathological classification of implant periapical lesion: A systematic review and treatment decision tree
Source: PLoS One. 2022 Dec 22;17(12):e0277387. doi: 10.1371/journal.pone.0277387 (PMC9778521; doi:10.1371/journal.pone.0277387)
Supplement: S1 File — (ZIP) [file pone.0277387.s001.zip › support files/Included study/Kim 2013.pdf]

FINAL CONSIDERATIONS

The hybrid obturator prosthesis restored the aesthetic and functional satisfaction and improved the patient's quality of life. Furthermore, the prosthesis manufacture was successful because of the interaction of adequate clinical and laboratorial techniques. This fact facilitated the insertion and removal of the prosthesis, promoting greater stability and retention to the stomatognathic system.

REFERENCES

1. Abadi BJ, Byron RJ Jr. Maxillary obturator: a clinical case report. *Gen Dent* 2008;56:709–713
2. Koyama S, Sasaki K, Inai T, et al. Effects of defect configuration, size, and remaining teeth on masticatory function in post-maxillectomy patients. *J Oral Rehabil* 2005;32:635–641
3. Goiato MC, Santos DM, Moreno A, et al. Upper lip prosthesis retained to a palatal obturator: a clinical report. *J Craniofac Surg* 2011;22:e59–e62
4. Laine J, Vahatalo K, Peltola J, et al. Rehabilitation of patients with congenital unrepaired cleft palate defects using free iliac crest bone grafts and dental implants. *Int J Oral Maxillofac Implants* 2002;17:573–580
5. De Rezende ML, Amado FM. Osseointegrated implants in the oral rehabilitation of a patient with cleft lip and palate and ectodermal dysplasia: a case report. *Int J Oral Maxillofac Implants* 2004;19:896–900
6. Burns DR. The mandibular complete overdenture. *Dent Clin North Am* 2004;48:603–623
7. Goiato MC, Fernandes AU, dos Santos DM, et al. Positioning magnets on a multiple/sectional maxillofacial prosthesis. *J Contemp Dent Pract* 2007;8:101–107
8. Gumus HO, Tuna SH. An alternative method for constructing an obturator prosthesis for a patient with a bilateral cleft lip and palate: a clinical report. *J Esthet Restor Dent* 2009;21:89–95
9. Wieselmann-Penkner K, Arnetzl G, Mayer W, et al. Minimizing movement of an orbital prosthesis retained by an obturator prosthesis. *J Prosthet Dent* 2004;91:188–190
10. Cheng AC, Somerville DA, Wee AG. Altered prosthodontic treatment approach for bilateral complete maxillectomy: a clinical report. *J Prosthet Dent* 2004;92:120–124
11. Lopes JF, Pinto JH, de Almeida AL, et al. Cleft palate obturation with brånemark protocol implant-supported fixed denture and removable obturator. *Cleft Palate Craniofac J* 2010;47:211–215
12. Zarb G, Bolender C, Eckert S, et al. *Prosthodontic treatment for edentulous patients*. 12th ed. St Louis: CV Mosby 2004
13. McGivney GP, Carr AB, McCracken WL. *McCracken's removable partial prosthodontics*. 10th ed. St Louis: Mosby 2000
14. Dimashkieh MR, Morgano SM. A procedure for making fxed prosthodontics impressions with the use of preformed crown shells. *J Prosthet Dent* 1995;73:95–96
15. Goiato MC, Panzarini SR, Tomiko C, et al. Temporary flexible immediately removable partial denture: a case report. *Dent Today* 2008;27:114–116
16. Goiato MC, Garcia AR, Dos Santos DM, et al. Analysis of masticatory cycle efficiency in complete denture wearers. *J Prosthodont* 2010;19:10–13
17. Almog DM, Ganddini MR. Maxillary and mandibular overlay removable partial dentures for restoration of worn teeth. A three-year follow-up. *N Y State Dent J* 2006;72:32–35
18. Dawson PE. *Evaluation, diagnosis and treatment of occlusal problems*. 2nd ed. St Louis: Elsevier, 1989
19. De Carvalho WR, Barboza EP, Caula AL. Implant-retained removable prosthesis with ball attachments in partially edentulous maxilla. *Implant Dent* 2001;10:280–284
20. Goiato MC, dos Santos DM, Moreno A, et al. Prosthetic treatments for patients with oronasal communication. *J Craniofac Surg* 2011;22:1445–1447
21. Brignoni R, Dominici JT. An intraoral-extraoral combination prosthesis using an intermediate framework and magnets: a clinical report. *J Prosthet Dent* 2001;85:7–11
22. Goiato MC, Santos DM, Villa LM. Obturator for rehabilitation of cleft palate with implant-supported retention system. *J Craniofac Surg* 2010;21:151–154
23. Chambers MS, Lemon JC, Martin JW. Obturation of the partial soft palate defect. *J Prosthet Dent* 2004;91:75–79
24. Arigbede AO, Dosumu OO, Shaba OP, et al. Evaluation of speech in patients with partial surgically acquired defects: pre and post prosthetic obturation. *J Contemp Dent Pract* 2006;7:89–96

Postoperative Maxillary Cyst After Maxillary Sinus Augmentation

Jae Jin Kim, PhD, DDS, Marcelo Freire, PhD, DDS,† Jung-Hoon Yoon, PhD, DDS,‡ Hak Kyun Kim, PhD, DDS**

Abstract: The posterior edentulous maxilla is a critical anatomic region for dental implant therapy. Because of severe alveolar bone resorption and maxillary sinus pneumatization, low bone volume is often presented clinically. Although maxillary sinus augmentation has been developed to promote bone reconstruction and oral rehabilitation, complications have been reported. Possible complications include paranasal sinusitis, loss of the graft, and displacement of an implant into the antrum. In this study, we present an observed rare complication of maxillary sinus augmentation, a postoperative maxillary cyst that occurred 10 years after treatment.

Key Words: Maxillary sinus augmentation, dental implant, postoperative maxillary cyst

An adequate quality and quantity of the alveolar bone are required for the reconstruction of bone for dental implant surgeries. The edentulous posterior maxilla often presents insufficient bone because of alveolar bone resorption after tooth loss and the pneumatization of the maxillary sinus. Over the years, numerous methods have been developed for the management of the edentulous posterior maxilla for dental implants.[1] To increase the amount of bone in the posterior maxilla, Boyne and James[2] first reported the maxillary sinus augmentation technique, which has become a common surgical procedure. However, various complications related to maxillary sinus augmentation, including paranasal sinusitis, oroantral fistula, loss of the graft, and an implant displacement into the sinus, can lead to failure of dental implants.[3] Those complications are mainly attributed to a tear or perforation of the schneiderian membrane.

From the *Department of Oral and Maxillofacial Surgery, Research Institute for Medical Science, College of Medicine, Chungnam National University, Daejeon, Republic of Korea; †Department of Oral Medicine, Infection and Immunity, Harvard School of Dental Medicine, Boston, Massachusetts; and ‡Department of Oral and Maxillofacial Pathology, College of Dentistry, Wonkwang Bone Regeneration Research Institute, Daejeon Dental Hospital, Wonkwang University, Daejeon, Republic of Korea.
Received March 27, 2013.
Accepted for publication June 23, 2013.
Address correspondence and reprint requests to Hak Kyun Kim, DDS, PhD, Department of Oral and Maxillofacial Surgery, Research Institute for Medical Science, College of Medicine, Chungnam National University, 55 Munwha-ro, Jung-gu, Daejeon 301-747, Republic of Korea; E-mail: hkkim4022@cnu.ac.kr
The authors report no conflicts of interest.
Copyright © 2013 by Mutaz B. Habal, MD
ISSN: 1049-2275
DOI: 10.1097/SCS.0b013e3182a238a4

Copyright © 2013 Mutaz B. Habal, MD. Unauthorized reproduction of this article is prohibited.

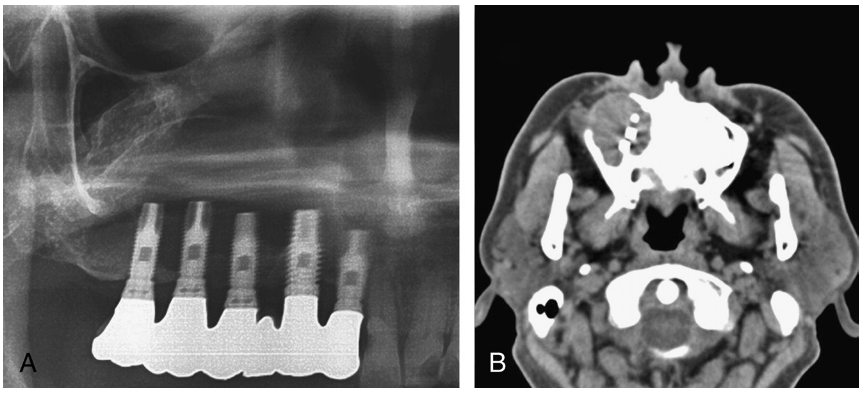

FIGURE 1. At initial visit. A, Radiographic analysis of unilocular radiolucency around the dental implant fixtures. B, Axial computed tomographic scan of the cystic lesion surrounding the dental implants in the grafted area.

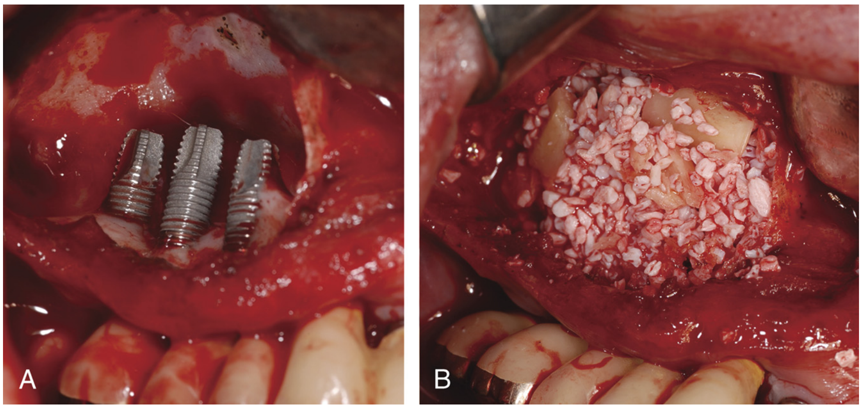

FIGURE 2. Intraoperative view. A, The dental implants inside the maxillary bone after the enucleation of the cyst. B, The grafted materials (Bio-Oss + autogenous bone) in the defect.

Here, we present an unusual complication, a postoperative maxillary cyst (POMC), after maxillary sinus augmentation and the placement of dental implants.

CLINICAL REPORT

A 60-year-old South Korean man presented with a complaint of masticatory difficulty with a slow-growing, painless tumorous lesion in his right palate in February 2010. Results of the oral examination disclosed a fluctuant swelling in the right posterior palate with a normal overlying mucosa. Results of the radiographic examinations including panoramic radiography and computed tomographic scan demonstrated a well-delineated unilocular radiolucency surrounding 4 of 5 dental implant fixtures (Figs. 1A, B).

His dental record indicated that the corticocancellous bone from the iliac crest was grafted into the right maxillary sinus floor in March 1999. Five months after the graft, 5 dental implants were placed in the grafted site. An implant-supported prosthesis was made in March 2000. Since then, the patient has not been visited our clinic.

During a surgical procedure in March 2010, a lesion was surrounded by the maxillary bone and out of contact with the sinus membrane and the involved dental implants showed an osseointegration with the remaining alveolar bone (Fig. 2A). The lesion was enucleated without removal of any implant, and the defect was filled with a xenograft (Bio-Oss; Geistlich Pharma, Wolhusen, Switzerland) and the autogenous cortical bone from the mandibular ramus (Fig. 2B).

Results of the histopathologic examination revealed that the lesion was a cyst, characterized by a pseudostratified ciliated columnar epithelium and a partly stratified squamous epithelium. There were some inflammatory cells in the cyst wall (Fig. 3). A pathologic diagnosis of a POMC was made.

The patient's postoperative course was uneventful. Results of the routine physical examination and panoramic radiography showed no problems related to the lesion until 2 years after the surgery.

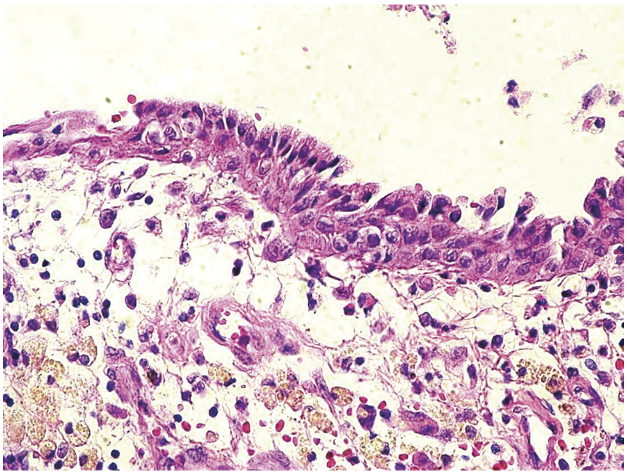

FIGURE 3. Histopathologic analysis of the cystic lesion. Tissue is lined by a pseudostratified ciliated columnar epithelium with a subepithelial inflammatory reaction (hematoxylin-eosin, original magnification × 200).

© 2013 Mutaz B. Habal, MD

Copyright © 2013 Mutaz B. Habal, MD. Unauthorized reproduction of this article is prohibited.

DISCUSSION

This current study illustrated an exceptional complication of the maxillary sinus augmentation. To our knowledge, there have been 2 previous reports of the POMC after maxillary sinus augmentation, which developed 6 months and 3 years after surgery.[4,5] However, the cyst in the current study was detected while the dental implants are functioning for 10 years. This late detection was attributed to a lack of symptoms presented by the patient and inadequate recall.

The previously reported POMC arises in the maxilla as a delayed complication of radical surgical intervention including Caldwell-Luc operation, and the cyst is thought to arise from the entrapment of epithelial remnants in the surgical site during closure.[6] Therefore, 1 hypothesis is that a tearing or perforation of the maxillary sinus membrane occurred during the sinus elevation in the current study. In addition, it is possible that an entrapped sinus membrane in a large amount of the grafted bone was responsible for the development of the cyst. Unfortunately, the surgeon might not have detected the perforation of the membrane while placing the graft into the maxillary sinus.

There is a traditional protocol for the lateral approach, which suggests the lateral approach rather than the osteotome sinus floor elevation technique when the residual bone height is less than 5 mm.[7] However, the osteotome technique is simply executed and less invasive than the lateral approach is. Successful osteotome technique or crestal approach and simultaneous implants installation in the posterior maxilla with less than 3-mm residual bone height have been reported.[8,9] The challenge with the osteotome technique or the crestal approach is that a perforation or tear in the schneiderian membrane during the surgery may not be detected by the surgeon, especially when dental implants or graft materials are placed into the maxillary sinus through the crestal osteotomy sites. Although the POMC arose from the maxillary sinus augmentation using the lateral approach technique in the current study, a large amount of graft using the osteotome technique is at more risk for a development of a POMC.

To prevent a perforation or tear of the maxillary sinus membrane, a careful elevation of the membrane with adequate access to the surgical site is important. If a tear occurs, any mucosal remnants adhering to the sinus floor or being embedded in the graft should be completely eliminated to prevent a development of a cystic lesion. Furthermore, there should be long-term follow-ups for all the patients who have undergone maxillary sinus augmentation to recognize a lesion suggestive of a cyst as early as possible.

CONCLUSIONS

The incidence of POMC after sinus augmentation and implant surgery is rare. The presentation of this current study demonstrates evidence of an additional complication after bone augmentation of the maxillary sinus. Further investigative follow-up of patients is necessary to increase the understanding of etiology, incidence, and therapy.

REFERENCES

1. Raja SV. Management of the posterior maxilla with sinus lift: review of techniques. *J Oral Maxillofac Surg* 2009;67:1730–1734
2. Boyne PJ, James RA. Grafting of the maxillary sinus floor with autogenous marrow and bone. *J Oral Surg* 1980;38:613–616
3. Barone A, Santini S, Sbordone L, et al. A clinical study of the outcomes and complications associated with maxillary sinus augmentation. *Int J Oral Maxillofac Implants* 2006;21:81–85
4. Misch CM, Misch CE, Resnik RR, et al. Post-operative maxillary cyst associated with a maxillary sinus elevation procedure: a case report. *J Oral Implantol* 1991;17:432–437
5. Lockhart R, Ceccaldi J, Bertrand JC. Postoperative maxillary cyst following sinus bone graft: report of a case. *Int J Oral Maxillofac Implants* 2000;15:583–586
6. Gardner D, Gullane P. Mucoceles of the maxillary sinus. *Oral Surg Oral Med Oral Pathol* 1986;62:538–543
7. Jensen OT. Treatment planning for sinus grafts. In: Jensen OT, ed. *The Sinus Bone Graft*. Illinois: Quintessence, 1999:49–68
8. Nedir R, Nurdin N, Szmukler-Moncler S, et al. Osteotome sinus floor elevation technique without grafting material and immediate implant placement in atrophic posterior maxilla: report of 2 cases. *J Oral Maxillofac Surg* 2009;67:1098–1103
9. Kim DY, Itoh Y, Kang TH. Evaluation of the effectiveness of a water lift system in the sinus membrane-lifting operation as a sinus surgical instrument. *Clin Implant Dent Relat Res* 2012;14:585–594

Five-Year Follow-up of Modified Implant-Supported Overdenture in an Iliac Crest Autograft Failure: Clinical Report

*Rodrigo dos Santos Pereira, DDS, MSc,**
*Fernanda Brasil Daura Jorge Boos, DDS, MSc,**
Eduardo José Veras Lourenço, DDS, MSc,†
*Eduardo Hochuli-Vieira, DDS, MSc,**
Paulo da Costa Rodrigues, DDS,‡
Nicolas Homsi, DDS, MSc§

Abstract: The treatment of extensive pathologic lesions in the jaw, most of the time, can generate rehabilitation problems to the patient. The solid ameloblastoma is a locally invasive odontogenic tumor with a high recurrence rate. Its treatment is aggressive and accomplished through resection with safety margin. The criterion standard for reconstruction is autogenous bone, but it can provide a high degree of resorption, causing inconvenience to the patient because of lack of rehabilitative option. This study aimed to describe a patient with ameloblastoma treated through resection and reconstruction with autogenous bone graft, in which, after an extensive resorption of the graft was made, a modified bar was applied to support a prosthetic implant overdenture.

Key Words: Dental implants, ameloblastoma, dental prosthesis, mandible

Most of mandibular deformities are consequences of tumor radical surgical treatments; mandibular reconstruction on these

From the *Department of Surgery and Integrated Clinic, Dental School of Araçatuba, Universidade Estadual Paulista UNESP, São Paulo; †Department of Periodontics, Veiga de Almeida University, Rio de Janeiro; ‡Department of Diagnosis and Surgery, Araraquara Dental School, São Paulo State University, São Paulo; ‡Military Police Hospital of Rio de Janeiro State; and §Department of Oral and Maxillofacial Surgery, Fluminense Federal University, Rio de Janeiro, Brazil.
Received March 28, 2013.
Accepted for publication June 23, 2013.
Address correspondence and reprint requests to Rodrigo dos Santos Pereira, DDS, MSc, Department of Surgery and Integrated Clinic, Dental School of Araçatuba, São Paulo State University, Rua José Bonifácio, 1193, Bloco 10A, 86020-030, Araçatatuba, São Paulo, Brazil;
E-mail: rd2502@yahoo.com.br
The authors report no conflicts of interest.
Copyright © 2013 by Mutaz B. Habal, MD
ISSN: 1049-2275
DOI: 10.1097/SCS.0b013e3182a23777

Copyright © 2013 Mutaz B. Habal, MD. Unauthorized reproduction of this article is prohibited.
