# Supplementary material for: A novel histopathological classification of implant periapical lesion: A systematic review and treatment decision tree
Source: PLoS One. 2022 Dec 22;17(12):e0277387. doi: 10.1371/journal.pone.0277387 (PMC9778521; doi:10.1371/journal.pone.0277387)
Supplement: S1 File — (ZIP) [file pone.0277387.s001.zip › support files/Included study/Manfro2018.pdf]

Apicoectomy and Scanning Electron Microscopy Analysis of an Implant Infected by Apical (Retrograde) Peri-implantitis: A Case Letter

Rafael Manfro, DDS, MSC, PhD[1]
Gislaine Felipe Garcia, DDS, MSC[1]
Marcelo Carlos Bortoluzzi, DDS, MSC, PhD[2]
Vinicius Fabris, DDS, MSC[3]
Atais Bacchi, DDS, MSC, PhD[4]*
Carlos Nelson Elias, DDS, MSC, PhD[5]

Introduction

The loss of 1 or more teeth is considered a physical and psychological trauma for most individuals, regardless of age or social class. Immediate implant insertion is an excellent alternative to substitute teeth indicated for extraction.[1] Previous studies showed that immediate implants placed on alveolar bone compromised by periapical diseases have similar success rates in comparison to immediate implants placed on healthy alveolar bone.[1–5] In cases of chronically infected apical alveolar bone, a severe curettage of the lesion followed by chemical cleaning of the alveolar bone is necessary, as otherwise there is risk of retrograde peri-implantitis.[2,6–8] A relevant number of reports of infected implants in chronically infected apical alveolar bone have been observed in literature.[9–11]

Macllister et al[12] were the first to show clinical cases of periapical lesions on the implants. These lesions were described later as "apical peri-implantitis" or "retrograde peri-implantitis."[13] In 2013, a new classification for these lesions was proposed considering the type of lesion and its etiology related to the neighboring teeth.[11] These lesions are usually identified through the examination of images that exhibit radiolucent areas associated with implants or through painful symptoms reported by patients. More rarely, the area of these lesions may be swollen, with pus and/or fistula.[11,13,14]

Several reasons may explain the appearance of a lesion in the implant periapical region. Among the most common are bone overheating during milling, implant contamination, a bone cavity longer than the implant, and implant placement immediately after extraction of a tooth with an untreated or unhealed preexisting lesion.[13,15] The periapical lesion may also be a result of lesions in neighboring teeth that induce contamination of the implant surface.[11]

There are several reports of periapical lesion in the literature, but an adequate treatment protocol with a successful prognosis for these lesions had not yet been proposed.[13,14] The use of implant apicoectomy is a common treatment,[15] although some authors showed similar results with only bone curettage.[16]

This case letter reports on an apically infected implant that was immediately placed in a failed endodontic site. The protocol proposed was to remove the infected portion of the implant due to the difficulty to access the lesion, disinfect the bone with curettage and irrigation, and maintain the part of the implant that exhibited osseointegration. This report also evaluates the need for debridement in those infected sites before implant placement.

Case Report

A female patient, 45 years old, sought treatment for pain in the area of tooth No. 9. The patient related that over the past year, the tooth was extracted and an implant was placed. The patient reported occurrence of pain since implant placement. Initially, the pain was caused upon chewing, but lately it had evolved into spontaneous pain reoccurring several times per day. This implant was placed immediately after the extraction of a tooth that had root perforation and an intracanal post.

No changes in the region were observed on clinical examination, and the mucosa over the implant was normal in terms of color, continuity, and volume. On imaging examination, however, a well-defined unilocular radiolucent lesion was observed on the implant apex toward the palate (Figure 1). By examining the medical records provided by the professional who performed the treatment, before the implant placement, the extracted tooth showed a radiolucent lesion associated with the tooth drilling, most likely of endodontic origin. The implant had a size of 4.0-mm diameter × 15-mm length.

Initially, an antibiotic (cephalexin 500 mg, 12/12 hours) was

[1] Departament of Oral Implantology, SOEBRAS, Florianópolis, SC, Brazil.
[2] Department of Dentistry, State University of Ponta Grossa–UEPG, Ponta Grossa, PR, Brazil.
[3] Department of Oral Surgery, Meridional Faculty–IMED, Passo Fundo, RS, Brazil.
[4] Department of Prosthodontics, Meridional Faculty–IMED, Passo Fundo, RS, Brazil.
[5] Biomaterials Laboratory, Military Institute of Engineering, Rio de Janeiro, RJ, Brazil.
* *Corresponding author,* e-mail: atais_bacchi@yahoo.com.br
DOI: 10.1563/aaid-joi-D-16-00162

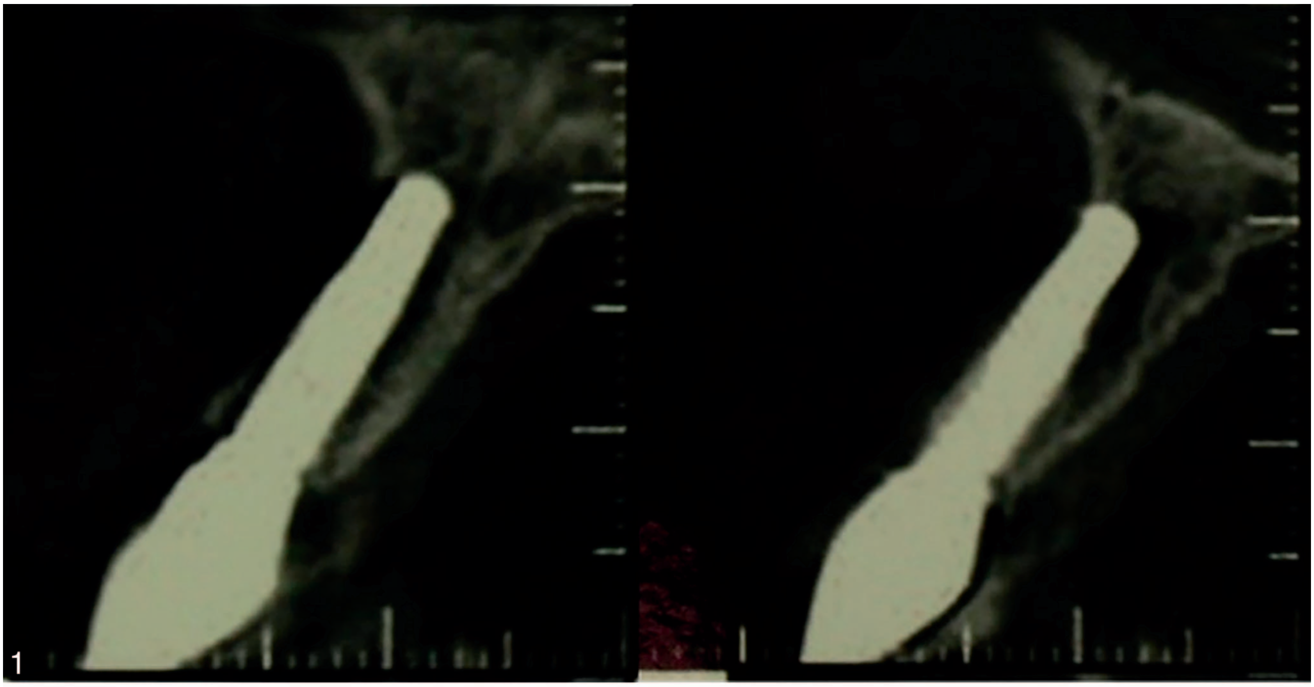

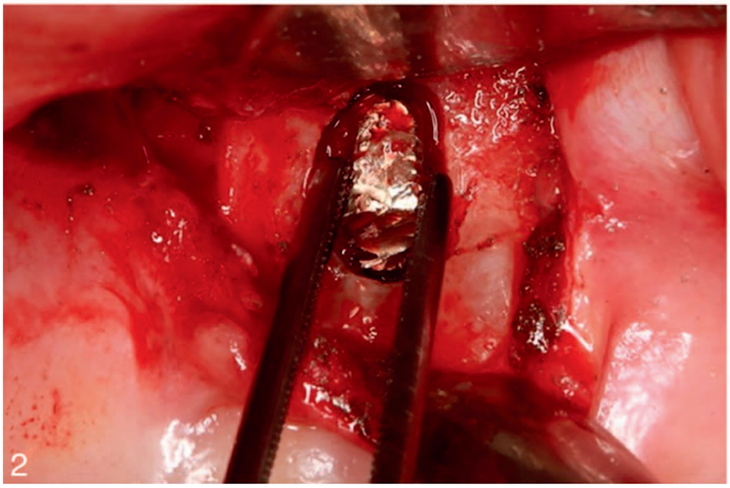

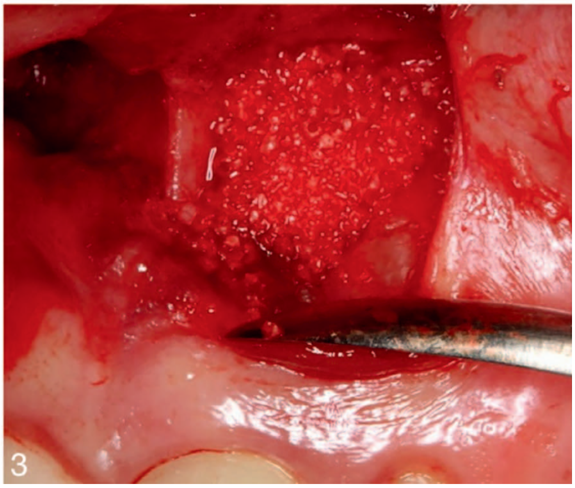

Figures 1–3. Figure 1. Cone-beam tomography of the region of tooth No. 9 where the radiolucent lesion was observed. **Figure 2.** Removal of the apical part of the implant. **Figure 3.** Surgical defect with bone substitute.

used for 7 days. The patient reported a decrease in pain in the beginning, but after the end of the medication period, the pain returned with the same intensity.

The surgery to remove the lesion and the implant apex was then elected. The patient signed an informed consent. A sulcular access was performed to preserve the gingival contour. Subsequently, a full-thickness flap with a subperiosteal detachment was performed followed by an osteotomy to access the apical part of the implant. The implant was sectioned using a transmetal milling bur (Ref 671798, Dentsply Maillefer, Ballaiguess, Switzerland) with a 5-mm cutting surface set at high speed under copious irrigation of saline at the cervical region of the lesion. After cutting the implant and removing the apical fragment, the bone cavity could be accessed. Little fibrous content was found, and much was debrided by curettage. Figure 2 shows tissue adhesion to the implant.

The bone cavity was washed and irrigated with saline. Then, the region was treated with pH-neutral 24% ethylenediaminetetraacetic acid (EDTA) root surface conditioner (PrefGel, Straumann, Basel, Switzerland) for 2 minutes, filled with a alloplastic biphasic calcium phosphate material (BoneCeramic, Straumann), and subsequently coated with an absorbable collagen haemostatic sponge (Hemospon, Technew, Rio de Janeiro, Brazil; Figure 3). Despite partial removal of the implant, the remaining size (about 7 mm) was considered functionally sufficient to hold the prosthesis.

The implant fragment was packed in an adequate vial and sent for examination by scanning electron microscopy (SEM). In the images obtained, bacterial colonies could be observed in the removed region (Figures 4 and 5). The implant was not osseointegrated in the chronically infected apical alveolar bone.

In the first days after the implant apex was removed, the

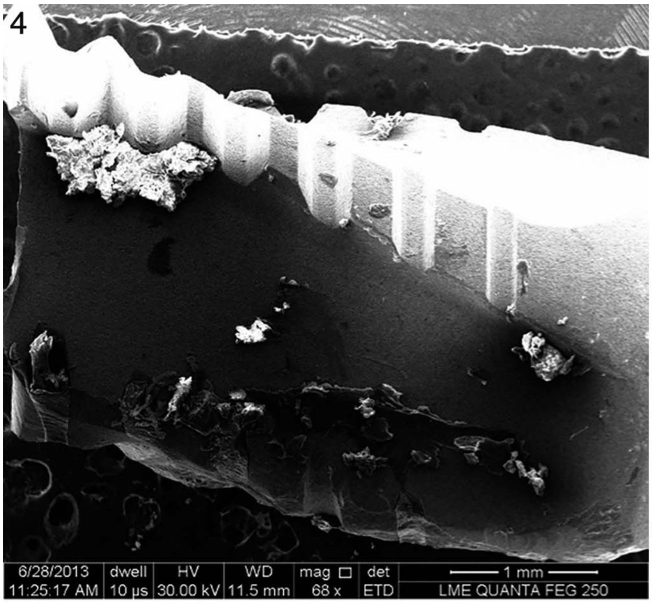

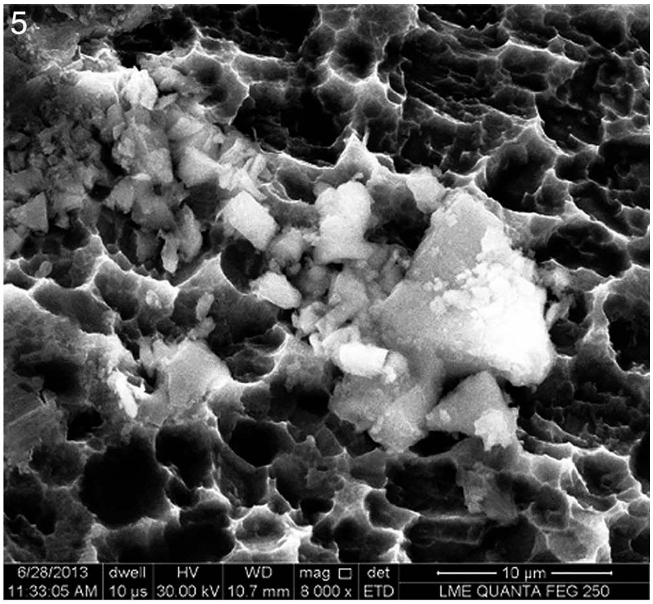

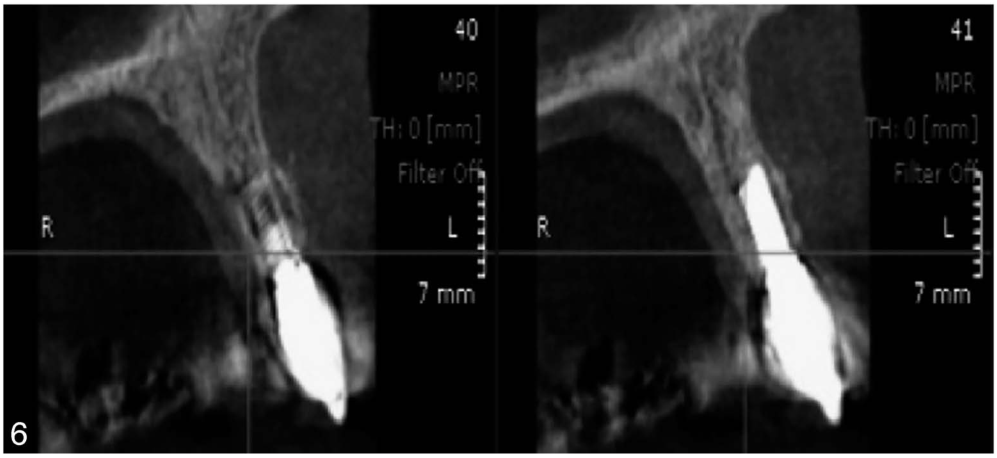

Figures 4–6. Figure 4. Scanning electron microscopy (SEM) image at ×68 in which a colony of bacteria can be observed adhered to the implant surface. **Figure 5.** SEM at ×8.000 indicating the contaminated area of the implant. **Figure 6.** Cone-beam tomography 4 months after surgery.

patient reported that pain slightly decreased. Painful symptoms decreased over time, and 4 months after the intervention, the patient did not report any pain symptoms (Figure 6).

After 6 months, the patient was clinically uneventful. Images obtained by computerized tomography demonstrated complete new bone formation in the previously infected area. The patient has been followed for 5 years without symptomatology.

Discussion

Periapical lesions are uncommon in implants.[8] When these lesions are diagnosed by clinical examination or found in imaging examinations, the professional should determine their etiology and treat them to prevent a possible implant loss.[6,8–11] In general, these lesions are associated with an implant placed in the region of a previous endodontically treated tooth. As reported, the apex of the endodontically treated tooth is not aseptic.[6] Therefore, a correct curettage with debridement of the extraction socket and bacterial culprits is necessary. If the debridement is not (or incorrectly) performed, the bacteria can remain, and an inflammation might start later, as in the present case.

The principal bacteria identified in endodontic failed sites are *Enterococcus faecalis*, *Peptostreptococcus micros, Fusobacterium necrophorum*, *Fusobacterium nucleatum*, *Prevotella intermedia/nigrescens*, *Porphyromonas gingivalis*, and *Porphyromonas endodontalis*.[17] After tooth extraction, these bacteria might assume a vegetative state if the chronically infected alveolar bone is not adequate disinfected.[18,19] Subsequently, these bacteria might not be eliminated by immunity or antibiotic therapy during bone healing because the biofilm assumes a protective form by means of a surficial coating.[18,19] Therefore, even when the alveolar bone is considered healed, the vegetative bacteria present might be reactivated and colonize the implant surface, and symptomatic inflammation take place after days or even months.[6,18,19]

Implants with lesions and large healthy bone can be treated to maintain the fixation.[13–16] Several treatment options have been reported. Curettage of the chronically infected apical alveolar bone with complete removal of all granulation tissue may resolve some cases.[7,8,16] In other cases, removing part of the implant (apicoectomy) is required.[7,8,15] The choice will depend on the difficulty of removing the lesion and on the possibility of new osseointegration of the region. In the case presented, apicoectomy was chosen mainly because of the difficulty in accessing the lesion, which was in the palatal area of the implant. Implants with a small healthy area must be removed and replaced with another implant.[14,15] In the present case, the implant remained with a length of 7 mm after apicoectomy. Studies have shown adequate outcomes for short implants supporting single crowns with regard to survival and success.[20,21] In the mentioned studies, short implants were considered those with a length <10 mm. However, one study highlighted the increased risk of using implants with a length <6 mm.[21]

Intense irrigation is necessary during the cutting of the implant apex. Heating of the bone in the surrounding region must be prevented so that the temperature does not reach values higher than 47°C and bone necrosis does not occur.[22,23]

The SEM analysis allowed observation that the bacterial colony adheres firmly to the implant surface and that the location of the bacterial colony delimits the implant osseointegration area (ie, from the colony area to the implant apex, there was no osseointegration area). Previous studies have observed the need to focus on periapical microbiota to establish a specific etiology and differential diagnoses in comparison to marginal peri-implantitis and related lesions.[6,8]

Some articles have demonstrated success with curettage of the chronically infected apical alveolar bone.[7,8,12] However, the removal of the contaminated implant portion might be desirable depending on the location of the contamination. Bacteria might remain attached on the implant surface when curettage is performed and the contaminated portion of the implant is maintained. Therefore, the contaminated area might be unlikely to lead to new osseointegration.

After removal of the contaminated portion of the implant, surface treatment of the surgical site with saline irrigation and 24% EDTA is important to remove the smear layer. The antimicrobial effect of these chemotherapeutic agents also allows for improved cell proliferation.[24] Decontaminated surfaces are reported to have higher wettability and therefore provide higher osteoblast proliferation and differentiation on the surfaces.[25] As result, improved reosseointegration of the previous infected region can be achieved.[25]

Conclusions

Implant apicoectomy followed by bone curettage, saline irrigation, and application of EDTA-based conditioner can be a successful treatment option when retrograde peri-implantitis is located in a region with anatomical limitations for chemical/mechanical bacterial elimination.

Before choosing this technique, the professional must take into consideration the location of the healthy implant area and the expected length of the implant after apicoectomy.

Abbreviation

EDTA: ethylenediaminetetraacetic acid
SEM: scanning electron microscopy

Note

The authors declare no conflict of interest.

References

1. Waasdorp JA, Evian CI, Mandracchia M. Immediate placement of implants into infected sites: a systematic review of the literature. *J Periodontol*. 2010;81:801–808.
2. Crespi R, Cappare P, Gherlone E. Immediate loading of dental implants placed in periodontally infected and non-infected sites: a 4-year follow-up clinical study. *J Periodontol*. 2010;81:1140–1146.
3. Del Fabbro M, Boggian C, Taschieri S. Immediate implant placement into fresh extraction sites with chronic periapical pathologic features combined with plasma rich in growth factors: preliminary results of single-cohort study. *J Oral Maxillofac Surg*. 2009;67:2476–2484.
4. Fugazzotto P. A retrospective analysis of immediately placed implants in 418 sites exhibiting periapical pathology: results and clinical considerations. *Int J Oral Maxillofac Implants*. 2012;27:194–202.
5. Fugazzoto PA. A retrospective analysis of implants immediately placed in sites with and without periapical pathology in sixty-four patients. *J Periodontol*. 2012;83:182–186.
6. Flanagan D. Implant placement in failed endodontic sites: a review. *J Oral Implantol*. 2016;42:224–230.
7. Ramanauskaite A, Joudzbalys G, Tözüm TF. Apical/retrograde periimplantitis/implant periapical lesion: etiology, risk factors and treatment options: a systematic review. *Implant Dent*. 2016;25:684–697.
8. Sarmast ND, Wang HH, Soldatos NK, et al. A novel treatment decision tree and literature review of retrograde peri-implantitis. *J Periodontol*. 2016;87:1458–1467.
9. Quirynen M, Gijbels F, Jacobs R. An infected jawbone site compromising successful osseointegration. *Periodontol 2000*. 2003;33:129–144
10. El Askary AS, Meffert RM, Griffin T. Why do dental implants fail? Part I. *Implant Dent*. 1999;8:173–185.
11. Kadkhodazadeh M, Amida R. A new classification for the relationship between periodontal, periapical, and periimplant complications. *Iran Endod J*. 2013;8:103–108.
12. McAllister BS, Masters D, Meffert RM. Treatment of implants demonstrating periapical radiolucencies. *Pract Periodontics Aesthet Dent*. 1992;4:37–41.
13. Piattelli A, Scarano A, Balleri P, Favero GA. Clinical and histologic evaluation of an active "implant periapical lesion": a case report. *Int J Oral Maxillofac Implants*. 1998;13:713–716.
14. Jalbout ZN, Tarnow DP. The implant periapical lesion: Four case reports end review of the literature. *Pract Periodontics Aesthet Dent*. 2001;13:107–112.
15. Balshi SF, Wolfinger GJ, Balshi TJ. A retrospective evaluation of a treatment protocol for dental implant periapical lesions: long-term results of 39 implant apicoectomies. *Int J Oral Maxillofac Implants*. 2007;22:267–272.
16. Penarrocha-Diago M, Mestre-Ferrin L, Penarrocha-Oltra D, Canullo L, Piatelli A. Inflammatory periapical lesion prior to osseointegration: a case series study. *Int J Oral Maxillofac Implants*. 2013;28:158–162.
17. Gomes BP, Pinheiro ET, Gade-Neto CR, et al. Microbiological examination of infected dental root canals. *Oral Microbiol Immunol*. 2004;19:71–76.
18. Ricucci D, Siqueira JF Jr, Bate AL, Pitt Ford TR. Histologic investigation of root canal-treated teeth with apical periodontitis: a retrospective study from twenty-four patients. *J Endod*. 2009;35:493–502.
19. Al-Ahmad A, Ameen H, Pelz K, et al. Antibiotic resistance and capacity for biofilm formation of different bacteria isolated from endodontic infections associated with root-filled teeth. *J Endod*. 2014;40:223–230.
20. Mezzomo LA, Miller R, Triches D, Alonso F, Shinkai RS. Meta-analysis of single crowns supported by short (<10 mm) implants in the posterior region. *J Clin Periodontol*. 2014;41:191–213.

21. Edher F, Nguyen CT. Short dental implants: a scoping review of the literature for patients with head and neck cancer. *J Prosthet Dent*. 2017;pii: S0022-3913(17)30426-2.

22. Eriksson R, Albrektsson T. Temperature threshold levels for heat induced bone tissue injury: a vital-microscopic study in the rabbit. *J Prosthet Dent*. 1983;50:101–107.

23. Eriksson RA, Albrektsson T. The effect of heat on bone regeneration: an experimental study in rabbits using the bone growth chamber. *J Oral Maxillofac Surg*. 1984;42:705–711.

24. Kotsakis GA, Lan C, Barbosa J, Lill K, Chen R, Rudney J, Aparicio C. Antimicrobial agents used in the treatment of peri-implantitis alter the physicochemistry and cytocompatibility of titanium surfaces. *J Periodontol*. 2016;87:809–819.

25. Gittens RA, Olivares-Navarrete R, Cheng A, et al. The roles of titanium surface micro/nanotopography and wettability on the differential response of human osteoblast lineage cells. *Acta Biomater*. 2013;9:6268–6277.
