# Supplementary material for: A novel histopathological classification of implant periapical lesion: A systematic review and treatment decision tree
Source: PLoS One. 2022 Dec 22;17(12):e0277387. doi: 10.1371/journal.pone.0277387 (PMC9778521; doi:10.1371/journal.pone.0277387)
Supplement: S1 File — (ZIP) [file pone.0277387.s001.zip › support files/Included study/Mccracken2012.pdf]

A Residual Granuloma in Association With a Dental Implant

Michael S. McCracken, DDS, PhD,* Ramakiran V. Chavali, BDS, MS,† Nasser Said Al-Naief, DDS,‡ and Paul D. Eleazer, DDS, MS§

At times, dental implants are placed into sites with a history of periapical pathology. Sometimes the infection is active, and other times the tooth may have been extracted years before implant placement. In either case, the possibility exists for long-term residual cysts or infections that can negatively impact the prognosis of the implant. In this case report, an implant is placed into a healed mandibular ridge several months after extraction of the tooth. A radiolucency was noted on routine radiographic examination 2 years later. Surgical inspection and histology revealed a periapical granuloma with acute and chronic inflammatory cells. After surgical curettage of the site, the patient healed without complication. Implants may develop apical pathology as a result of a pre-existing long-term residual infection. (Implant Dent 2012;21:87–90)

Key Words: implant, granuloma, infection, radiolucency

Active infection in close proximity to dental implants is a concern for clinicians. However, evidence suggests that implants can integrate and function next to pulpally infected natural teeth. Shabahang et al[1] placed implants adjacent to intentionally infected teeth in dogs and found favorable healing. Steiner[2] noted periapical healing of a pulpally necrotic tooth without affecting an implant in close proximity. In a group of 20 implants with endodontically treated adjacent natural teeth, Laird et al[3] found a 95% survival of implants.

When the site itself is infected, some clinicians find that immediate implant placement into an infected socket gives equivalent results.[4,5] Generally however, most studies recommend debridement of the site before immediate implant placement.[6–9] Some have proposed that antibiotics aid in success of immediate implant placement or bone grafting before implant placement.[10–12] In a meta-analysis of seven randomized control trials, Esposito et al[13] concluded that immediate implant placement may lead to a lowering of prognosis but that there is insufficient statistically significant evidence to suggest that immediate placement in a fresh extraction site is contraindicated. Crespi et al[14] report 100% success 24 months for implants placed immediately after extraction. Waasdorp et al[15] reported in their systematic review that infected sites did hinder success. Casap et al[7] monitored implants placed into a variety of infected sites, including periapical infections, with satisfactory implant longevity. Also, Villa and Rangert[16] observed 33 implants replacing teeth with endodontic or periodontal infections and noted a 97.4% survival rate at 1 year.

However, some implants fail and we do not always understand the reason for these failures. It is known that endodontic therapy, and even extraction of an infected tooth, does not always eradicate an apical infection. In cases of chronic apical periodontitis, the bacteria form a biofilm which is typically mixed in composition.[17–19] These bacteria are frequently obligate anaerobes and facultative anaerobes,[20] and may be resistant to drug therapy.[21,22] The infection may sometimes be fungal, such as the stubborn actinomycete pathogen.[23]

Despite careful endodontic therapy and endodontic retreatment, some infections are intractable and result in extraction of the tooth. These infections may persist in the alveolus, only to be aggravated by subsequent osteotomy preparation and implant placement. It is possible that the infection then recurs, or becomes active, in the region of the apex of the dental implant. In a pivotal clinical study addressing this topic, Nelson and Thomas[24] monitored the flora found in the alveolus when placing dental implants in healed alveolar ridges. Teeth were extracted and the sites were allowed to heal. Even after healing for 3 months, 21% of osteotomies prepared for implant placement revealed a positive culture. The authors speculated that these residual bacteria may contribute to early implant failure. We report here on a case of an implant

*Professor, Department of General Dental Sciences, University of Alabama at Birmingham School of Dentistry, Birmingham, AL.
†Assistant Professor, Department of Prosthodontics, University of Alabama at Birmingham School of Dentistry, Birmingham, AL.
‡Associate Professor, Department of Pathology and Medicine, University of Pacific in San Francisco, San Francisco, CA.
§Professor, Department of Endodontics, University of Alabama at Birmingham School of Dentistry, Birmingham, AL.

Reprint requests and correspondence to: Paul D. Eleazer, DDS, MS, 1530 3rd Avenue South, SDB 417, UAB School of Dentistry, Birmingham, AL 35294, Phone: 205.935.5067, Fax: 205.975.9197, E-mail: eleazer@uab.edu

ISSN 1056-6163/12/02102-087
Implant Dentistry
Volume 21 • Number 2
Copyright © 2012 by Lippincott Williams & Wilkins
DOI: 10.1097/ID.0b013e31824c2b52

Copyright © Lippincott Williams & Wilkins. Unauthorized reproduction of this article is prohibited.

placed into a site with a history of a persistent periradicular lesion. The implant was placed 5 months after extraction of the tooth. Histological examination of the adjacent tooth revealed normal pulpal histology, whereas the tissue retrieved from the apex of the implant revealed a histological presentation consistent with a periapical granuloma.

CLINICAL REPORT

A 61-year-old woman presented to the UAB Dental School for comprehensive care. The patient was referred to graduate prosthodontics for treatment. She was missing several teeth, and exhibited deep pockets in several sites. She had two fixed partial dentures and a history of routine operative dentistry with sporadic dental cleanings. She had not been to the dentist in 3 years.

The patient was diagnosed with active primary and recurrent caries, defective restorations, missing teeth, and localized severe chronic periodontitis. Also, tooth number 25 was fractured, and teeth 24 to 26 had apical radiolucencies consistent with perio-endo abscesses (Fig. 1). These teeth were sensitive to percussion, and purulence was noted in the area.

Teeth numbers 23 to 26 were extracted, and an interim acrylic partial denture was placed. A graft was not used due to the extent of the localized infection. In addition to her other treatment needs, the patient received a sinus graft, and implants in the areas of teeth numbers 2, 3, 4, 5, 12, 19, 23, 26, and 30. Implants 23 and 26 were placed approximately 5 months after extraction (BioHorizons Inc., Birmingham, AL). The implants used were 3.0 mm in diameter and 15 mm in length. The residual ridge in the area at the time of surgery was intact, with no signs of infection. It was completely healed from the extractions, with normal soft and hard tissue contours. Both implants demonstrated good primary stability, in excess of 40 N · cm. The implants were immediately loaded with an acrylic fixed partial denture.

The patient healed without complication from each of her implant surgeries, and all implants were clinically integrated. Implants and teeth were restored in a satisfactory manner by a resident in the graduate prosthodontics program. Implants 23 and 26 were restored with a 4-unit metal-ceramic fixed partial denture. The patient was satisfied with her dental treatment and function, and was placed on a 6-month maintenance program.

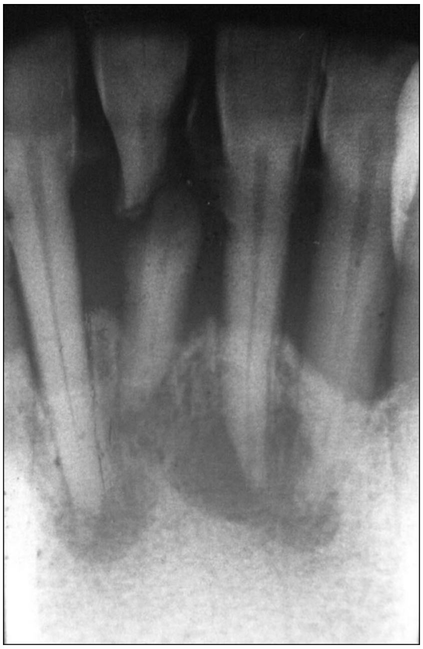

Fig. 1. Initial presentation of patient. Apical pathology was noted in the mandibular incisor area and a fracture of tooth 25.

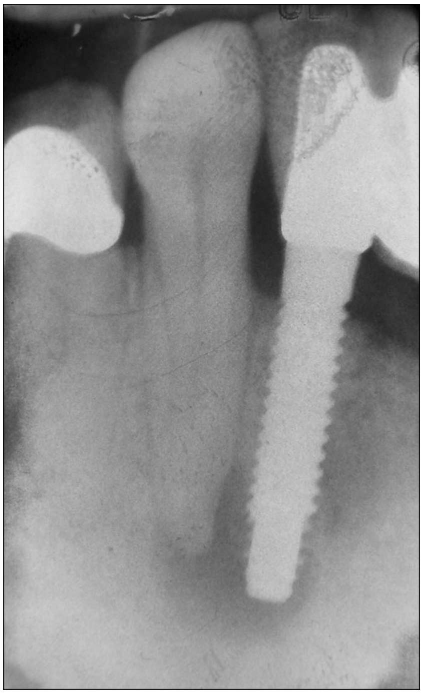

Fig. 2. Routine radiographic examination revealed a radiolucency associated with the apex of implant 26. Both the implant and tooth 27 responded normally to clinical testing.

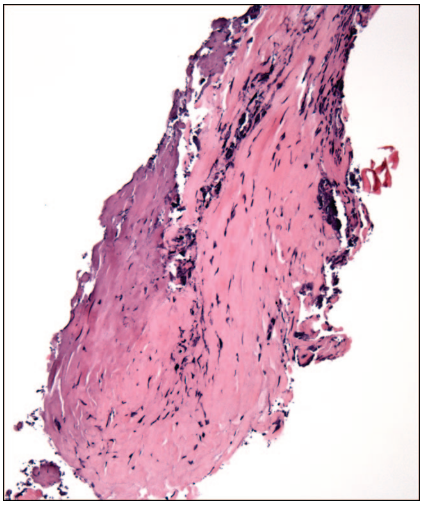

Fig. 3. Histological evaluation of the pulp from tooth 27 reveals vital pulp tissue (×40).

Approximately 2 years after implant restoration, routine radiographic examination revealed a 6-mm circumscribed radiolucency at the apex of implant 26 (Fig. 2). The radiolucency approached the apex of tooth 27. Both the implant and the tooth were asymptomatic, with normal response to percussion and cold tests. A clinical decision was made to surgically explore the lesion. Before surgery, elective endodontics was performed on tooth 27. A pulp biopsy from tooth 27 confirmed vital pulp tissue (Fig. 3).

One month after the endodontic procedure, the patient presented for surgical curettage of the lesion at the apex of implant 26. An incision was made at the mucogingival junction in the region of the premolars and was extended to the midline; a vertical releasing incision was placed near the midline. A full thickness mucoperiosteal flap was elevated. After elevation, a 4-mm fenestration was noted in the apical region of implant 26 (Fig. 4). This area was explored and found to contain soft tissue. The soft tissue was removed with a surgical curette, being careful not to aggressively scratch the surface of the implant. The soft tissue lesion was approximately 8 mm in di-

Copyright © Lippincott Williams & Wilkins. Unauthorized reproduction of this article is prohibited.

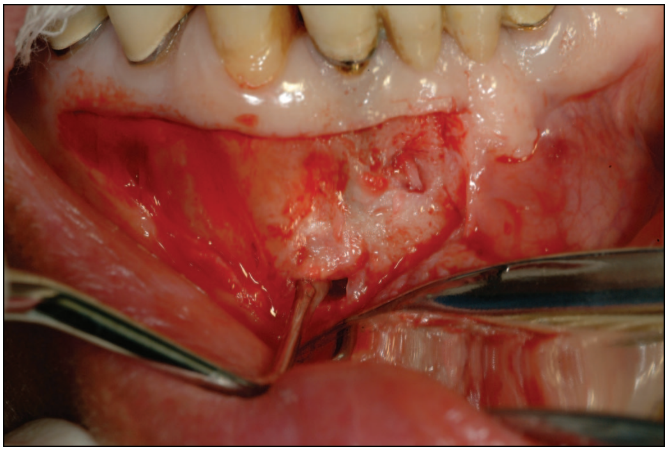

Fig. 4. Presentation of lesion at time of exploratory surgery.

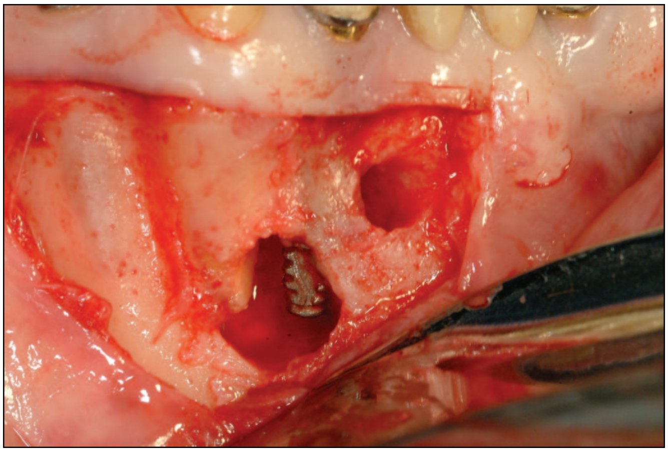

Fig. 5. After removal of soft tissue, a large bony defect remained, encompassing both the apex of the implant, the apex of tooth 27, and an additional thru-and-thru defect opening through the buccal plate in the gingival direction.

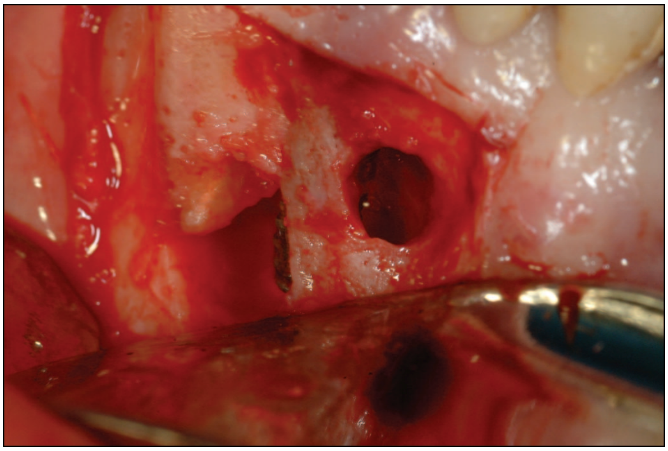

Fig. 6. Lateral view of the defect gives an appreciation for the dimensions of the lesion.

ameter and 8-mm deep. It included the apex of both the implant and the canine. The defect extended superiorly toward the midline, where it perforated the buccal plate in a second location (Fig. 5). The full extent of the defect can be appreciated from the lateral view (Fig. 6). No graft material was placed, which is typical for surgical endodontic procedures, and the wound was closed with absorbable sutures. The patient healed without complications.

The soft tissue excavated from the area was sent to a pathologist for evaluation. The biopsy was read as a periapical granuloma with generalized chronic and acute inflammation, with associated vital reactive bony spicules. Clinical and radiographic examination 8 months after surgery showed good healing and new bone formation (Fig. 7).

Discussion

This case documents a periapical granuloma associated with the apex of a dental implant. There are at least three possibilities to explain the bacterial etiology of this lesion. First, the infection could originate from an adjacent tooth. This hypothesis can be ruled out as pulp tissue was examined histologically and was found to be vital, without signs of infection. Second, bacteria could have been introduced into the osteotomy at the time of implant placement. Although this is possible, one would suspect that this infection would be generalized around the implant, causing the implant to fail, or that symptoms would be noted in the weeks after surgery. The third, and most likely possibility is that the bacteria responsible for the apical infection originated with the natural teeth, and persisted in the granulomatous tissue. This theory is supported by the findings of Nelson and Thomas,[24] mentioned earlier, who showed that viable bacteria could be cultured from healed alveolar ridges. Iwu et al[25] demonstrated persistence of bacteria in granulomatous associated with endodontically treated teeth at surgery.

Clearly, if left untreated, this apical lesion could cause failure of the implant. This finding encourages the use of routine radiographic examination to view not only the crest of the ridge and crestal bone architecture, but also the apex of the implant. This may be especially important in sites where the natural tooth was removed due to apical pathology, or where extensive

Copyright © Lippincott Williams & Wilkins. Unauthorized reproduction of this article is prohibited.

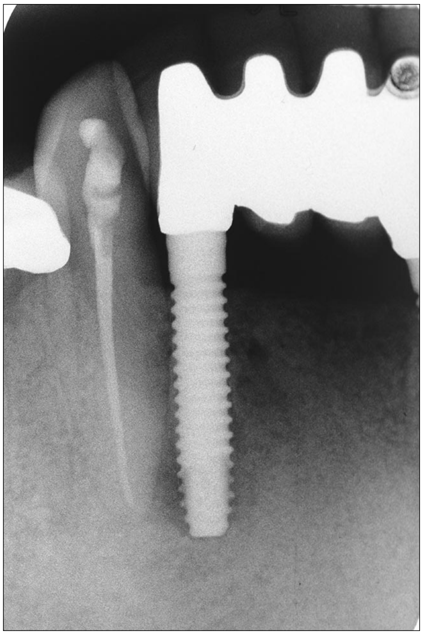

Fig. 7. Eight months after surgery, the radiolucency shows improvement, with evidence of bone fill in the region.

apical infection was present. Also, it seems prudent to aggressively curette extraction sockets after tooth extraction to remove as much of the granulomatous tissue as possible to aid healing.

For this patient, surgical intervention was necessary to eliminate the defect and promote bone regeneration. Although it is common to not place graft material following endodontic procedures such as an apicoectomy, it would be reasonable to graft this defect with a mineralized allograft or other material of choice to enhance healing with a scaffold. After more than 2 years, the patients' apical radiolucency was much smaller although persistent. It is the opinion of the authors that this represents a collagen scar at the apex of the implant. It will be monitored for any possible change.

CONCLUSIONS

This patient developed a granuloma at the apex of a dental implant. The evidence suggests that the bacterial origin of this lesion may have been from the previously extracted natural tooth, even though the alveolar ridge had healed for 5 months after the extraction. Clinicians should be aware of this possible persistence of intrabony infections and associated soft tissue lesions associated with dental implants.

DISCLOSURE

Dr. Michael McCracken is a speaker for BioHorizons, Inc., whose product is mentioned in this article, whereas the other coauthors claim to have no financial interest in any of the products or companies that have been stated within the article.

REFERENCES

1. Shabahang S, Bohsali K, Boyne PJ, et al. Effect of teeth with periradicular lesions on adjacent dental implants. *Oral Surg Oral Med Oral Pathol Oral Radiol Endod*. 2003;96:321–326.

2. Steiner DR. The resolution of a periradicular lesion involving an implant. *J Endod*. 2008;34:330–335.

3. Laird BS, Hermsen MS, Gound TG, et al. Incidence of endodontic implantitis and implant endodontitis occurring with single-tooth implants: A retrospective study. *J Endod*. 2008;34:1316–1324.

4. Del Fabbro M, Boggian C, Taschiere S. Immediate implant placement in to fresh extraction sites with chronic periapical pathologic features combined with plasma rich in growth factors: Preliminary results of single-cohort study. *J Oral Maxillofac Surg*. 2009;67:2476–2484.

5. Chang SW, Shin SY, Hong JR, et al. Immediate implant placement into infected and non infected extraction sockets: A pilot study. *Oral Surg Oral Med Oral Pathol Oral Radiol Endod*. 2009;107:197–205.

6. Novaes AB Jr, Novaes AB. Immediate implants placed into infected sites: A clinical report. *Int J Oral Maxillofac Implants*. 1995;10:609–613.

7. Casap N, Zeltser C, Wexler A, et al. Immediate placement of dental implants into debrided infected dentoalveolar sockets. *J Oral Maxillofac Surg*. 2007;65:384–392.

8. Naves Mde M, Horbylon BZ, Gomes CF, et al. Immediate implants placed into infected sockets: A case report with 3-year follow-up. *Braz Dent J*. 2009;20:254–258.

9. Truninger TC, Philipp AO, Siegenthaler DW, et al. A prospective, controlled clinical trial evaluating the clinical and radiological outcome after 3 years of immediately placed implants in sockets exhibiting periapical pathology. *Clin Oral Implants Res*. 2011;22:20–27.

10. Lindeboom JA, Frenken JW, Tuk JG, et al. A randomized prospective controlled trial of antibiotic prophylaxis in intraoral bone-grafting procedures: Preoperative single-dose penicillin versus preoperative single-dose clindamycin. *Int J Oral Maxillofac Surg*. 2006;35:433–439.

11. Esposito M, Grusovin MF, Talati M, et al. Interventions for replacing missing teeth: Antibiotics at dental implant placement to prevent complications. *Cochrane Database Syst Rev*. 2008;(3):CD004152.

12. Abu-Ta-a M, Quirynen M, Teughels W, et al. Asepsis during periodontal surgery involving oral implants and the usefulness of peri-operative antibiotics: A prospective, randomized controlled clinical trial. *J Clin Periodontol*. 2008;35:58–63.

13. Esposito M, Grusovin MG, Polyzos IP, et al. Interventions for replacing missing teeth: Dental implants in fresh extraction sockets (immediate, immediate-delayed and delayed implants). *Cochrane Database Syst Rev*. 2010;8:CD005968.

14. Crespi R, Cappare P, Gherlone E. Fresh-socket implants in periapical infected sites in humans. *J Periodontol*. 2010;81:378–383.

15. Waasdorp JA, Evian CI, Mandracchia M. Immediate placement of implants into infected sites: A systematic review of the literature. *J Perodontol*. 2010;81:801–808.

16. Villa R, Rangert B. Immediate and early function of implants placed in extraction sockets of maxillary infected teeth: A pilot study. *J Prosthet Dent*. 2007;97:S96–S108.

17. Noiri Y, Ehara A, Kawahara T, et al. Participation of bacterial biofilms in refractory and chronic periapical periodontitis. *J Endod*. 2002;28:679–683.

18. Sunde PT, Olsen I, Debelian GJ, et al. Microbiota of periapical lesions refractory to endodontic therapy. *J Endod*. 2002;28:304–310.

19. Zhang S, Wang QQ, Zhang CF, et al. Identification of dominant pathogens in periapical lesions associated with persistent apical periodontitis. *Chin J Dent Res*. 2010;13:115–121.

20. Fujii R, Saito Y, Tokura Y, et al. Characterization of bacterial flora in persistent apical periodontitis lesions. *Oral Microbiol Immunol*. 2009;24:502–505.

21. Barnard D, Davies J, Figdor D. Susceptibility of *Actinomyces israelii* to antibiotics, sodium hypochlorite and calcium hydroxide. *Int Endod J*. 1996;29:320–326.

22. Skucaite N, Peciuliene V, Vitkauskiene A, et al. Susceptibility of endodontic pathogens to antibiotics in patients with symptomatic apical periodontitis. *J Endod*. 2010;36:1611–1616.

23. Al-Hezaimi K. Apical actinomycosis: Case report. *J Can Dent Assoc*. 2010; 76:a113

24. Nelson S, Thomas G. Bacterial persistence in dentoalveolar bone following extraction: A microbiological study and implications for dental implant treatment. *Clin Implant Dent Relat Res*. 2010;12:306–314.

25. Iwu C, MacFarlane TW, MacKenzie D, et al. The microbiology of periapical granulomas. *Oral Surg Oral Med Oral Pathol*. 1990;69:50205.

Copyright © Lippincott Williams & Wilkins. Unauthorized reproduction of this article is prohibited.
