# Supplementary material for: A novel histopathological classification of implant periapical lesion: A systematic review and treatment decision tree
Source: PLoS One. 2022 Dec 22;17(12):e0277387. doi: 10.1371/journal.pone.0277387 (PMC9778521; doi:10.1371/journal.pone.0277387)
Supplement: S1 File — (ZIP) [file pone.0277387.s001.zip › support files/Included study/Mccrea2014.pdf]

Nasopalatine Duct Cyst, a Delayed Complication to Successful Dental Implant Placement: Diagnosis and Surgical Management

Shane Joseph McCrea, MMedSci, MSc

The dental literature reports frequently on both the success and survival of dental implants, whereby the focus remains on the biological response of hard and soft tissue to the implants. The predication and anticipation of adverse implant events can then lead to the preemption of implant loss. However, biological situations can arise that are outside the control of the clinician. The author reports a case history of the late manifestation of a nasopalatine duct cyst in close proximity to a dental implant and its subsequent surgical management.

Key Words: implantology, nasopalatine duct cyst, symphyseal graft

INTRODUCTION

The use of dental implants for oral rehabilitation is now an accepted form of treatment. The dental literature reports frequently on both the success and survival of dental implants, whereby the focus remains on the biological response of hard and soft tissue to the implants: peri-implantitis, periapical implantitis, marginal bone level maintenance,[1] increased pocket-probing depths, mucositis, suppuration, and attachment loss.[2] The predication and anticipation of adverse implant events can then lead to the preemption of those events. However, biological situations can arise that are outside the control of the clinician. The author reports a case history of the late manifestation of a nasopalatine duct cyst (NPDC) in close proximity to a dental implant and its subsequent surgical management.

The World Health Organization classifies the NPDC as a developmental, epithelial, nonodontogenic cystic lesion of the maxilla. The nasopalatine duct cyst is the most common nonodontogenic cyst of the oral cavity,[3] occurring in about 1% of the population.[4] Radiographically, it can be mistaken for an apical lesion of endodontic origin. The cyst is believed to arise from the epithelial remnants of the nasopalatine duct, an embryological structure connecting the oral and nasal cavities in the area of the incisive canal.[5] The cyst is unique, occurring in a single location, the midline of the anterior maxilla, developing at almost any age. However, the most common occurrence is in the fourth and sixth decades,[4] with most studies showing a slight male predominance.[6] The cysts are usually asymptomatic, being an incidental radiographic discovery. The most common reported clinical symptom is swelling of the anterior part of the palate in the vicinity of the incisal papilla and sometimes intermittent pain over a long period of time.[7] If the cyst has caused resorption of the overlying bone, it is fluctuant and appears blue.[8] Mucoid discharge may occur, causing patients to complain of a salty or foul taste. The more advanced cases can cause pain and itching.[7,8]

Radiographic findings

Two-dimensional radiographs usually demonstrate a well-circumscribed round or oval radiolucency with a sclerotic border at or near the midline of the anterior maxilla, between and apical to the central

British Society of Oral Implantology Southport, Merseyside, UK.
* *Corresponding author,* e-mail: shanemccrea@aol.com
DOI: 10.1563/AAID-JOI-D-12-00011

incisor teeth. Sometimes the radiolucency is heart-shaped as a result of superimposition of the nasal spine or because of resistance of the adjacent roots. Root resorption is rare. The radiographic diameter of the cyst can range from a small lesion greater than 6 mm to lesions as large as 6 cm. The average diameter is 1.5 cm.[4]

Differential diagnosis must include a radicular cyst and granuloma since they may be similar to asymmetrically placed nasopalatine duct cysts. Other conditions will include an enlarged nasopalatine duct, central giant cell granuloma, osteitis fistulizing in the palatine direction, or a bucconasal or buccosinusoidal communication. The tentative diagnosis will be based on the clinical history and findings and the radiological examination that may include periapical radiographs, occlusal radiographs, and tomography (linear and cone-beam computerized tomography).

Treatment protocols

The clinical presentation together with the aid of radiographic modalities of simple plain films and possibly cone-beam computerized tomography will determine the extent of the involvement of neighboring anatomic structures and, therefore, the surgical intervention: marsupialization of smaller swellings may be sufficient; larger swellings will sometimes require marsupialization followed by cystectomy in all cases[9] and autogenous bone grafting. Suter et al[10] have reported on the use of the iliac crest as a donor site.

If not diagnosed early, the NPDC can expand through the palatal and/or buccal walls and into the nasal cavity. The more expansive the cyst, the more complicated the surgical therapy will be.

Case Report

A 62-year-old white man presented at a private practice dedicated to implantology, restorative periodontology, and advanced restorative techniques. The patient's reason for attendance was the rehabilitation of 2 asymptomatic fractured upper right premolar teeth. It was noted that the patient had been rehabilitated 3 years ago with a successful implant replacement of the upper left central incisor (tooth 21, FDI notation). The clinical examination included that of the implant in position 21: a "grey-blue" soft/fluctuant translucent mucosal swelling was found on the palatal aspect of the implant, measuring approximately 1 cm in diameter. The anterior palatal mucosa was fully depressible with no bony resistance. A fluctuant buccal swelling was also present at tooth 11. Radiographic examination (periapical radiograph, Figure 1a, and cross-sectional linear tomogram, Figure 2a) showed 2 separate radiolucent areas at the central incisors, the larger of which had a round, well-demarcated cortex and a diameter estimated at > 1.5 cm, using the dimensions of the dental implant as the magnification factor. The second distinct radiolucency was at the apex of tooth 11 and superimposed on the larger radiolucency: tooth 11 had been root-treated and restored with a cast post-crown. Neither tooth 11 nor implant 21 displayed any mobility.

The patient's medical history did not reveal any pathological condition. On the basis of the clinical and radiological examination, a tentative diagnosis of nasopalatine duct cyst was made for the larger radiolucency with a separate diagnosis of periapical granuloma at the apex of tooth 11. The patient was advised of the extent of the primary lesion and the loss of the anterior bony palate. The necessity of excision of the prime lesion—the nasopalatine duct cyst—was explained as was the necessity for a bone graft; the symphyseal graft was to be utilized to support the palatal soft tissues and protect an underlying particulate xenograft (Bio-Oss, Geistlich Pharma, Wolhusen, Switzerland). Full written consent was obtained.

Surgical procedure

One hour preoperatively, oral medication of 2 g amoxicillin, 800 mg ibuprofen, and 2 mg dexamethasone was administered. Additionally, mouthrinse of chlorhexidine gluconate 2% was given 20 minutes prior to surgery. Surgery was carried out under intravenous sedation of midazolam and local anesthesia (articaine 4% and 100 000 adrenaline). A palatal mucoperiosteal flap was raised with incisions made in the palatal sulcus of all teeth anterior to the first premolars, thus exposing the cyst (Figure 3a). The friable cyst was dissected from the overlying palatal mucosa and found to be attached at the incisal foramen where its dissection was completed. The cyst was preserved for histologic examination. The palatal aspect of the implant in position 21 was fully exposed (Figure 3b), together with the palatal

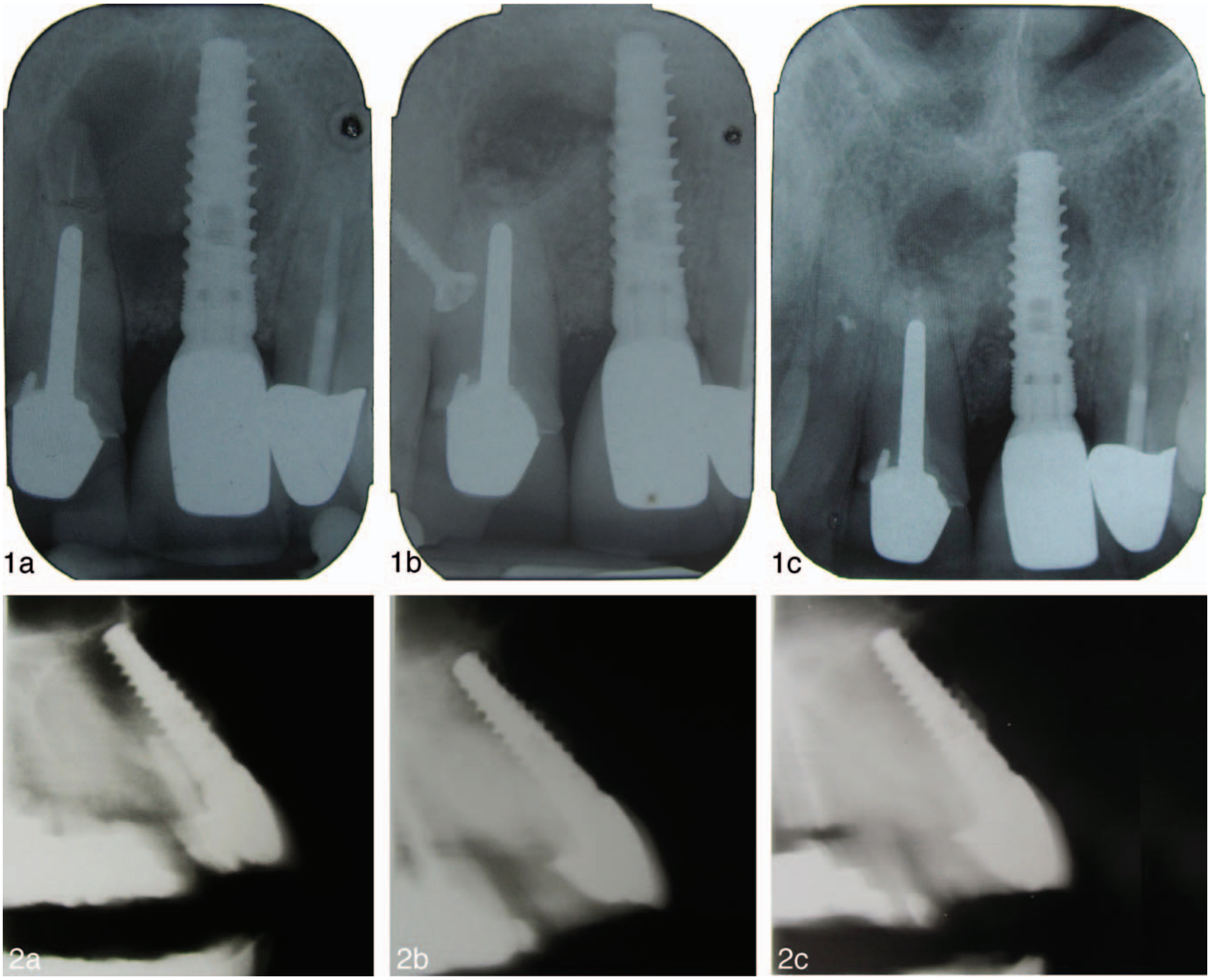

FIGURES 1 AND 2. FIGURE 1. (a) Initial diagnostic periapical radiograph. (b) Periapical radiograph taken immediately after surgery. (c) Periapical radiograph taken 24 months postoperatively. **FIGURE 2.** (a) Initial diagnostic linear tomogram. (b) Linear tomogram taken immediately after surgery. (c) Linear tomogram taken 24 months postoperatively.

aspect of tooth 21. A semilunar incision was made buccal to positions 11 and 21, thus exposing the buccal bony fenestration (Figure 3c). The cyst attached to the root apex of tooth 11 was removed, an apicectomy performed, and a retrograde filling of glass-ionomer made. This cyst was also sent for histologic examination. The dimensions of the cystic cavity were determined and mapped onto the symphyseal region (Figure 3d). The symphyseal cortex was then harvested together with some underlying medullary bone (Figure 3e and f). The harvested symphyseal plate was placed at the recipient site, and the fixation hole sites were determined so that the screw attachments would by interdentally between the lateral incisors and the canines (Figure 3g). Bone xenograft granules (0.25–1 mm) were mixed with the harvested medullary bone scrapings and packed into the palatal voids, thus covering the exposed root surface of tooth 11 and the implant surface 21. The harvested symphyseal cortex was then placed into the palatal bony void and fixed laterally using 11-mm self-tapping screws (Figure 3h).

The palatal mucoperiosteal flap was replaced and secured with 4.0 Vicryl resorbable continuous interdental sutures. The buccal void at tooth 11 was further filled with xenograft and covered with a resorbable membrane (Bio-Gide, Geistlich Pharma). The buccal flap was closed with the same suture material (Figure 3i). The symphyseal donor site was also closed with the same suture material. A postoperative periapical radiograph together with

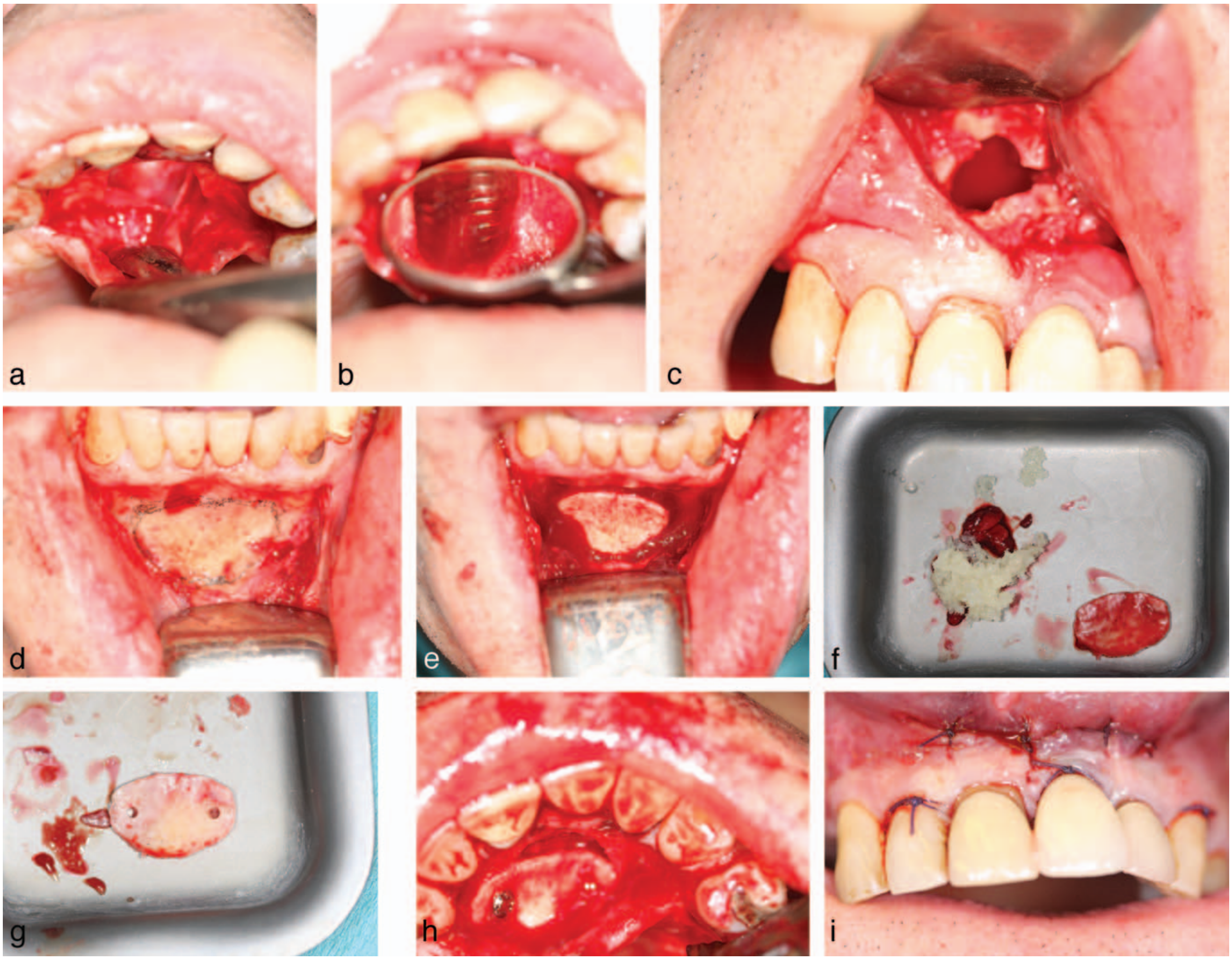

Figure 3. (a) Intrasulcular incisions were made to allow the complete raising of the palatal mucoperiosteal flap, taking care not to penetrate the midline underlying friable cystic wall. (b) The implant palatal surface appears completely devoid of osseous tissue. (c) The buccal flap has been raised over tooth 11 to relieve an apical bony dehiscence and underlying cavity. (d) The dimensions of the required symphyseal graft have been mapped out onto the bony cortex. (e) The symphyseal cortex has been completely penetrated using a small round bur (no. 1) and elevated slightly from the underlying trabecular bone. (f) The harvested cortical plate. (g) The screw holes for fixation of the graft have been made. (h) The symphyseal graft is secured in place with 11-mm screws, thus covering the underlying Bio-Oss granules. (i) The palatal and buccal flaps have been replaced and secured with 4.0 Vicryl sutures.

a linear tomogram were taken (Figures 1b and 2b). Postoperatively, the patient was prescribed amoxicillin 500 mg, 3 times daily for 7 days and ibuprofen as needed.

Postoperatively

Sutures were removed at 10 days. The fixation screws were removed after 6 months using simple incisions. At 6 months, 12 months, and 24 months postoperatively, clinical and radiographic examinations were carried out; all showed satisfactory healing (Figures 1c, 2c, and 4a and b). The stability of both tooth 11 and implant 21 were tested for mobility—neither was mobile.

Histology

The large specimen measured approximately 1.5 cm in diameter. The fibrous wall was lined by thin, stratified squamous epithelium and partly by pseudostratified columnar epithelium and cuboidal epithelium. A few nerve bundles and blood vessels were also present in the wall. All of these findings were suggestive of a nasopalatine cyst.

The small specimen displayed inflamed granula-

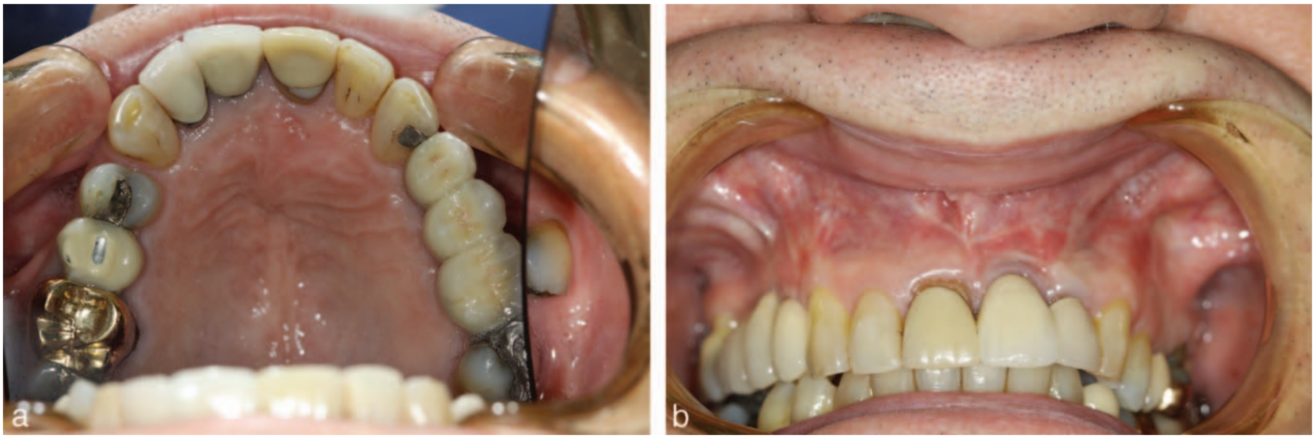

Figure 4. (a) Palatal tissues 24 months postoperatively. (b) Buccal operative site 24 months postoperatively.

tion tissue with a few histiocytic multinucleated giant cells in it. It also displayed inflamed scar tissue. These findings were indicative of periapical granuloma.

Discussion

Regardless of the perceived success of the biological events of the osseointegration of dental implants, those same implants will be affected by adverse events in adjacent anatomic sites. Crestal bone peri-implantitis remains a focus for many operators, and consequently, various treatments for such a condition have been reported.[11] Equally so, periapical implantitis is also reported, and treatment concepts[12,13] have been tried out with some success. Where large-scale radiolucencies appear in close proximity to dental implants, they will indicate the loss of bone-implant-contact and predict the future loss of the implant. This case study presents a situation of the formally successful osseointegration of a dental implant whose adjacent bony alveolus has been resorbed by an expansive lesion. The surgical requirement was not only to remove the expansive lesion but also to produce an environment that would allow the regeneration of the lost bone-implant-contact.

The nasopalatine duct cyst is a self-contained expansive lesion that is caused to become active by infection within the duct. The bone at the implant surface was not lost as a result of infection, but as a result of resorption from an external source. Therefore, it could be postulated that with the removal of the external force (the NPDC), a bone-implant-contact could be reestablished. The xenograft particles had a twofold function. First, the particles provide the classical scaffold function for osseoconduction through the blood clot and therefore renewed bone regeneration to the implant surface. Second, the presence of the xenograft particulate mass reduces the void left after removal of the cyst, thus reducing the chances of infection. The postoperative 24-month radiographs (Figures 1c and 2c) would suggest a close approximation of the newly formed bone to the implant surface and the continuation of bony remodelling in the former cystic void.

Immobilization is a prerequisite for bone repair and regeneration. The immobilization of the xenograft and autogenous medullary bone-scrapings and blood clot was facilitated by using the symphyseal graft. It has been reported that the iliac crest can be used for such a purpose.[10] However, the symphyseal graft harvesting procedure is a known technique practiced by implantologists and oral-maxillofacial surgeons and dispenses with the complicated procedures of iliac bone harvesting. In view of the results obtained in this case report (Figures 1c, 2c, and 4a), the author would advocate the described technique in cases of palatal bone loss.

The presence of the nasopalatine cyst remains a chance finding due to its largely asymptomatic nature. Its unchecked expansion will result in destruction of the palatal and/or buccal cortical walls and sometimes even the nasal floor. The presence of dental implants in the direction of expansion will certainly endanger those implants. The annual review of dental implants in the anterior

maxilla must be strongly reinforced to allow the early diagnosis of the NPDC and its ease of excision.

Abbreviations

NPDC: nasopalatine duct cyst

References

1. Smith DE, Zarb GA. Criteria for success of osseointegrated endosseous implants. *J Prosthet Dent*. 1989;62:567–572.
2. Esposito M, Thomsen P, Molne J, Gretzer C, Ericson LE, Lekholm U. Immunohistochemistry of soft tissues surrounding late failures of Branemark implants. *Clin Oral Implants Res*. 1997;8:352–366.
3. Shear M, Speight P. Nasopalatine duct (incisive canal). In: *Cysts of the Oral and Maxillofacial Regions*. 4th ed. Hoboken, NJ: Wiley-Blackwell.
4. Francoli JE, Marques NA, Aytes LB, Escoda CG. Nasopalatine duct cyst: report of 22 cases and review of the literature. *Med Oral Patol Oral Cir Bucal*. 2008;13;E438–E443.
5. Neville BW, Damm DD, Allen CM, Bouquot JE. *Oral & Maxillofacial Pathology*. 2nd ed. Philadelphia, Pa: WB Saunders; 2002.
6. Wood NK, Goaz PW. Differential diagnosis of oral and maxillofacial lesions. In: Wood NK, Goaz PW, eds. *Inter-Radicular Radiolucencies*. 5th. ed. Maryland Heights, Mo: Mosby; 1997:303–305.
7. Swanson KS, Kaugars GE, Gunsolley JC. Nasopalatine duct cyst: an analysis of 334 cases. *J Oral Maxillofac Surg*. 1991;49:268–271.
8. Valesquez-Smith MT, Mason C, Coonar H, Bennett J. A nasopalatine cyst in an 8 year old child. *Int J Paediatr Dent*. 1999;9:123–127.
9. Gnanasekhar JD, Walvekar SV, Al-Kandari AM, Al-Duwairi Y. Misdiagnosis and mismanagement of a nasopalatine duct cyst and its corrective therapy. A case report. *Oral Surg Oral Med Oral Pathol Oral Radiol Endod*. 1995;80:465–470.
10. Suter VG, Büttner M, Altermatt HJ, Reichart PA, Bornstein MM. Expansive nasopalatine duct cysts with nasal involvement mimicking apical lesions of endodontic origin: a report of two cases. *J Endod*. 2011;37:1320–1326.
11. Froum S, Yamanaka T, Cho SC, Kelly R, St James S, Elian N. Techniques to remove a failed integrated implant. *Compend Contin Educ Dent*. 2011;32:22–26, 28–30; quiz 31–32.
12. Jalbout ZN, Tarnow DP. The implant periapical lesion: four case reports and review of the literature. *Pract Proced Aesthet Dent*. 2001;13:107–112; quiz 114.
13. Romanos GE, Froum S, Costa-Martins S, Meitner S, Tarnow DP. Implant periapical lesions: etiology and treatment options. *J Oral Implantol*. 2011;37:53–63.
