# Supplementary material for: A novel histopathological classification of implant periapical lesion: A systematic review and treatment decision tree
Source: PLoS One. 2022 Dec 22;17(12):e0277387. doi: 10.1371/journal.pone.0277387 (PMC9778521; doi:10.1371/journal.pone.0277387)
Supplement: S1 File — (ZIP) [file pone.0277387.s001.zip › support files/Included study/Nedir2007.pdf]

Starch-Induced Implant Periapical Lesion: A Case Report

Rabah Nedir, DMD[1]/Mark Bischof, DMD[1]/Ollivier Pujol, PhD[2]/Raymond Houriet, PhD[3]/
Jacky Samson, MD[4]/Tommaso Lombardi, MD, DMD, PD[5]

This paper reports an implant periapical lesion (IPL) with a previously unreported etiology. The presence of an osteolytic area around the apex and around the middle portion of a stable Straumann hollow-screw implant was found on periapical radiographs 3.5 years after implant placement. Case management involved curettage of the soft tissue surrounding the implant apex as well as resection of the nonosseointegrated portion of the implant. Histopathologic examination revealed a connective fibrous tissue containing a dense chronic inflammatory infiltrate with a foreign-body material. Polarized light microscopy and Fourier transform infrared microspectroscopy identified the foreign-body material as starch particles. Etiology of this IPL was thus related to a foreign-body reaction to starch particles. This exogenous contamination probably originated from starch-coated gloves during the surgical procedure. This case report suggests that IPL may successfully be treated by debridement and implant resection instead of implant removal. Peri-implant apical soft tissue should be systematically submitted for histopathologic examination. INT J ORAL MAXILLOFAC IMPLANTS 2007;22:1001–1006

Key words: *apical peri-implantitis, dental implants, granuloma, implant failure, radiolucency, starch*

Implant periapical lesion (IPL) is a rare pathology on which single case reports[1–5] and 2 studies[6,7] have been published. In 1995, Reiser and Nevins[6] observed 10 IPLs among 3,800 examined implants. Recently, Quirynen et al[7] reported 10 IPLs among 539 single implants. IPL was first introduced as a distinct entity by McAllister et al.[8] Synonyms are "apical peri-implantitis" or "retrograde peri-implantitis."[9]

[1]Assistant Professor, Department of Stomatology and Oral Surgery, School of Dental Medicine, University of Geneva, Switzerland; Clinical Director, Swiss Dental Clinics Group, Switzerland.
[2]Researcher, Powder Technology Laboratory, Swiss Federal Institute of Technology, Lausanne, Switzerland.
[3]Senior Researcher, Powder Technology Laboratory, Swiss Federal Institute of Technology, Lausanne, Switzerland.
[4]Professor and Chairman, Department of Stomatology and Oral Surgery, School of Dental Medicine, University of Geneva, Switzerland.
[5]Associate Professor, Laboratory of Oral Histopathology, Department of Stomatology and Oral Surgery, School of Dental Medicine, University of Geneva, Switzerland.

Correspondence to: Dr Rabah Nedir, Swiss Dental Clinics Group, CdR Clinique de Soins Dentaires SA, Rue du Collège 3, 1800 Vevey, Switzerland. Fax: +41 21 922 22 98. E-mail: rabah.nedir@swissdentalclinics.ch

The initial clinical manifestations of IPL are swelling and tenderness in the region of the affected implant, followed by the development of a sinus tract.[10] In addition, radiolucency is observed at the apical portion of the implant, although the coronal portion is still supported by normal bone architecture in contact with a clinically stable implant. Differential diagnosis of IPL includes pre-existing "necrotic bone" infection, infection from a neighboring tooth, or contamination during surgery. The condition appears to originate with pathogens (bacteria).

This paper reports on a case of IPL associated with a foreign-body reaction to starch particles. To the authors' knowledge, this etiology has never been previously reported. The patient was successfully treated by implant apical resection and thorough curettage.

CASE REPORT

A 56-year-old white woman in good general health presented with a painful swelling in the area of the right cheek. Clinical examination revealed an abscess in the apical region of a 14-mm-long Straumann hollow-screw implant (Straumann, Basel, Switzerland) supporting a single crown in the maxillary right sec-

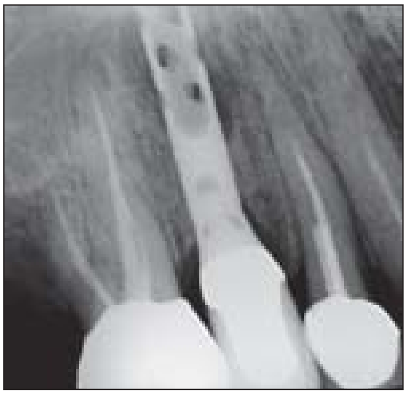

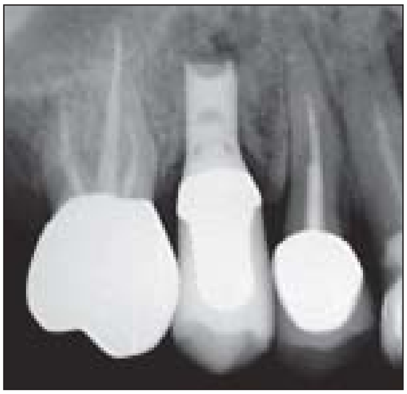

Fig 1 *(left)* Preoperative radiograph revealing the presence of an implant periapical lesion around a hollow-screw perforated implant.

Fig 2 *(right)* Radiograph obtained 2 years after implant resection. Note the nearly complete new bone formation.

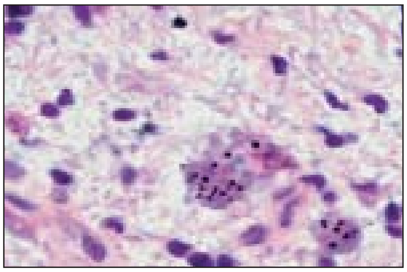

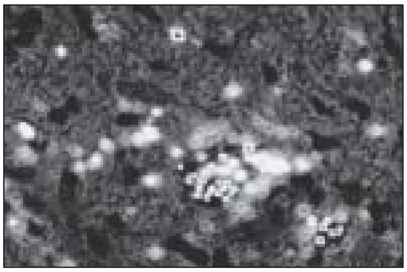

Fig 3 *(left)* Histopathologic examination of the tissue biopsy. Foreign particles were mainly phagocytized by macrophages.

Fig 4 *(right)* Histopathologic examination under polarized light of the biopsy specimen. The foreign particles showed the typical Maltese cross aspect.

ond premolar region. A periapical radiograph showed the presence of an osteolytic area around both the apex and the middle portion of the implant (Fig 1). Probing depth was less than 3 mm, and the cervical peri-implant soft tissues showed no signs of inflammation.

Extraction of the maxillary right second premolar had been carried out because endodontic treatment was failing. After a 5-month postextraction healing period, an implant was placed. This implant had been in place for 3.5 years at the time the patient presented with an IPL.

Systemic antibiotics were prescribed for 12 days (amoxicillin [Amoxi-Mepha]; Mepha Pharma, Aesch, Switzerland; 750 mg 3 times per day). After 1 week, the inflammatory symptoms abated, but a sinus tract with a mild purulent discharge developed in the following days. A mucoperiosteal exploratory flap was raised to determine the possible cause of the infection.

Flap reflection revealed a bone fenestration of the buccal cortical plate with about 5 mm of residual cervical bone. Inflamed tissue surrounded the apical portion of the implant. Upon debridement, yellowish exudate was present, and an oroantral communication was observed. The perforated hollow design of the implant apex did not allow thorough curettage of the region. Thus, it was decided to amputate the nonosseointegrated implant extremity, which would eliminate the implant perforations. Resection was performed with a tapered fissure bur under copious irrigation. The resulting bone cavity communicated with the maxillary sinus but did not involve the adjacent teeth.

There were no postoperative complications, and healing has been uneventful. Recurrence of the sinus tract was not observed over a 2-year follow-up period, and nearly complete new bone formation was radiographically visible around the resected area (Fig 2). The implant is still stable and functional.

Histopathologic examination of the curetted tissue around the implant extremity revealed fibrous connective tissue containing a dense chronic inflammatory infiltrate consisting of lymphocytes, plasma cells, and occasionally, macrophages (Figs 3 and 4). Numerous small round foreign bodies were observed; some had been phagocytized by macrophages. These foreign particles were birefringent under polarized light and showed a typical Maltese cross aspect that might be attributed to starch.

To confirm the presence of starch particles in the inflammatory tissue, physicochemical characterization was carried out by Fourier transform infrared (FTIR) microspectroscopy. The FTIR microspectroscope (Spectrum Spotlight 200 FTIR Microscope System; PerkinElmer, Schwerzenbach, Switzerland) was operated in reflection mode between 500 and 4,000 cm^{-1}. This technique generates infrared signals of the inclusion area. It enables the identification of differences between chemical compounds and the spatial distribution of features of a sample, such as individual contaminants.[11,12] A tissue sample was embedded in paraf-

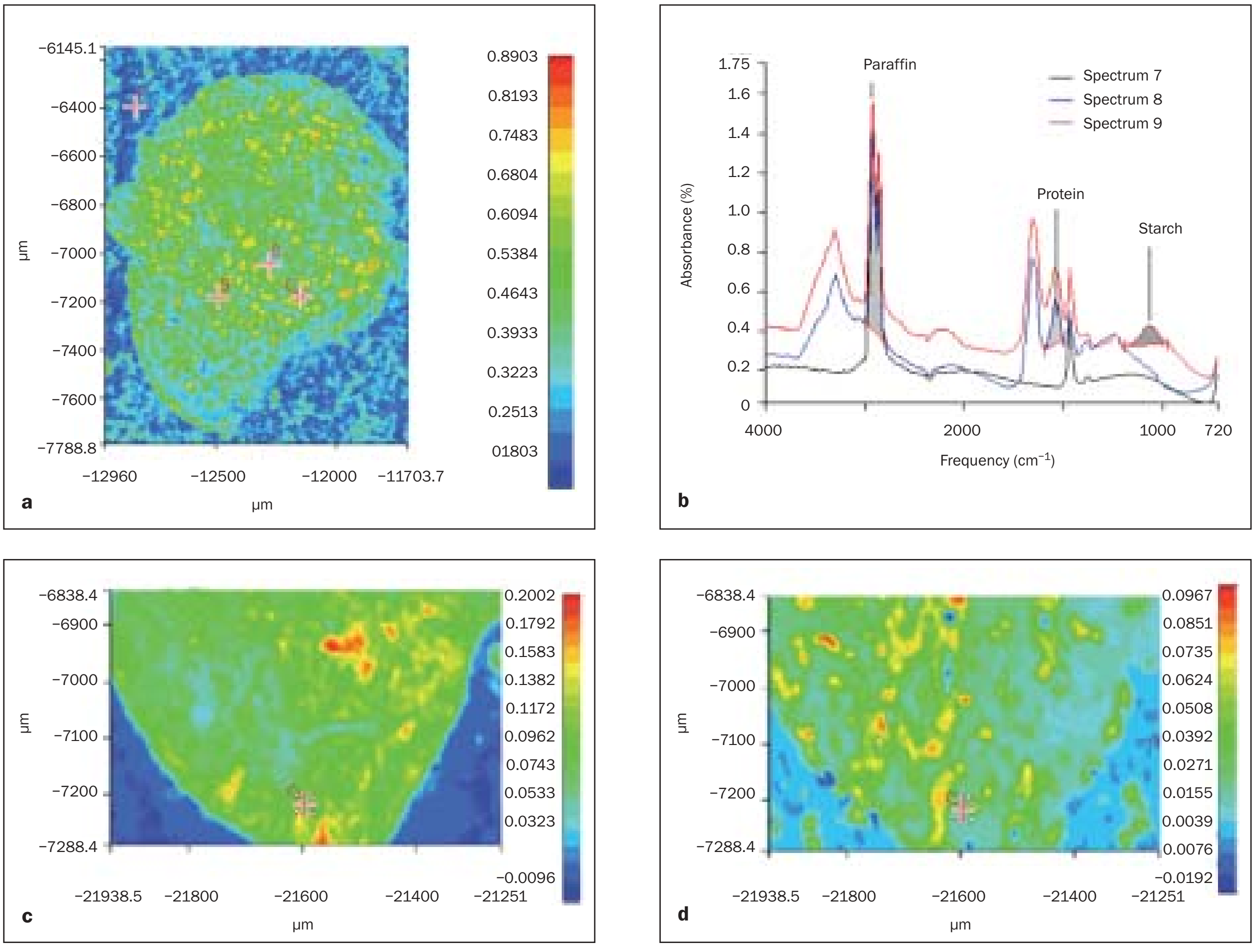

Figs 5a to 5d FTIR analysis of the biopsy specimen. *(a)* FTIR map of the total absorbance of a granuloma cross section. The crosses marked 7, 8, and 9 correspond to 3 different points of analysis. *(b)* FTIR spectra recorded at the points marked 7, 8, and 9 in Fig 5a. *(c)* FTIR map obtained with a typical protein absorption band (1520 cm^{-1}). *(d)* FTIR map obtained with a starch absorption band (1030 cm^{-1}) of the same area.

fin wax, and sections were deposited on a glass microscope slide coated with silver nanoparticles. The measurements were performed at room temperature.

An FTIR map of a 2.06-mm^2 cross section is shown in Fig 5a, built out of the total absorbance of the sample. It shows the specimen and the surrounding paraffin matrix. Inside the tissue, FTIR spectra were recorded at the 3 points marked 7, 8, and 9 (Fig 5a). They are displayed in Fig 5b. Spectrum 7 showed vibration bands of 2850 to 3000 cm^{-1}, 1450 to 1470 cm^{-1}, and 1370 to 1380 cm^{-1}. They are typical of alkane-like paraffin compound absorption. Spectrum 8 exhibited peaks at 1630 cm^{-1} and 1520 cm^{-1}, corresponding respectively to amide I (-C=O) absorption and amide II (-NH-) absorption bands of cellular tissue proteins. Compared with spectrum 8, spectrum 9 identified a new band at around 1030 cm^{-1}. This vibration, identified as a C-O absorption band, is typical of a polysaccharide-like starch.

Fig 5c shows an FTIR map obtained at high magnification with a typical protein absorption band (1520 cm^{-1}) of a sample area. This mapping allowed discrimination between the curetted material of the IPL and the surrounding paraffin. Fig 5d presents a mapping of the spatial absorbance distribution of starch in this area. The starch distribution in the tissue was not homogeneous; starch particles seemed to agglomerate. Fig 5d shows isolated starch particles of about 15 ± 5 μm in diameter.

DISCUSSION

The presence of a delimited endosseous radiolucency may evoke a retrograde apical pathosis. It can also be due to excessive drilling or to an implant placed in a pre-existing intrabony scar lesion. Such radiolucencies would be "noninfected" retrograde

peri-implant radiolucencies and would require only clinical and radiologic follow-up.[10]

To date, little is known about the etiopathogenesis of IPL. It appears to have a multifactorial origin.[13–15] IPL is thought to be related to pre-existing bone infection,[16] residual root particles, or foreign bodies[6] introduced during surgery.[4] Furthermore, overheating,[2,8] excessive tightening, aseptic necrosis of the bone inside the hollow portion of the implant,[3] and infected endodontic lesions from adjacent teeth[4,8,16,17] have all been proposed as etiologic factors. However, these hypotheses are not supported by scientific data. Quirynen et al[7] observed that the incidence of IPL was significantly higher for implants with rough surfaces when compared with machined implants; however, the machined implant surface has been associated with a higher failure rate than the rough implant surface.

In the field of oral and endodontic surgery, it is recommended in clinical practice to histologically analyze every endosseous or periapical lesion to obtain a definitive diagnosis.[18] Final diagnosis of the IPL should also fall under this recommendation. Histologic examination should allow differentiation between inflammation- and non-inflammation–related pathologies (eg, central giant cell granuloma, metastasis).[19,20]

Histologic examination was reported for 5 case reports. In those instances, final histopathologic diagnoses were acute localized osteomyelitis,[1] aseptic bone necrosis,[2–4] and granulation tissue with acute inflammatory cells.[5] This last diagnosis was obtained through a biopsy of a specimen obtained without implant removal; in the other 4 cases, tissue specimens were retrieved after implant removal.

IPL associated with a foreign-body reaction due to starch particles has never been previously reported. Starch is added to rubber gloves as a lubricant, although many unwanted consequences are associated with its use, such as contact irritation and allergic reactions. Natural rubber latex proteins leak out of latex gloves and bind to surgical glove powder, this complex is responsible for latex allergy.[21] For many years, gynecological and thoracic surgeons have been aware of the risks of postoperative granuloma formation due to glove-powder contamination of the surgical wound.[19,22–24] Starch granules are found in surgical wounds proportionally to the number of the surgical team members using powdered gloves.[25] In fact, alternatives to powdered gloves exist, and certain medical institutions and centers have chosen to eliminate the use of powdered gloves.[26]

In the oral surgery literature, only a single case of foreign-body reaction to starch particles has been reported following extraction. It appeared as a firm submucosal nodule, 5 mm in diameter, in the buccal sulcus, which was excised 20 days after teeth extraction.[27]

In implant dentistry, the risk of starch contamination by medical gloves has been discussed by Field[21] and Belvedere and Lambert[28] as a source of complications. Although manufacturers recommend that implants be placed with specific instruments, accidental contact cannot be ruled out. Most surgical instruments, such as spirals drills or depth gauges, are manipulated by gloved hands.

Many foreign bodies are able to produce inflammatory reactions; such reactions could explain some implant failures. They can be introduced at the implant site during dental procedures (eg, endodontic or restorative obturation materials), extraction procedures, or implant surgery.

In order to prevent the occurrence of the IPLs, it is important to thoroughly debride the infected socket following extraction. Implants should be placed after bony socket maturation, when lesions are no longer visible on radiographs.[10] It also appears that foreign bodies may dwell inside the bone and trigger IPL even after thorough debridement, irrigation of the extraction sockets, and sufficient healing time. Contamination of the implant site should be prevented. Implants should never come in contact with saliva, teeth, oral tissues, or the surgeon's gloves. Both careful implant site selection and surgical technique may further reduce the incidence of infected IPL.[6]

There is no consensus as to which therapy should be favored to treat an IPL. Several treatment options have been reported, including antibiotics, lesion excision, debridement, and implant removal. Factors that may influence treatment choice include implant stability, peri-implant probing depth, the status of adjacent teeth, and implant position and angulation. Moreover, the type and quality of the prosthetic rehabilitation should also be taken into account.

Anti-infectious therapy may be effective to treat the acute phase and as an adjunct to surgical treatment. It will rarely suffice to totally eliminate the underlying pathology.

Implant removal will usually be accompanied by considerable bone loss and alteration of the remaining bone along with the surrounding soft tissues. After several months of healing, an autologous bone and/or gingival graft may be necessary. The subsequent healing period, as well as the difficulties inherent to this type of complex treatment, may discourage the patient from pursuing implant therapy. Hence, a conservative surgical treatment, as described in this paper, may be proposed as a valid treatment option as opposed to implant removal.

Furthermore, resection of the nonintegrated portion of the implant allows the removal of a biopsy specimen from the surrounding tissue, which may lead to further treatments. This treatment approach has the advantage of maintaining the implant in function.

Resection of the apical portion of the implant may be indicated when the geometry (implant type) does not allow proper debridement. Implant removal should be considered only as a last resort in cases where debridement has proven unsuccessful.

The extraction of any adjacent tooth that could be the source of infection should be considered if the infection is untreatable. It may therefore be possible to eliminate retrograde infection by debridement and hence maintain the implant if it remains clinically stable and if the infection is restricted to the endosseous region.[8,9,29] Implant resection should facilitate complete mechanical debridement of the inflammatory granulation tissue.

Apex resection may often be indicated in cases of IPL of a perforated implant.[6,10,30] In addition to the conservative surgery, some authors have proposed the use of biomaterials in the resected area.[5,6,10,29] However, there is insufficient data in the literature to warrant their systematic use.

In the present case, only implant resection and debridement were performed. This treatment was chosen in part because of the good implant stability and presence of residual cervical bone around the coronal region. Furthermore, bone fenestration (rather than dehiscence was observed in the present case). Graft material was not used because of the presence of residual cervical bone. After 2 years follow-up, the crestal bone level appears normal in radiographs.

CONCLUSION

Exogenous contamination may provoke an IPL of an osseointegrated implant even several years after implant insertion. Although the occurrence of starch-induced granuloma seems rare, the use of starch-coated gloves should be avoided during surgical procedures. In order to identify the etiology of the pathosis, curetted tissues should be systematically analyzed histopathologically. The development of such a lesion should be considered a complication rather than a failure. In this case, the IPL was treated by implant debridement and resection rather than implant removal. This conservative approach has been effective and has maintained the implant in function.

ACKNOWLEDGMENT

Serge Szmukler-Moncler and Nathalie Nurdin are acknowledged for their efficient help during manuscript review.

REFERENCES

1. Piattelli A, Scarano A, Piattelli M. Abscess formation around the apex of a maxillary root form implant: Clinical and microscopical aspects. A case report. J Periodontol 1995;66:899–903.
2. Piattelli A, Scarano A, Balleri P, Favero G. A. Clinical and histologic evaluation of an active "implant periapical lesion": A case report. Int J Oral Maxillofac Implants 1998;13:713–716.
3. Piattelli A, Scarano A, Piattelli M, Podda G. Implant periapical lesions: Clinical, histologic, and histochemical aspects. A case report. Int J Periodontics Restorative Dent 1998;18:181–187.
4. Scarano A, Di Domizio P, Petrone G, Lezzi G, Piattelli A. Implant periapical lesion: A clinical and histologic case report. J Oral Implantol 2000;26:109–113.
5. Chaffee NR, Lowden K, Tiffee JC, Cooper LF. Periapical abscess formation and resolution adjacent to dental implants: A clinical report. J Prosthet Dent 2001;85:109–112.
6. Reiser GM, Nevins M. The implant periapical lesion: Etiology, prevention, and treatment. Compend Contin Educ Dent 1995;16:768–777.
7. Quirynen M, Vogels R, Alsaadi G, Naert I, Jacobs R, van Steenberghe D. Predisposing conditions for retrograde peri-implantitis, and treatment suggestions. Clin Oral Implants Res 2005;16:599–608.
8. McAllister BS, Masters D, Meffert RM. Treatment of implants demonstrating periapical radiolucencies. Pract Periodontics Aesthet Dent 1992;4:37–41.
9. Flanagan D. Apical (retrograde) peri-implantitis: A case report of an active lesion. J Oral Implantol 2002;28:92–96.
10. Jalbout ZN, Tarnow DP. The implant periapical lesion: Four case reports and review of the literature. Pract Proced Aesthet Dent 2001;13:107–112.
11. Lamontagne J, Durrieu F, Planche JP, Mouillet V, Kister J. Direct and continuous methodological approach to study the ageing of fossil organic material by infrared microspectrometry imaging: Application to polymer modified bitumen. Anal Chim Acta 2001;444:241–250.
12. Skrtic D, Antonucci JM, Eanes ED, Eidelman N. Dental composites based on hybrid and surface-modified amorphous calcium phosphates. Biomaterials 2004;25:1141–1150.
13. Esposito M, Hirsch JM, Lekholm U, Thomsen P. Biological factors contributing to failures of osseointegrated oral implants. (I). Success criteria and epidemiology. Eur J Oral Sci 1998;106:527–551.
14. Esposito M, Hirsch JM, Lekholm U, Thomsen P. Biological factors contributing to failures of osseointegrated oral implants. (II). Etiopathogenesis. Eur J Oral Sci 1998:106:721–764.
15. Oh TJ, Yoon J, Wang HL. Management of the implant periapical lesion: A case report. Implant Dent 2003;12:41–46.
16. Sussman HI. Periapical implant pathology. J Oral Implantol 1998;24:133–138.
17. Tseng CC, Chen YHM, Pang IC, Weber HP. Peri-implant pathology caused by periapical lesion of an adjacent natural tooth: A case report. Int J Oral Maxillofac Implants 2005;20:632–635.
18. Talacko AA, Aldred MJ, Abbott PV, Smith ACH, Nerwich AH. Periapical biopsy? Oral Surg Oral Med Oral Pathol 2000;89:532–534.

19. Selden HS. Central giant cell granuloma: A troublesome lesion. J Endod 2000;26:371–373.
20. Levi PA, Kim DM, Harsfield SL, Jacobson ER. Squamous cell carcinoma presenting as an endodontic-periodontic lesion. J Periodontol 2005;76:1798–1804.
21. Field EA. The use of powdered gloves in dental practice: A cause for concern? J Dent 1997:25:209–214.
22. Antopol W. Lycopodium granuloma: Its clinical and pathological significance, together with a note on granuloma produced by talc. Arch Pathol 1933;16:326–331.
23. Neely J, Davies JD. Starch granulomatosis of the peritoneum. Br Med J 1971;3:625–629.
24. Steiner Z, Mogilner J, Bahar Y. Gastric outlet obstruction due to starch granuloma and peritonitis. Eur J Pediatr Surg 1996;6:364–366.
25. Hunt TK, Slavin JP, Goodsan WH. Starch powder contamination of surgical wounds. Arch Surg 1994;129:825–827.
26. Cuming RG. Reducing the hazards of exposure to cornstarch glove powder. AORN J 2002;76:288–295.
27. Wilson DF, Garach V. Surgical glove starch granuloma. Oral Surg Oral Med Oral Pathol 1981;51:342–345.
28. Belvedere PC, Lambert DL. Negative effects of powdered latex gloves in clinical dentistry. J Long Term Effects Med Implants 1994;4:119–125.
29. Bretz WAG, Matuck AN, de Oliveira G, Moretti AJ, Bretz WA. Treatment of retrograde peri-implantitis: Clinical report. Implant Dent 1997;6:287–290.
30. Shaffer MD, Juruaz DA, Haggerty PC. The effect of periradicular endodontic pathosis on the apical region of adjacent implants. Oral Surg Oral Med Oral Pathol Oral Radiol Endod 1998;86:578–581.
