# Supplementary material for: A novel histopathological classification of implant periapical lesion: A systematic review and treatment decision tree
Source: PLoS One. 2022 Dec 22;17(12):e0277387. doi: 10.1371/journal.pone.0277387 (PMC9778521; doi:10.1371/journal.pone.0277387)
Supplement: S1 File — (ZIP) [file pone.0277387.s001.zip › support files/Included study/Park 2004.pdf]

Journal of Oral Implantology

SUCCESSFUL MANAGEMENT OF DENTAL IMPLANTS IN POSTOPERATIVE MAXILLARY CYST: A CASE REPORT WITH A THIRTEEN-YEAR FOLLOW-UP.

--Manuscript Draft--

**Manuscript Number:**	aaid-joi-D-19-00191R1
**Full Title:**	SUCCESSFUL MANAGEMENT OF DENTAL IMPLANTS IN POSTOPERATIVE MAXILLARY CYST: A CASE REPORT WITH A THIRTEEN-YEAR FOLLOW-UP.
**Short Title:**	Dental Implants in Postoperative Maxillary Cyst: A Report
**Article Type:**	Case Report
**Keywords:**	Postoperative maxillary cyst; maxillary sinus; Dental Implants; peri-implantitis; debridement
**Corresponding Author:**	Philip Kang, DDS<br>Columbia University<br>New York, New York UNITED STATES
**Corresponding Author Secondary Information:**	
**Corresponding Author's Institution:**	Columbia University
**Corresponding Author's Secondary Institution:**	
**First Author:**	Won-Bae Park, DMD, PhD
**First Author Secondary Information:**	
**Order of Authors:**	Won-Bae Park, DMD, PhD
	Young-Jin Kim, MD
	Ji-Young Han, DMD, PhD
	Philip Kang, DDS
**Order of Authors Secondary Information:**	
**Abstract:**	No required for a case report

Powered by Editorial Manager® and ProduXion Manager® from Aries Systems Corporation

Article File
Click here to access/download;Article File;Manuscript_POMC_JOI_Revised 1.docx

SUCCESSFUL MANAGEMENT OF DENTAL IMPLANTS IN POSTOPERATIVE MAXILLARY CYST: A CASE REPORT WITH A THIRTEEN-YEAR FOLLOW-UP.

Dental Implants in Postoperative Maxillary Cyst: A Report

Won-Bae Park, DMD, PhD[1], Young-Jin Kim, MD[2], Ji-Young Han, DMD, PhD[3], Philip Kang, DDS[4*]

[1]Adjunct Professor, Department of Periodontology, School of Dentistry, Kyung Hee University

Private Practice in Periodontics and Implant Dentistry, Seoul, Korea

[2]Adjunct Professor, Nowon Eulji Medical Center, Eulji University, Private Practice in Otorhinolaryngology Clinic, Seoul, Korea

[3]Associate Professor, Department of Periodontology, School of Dentistry, Kyung Hee University, Seoul, Korea

[4*]Assistant Professor, Division of Periodontics, Section of Oral and Diagnostic Sciences, Columbia University, College of Dental medicine, New York, NY, USA

Correspondence

Philip Kang (pyk2104@cumc.columbia.edu), Address: Section of Oral, Diagnostic, and Rehabilitation Sciences Columbia University College of Dental Medicine, #PH7E-124, 630 W. 168 St., New York, NY 10032

Acknowledgments

The authors would like to thank Jung Sun Jang, Medical Device Evaluation Team Manager in Genoss (Suwon, Korea) for his work on histopathology.

Conflicts of Interest

The authors report no conflicts of interest related to this case report.

Abstract

The report is presenting a case of implant placement in the postoperative maxillary cyst (POMC) with a follow-up of 13 years. The postoperative maxillary cyst (POMC) is a complication associated with various surgical interventions involving maxillary sinus diseases such as Caldwell-Luc operation, orthognathic surgery, and sinus grafting procedures. The lesion of POMC is believed to develop as a result of the changes of ciliated cells or the blockage of ostia inside the maxillary sinus. Two dental implants were placed near the lesion that was later confirmed to be POMC. Of the two dental implants placed, one was explanted and the other was successfully managed with surface debridement followed by guided bone regeneration. The removed specimen inclusive of the implant and surrounding tissue was evaluated with clinical photographs, radiographs, and histology and the findings are described in the paper.

Keywords

Postoperative maxillary cyst; maxillary sinus; dental implants; peri-implantitis; debridement

Introduction

The postoperative maxillary cyst (POMC) is a well-documented delayed complication associated with various surgical interventions involving maxillary sinus diseases.[1] The occurrence of POMC is caused by entrapment of ciliated cells of the nasal mucosa or by obliteration of the sinus ostia during radical sinus surgery.[2] Sinus surgeries shown to have developed POMC include the Caldwell-Luc operation,[3] orthognathic surgery,[4,5] and maxillary sinus bone augmentation.[6] Following maxillary sinus bone augmentation for the purpose of dental implant placement, limited reports of POMC cases have been presented at various time points post-operatively, 6 months[6], 3 years[7], and 10 years[8]. Kim et al. reported a case of postoperative maxillary cyst following the sinus bone graft procedure at a 10-year follow-up visit where radiologic and histologic analyses were performed.[8] The authors believe that a tearing or perforation of the maxillary sinus membrane with subsequent entrapment of the membrane with a large amount of graft particles may contribute the development of such cyst.[8] In rare occasions, the POMC

can present as a skin tumor on the cheek as reported by Matsuzaki et al. in more recent publication. [9] In terms of the management, marsupialization or enucleation of the cyst has been proposed as the effective treatment for POMC.[10]

This is a case report of the patient who had the dental implant installed thirteen years ago in an area which later found to be POMC. Clinical presentations, histopathologic evaluation, and radiological manifestations of the implant and its associated complications are discussed in detail. The management of the cystic lesion and the involved implants with enucleation, explantation, and surface decontamination followed by guided bone regeneration is presented as well.

Materials and Methods

A 57-year-old female patient presented to the private clinic in Seoul, Korea, to receive full-mouth rehabilitation of her maxilla with dental implants in September 2004. The patient was a non-smoker and had no systemic disease that would be contraindicated for implant therapy. However, the review of her medical history revealed that the patient had received a Caldwell-Luc operation for the treatment of chronic sinusitis 20 years ago.

The preoperative panoramic radiograph showed severe pneumatization of both maxillary sinuses. The limited volume of residual bone below the sinus floor due to pneumatization necessitated an augmentation procedure, but due to limited mouth opening by the patient, a trans-crestal approach was chosen instead of the traditional lateral-wall sinus grafting (Figure 1a).

The patient received an oral administration of 2.0g of Amoxicillin for prophylactic antibiotic coverage one hour before the procedure. Under local anesthesia, a mucoperiosteal flap was reflected to expose the posterior alveolar ridge. At the site of tooth # 2, Summer's osteotomes were utilized to elevate the sinus floor. The Schneiderian membrane was intact without any visible perforation and there was no addition of any bone graft substitutes. Subsequently, the hydroxyapatite-coated implant (Zimmer TSV 4.8 x 10 mm) was inserted with proper mechanical stability and all nine implants were submerged (Figure 1b). The flaps were sutured passively without tension and primary closure was achieved (Nylon 4-0, Ethilon® 4.0, Ethicon, Cincinnati, OH, USA). A panoramic radiograph was taken immediately after the surgery (Figure 1b). Postoperatively, antibiotics (Ciprofloxacin 500mg, Ildong Pharmaceutical Co. Seoul, Korea) and nonsteroidal anti-inflammatory medication (Etodol® 200mg, Yuhan Co. Seoul, Korea) were prescribed for 10 days. The patient was also instructed to rinse with 0.12% chlorhexidine solution (Hexamedine, Bukwang Pharmaceutical, Seoul, Korea) for 30 seconds, 2 times a day for 1 week.

The healing was uneventful without any complication. After six months of healing, the second-stage surgery was performed to uncover all the implants. One of the implants, # 4, was removed due to non-integration. However, all implants had achieved proper osseointegration and the final prosthesis was delivered after two months (Figure 1c). The patient was closely monitored annually.

Results

In January 2018, thirteen years after the implant placement, the patient complained of masticatory discomfort with gingival bleeding and swelling. Both panoramic radiograph and cone-beam computed tomography (CBCT; rainbowTM CT, Dentium, Suwon, Korea) confirmed evidence of severe bone resorption around the area of implant # 2 associated with clinical signs of peri-implantitis (Figure 1d,

Figure 2). In addition, the panoramic, coronal, and sagittal views of the right maxillary sinus on the CBCT scan showed a unilocular radiolucency with a well-defined margin. The breadth of the nasal cavity was abnormally wide. The sinus was opacified and the natural ostia seemed to be obstructed (Figure 2a-c). The axial view further confirmed the expansive nature and the disappearance of the anterior lateral bone plate near what appeared to be a cystic lesion (Figure 2d). The decision was made to section the bridge and remove the implant #2.

Two weeks after the explantation, the patient presented with severe facial swelling. The buccal vestibule showed significant edema and active discharge of exudate with oro-antral communication at the site (Figure 3a). A re-entry procedure through the lateral wall of the sinus was necessary in order to evaluate and manage the site of active infection.

In the area of explantation, a fistulectomy was performed. The horizontal marginal incision and vertical incisions were used to reflect the facial mucoperiosteal flap. Upon reflection, the bone plate was noted to be missing and the proliferative granulation tissue was present (Figure 3b). The soft tissue mass was resected from the surrounding bony wall and the implant body was visible (Figure 3c). The removed specimen was fixed in neutral buffered formalin solution (Sigma Aedrich, St. Louis, MO, USA) for histopathological examination.

A titanium brush (Genoss, Suwon, Korea) was utilized to debride and mechanically decontaminate the exposed implant surface (Figure 3c). The implant surface was further treated for chemical detoxification with tetracycline HCl (Oxymycin 500mg, Chong Kun Dang Pharmaceutical Co., Seoul, Korea) for five minutes at 100 mg/mL with saline solution (Figure 3d). The cystic cavity was thoroughly irrigated with saline solution. Then, biphasic calcium phosphate (Osteon III, Genoss, Suwon, Korea) was condensed

around the implant surface as well as the enucleated space, but without any barrier membrane (Figure 3e). The flaps were sutured passively with 4-0 Nylon (Figure 3f). The radiologic images confirmed that space was uniformly occupied by the bone graft as shown in Figures 3g, 3h, and 3i. Post-operatively, the patient was covered with Ciprofloxacin 500mg (Ildong Pharmaceutical Co. Seoul, Korea) and non-steroidal anti-inflammatory drug (Etodol® 200mg, Yuhan Co. Seoul, Korea) for two weeks. The patient was also advised to rinse with 0.12% chlorhexidine solution (Hexamedine, Bukwang Pharmaceutical, Seoul, Korea) for thirty seconds, twice a day for two weeks.

The specimen was confirmed histopathologically as POMC. Some cilia were observed, but ciliary loss due to chronic inflammation was also evident. The cystic lesion was lined with pseudostratified columnar epithelial cells with numerous inflammatory cells infiltrating into the connective tissue (Figure 4a). Proliferative granulation tissue was observed with innumerable lymphocytic cells (figure 4b).

Within two weeks postoperatively, all clinical symptoms disappeared, and there was no recurrence during the first year (Figure 5a). At the one-year visit, the CBCT showed increased bone density in the space and the grafted area showed a high degree of homogeneity (Figure 5b and 5c).

Discussion

Traditionally, a radical surgical approach such as Caldwell-Luc operation was commonly utilized to remove the maxillary sinus mucosa in conjunction with inferior meatal antrostomy. In addition to POMC, other examples of delayed complications have been described such as chronic maxillary neuralgia, infraorbital nerve hyperesthesia, and recurrent sinusitis.[11,12] As evident in the patient in the current report,

facial bone deformities have also been reported at long-term follow-ups.[12] Because of these reasons, rather than the traditional approach, the more conservative surgical technique combined with endoscopes has gained popularity in recent years. [13,14] The authors also believe that any patient with a history of prior sinus surgery should be evaluated with proper CBCT imaging prior to any implant or sinus graft surgery.

Several reports of implant installation and maxillary sinus augmentation in cysts, mucoceles, polypoid mass, and severely thickened mucosal lining are available. [13-18] In these lesions, failures of dental implants and grafts were also frequently documented. [19.] However, to the best of authors' knowledge, there has not been any report of implant placement into POMC or the management of POMC-affected implant with decontamination and guided bone regeneration.

The patient reported in the present paper had been inaccurately diagnosed as having normal sinus at the time of implant placement. Because of radiographic misinterpretation, a trans-crestal sinus elevation was undertaken in the area of POMC with subsequent implant placement. The osseointegration and masticatory function were maintained without any complications until the implant was removed thirteen-year postoperatively due to peri-implantitis. An episode of active infection following the implant removal necessitated a surgical entry and this provided an opportunity to obtain the specimen for histological evaluation for a more definitive diagnosis of the lesion as POMC. In retrospect, the history of Caldwell-Luc operation for the treatment of chronic sinusitis twenty years prior to the implant surgery also prompted speculation of the lesion being POMC.

The common differential diagnosis of cyst near the maxillary sinus includes pseudocyst and mucous retention cyst. Pseudocyst does not have epithelial lining whereas mucous retention cyst presents as a

dome-shaped cyst with epithelial lining.[20] The histopathologic finding of POMC is characterized by the lining of ciliated pseudostratified columnar epithelium. [8,10] In this patient's specimen, some ciliary loss was also found because the POMC lesion was influenced by the presence of peri-implantitis. This is consistent with the results of Gudis et al. where the loss of ciliary function was associated with disturbed mucociliary clearance and ventilation of the sinus.[21] In particular, the unilocular lesion in the present patient was separated from the confine of the maxillary sinus and the facial bone plate was also non-existence.

The accessibility for the POMC lesion enucleation and detoxification of the contaminated implant surface is of the essence. In this patient, at the time of implant #2 removal, implant #3 was decided to be left in place due to lack of mobility and relatively well-maintained osseointegration. However, when the surgical entry had to be initiated when the patient returned with an active infection, the treatment of contaminated implant surface was also indicated. Following numerous recommendations, although the efficacy is inconclusive, mechanical debridement with titanium brush and chemical detoxification with tetracycline HCl were utilized for this particular case.[22, 23, 24] Currently, there isn't any verification method to assess the completeness of detoxification and biofilm removal. Thus, the clinical decision largely depends on the expertise of individual clinicians. In this patient, a titanium brush was used until there was no visible debris attached to the implant surface and the implant surface appeared much shinier. Much effort was made to access and debride as many exposed threads as possible. Moreover, Tetracycline HCl 100 mg / mL was applied onto the exposed surface for at least five minutes after using the titanium brush. Theoretically, Tetracycline HCl should eliminate bacteria on the contaminated implant surface, remove endotoxin, and inhibit collagenase activities.[25,26] However, the clinical efficacy of Tetracycline HCl, its

appropriate concentration, and the duration of application to exert the proposed effect has not been extensively documented in the literature.

The successful outcome of guided bone regeneration around the enucleated cystic cavity and contaminated implants may not be warranted. The true re-osseointegration with newly formed bone is also questionable although promising outcomes have been described in some reports. [27,28] In this report, an attempt was made to regenerate the bone because the area was well-isolated, thorough enucleation was possible, and the detoxification of the implant surface was achievable. As shown in the one-year CBCT, the grafted area was verified to have completely filled in with highly dense and homogenous bone.

Conclusion

Successful management and treatment of Postoperative Maxillary Cyst (POMC) and the affected implants can be achieved with thorough enucleation, mechanical debridement, and chemical detoxification followed by guided bone regeneration. The treated site is well-maintained for one year without any evidence of radiographic and clinical complications.

Abbreviations

CBCT cone beam computed tomography

HA hydroxyapatite

POMC postoperative maxillary cyst

References

1. Hasegawa M, Saito Y, Watanabe I, Kern EB. Postoperative mucoceles of the maxillary sinus. *Rhinology*. 1979;17:253-256.

2. Yamamoto H ,Takagi M. Clinicopathologic study of the postoperative maxillary cyst. ***Oral Surg Oral*** *Med Oral Pathol Oral Radiol***.*** 1986;62:544-548.

3. Unger JM, Dennison BF, Duncavage JA, Toohil RJ. The radiological appearance of the post-Caldwell-Luc maxillary sinus. *Clin Radiol.* 1986;37:77-81.

4. Sugar AW, Walker DM, Bounds GA. Surgical ciliated (postoperative maxillary) cysts following mid-face osteotomies. *British J Oral Maxillofac Surg***.** 1990;28:264-267.

5. Amin M, Witherow H, Lee R, Blenkinsopp P. Surgical ciliated cyst after maxillary orthognathic surgery: report of a case. *J Oral Maxillofac Surg.* 2003;61:138-141.

6. Misch CM, Misch CE, Resnik RR, Ismail YH, Appel B. Post-operative maxillary cyst associated with a maxillary sinus elevation procedure: a case report. *J Oral Implantol* 1991;17:432-437.

7. Lockhart R, Ceccaldi J, Bertrand JC. Postoperative maxillary cyst following sinus bone graft: report of a case. *Int J Oral Maxillofac Implants.* 2000;15:583-586.

8. Kim JJ, Freire M, Yoon JH, Kim HK. Postoperative Maxillary Cyst After Maxillary Sinus Augmentation. *Journal of Craniofacial Surgery*. 2013; 24(5):e521–e523.

9. Matsuzaki Y, Kaneko T, Makita E, Rokunohe A, Minakawa S, Nakano H, Sawamura D. Postoperative maxillary cyst presenting as a skin tumour on the cheek. *Eur J Dermatol*. 2017 Aug 1;27(4):433-434.

10. Yoshikawa Y, Nakajima T, Kaneshiro S, Sakaguchi M. Effective treatment of the postoperative maxillary cyst by marsupialization. *J Oral Maxillofac Surg.* 1982;40:487-491.

11. Ferekidis E, Tzounakos P, Kandiloros D, Kaberos A, Adamopoulos G. Modifications of the Caldwell-Luc procedure for the prevention of post-operative sensitivity disorders. *J Laryngol Otol.* 1996;110:228-231.

12 . DeFreitas J, Lucente FE. The Caldwell-Luc procedure: institutional review of 670 cases: 1975-1985. *Laryngoscope.* 1998;98:1297-1300.

13. Abu-Ghanem S, Kleinman S, Horowitz G, Balaban S, Reiser V, Koren I. Combined maxillary sinus floor elevation and endonasal endoscopic sinus surgery for coexisting inflammatory sinonasal pathologies: a one-stage double-team procedure. *Clin Oral Implants Res.* 2015; 26:1476-1481

14. Falco A, Amoroso C, Berardini M, D'Archivio L. A retrospective study of clinical and radiologic outcomes of 69 consecutive maxillary sinus augmentations associated with functional endoscopic sinus surgery. *Int J Oral Maxillofac Implants.* 2015;30:633-638.

15. Garg AK, Mugnolo GM, Sasken H. Maxillary antral mucocele and its relevance for maxillary sinus augmentation grafting: a case report. *Int J Oral Maxillofac Implants.* 2000;15:287-290.

16. Cortes AR, Corrêa L, Arita ES. Evaluation of a maxillary sinus floor augmentation in the presence of a large antral pseudocyst. *J Craniofac Surg.* 2012;23:e535-e537.

17. Kara MI, Kirmali O, Ay S. Clinical evaluation of lateral and osteotome techniques for sinus floor elevation in the presence of an antral pseudocyst. *Int J Oral Maxillofac Implants.* 2012;27:1205-1210.

18. Kfir E, Goldstein M, Abramovitz I, Kfir V, Mazor Z, Kaluski E. The effects of sinus membrane pathology on bone augmentation and procedural outcome using minimal invasive antral membrane balloon elevation. *J Oral Implantol.* 2014;40:285-293.

19. Mardinger O, Manor I, Mijiritsky E, Hirshberg A. Maxillary sinus augmentation in the presence of antral pseudocyst: a clinical approach. *Oral Surg Oral Med Oral Pathol Oral Radiol Endod.* 2007;103:180-184.

20. Gardner DG. Pseudocysts and retention cysts of the maxillary sinus. *Oral Surg Oral Med Oral Pathol.* 1984;58:561-567.

21. Gudis D, Zhao KQ, Cohen NA. Acquired cilia dysfunction in chronic rhinosinusitis. *Am J Rhinol Allergy*. 2012;26:1-6.

22. Valderrama P, Wilson TG Jr. Detoxification of implant surfaces affected by peri-implant disease: an overview of surgical methods. *Int J Dent.* 2013;2013:740680.

23. An YZ, Lee JH, Heo YK, Lee JS, Jung UW, Choi SH. Surgical treatment of severe peri-implantitis using a round titanium brush for implant surface decontamination: a case report with clinical reentry. *J Oral Implantol.* 2017;43:218-225.

24. Hurr Y, Woo JA, Kwon YH, Park JB, Heo SJ, Chung JH. Implant surface conditioning with tetracycline-HCl : a SEM study. *Key Engineering Materials.* 2008;361-363:849-852.

25. Zablotsky MH, Diedrich DL, Meffert RM. Detoxification of endotoxin-contaminated titanium and hydroxyapatite-coated surfaces utilizing various chemotherapeutic and mechanical modalities. *Implant Dent.* 1992;1:154-158.

26. Golub LM, Goodson JM, Lee HM, et al. Tetracyclines inhibit tissue collagenases. Effects of ingested low-dose and local delivery systems. *J Periodontol.* 1985;56(11 Suppl):93-97.

27. Renvert S, Polyzois I, Maguire R. Re-osseointegration on previously contaminated surfaces: a systematic review. *Clin Oral Implant Res.* 2009; 20 (suppl 4):216–227.

28. Mohamed S, Polyzois I, Renvert S, Claffey N. Effect of surface contamination on osseointegration of dental implants surrounded by circumferential bone defects. *Clin Oral Implants Res.* 2010;21:513–519.

Captions

FIGURE 1.

a. Pre-operatory panoramic radiograph

b. Immediately after the implant surgery

c. Six months after the surgery. Implant # 4 was removed due to non-integration

d. 13 years post-loading of the implants. Severe bone loss around implant #2 (white arrows)

FIGURE 2

a. Panoramic view of CBCT scan. A separate compartment below the sinus floor where two implants are located. The cavity is wide and opacified.

b. Coronal view of CBCT. The lesion is isolated from the maxillary sinus by fibrous septum.

c. Sagittal view of CBCT. The lesion is clearly lined with the thin cortical bone.

d. Axial view of CBCT. The lesion is expansive in medio-lateral dimension (yellow arrows) with missing facial bone plate (white arrow).

FIGURE 3

a. The site of explantation showing active discharge of pus

b. Careful dissection and reflection of the buccal flap. Underlying granulation tissue is visible.

c. Enucleation of the lesion is performed in conjunction with the mechanical debridement with a titanium brush

d. Tetracycline HCl is mixed with saline solution and applied for 5 minutes.

e. The cavity is filled with biphasic calcium phosphate.

f. The flap is sutured without tension

g. Panoramic radiograph immediately after the surgery

h. Panoramic view of CBCT immediately after bone grafting

i. Coronal view of CBCT. The cavity is completely filled with bone graft.

FIGURE 4

a. The cystic lesion is lined with ciliated, pseudostratified columnar epithelium (H&E stain, 100 x). Ciliary loss is also observed.

b. The lymphocytic cells infiltrated in the connective tissue. Highly inflammatory proliferative tissue and cholesterol clefts are seen (H&E stain, 50 x)

FIGURE 5

a. Clinical photograph of the area one year after the surgery

b. Panoramic view of the CBCT taken one year after the treatment showing increased radiopacity of the grafted site

c. Coronal view of CBCT. There is increased bone density with a high degree of homogeneity.

 Click here to access/download;Figure;Figure 1.tif

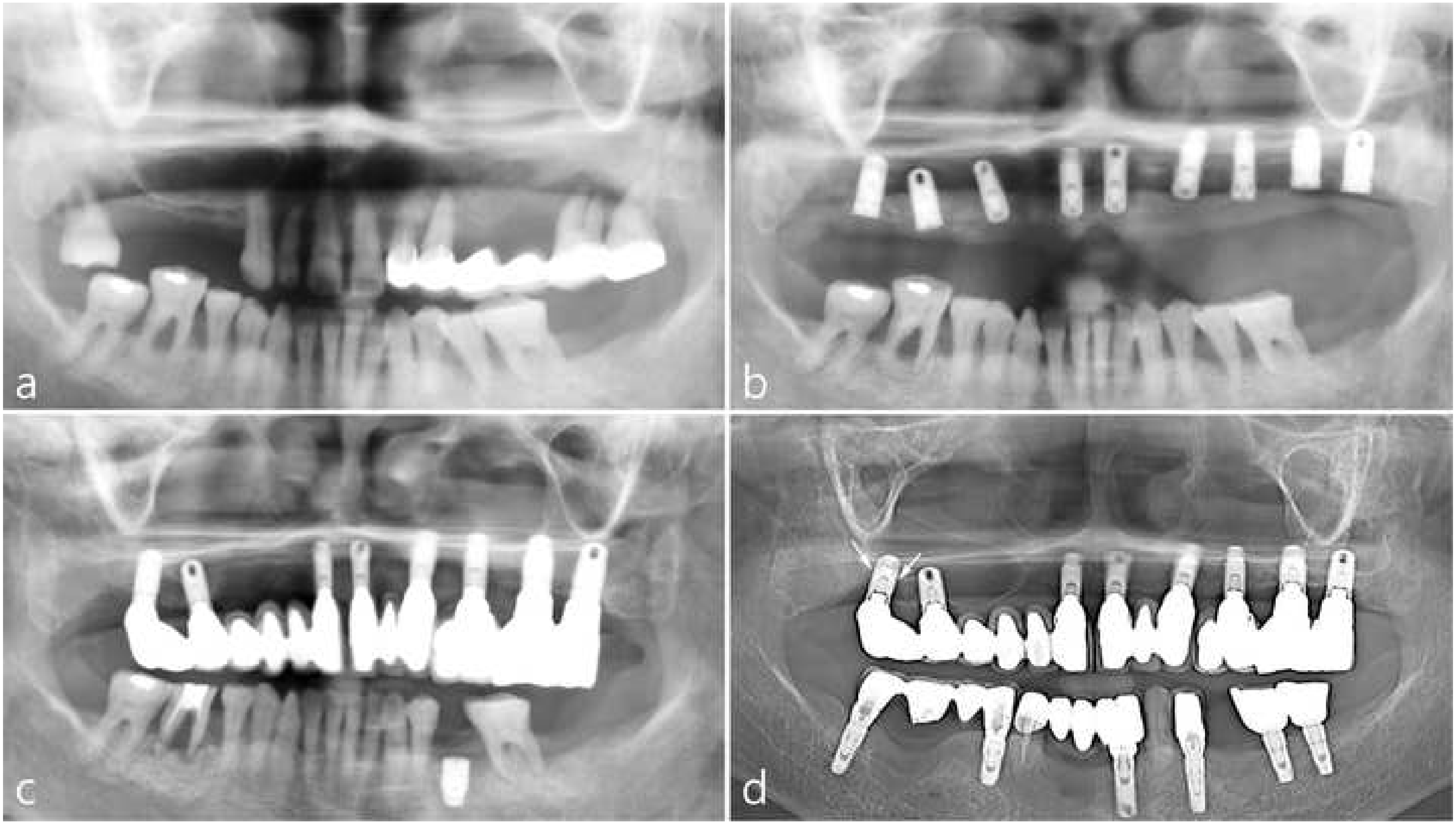

Figure

Click here to access/download;Figure;Figure 2.tif

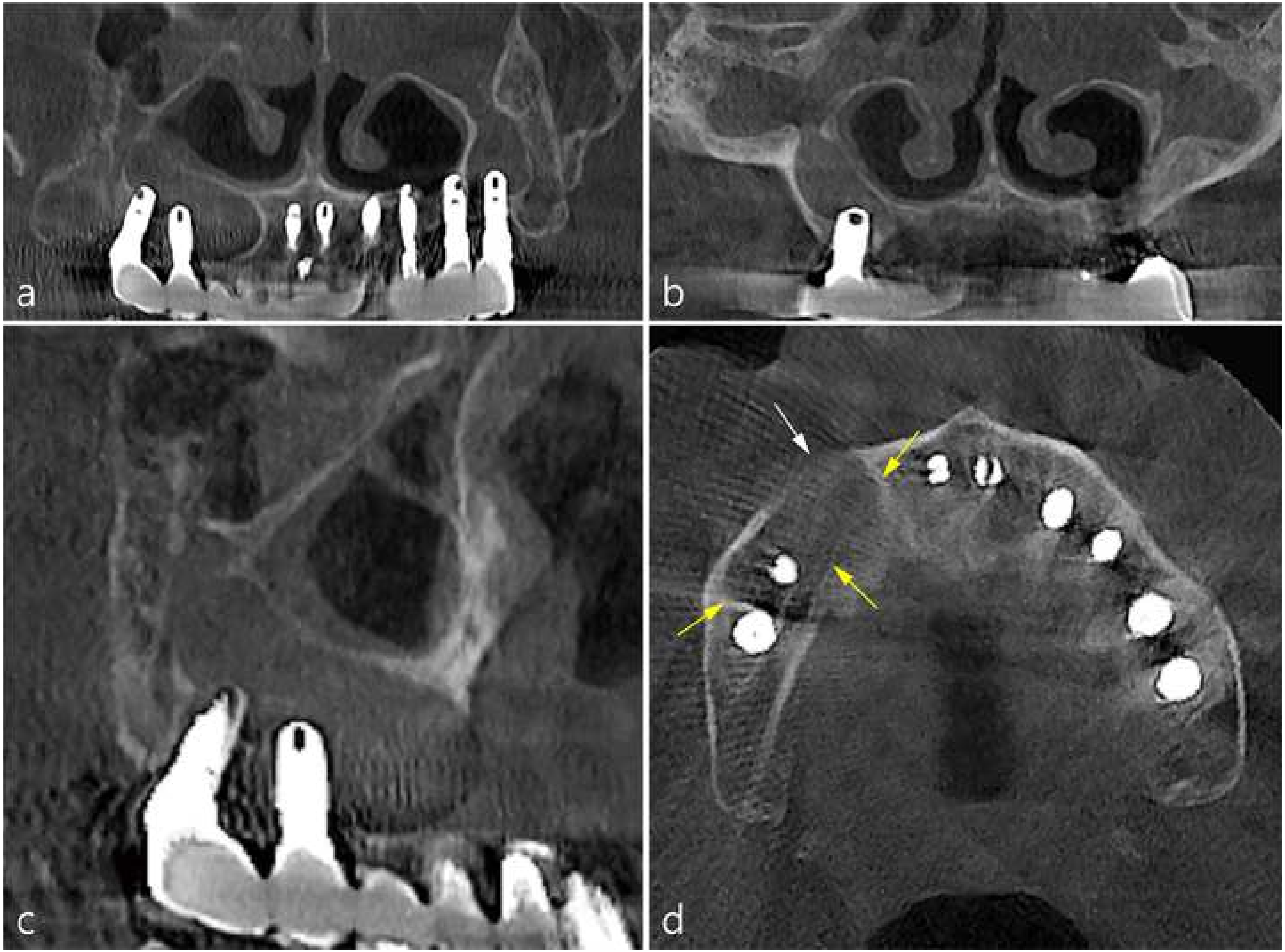

 Click here to access/download;Figure;Figure 3.tif

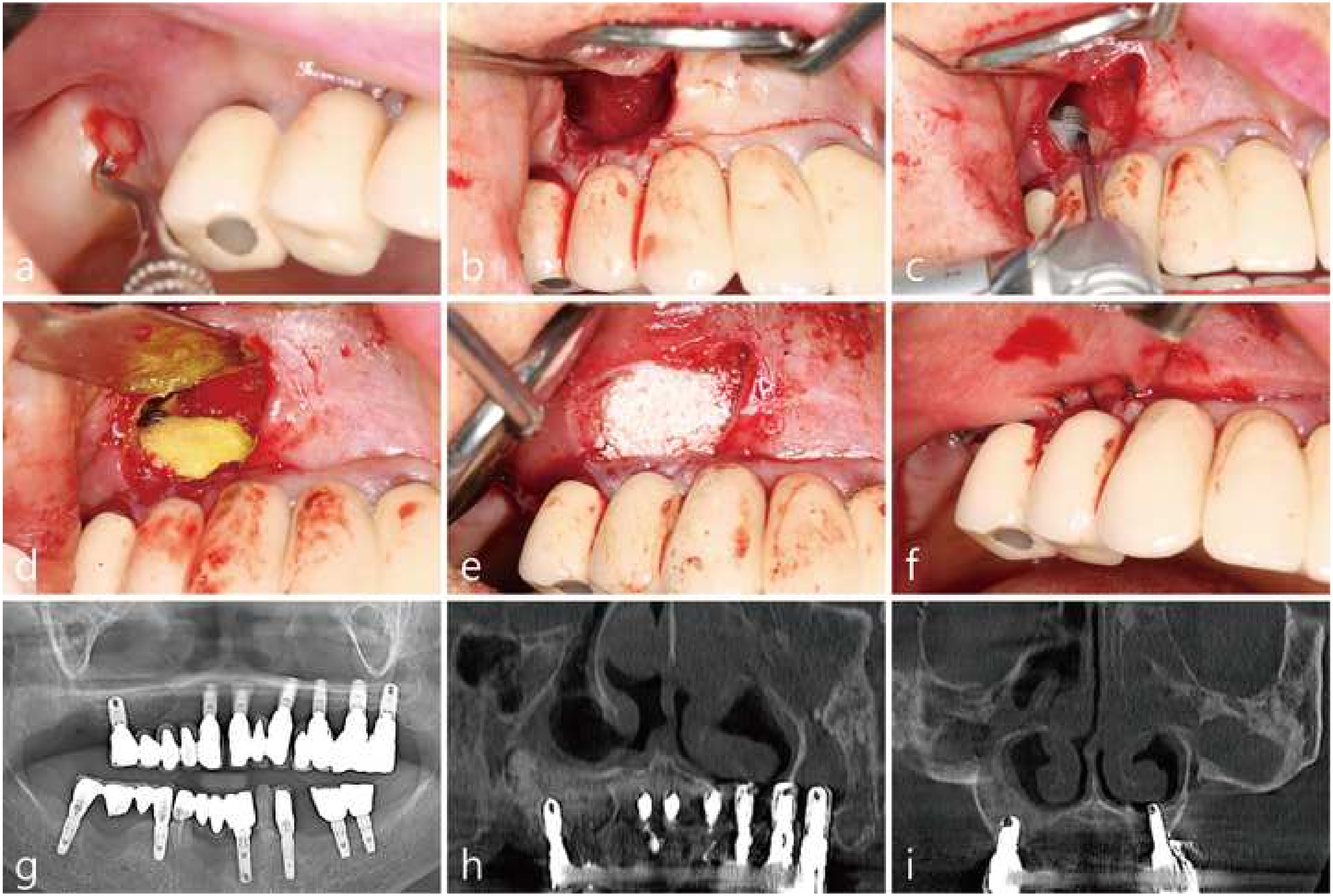

Click here to access/download;Figure;Figure 4.tif

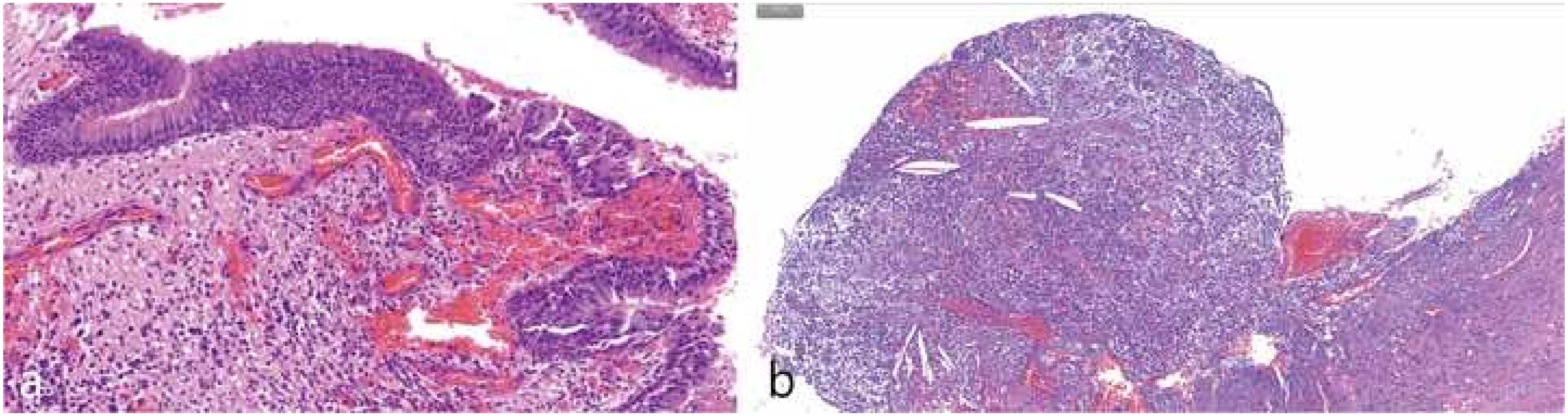

Click here to access/download;Figure;Figure 5.tif

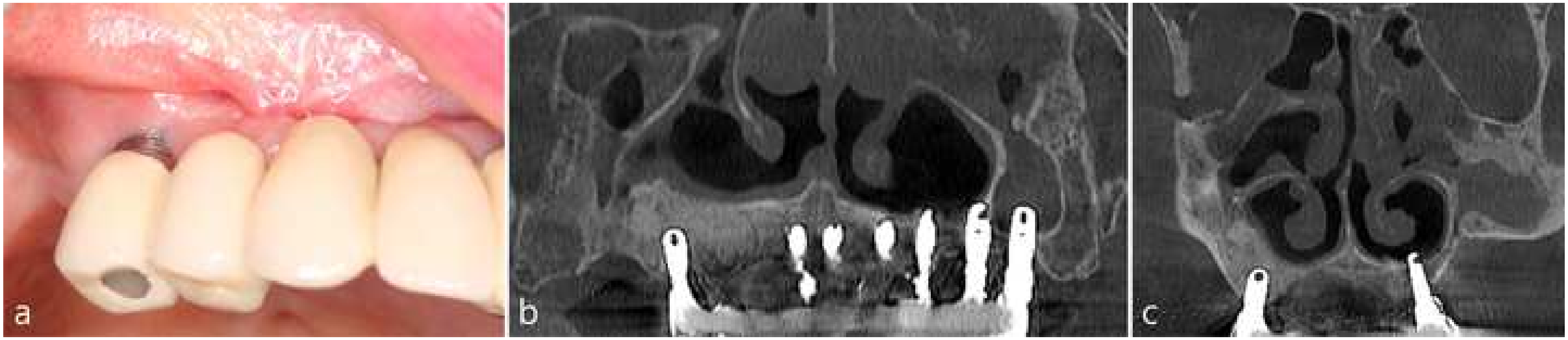

Click here to access/download
Cover Letter
POMC_JOI_Cover Letter.docx

Click here to access/download
Copyright Form
Kang_copyright transfer.pdf

Click here to access/download
Rebuttal Letter (for revisions)
JOI rebuttal letter.docx
