# Supplementary material for: A novel histopathological classification of implant periapical lesion: A systematic review and treatment decision tree
Source: PLoS One. 2022 Dec 22;17(12):e0277387. doi: 10.1371/journal.pone.0277387 (PMC9778521; doi:10.1371/journal.pone.0277387)
Supplement: S1 File — (ZIP) [file pone.0277387.s001.zip › support files/Included study/piattelli 1995.pdf]

Abscess Formation Around the Apex of a Maxillary Root Form Implant: Clinical and Microscopical Aspects. A Case Report*

A. Piattelli, A. Scarano, and M. Piattelli

THIS CASE REPORT DESCRIBES THE OCCURRENCE of an abscess in the periapical area of a root form maxillary titanium implant, with a radiolucent appearance and purulent discharge. The removal of the infected periapical tissues was carried out but the clinical symptoms of infection persisted, an attempt at guided tissue regeneration with a freeze-dried dura mater membrane notwithstanding, and it was necessary to remove the implant. The microscopical examination showed that it was possible to observe nonviable bone tissue, with absence of bone cells and areas of bone resorption in the apical portion of the implant. These microscopical features were compatible with an acute localized suppurative osteomyelitis. *J Periodontol 1995;66:899–903.*

Key Words: Dental implants/adverse effects; osteomyelitis, periapical abscess; periapical periodontitis; titanium.

The widespread use of root form implants in the last few years has produced different types of complications. Many reports have described the diagnosis and treatment of peri-implantitis; i.e., peri-implant infections with involvement of the coronal portion of the implants. More recently another type of complication, characterized by an infection located at the apex of an implant, has been described.[1] In this case it is possible to observe bleeding, suppuration, loss of alveolar bone, and pocket formation.[1] Etiologic factors can include surgical trauma, overheating of bone, or overloading of the implants at an early stage;[1] this overloading could produce microfractures around the implant.[2]

Meffert et al.[3,4] have divided implants with complications in three types: 1) ailing implant: bone loss with pocket formation, the implant is static at maintenance checks; 2) failing implant: bone loss irrespective of therapy, bleeding upon probing, purulent exudate; 3) failed implant: mobility, dull sound on percussion, peri-implant radiolucency. The ailing and failing implant may be treated: the failed implant must be removed because bone loss will continue.[3–4]

The aim of the present case report is an evaluation of the clinical and microscopical aspects of a failed implant in which there had been the development of an abscess at the apex of a maxillary titanium implant.

CASE REPORT

A 40-year-old female patient came to our Department for the loss of teeth 14 and 24 due to caries (Fig. 1). These teeth had been removed about 10 years earlier. In February 1994 the patient underwent the insertion of two titanium implants (length 15 mm, diameter 3.75 mm)† (Fig. 2). Periapical radiographs of the upper premolar regions, taken just before the implant insertion, showed the complete absence of bone pathology. A routine sterile surgical technique under generous saline irrigation was employed; an antibiotic was prescribed for 10 days postoperatively. The postoperative course was uneventful and the surgical site healed without complications. Two months later there was a sudden onset of pain and swelling in the vestibular mucosa in the upper right premolar region, while the implant placed in the upper left premolar region was incorporated without complications. A fistula, with a slight purulent discharge, developed in the following days (Figs. 3 and 4). An intraoral radiograph showed at the apex of the implant the presence of a radiolucent, well-defined area of about 0.5 cm in diameter, involving the five upper spires of the implant (Fig. 5). An antibiotic treatment was instituted and a partial resolution of the clinical signs of the infection was noted. In May 1994 it was decided to carry out the removal of the infected tissue at the apex of the implant. Under local anesthesia a mu-

*Dental School, University of Chieti, Italy.

†Implant Innovations, West Palm Beach, FL.

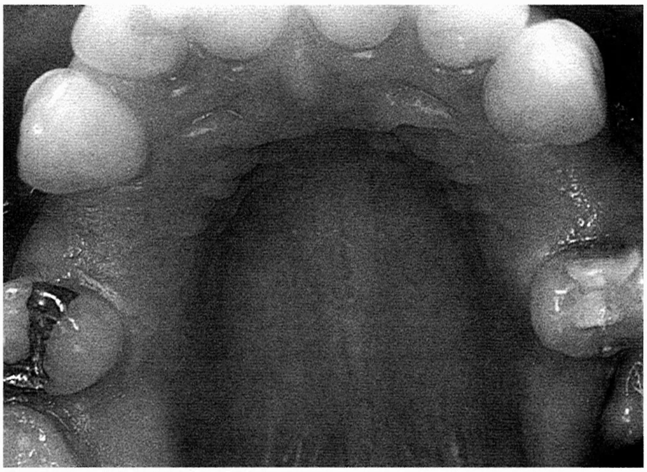

Figure 1. Clinical aspect of the patient.

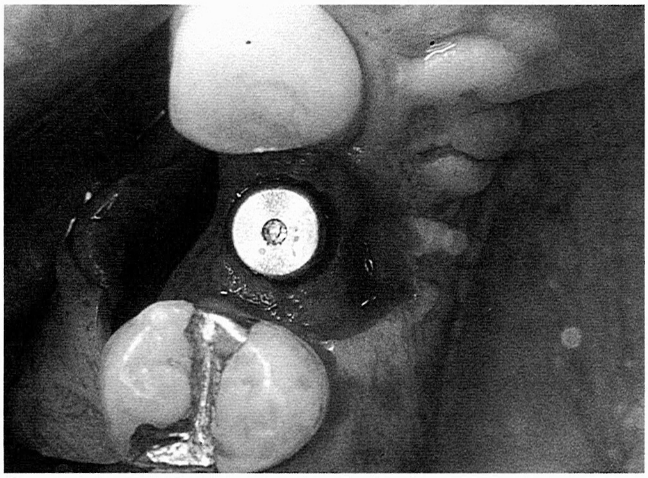

Figure 2. Implant insertion.

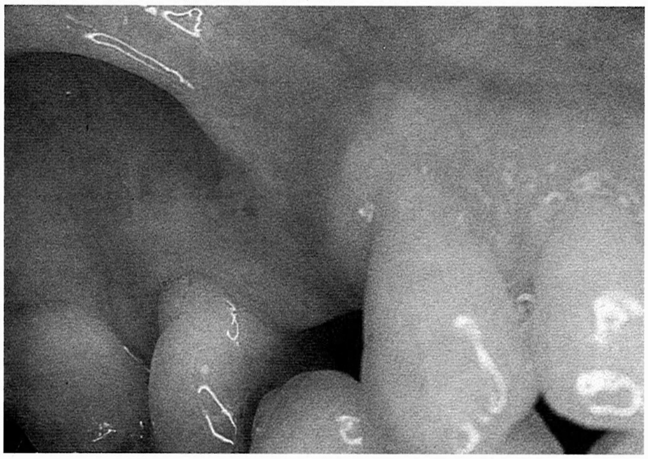

Figure 3. It is possible to see a fistula with purulent discharge.

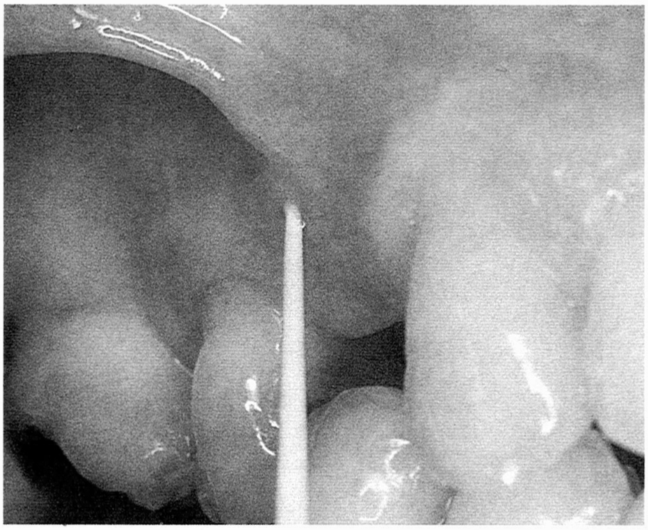

Figure 4. A gutta-percha point is inserted inside the fistula.

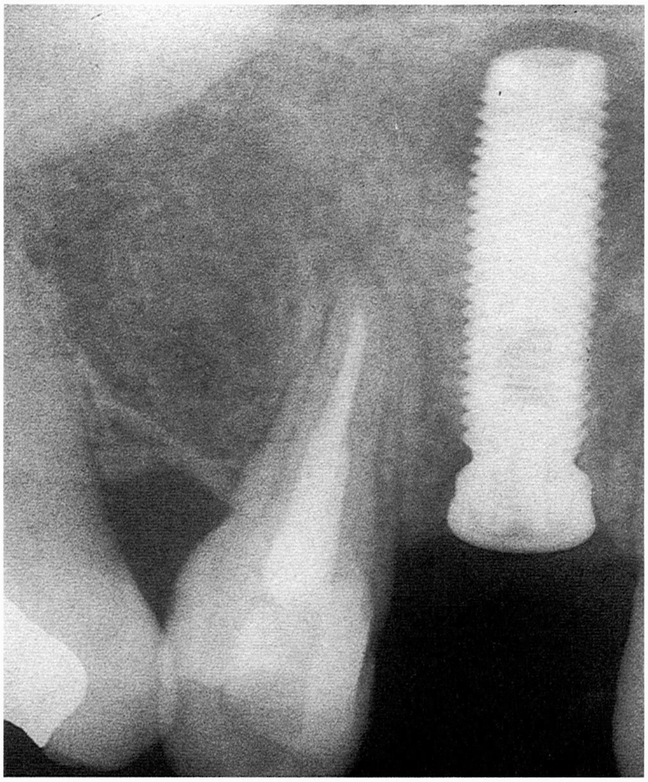

Figure 5. A radiolucent lesion is present at the apex of the implant.

coperiosteal flap was elevated (Fig. 6) and all the infected granulomatous tissue was removed with a titanium curet, the area was generously irrigated with saline solution and antibiotics (Fig. 7). A freeze-dried dura mater (FDDMA) membrane[‡] was inserted over the osseous defect, but the purulent discharge continued unabated, and in June 1994 it was decided to remove the implant. Under local anesthesia a mucoperiosteal flap was elevated, the FDDMA membrane was removed and under profuse saline irrigation the implant with the surrounding tissues were unscrewed (Fig. 8) with the use of torsional force; a dia-

[‡]Lyodura, Braun, Melsungen, Germany.

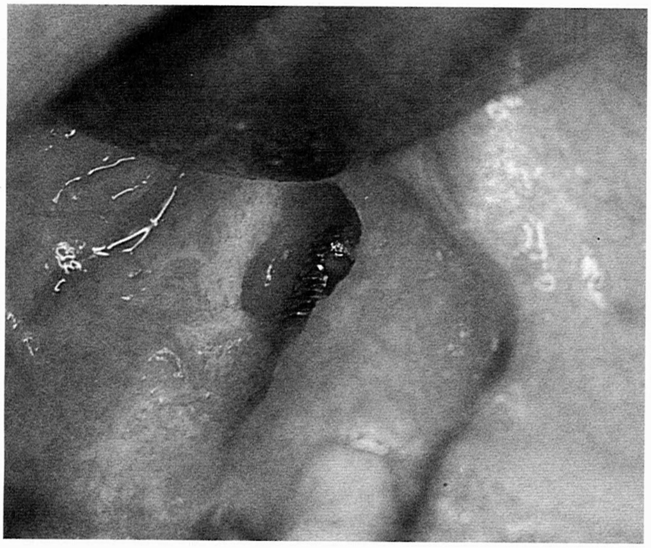

Figure 6. Clinical appearance after the flat elevation; it is possible to see the bone fenestration at the level of the apical part of the implant.

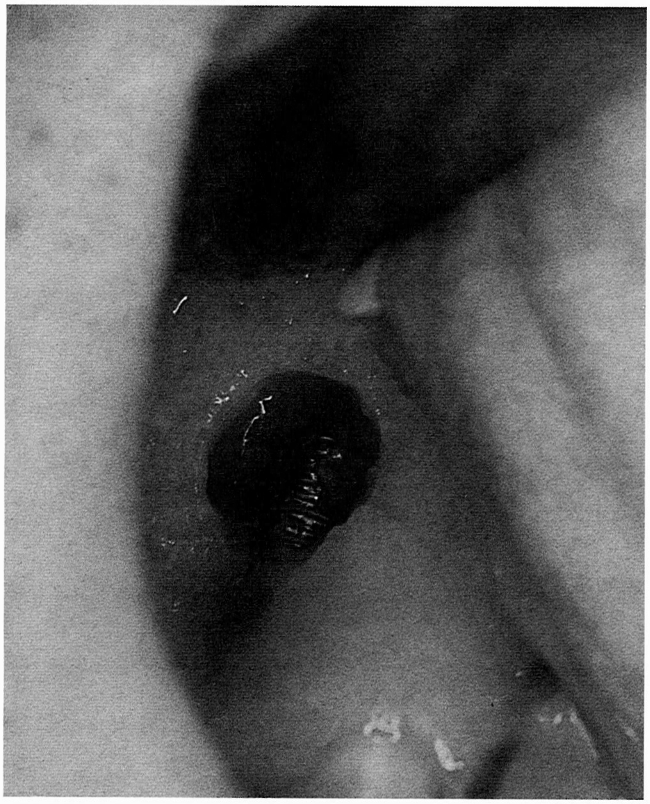

Figure 7. A curettage and debridement of the apical portion of the implant has been carried out.

mond bur was not used, in order to save the maximum amount of bone. The specimen was immediately fixed in 10% buffered formalin and processed to obtain thin ground sections according to the cutting-grinding technique.[5] Briefly the specimen was dehydrated in an ascending series of alcohols and embedded in a glycolmeth-

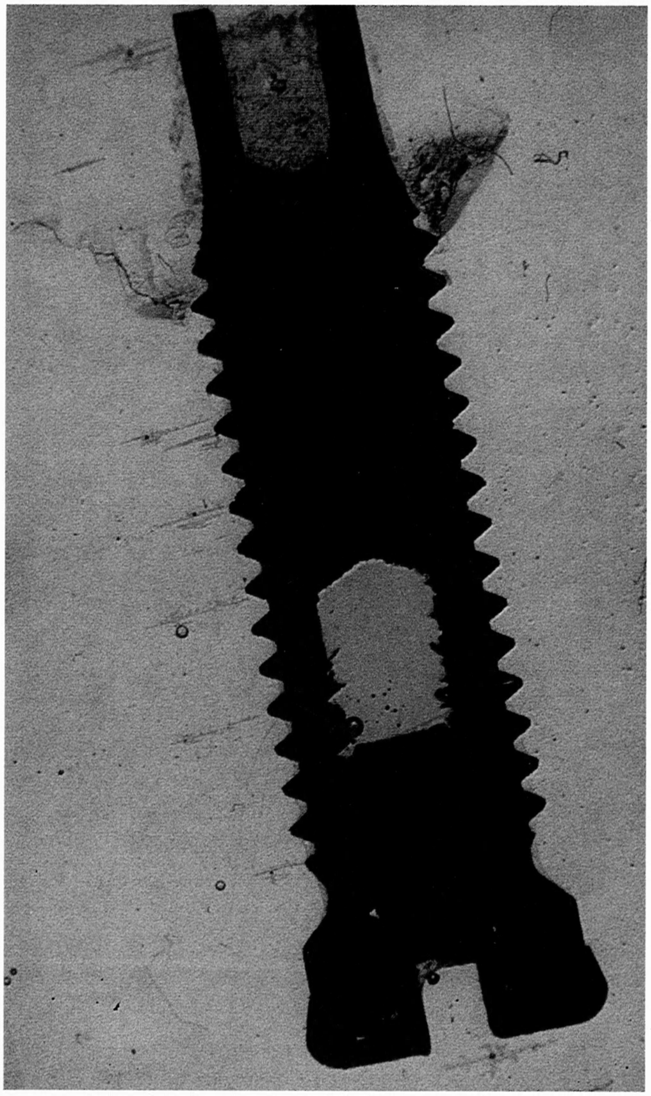

Figure 8. A low-power view of the implant; it is possible to see that in the most apical part of the implant is present a tissue stained with basic fuchsin (original magnification ×6).

acrylate resin.[§] After polymerization the specimen was sectioned with a diamond saw at a thickness of about 200 μm and ground down to about 30 μm. After polishing the slides were stained with basic fuchsin-methylene blue or with toluidine blue, and observed under normal light in a microscope.[||]

The microscopic examination showed that it was possible to observe small colonies of bacteria around the outer perimeter of the implant. There was the presence of tissue that stained with basic fuchsin inside the hole in the apical part of the implant (Fig. 8). It was not possible to recognize viable bone trabeculae or a peripheral border of osteoblasts; the osteocytes were absent, while it was possible to see areas of bone resorption (Figs. 9 and 10).

§Technovit 7200 VLC, Kulzer, Wehreim, Germany.
||Leitz, Wetzlar, Germany.

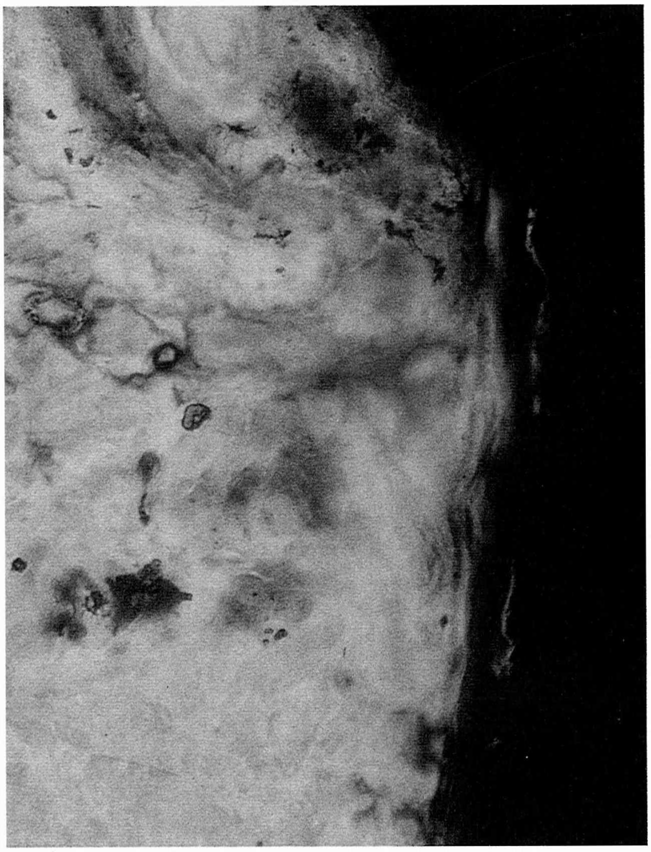

Figure 9. Higher magnification of the tissue inside the apical portion of the implant; presence of necrotic bone with no recognizable trabeculae. No bone cells can be recognized (basic fuchsin; original magnification ×200).

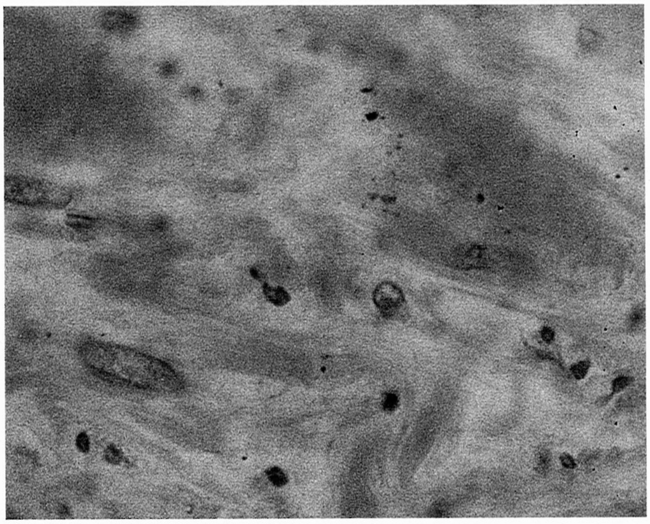

Figure 10. The bone has lost its lamellar appearance (basic fuchsin; original magnification ×1,000).

The microscopical pattern was compatible with a diagnosis of acute localized suppurative osteomyelitis.

DISCUSSION

It has been reported that implant failure can be due to a premature loading by overlying dentures, bone overheating or bacterial colonization.[1,2,6] Zarb[7] has stated that biomechanical factors are responsible for most implant failures.

Implant failures are characterized by implant mobility, marginal swelling and redness, bleeding and/or suppuration on probing, increased probing depth, peri-implant radiolucency, and loss of alveolar bone height.[8–9] In peri-implantitis it has been suggested that the remaining natural teeth may act as a reservoir of periodontopathic bacteria which can colonize and involve the peri-implant tissues.[10]

Apse et al.[11] found that the prevalence of severity of marginal inflammation seems to be similar around teeth and implants in partially edentulous jaws in man. Pathogens associated with periodontal disease have been isolated from failing implants.[12] Mombelli et al.[8] found that the proportions of subgingival spirochetes, fusiforms, motile rods, and curved rods increased in specimens retrieved from implants which presented a mean probing depth of 8.5 mm and alveolar bone loss as compared with specimens obtained from implants with healthy marginal tissues.

Rosenberg et al.[9] found that implants failing due to infection showed high proportions of spirochetes and motile rods. One circumstance that could be implicated, in our case, in the etiology of the peri-implant abscess is the presence of a resting osteitis following a not yet healed periapical tooth lesion; this fact can however be excluded because the patient had lost the premolars many years previously, and periapical radiographs, taken before the implant insertion did show a complete absence of bone pathology. Treatment of an infection at the apex of an implant can tax the surgical capabilities of the clinician. Three approaches have been proposed for mandibular involved implants, an intraoral transmandibular approach, an intraoral periosteal dissection, and an extraoral approach.[1] Aggressive management with debridement of the affected site must be carried out, if we are to hope for a resolution of the pathological process. In our case the morphology of the apical part of the implant probably prevented a thorough curettage and the infective process was not eliminated, with ultimate loss of the implant. Goldman[6] thinks that a complete removal of all infected tissues around the apex of an implant is probably questionable.

Guided tissue regeneration (GTR) has also been used in recent years for treatment of ailing and failing implants to protect the eventually grafted material and to keep it in place[3–4] and Goldman[6] reported the successful use of GTR around a failing implant. However, Grunder et al.[13] in a histologic study in the beagle dog found that the treatment of peri-implantitis using GTR did not enhance

clinical parameters of bone formation around "diseased" implants, while bone regeneration around sterile implants was obtained by Dahlin et al.[14] and by Becker et al.[15–16] Freeze-dried dura mater (FDDMA) membranes have been reported recently to be useful in guided tissue regeneration in humans.[17–18] Lang et al.[19,20] demonstrated that for the regeneration of jaw defects in humans it is necessary to have an undisturbed healing period of at least 6 months, and that an earlier membrane removal produced a lower bone regeneration. In our case the fact that the membrane was removed, because of the continuing presence of infection, earlier than the 6 months period was certainly responsible for the lack of success in bone regeneration. In our patient, with all probability, the presence of pathogenic bacteria in the pathological tissues not removed from the apical portion of the implant, where the microscopical aspect of empty osteocyte lacunae, nonviable bone trabeculae and areas of resorption indicated death of bone, probably prevented the formation of new bone and produced the eventual loss of the implant.

Acknowledgments
This work was partially supported by the National Research Council (C.N.R.) and by the Ministry of University, Research, Science, and Technology (M.U.R.S.T.).

REFERENCES

1. Balshi TJ, Pappas CE, Wolfinger GJ, Hernandez RE. Management of an abscess around the apex of a mandibular root form implant: Clinical report. *Implant Dent* 1994;3:81–85.
2. Meffert RM. Periodontitis and peri-implantitis: one in (sic) the same? *Pract Periodontics and Aesthetic Dent* 1993;5:79–82.
3. Meffert RM. How to treat ailing and failing implants. *Implant Dent* 1992;1:25–33.
4. Meffert RM, Langer B, Fritz ME. Dental implants: a review. *J Periodontol* 1992;63:859–870.
5. Donath K, Breuner G. A method for the study of undecalcified bones and teeth with attached tissues. *J Oral Pathol* 1982;11:318–326.
6. Goldman MJ. Bone regeneration around a failing implant using guided tissue regeneration. A case report. *J Periodontol* 1992;63: 473–476.
7. Zarb GA. Osseointegration: A requiem for the periodontal ligament? *Int J Periodontics Restorative Dent* 1991;11:88–91.
8. Mombelli A, Van Oosten MAC, Scurch E, Lang N. The microbiota associated with successful or failing osseointegrated titanium implants. *Oral Microbiol Immunol* 1987;2:145–151.
9. Rosenberg ES, Torosian JP, Slots J. Microbial differences in 2 clinically distinct types of failures of osseointegrated implants. *Clin Oral Impl Res* 1991;2:135–144.
10. Quirynen M, Listgarten MA. The distribution of bacterial morphotypes around natural teeth and titanium implants ad modum Branemark. *Clin Oral Impl Res* 1990;1:8–12.
11. Apse P, Ellen RP, Overall CM, Zarb GA. Microbiota and crevicular fluid collagenase activity in the osseointegrated dental implant sulcus: a comparison of sites in edentulous and partially edentulous patients. *J Periodont Res* 1989;24:96–105.
12. Becker W, Becker BE, Newman MG, Nyman S. Clinical and microbiologic findings that may contribute to dental implant failure. *Int J Oral Maxillofac Implants* 1990;5:31–38.
13. Grunder U, Hurzeler MB, Schupbach P, Strub JR. Treatment of ligature-induced periimplantitis using guided tissue regeneration: a clinical and histological study in the beagle dog. *Int J Oral Maxillofac Implants* 1993;8:282–293.
14. Dahlin C, Sennerby L, Lekholm U, Linde A, Nyman S. Generation of new bone around titanium implants using a membrane technique: an experimental study in rabbits. *Int J Oral Maxillofac Implants* 1989;4:19–25.
15. Becker W, Becker BE, Handelsman M, Ochsenbein C, Albrektsson T. Guided tissue regeneration for implants placed into extraction sockets: A study in dogs. *J Periodontol* 1991;62:703–709.
16. Becker W, Becker BE. Guided tissue regeneration for implants placed into extraction sockets and for implant dehiscences: surgical techniques and case reports. *Int J Periodontics Restorative Dent* 1990;10:376–361.
17. Yukna RA. Clinical human comparison of expanded polytetrafluoroethylene barrier membrane and freeze-dried dura mater allografts for guided tissue regeneration of lost periodontal support. I. Mandibular molar class II furcations. *J Periodontol* 1992;63:431–442.
18. Fontana E, Trisi P, Piattelli A. Freeze-dried dura mater for guided tissue regeneration in post-extraction dental implants: A clinical and histologic study. *J Periodontol* 1994;65:658–665.
19. Lang NP, Bragger U, Hammerle CHF, Sutter F. Immediate transmucosal implants using the principles of guided tissue regeneration. I. Rationale, clinical procedures and 30-months results. *Clin Oral Implant Res* 1994;5:154–163.
20. Lang NP, Hammerle CHF, Bragger U, Lehmann B, Nyman SR. Guided tissue regeneration in jawbone defects prior to implant placement. *Clin Oral Implant Res* 1994;5:92–97.

Send reprint requests to: Dr. Adriano Piattelli, Via F. Sciucchi 63, 66100 Chieti, Italy.

Accepted for publication April 28, 1995.
