# Supplementary material for: A novel histopathological classification of implant periapical lesion: A systematic review and treatment decision tree
Source: PLoS One. 2022 Dec 22;17(12):e0277387. doi: 10.1371/journal.pone.0277387 (PMC9778521; doi:10.1371/journal.pone.0277387)
Supplement: S1 File — (ZIP) [file pone.0277387.s001.zip › support files/Included study/Piattelli 1998 (2).pdf]

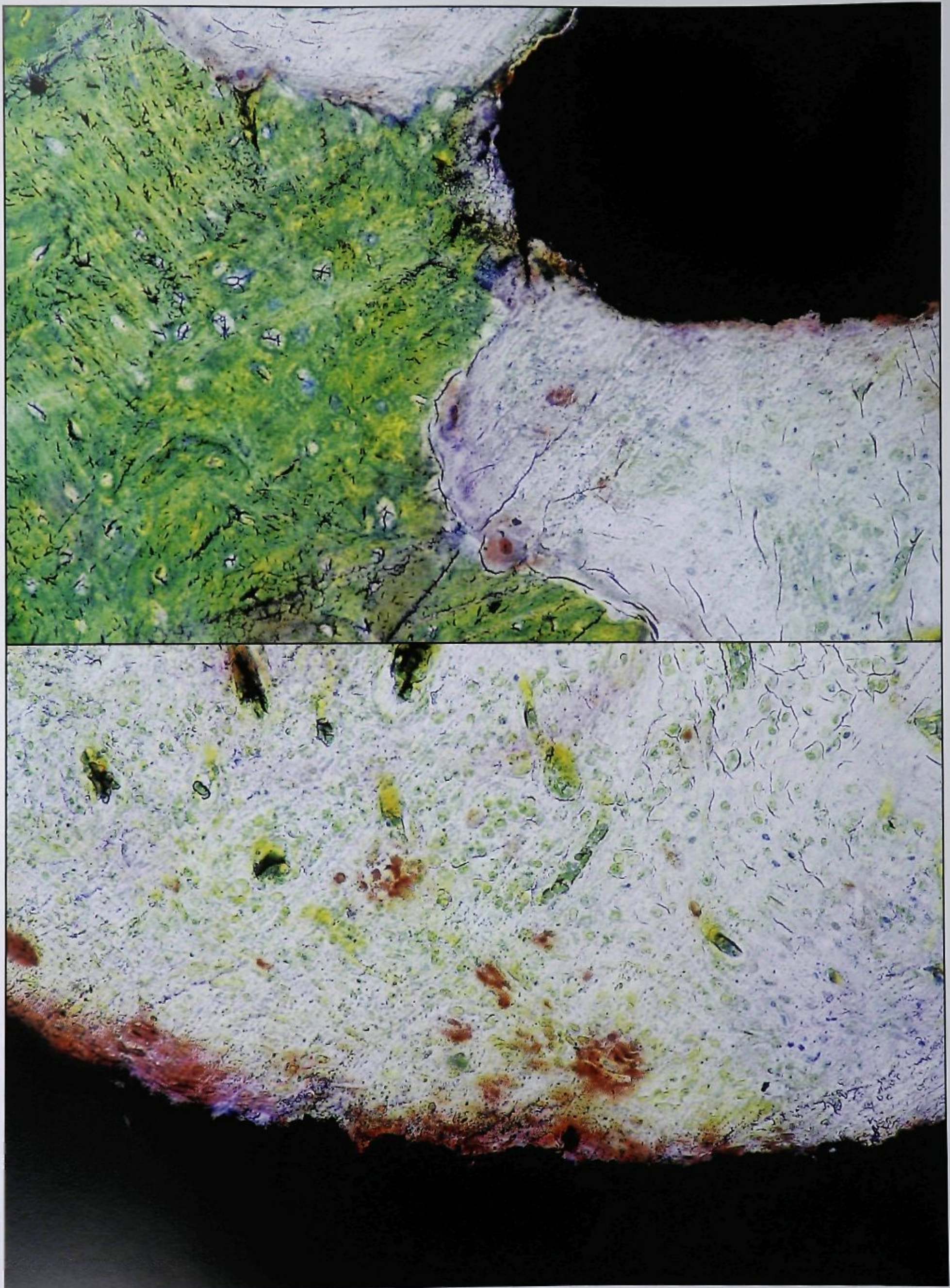

Implant Periapical Lesions: Clinical, Histologic, and Histochemical Aspects. A Case Report

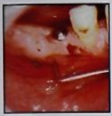

*Adriano Piattelli, MD, DDS**
*Antonio Scarano, DDS***
*Maurizio Piattelli, MD, DDS****
*Gianluca Podda, DDS*****

A new entity, the "implant periapical lesion," has recently been described. The etiology of this condition could be attributed to overheating of the bone, overloading of the implant, presence of a pre-existing infection or of residual root particles and foreign bodies in the bone, implant contamination during production or during insertion, or placement of the implant in an infected maxillary sinus. In this report, a titanium plasma-sprayed implant had been inserted into the mandible of a 53-year-old patient; after 5 months a fistula developed and periapical radiography showed a large radiolucent image around the apical portion of the implant. The implant was removed, and histologic examination showed necrotic bone and an inflammatory infiltrate inside the hollow portion of the implant. The etiology of the implant failure in this instance could be related to a fracture and vascular impairment of the bone inside the implant during insertion, to external contamination of the implant, or to the poor bone quality of the implant site. (Int J Periodont Rest Dent 1998;18:181–187.)

*Professor of Oral Medicine and Pathology, Dental School, University of Chieti, Italy; and Honorary Senior Lecturer, Eastman Dental Institute for Oral Health Care Sciences, London, United Kingdom.
**Research Fellow, Dental School, University of Chieti, Italy.
***Researcher, Dental School, University of Chieti, Italy.
****Research Fellow, Dental School, University of Rome, Italy.

Reprint requests: Prof Adriano Piattelli, Via F. Sciucchi 63, 66100 Chieti, Italy.

An infection located at the apex of an implant has been recently described[1,2] and has been defined by Reiser and Nevins[3] as an "implant periapical lesion." This complication may be caused by surgical trauma from overheating of the bone, overloading of the implant at an early stage resulting in the production of microfractures around the implant, or the presence of a pre-existing infection in the bone.[1,4] Other causes may be the presence of residual root particles and foreign bodies, the contamination of the implant by the manufacturer during production or by the clinician during insertion, or the placement of an implant in an infected maxillary sinus.[3]

Following is a proposed classification of dental implants with complications[5]:

1. Ailing implant: bone loss, pocket formation, the implant is static at maintenance checks

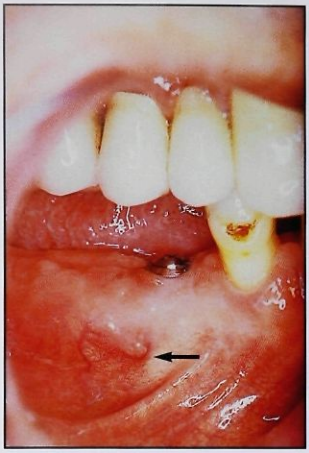

Fig 1 Fistula in the vestibular mucosa (arrow).

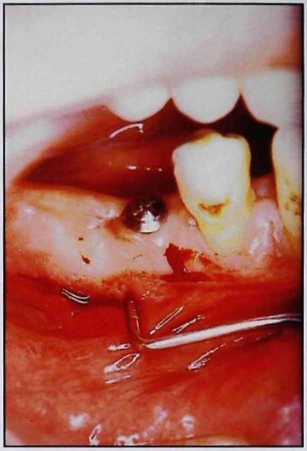

Fig 2 Metal can be reached with a periodontal probe.

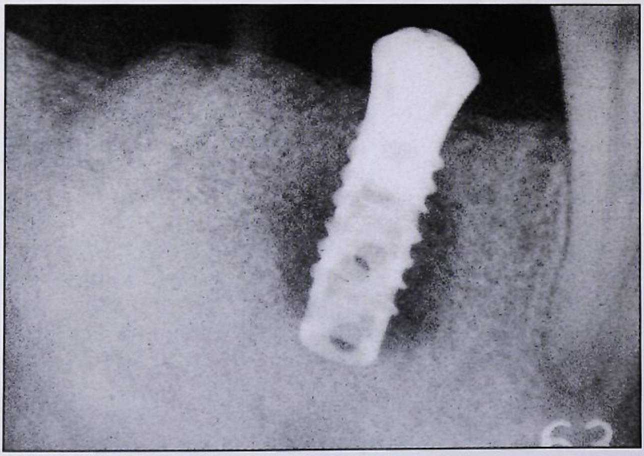

Fig 3 Periapical radiograph reveals a radiolucency that involves most of the apical portion of the implant.

2. Failing implant: bone loss notwithstanding therapy, bleeding on probing, purulent discharge
3. Failed implant: mobility, dull sound on percussion, peri-implant radiolucency

The first two types of implant complications may be subjected to treatment, while the failed implant should be extracted.[5,6]

The objective of the present case report was to evaluate the clinical, histologic, and histochemical findings in a implant with an implant periapical lesion.

Case report

A 53-year-old man underwent the insertion of two Bonefit implants (ITI) in the right posterior mandible: one implant was inserted into the premolar region and the other into the molar region. Panoramic and periapical radiographs showed no pre-existing bone pathology. The implants were not loaded, and 5 months after insertion a fistula was observed at the level of the premolar vestibular mucosa (Fig 1). The lesion had the discharge of a purulent exudate with swelling, redness, and pain. The other implant did not present any problems. A probe was inserted into the fistula, and it was possible to touch the metal surface with the tip of the

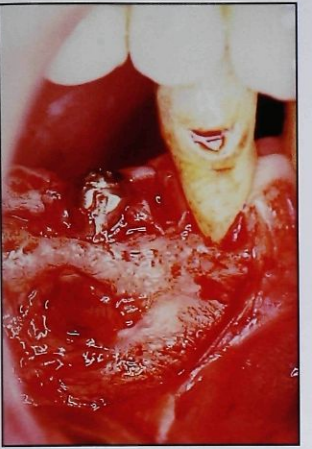

Fig 4 (left) *After elevation of the mucoperiosteal flap, the destruction of the mandibular vestibular plate and the presence of granulation tissue is evident.*

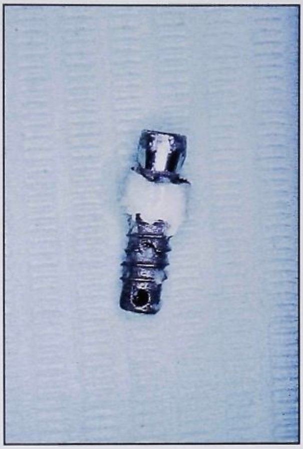

Fig 5 (right) *Implant removal reveals bone in close apposition with metal.*

probe (Fig 2). No vertical probing depth was noted. A periapical radiograph showed the presence of a wide osteolytic area around the apex and the middle portion of the implant (Fig 3). A systemic antibiotic treatment with metronidazole and a local treatment with Actisite fibers (Alza) was instituted, but the symptoms continued unabated. A mucoperiosteal flap was elevated, and a fenestration, with the apical portion of the implant embedded in granulation tissue, was observed (Fig 4). The implant was removed with a trephine bur (Fig 5).

The specimen was immediately fixed in 10% buffered formalin and processed to obtain thin ground sections with the Precise 1 Automated System (Assing).[7] In short, the specimen was dehydrated in an ascending series of alcohol and embedded in a glycolmethacrylate resin (Technovit 7200 VLC, Kulzer). After polymerization, the specimen was sectioned with a high-precision diamond disk at a thickness of about 150 μm and ground down to about 30 μm. After polishing, the slides were stained with acid fuchsin–toluidine blue and observed under normal light in a Leitz Laborlux microscope (Leitz). The histochemical staining for alkaline and acid phosphatases was done according to a technique already described.[8]

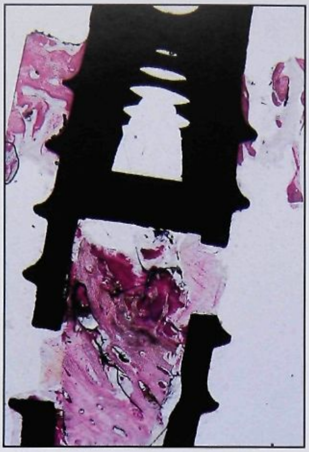

Fig 6 At the more coronal aspect, compact vital bone is observed to be in direct contact with the implant. The hollow portion shows evidence of necrotic bone. (Original magnification × 30; acid fuchsin–toluidine blue stain.)

Results

At low power the implant was seen to be in contact with vital bone in the most coronal 2 mm, while at the more apical portion bone was absent (Fig 6). The hollow portion of the implant was largely filled by bone and inflammatory tissue with an absence of vascular structures. The inflammatory cell infiltrate showed a prevalence of macrophages and lymphocytes, with plasma cells and granulocytes also in evidence to a lesser extent (Fig 7). At high power the bone that was observed in direct contact with the implant showed evidence of mature osteons in which bacteria, osteoclasts, and inflammatory cells were absent. In one field, however, some inactive osteoblasts were visible. In this zone there was no empty space visible between the implant and the bone. The hollow portion of the implant was filled with bone that, under very high magnification, showed evidence of empty osteocytic lacunae.

This bone showed all the characteristics of a bony sequestrum. Near this bone many lymphocytes were present (Fig 8). Furthermore, small fragments of this sequestrum could be seen breaking away and migrating toward the external part of the implant. These fragments, 0.2 mm in size, appeared to provoke a foreign body reaction. The histochemical analysis for alkaline phosphatase showed a complete absence of actively secreting osteoblasts, but the staining for acid phosphatase (ACP) showed a notable macrophage or osteoclast activity. At the external aspect of the apical portion of the implant no inflammatory cell infiltrate was observed, although active ACP-positive osteoclasts were observed. In many areas Howship's lacunae were evident with their corresponding osteoclasts (staining positive for ACP) actively carrying out bone resorption (Fig 9). Some positively stained macrophages were in direct contact with the implant surface (Fig 10), but others were evident within the inflamed tissue. The pathologic diagnosis was of bone aseptic necrosis with the presence of a bone sequestrum (of the bone inside the hollow portion of the implant).

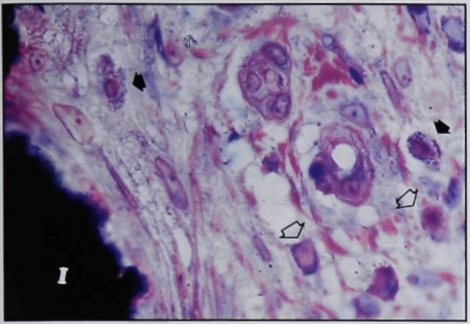

Fig 7 *At higher magnification, numerous granulocytes (arrows) and lymphocytes (white arrows) are observed. No bacteria are present. I = implant. (Original magnification × 1200; acid fuchsin–toluidine blue stain.)*

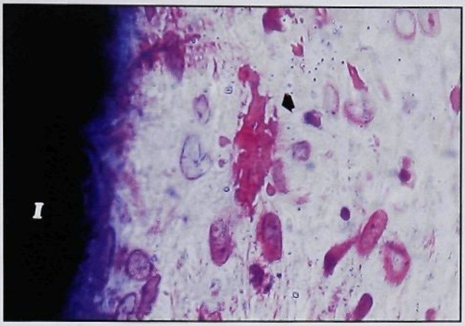

Fig 8 *Small bony sequestrum (arrow) lying free in close proximity to the implant (I).(Original magnification × 1200; acid fuchsin–toluidine blue stain.)*

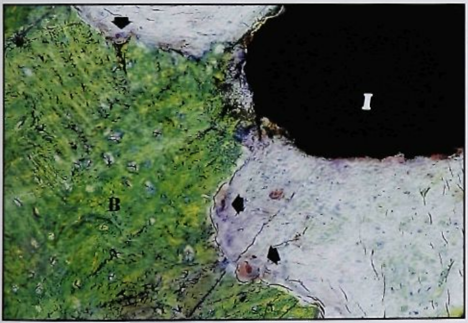

Fig 9 *Apical portion of the implant (I). Howship's lacunae and their corresponding osteoclasts are visible at the external aspect (arrows). The osteoclasts are stained positive for ACP. B = bone. (Original magnification × 100; ACP counterstained with toluidine blue.)*

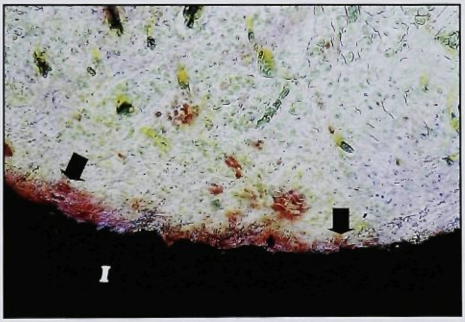

Fig 10 *High-power view of the hollow portion of the implant. Numerous macrophages, which are positive for ACP (arrows), are seen in contact with the implant surface (I). (Original magnification × 100; ACP counterstained with toluidine blue.)*

Discussion

Lekholm et al[9] reported a cumulative implant failure rate of 6.7% after 5 years of functional loading. Implant failures have been reported to result from premature loading by overlying dentures, occlusal overload, infection in the mouth, bone overheating, bacterial colonization, operator error, or bone of insufficient quality and/or quantity.[1,4,5,10] In the present case, the presence of a bone aseptic necrosis inside the hollow part of the implant could have been the origination point that led to the ultimate loss of the implant. This necrosis may have been produced either by a fracture of the bone inside the hollow portion during implant insertion with subsequent vascular impairment, or by bone overheating during surgery. Another explanation for the occurrence of the infection in the present case could be a contamination of the implant surface either during production or during insertion. This possibility, however, is probably contradicted by the presence of activated macrophages in only a few areas of the implant perimeter and not on the whole surface of the titanium. Moreover, the fact that the implant had been inserted in a location in which the bone quality was poor must be considered and can help in explaining the failure of the implant to osseointegrate because of the scarcity of osteoprogenitor cells. On the other hand, the possibility that the lesion resulted from a pre-existing bone infection in the implant site can be excluded because the preoperative radiographs were negative. Premature overloading can also be excluded because the implant had not been loaded. Mellonig et al[11] divide implant failures into infectious failure (peri-implantitis) and traumatic failure (retrograde peri-implantitis). It is still not certain whether an implant periapical lesion is composed of healthy tissue, new tissue destruction, or activation of a pre-existing condition.[3] Implant failures are characterized by mobility, marginal swelling and redness, bleeding and/or suppuration on probing, increased probing depth, peri-implant radiolucencies, and alveolar bone height loss.[12]

For teeth with peri-implantitis, it has been suggested that remaining natural teeth act as a reservoir of bacteria for involvement with the peri-implant tissues.[13] In partially edentulous patients, the degree of marginal inflammation processes appears to be similar around teeth and implants,[14] and pathogens usually seen in periodontal disease have been isolated from failing implants.[15] Rosenberg et al[12] found that a high proportion of spirochetes and motile rods were observed in implants that failed because of infection. Treatment of an infection at the apex of an implant can be very difficult. An intraoral transmandibular approach, an intraoral periosteal dissection, and an extraoral approach have been proposed for mandibular implants.[1] A thorough debridement of the infected site must be executed with complete elimination of all inflamed granulation tissue to obtain a resolution of the pathologic processes. In some instances it is necessary to remove part of the implant to execute a complete toilette of the affected tissue. In this respect, Reiser and Nevins[3] reported the case of an implant with a radiolucency in a periapical location in which a resection of the most apical part of the implant was carried out successfully. Additional data are certainly necessary for a more comprehensive understanding of the etiopathologic and clinical problems related to the implant periapical lesion.

Acknowledgments

This work was partially supported by the National Research Council (CNR), Rome, Italy, and by the Ministry of University, Research, Science, and Technology (MURST), Rome, Italy.

References

1. Balshi TJ, Pappas Ce, Wolfinger GJ, Hernandez RE. Management of an abscess around the apex of a mandibular root form implant: Clinical report. Implant Dent 1994;3:81–85.
2. Piattelli A, Scarano M, Piattelli M. Abscess formation around the apex of a maxillary root form implant: Clinical and microscopical aspects. A case report. J Periodontol 1995;66:899–903.
3. Reiser GM, Nevins M. The implant periapical lesion: Etiology, prevention and treatment. Compendium 1995;16:768–777.
4. Meffert RM. Periodontitis and periimplantitis: One in (sic) the same? Pract Periodontics Aesthet Dent 1993;5:79–82.
5. Meffert RM. How to treat ailing and failing implants. Implant Dent 1992;1:25–33.
6. Meffert RM, Langer B, Fritz ME. Dental implants: A review. J Periodontol 1992;63:859–870.
7. Piattelli A, Scarano A, Quaranta M. A new high-precision, cost-effective cutting system for producing thin sections of oral tissues containing dental implants. Biomaterials 1997;18:577–579.
8. Piattelli A, Scarano A, Piattelli M. Detection of alkaline and acid phosphatases around titanium implants: A light microscopical and histochemical study in rabbits. Biomaterials 1995;16:1333–1338.
9. Lekholm U, van Steenberghe D, Herrmann I, Bolender C, Former T, Gunne J, et al. Osseointegrated implants in the treatment of partially edentulous jaws: A prospective 5-year multicenter study. Int J Oral Maxillofac Implants 1994;9:627–635.
10. Jaffin RA, Berman CL. The excessive loss of Brånemark fixtures in type IV bone: A 5-year analysis. J Periodontol 1991;62:2–4.
11. Mellonig JT, Griffiths G, Mathys E, Spitznagel J. Treatment of the failing implant: Case reports. Int J Periodont Rest Dent 1995;15:385–395.
12. Rosenberg ES, Torosian JP, Slots J. Microbial differences in 2 clinically distinct types of failures of osseointegrated implants. Clin Oral Implants Res 1991;2:135–144.
13. Quirynen M, Listgarten MA The distribution of bacterial morphotypes around natural teeth and titanium implants ad modum Brånemark. Clin Oral Implants Res 1990;1:8–12.
14. Apse P, Ellen RP, Overall CM, Zarb GA. Microbiota and crevicular fluid collagenase activity in the osseointegrated dental implant sulcus: A comparison of sites in edentulous and partially edentuluos patients. J Periodont Res 1989;24:96–105.
15. Becker W, Becker BE, Newman MG, Nyman S. Clinical and microbiologic findings that may contribute to dental implant failure. Int J Oral Maxillofacial Implants 1990;5:31–38.
