# Supplementary material for: A novel histopathological classification of implant periapical lesion: A systematic review and treatment decision tree
Source: PLoS One. 2022 Dec 22;17(12):e0277387. doi: 10.1371/journal.pone.0277387 (PMC9778521; doi:10.1371/journal.pone.0277387)
Supplement: S1 File — (ZIP) [file pone.0277387.s001.zip › support files/Included study/piattelli1998 (1).pdf]

Clinical and Histologic Evaluation of an Active "Implant Periapical Lesion": A Case Report

Adriano Piattelli, MD, DDS*/Antonio Scarano, DDS**/Piero Balleri, MD, DDS***/
Gian Antonio Favero, MD, DDS****

A new entity called "implant periapical lesion" has recently been described. This lesion could be the result of, for example, bone overheating, implant overloading, presence of a preexisting infection or residual root fragments and foreign bodies in the bone, contamination of the implant, or implant placement in an infected maxillary sinus. This case report describes a titanium implant that was placed in the maxillary premolar region. A fenestration involving the middle portion of the implant was present. After 7 months, the apical portion of the implant showed radiolucency. This lesion rapidly increased in size and a vestibular fistula appeared. A systemic course of antibiotics was not successful, and the implant was then removed. The histologic examination showed the presence of necrotic bone inside the antirotational hole of the implant. The etiology of the implant failure in this instance could possibly be related to bone overheating associated with an excessive tightening of the implant and compression of the bone chips inside the apical hole, producing subsequent necrosis.
(Int J Oral Maxillofac Implants 1998;13:713–716)

Key words: bone necrosis, implant failure, titanium implant

Reiser and Nevins[1] have recently described an infection located at the apex of an implant, which they defined as an "implant periapical lesion." This complication may cause several problems including loss of the implant, and its etiology may be related to bone overheating, implant overloading with peri-implant microfractures, or the presence of preexisting infections in the bone.[1] The presence of residual root particles and foreign bodies, implant contamination during production or placement, or implant placement in an infected maxillary sinus may also cause this lesion.[1] A proposed classification of dental implant complications is as follows[2]:

1. Compromised successful implant: presence of inflammation, hyperplasia, and fistula formation near a successfully osseointegrated implant
2. Failing implant: progressive bone loss in a functional implant
3. Failed implant: infection around a compromised implant

Implant periapical lesions have been considered inactive or infected lesions.[1] The inactive lesion may be similar to the periapical scar found at a tooth apex and characteristically presents no clinical symptomatology, while the infected lesion is usually accompanied by fistula development.[1]

The aim of this case report is to present an analysis of the clinical, histologic, and histochemical features of a maxillary implant periapical lesion.

*Professor of Oral Medicine and Pathology, Dean, Dental School, University of Chieti, Chieti, Italy; Honorary Senior Lecturer, Eastman Dental Institute for Oral Health Care Sciences, London, United Kingdom.
**Research Fellow, Dental School, University of Chieti, Chieti, Italy.
***Private Practice, Lamporecchio (Pistoia), Italy.
****Professor, Dental School, University of Padua, Padua, Italy.

Reprint requests: *Prof Adriano Piattelli, Via F. Sciucchi 63, 66100 Chieti, Italy. E-mail: apiattelli@unich.it*

Copyright © 2000 by Quintessence Publishing Co, Inc. Printing of this document is restricted to personal use only. No part of this article may be reproduced or transmitted in any form without written permission from the publisher.

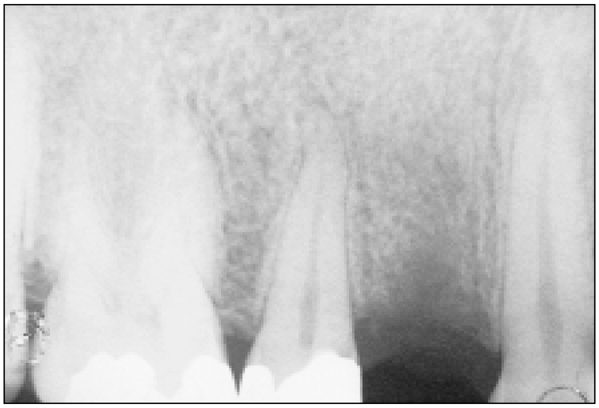

Fig 1 Pretreatment radiograph demonstrating that no preexisting bone pathology is present.

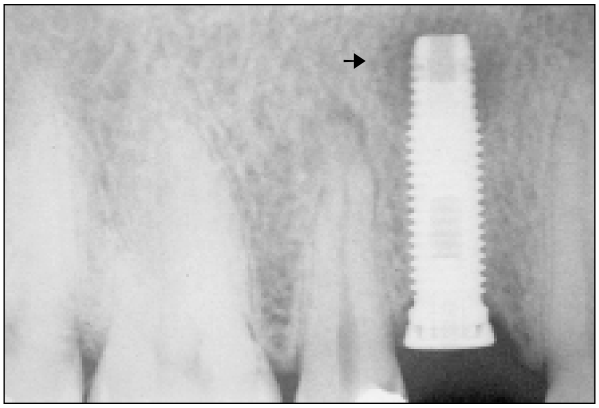

Fig 2 After 7 months, a radiolucency is present around the apical portion of the implant *(arrow)*. A periapical lucency is present at the apex of the second premolar.

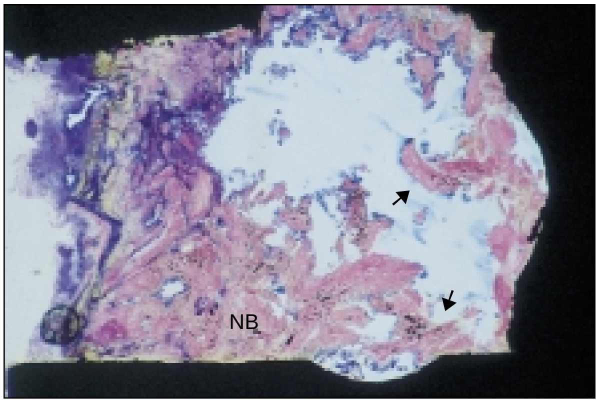

Fig 3 Necrotic bone (NB) with empty osteocyte lacunae *(arrows)* is present inside the antirotational hole of the implant (acid fuchsin–toluidine blue; original magnification × 50).

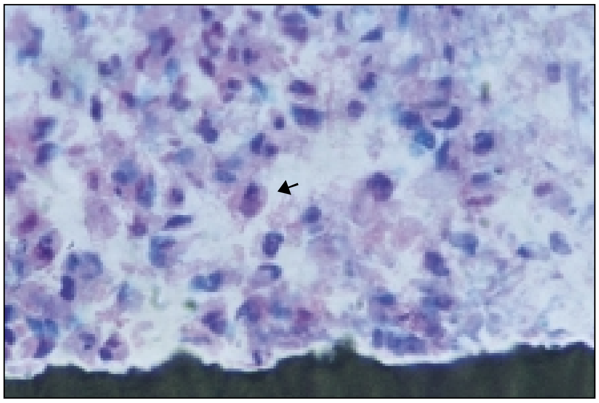

Fig 4 Many granulocytes *(arrow)* are present near the implant surface (acid fuchsin–toluidine blue; original magnification × 400).

Case Report

A 24-year-old female underwent the placement of one 3.75 mm × 15 mm Brånemark implant (Nobel Biocare, Göteborg, Sweden) in the premolar region of the maxilla (right first premolar). The tooth had been extracted 2 months previously because of nonrestorable caries. A periapical radiograph showed no preexisting bone pathology (Fig 1). After implant placement, it was possible to see a fenestration involving three threads in the middle portion of the implant. This fenestration was treated with a bovine cartilage membrane (New Bone, Martina, Italy).

Seven months later, a radiolucence was present at the apical portion of the implant (Fig 2); it was also possible to observe the presence of a periapical lucency at the apex of the second premolar. After another month, the lesion showed a conspicuous increase in size, and a vestibular fistula appeared. Systemic antibiotic treatment with metronidazole was instituted, but the symptoms continued unabated. A decision was made to remove the implant with a trephine bur.

The specimen was immediately fixed in 10% buffered formalin and processed with the Precise 1 Automated System (Assing, Rome, Italy) to obtain thin ground sections.[3] The specimen was dehydrated in an ascending series of alcohols and embedded in a glycolmethacrylate resin (Technovit 7200 VLC, Kulzer, Wehrheim, Germany). After polymerization, the specimen was sectioned with a high-precision diamond disc at a thickness of about 150 µm and ground to about 30 µm. After polishing, the slides were stained with acid fuchsin and toluidine blue and observed under normal light in a Leitz Laborlux microscope (Leitz, Wetzlar, Germany). The histo-

COPYRIGHT © 2000 BY QUINTESSENCE PUBLISHING CO, INC. PRINTING OF THIS DOCUMENT IS RESTRICTED TO PERSONAL USE ONLY. NO PART OF THIS ARTICLE MAY BE REPRODUCED OR TRANSMITTED IN ANY FORM WITHOUT WRITTEN PERMISSION FROM THE PUBLISHER.

chemical staining for alkaline and acid phosphatase was performed according to a technique previously described.[4]

Results

At low-power magnification, necrotic bone was observed inside the antirotational hole; all of the osteocyte lacunae were empty (Fig 3). Pyknotic cells were present in some areas between the necrotic bone trabeculae. The bone trabeculae appeared to be compressed, and some of them had undergone demineralization. No capillaries, osteoblasts, or epithelial cells were present. In the most apical portion, it was possible to observe many lymphocytes surrounding the necrotic bone. No multinucleated giant cells, macrophages, or osteoclasts were present. Plaque and granulocytes were observed near the implant surface (Fig 4). All other parts of the implant surface were surrounded by vital, compact, mature bone. The histochemical analysis for alkaline and acid phosphatases showed that no positive cells were present.

Discussion

Complications in implant dentistry can occur at any stage.[5] Analysis of failed implants is invaluable in preventing unnecessary failures and evaluating treatment outcomes.[6] Lekholm et al[7] reported a cumulative implant failure rate of 6.7% after 5 years of functional loading. The loss of anchorage can be the result of surgical trauma, contamination, or overload.[5] Failure to osseointegrate may be caused by overinstrumentation of the bone, producing inadequate implant immobilization.[8] Initial implant instability can be the result of poor bone-tapping technique, excessive countersinking, misinterpretation of bone quality,[4] or possibly inadequate implant length. Mellonig et al[9] categorize implant failures as infectious failure (peri-implantitis) and traumatic failure (retrograde peri-implantitis). It has been suggested that implant periapical lesions arise from a contaminated implant placed in a site with necrotic bone.[1] In the present case, the clinical and histologic features could be analyzed in the following manner:

1. *Contamination of the implant surface.* This hypothesis cannot be confirmed because the radiographic and histologic analysis showed that the major portion of the implant was surrounded by vital, mature, compact bone. Moreover, the histochemical analysis showed that no acid phosphatase–positive cells were present, and so no activated macrophages were observed. It is therefore most unlikely that only the antirotational hole region had been contaminated.

2. *Fenestration of the vestibular bone.* A fenestration of the vestibular bone had been present in the middle portion of the implant during the surgical placement; however, it is unlikely that a perforation could have determined necrosis of only the apical bone.

3. *Bone overheating during surgery.* Some of the histologic features could point to the occurrence of bone overheating during implant placement.

4. *Excessive tightening of the implant with compression of the bone chips.* An excessive in-depth positioning of the implant could have caused compression of the bone chips produced during the bone site preparation, which in turn could have resulted in ischemia, necrosis, and formation of a bone sequestrum. The histologic presence of compressed bone trabeculae and absence of blood vessels could favor this hypothesis.

5. *Presence of preexisting bone pathology.* The periapical radiograph taken before the implant was placed showed no preexisting bone pathology.

6. *Overloading of the implant.* The implant had not been loaded.

7. *Poor quality of the bone site.* The scarcity of osteoprogenitor cells as a result of poor bone quality at the surgical site can probably be considered to have exerted a negative influence on the osseointegration of the implant.

In this patient, the clinical history, radiographic analysis, and histologic examination suggest that the combination of bone overheating and bone chip compression during implant placement, together with poor bone quality at the surgical site, are the most likely causes of the periapical pathology. The periapical lucency at the apex of the second premolar was in all probability not relevant in the etiology of the implant periapical lesion. Thus it is extremely important to use minimally traumatic surgery and to carefully debride the surgical bone site to remove all the bone chips produced during surgery so as to minimize the risk of compression necrosis of these bone chips during implant placement.

Summary

It is still not certain whether the new entity known as "implant periapical lesion" involves healthy tissue, new tissue destruction, or activation of a preexisting condition.[1] Mobility, marginal swelling and redness, bleeding and/or suppuration on probing, increased probing depth, peri-implant radiolucencies, and loss of alveolar bone height characterize implant failures.[10] The remaining natural teeth as well as the peri-implant tissues can act as a reservoir for bacteria.[11] Treatment of a periapical implant lesion can be difficult. Thorough

COPYRIGHT © 2000 BY QUINTESSENCE PUBLISHING CO, INC. PRINTING OF THIS DOCUMENT IS RESTRICTED TO PERSONAL USE ONLY. NO PART OF THIS ARTICLE MAY BE REPRODUCED OR TRANSMITTED IN ANY FORM WITHOUT WRITTEN PERMISSION FROM THE PUBLISHER.

curettage of the infected site with complete removal of all granulation tissues must be attained. Some instances could require removal of part of the implant to facilitate complete debridement of the affected tissues. Additional data are needed to provide complete understanding of the idiopathic and clinical problems of the "implant periapical lesion."[12,13]

Acknowledgments

This work was partially supported by the National Research Council (CNR) and by the Ministry of University, Research, Science, and Technology (MURST).

References

1. Reiser GM, Nevins M. The implant periapical lesion: Etiology, prevention and treatment. Compend Contin Educ Dent 1995;16;768–777.
2. Newman MG, Flemmig TF. Bacterial-host interaction. In: Worthington P, Brånemark PI (eds). Advanced Osseointegration Surgery. Berlin: Quintessence, 1992.
3. Piattelli A, Scarano A, Quaranta M. High-precision cost-effective system for producing thin sections of oral tissues containing dental implants. Biomaterials 1997;18:577–579.
4. Piattelli A, Scarano A, Piattelli M. Detection of alkaline and acid phosphatases around titanium implants: A light microscopical and histochemical study in rabbit. Biomaterials 1995;16:1333–1338.
5. Tolman DE, Laney WR. Tissue-integrated prosthesis complications. Int J Oral Maxillofac Implants 1992;7:477–484.
6. Takeshita F, Suetsugu T, Higuchi Y, Oishi M. Histologic study of failed hollow implants. Int J Oral Maxillofac Implants 1996;11:246–250.
7. Lekholm U, van Steenberghe D, Herrman I, Bolender C, Former T, Gunne J, et al. Osseointegrated implants in the treatment of partially edentulous jaws: A prospective 5-year multicenter study. Int J Oral Maxillofac Implants 1994;9:627–635.
8. Zarb GA, Schmitt A. The longitudinal clinical effectiveness of osseointegrated implants: The Toronto study. Part I: Surgical results. J Prosthet Dent 1990;63:451–457.
9. Mellonig JT, Griffiths G, Mathys E, Spitznagel J. Treatment of the failing implant: Case reports. Int J Periodont Rest Dent 1995;15:385–395.
10. Rosenberg ES, Torosian JP, Slots J. Microbial differences in 2 clinically distinct types of failures of osseointegrated implants. Clin Oral Implants Res 1991;2:135–144.
11. Quirynen M, Listgarten MA. The distribution of bacterial morphotypes around natural teeth and titanium implants ad modum Brånemark. Clin Oral Implants Res 1990;1:8–12.
12. Balshi TJ, Pappas CE, Wolfinger GJ, Hernandez RE. Management of an abscess around the apex of a mandibular root form implant: Clinical report. Implant Dent 1994;3:81–85.
13. Piattelli A, Scarano M, Piattelli M. Abscess formation around the apex of a maxillary root form implant: Clinical and microscopical aspects. A case report. J Periodontol 1995;66:899–903.

COPYRIGHT © 2000 BY QUINTESSENCE PUBLISHING CO, INC. PRINTING OF THIS DOCUMENT IS RESTRICTED TO PERSONAL USE ONLY. NO PART OF THIS ARTICLE MAY BE REPRODUCED OR TRANSMITTED IN ANY FORM WITHOUT WRITTEN PERMISSION FROM THE PUBLISHER.
