# Supplementary material for: A novel histopathological classification of implant periapical lesion: A systematic review and treatment decision tree
Source: PLoS One. 2022 Dec 22;17(12):e0277387. doi: 10.1371/journal.pone.0277387 (PMC9778521; doi:10.1371/journal.pone.0277387)
Supplement: S1 File — (ZIP) [file pone.0277387.s001.zip › support files/Included study/Pistilli 2020.pdf]

Original Contributions

Clinical Dentistry

Retrograde peri-implantitis associated with residual cysts

3 Case reports

Roberto Pistilli, MD; Luigi Canullo, MDS; Maria Menini, DDS, PhD; Valeria Pistilli, DDS; Giampiero Rossi-Fedele, DDS, MClinDent, PhD; Paolo Pesce, DDS, PhD

ABSTRACT

Background and Overview. The authors describe 3 cases of retrograde peri-implantitis associated with residual cysts and their successful surgical and regenerative management. Implants were originally placed with both immediate and delayed protocols.

Case Description. Three patients sought treatment at the authors' specialist private practices with the chief symptoms of pain and swelling in association with implant-retained restorations. Clinical, radiographic, and histopathologic findings supported the diagnoses of infected residual cysts. The lesions were enucleated in toto, the implant surfaces were decontaminated, and bone regeneration procedures were carried out. Long-term recall visits, ranging from 3 through 10 years, confirmed successful retention of all implants treated accordingly.

Conclusions and Practical Implications. The authors describe an unusual clinical manifestation and emphasize the role of local procedures during implant placement in areas compromised with infection, cysts, or both and the importance of long-term recall visits.

Key Words. Apical periodontitis; dental implant; residual cyst; peri-implantitis; case report.

JADA 2020:■(■):■-■
https://doi.org/10.1016/j.adaj.2020.07.016

Dental implants are used commonly for the rehabilitation of edentulous areas as an alternative to fixed and removable prosthodontics.[1,2] Frequently reported reasons for tooth extraction include caries with its sequelae, including apical periodontitis and periodontal disease.[3-5] Granulatory tissues in chronic infection of endodontic or periodontal origin contain a relatively high proportion of epithelial tissue,[6] which can proliferate in the presence of inflammation, with subsequent formation of cysts.[7,8] On formation, true cysts are considered self-sustaining[8] and therefore can persist after extraction of the causative tooth, leading to the establishment of residual cysts in the edentulous area of the jaw.[8] Residual cysts commonly have slow growth and progressive and painless expansion, which appears clinically long after tooth extraction when becoming conspicuous or infected.[8] Residual cysts are a common cause of intraoral swelling in the elderly.[8]

The presence of residual cysts can be associated with long-term complications after implant placement and in the development of so-called "retrograde peri-implantitis."[9,10] The literature on timely consecutive retrograde peri-implantitis (that is, after tooth extraction and placement of an implant fixture in a compromised site)[10] with confirmed histopathologic diagnosis of cyst is scarce. In a study published in 2020, researchers reported a granuloma as the cause of an endodontic-periodontal defect.[11] This type of observational study allows for the acquisition of knowledge and information about rare diseases or conditions and the generation of hypotheses and research questions about clinically relevant issues, which provide an important educational tool and clinical resource.[12] The purpose of our case report is to present the diagnosis and management of 3 cases of retrograde peri-implantitis associated with residual cysts.

Copyright © 2020 American Dental Association. All rights reserved.

ARTICLE IN PRESS

CASE PRESENTATION

Case 1

A 28-year-old woman with unremarkable medical history was treated previously with a dental implant in the region of tooth no. 7. She stated that 4 years earlier, tooth no. 7 was extracted because it was deemed unsalvageable owing to fracture and a postextraction immediately restored implant was placed. On consultation at our office, she reported pain arising from the area, and swelling was present on clinical examination of the implant site. Radiographic examination (Figure 1) revealed a round, unilocular, well-defined, and corticated radiolucent lesion in the area, which was confirmed subsequently with computed tomography. Extensive bone loss compromising the buccal bone plate at the region of teeth nos. 7 and 8 was present (Figure 1). Clinically, the implant was stable, with no mobility.

Case 2

A 25-year-old man who smoked but had no additional relevant medical history was treated in a different surgery with a dental implant in the region of tooth no. 19 after unsuccessful endodontic treatment, which the presence of 2 separated, round, unilocular radiolucent areas in the periapical region suggested (Figure 2A). Radiographic examination 3 months after immediate implant placement found a reduction in the size of the radiolucency and the presence of radiopaque material in the lesion, consistent with previously extruded root canal filling material (Figure 2B). One year after placement, the patient had pain during a consultation at our office and radiographic examination of the area using periapical radiography and cone-beam computed tomography revealed an increase in size of the radiolucency (Figures 2C and 2D). Clinically, the implant was stable with no mobility.

Case 3

A 64-year-old man who smoked but had an otherwise noncontributory medical history had chief symptoms of pain and swelling in relation to the mandibular right edentulous alveolar ridge. The patient reported that the teeth were extracted 3 years earlier when the tooth-supported prosthesis failed, and that 3 months after the extractions the implants were placed and "allowed to heal." Subsequently, the patient underwent the second surgery and implant-related prosthetic procedures. Panoramic radiographic examination (Figure 3) showed an oval-shaped, unilocular, well-defined radiolucency associated with the implant in distal position in proximity to the inferior alveolar nerve. Both implants were stable clinically with no mobility.

Cyst enucleation

The patients received systemic antibiotic treatment with amoxicillin and clavulanic acid for 1 week before surgery. We opted for intrasulcular flap incisions with papilla disepithelization to expose the lesions. A mucoperiosteal flap was raised, the residual buccal cortical bone was removed as required, and the lesions were located. Once exposed, the lesions were removed

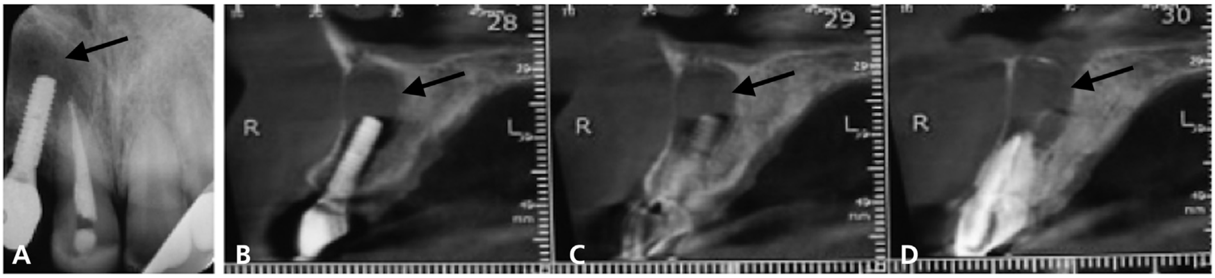

Figure 1. Case 1. Intraoral radiographic and cone-beam computed tomographic images showing a radiolucency in the region of tooth no. 7 (arrows) at 4 years after implant insertion. Periapical radiograph (**A**), cone-beam computed tomography sagittal slices (**B,C,D**).

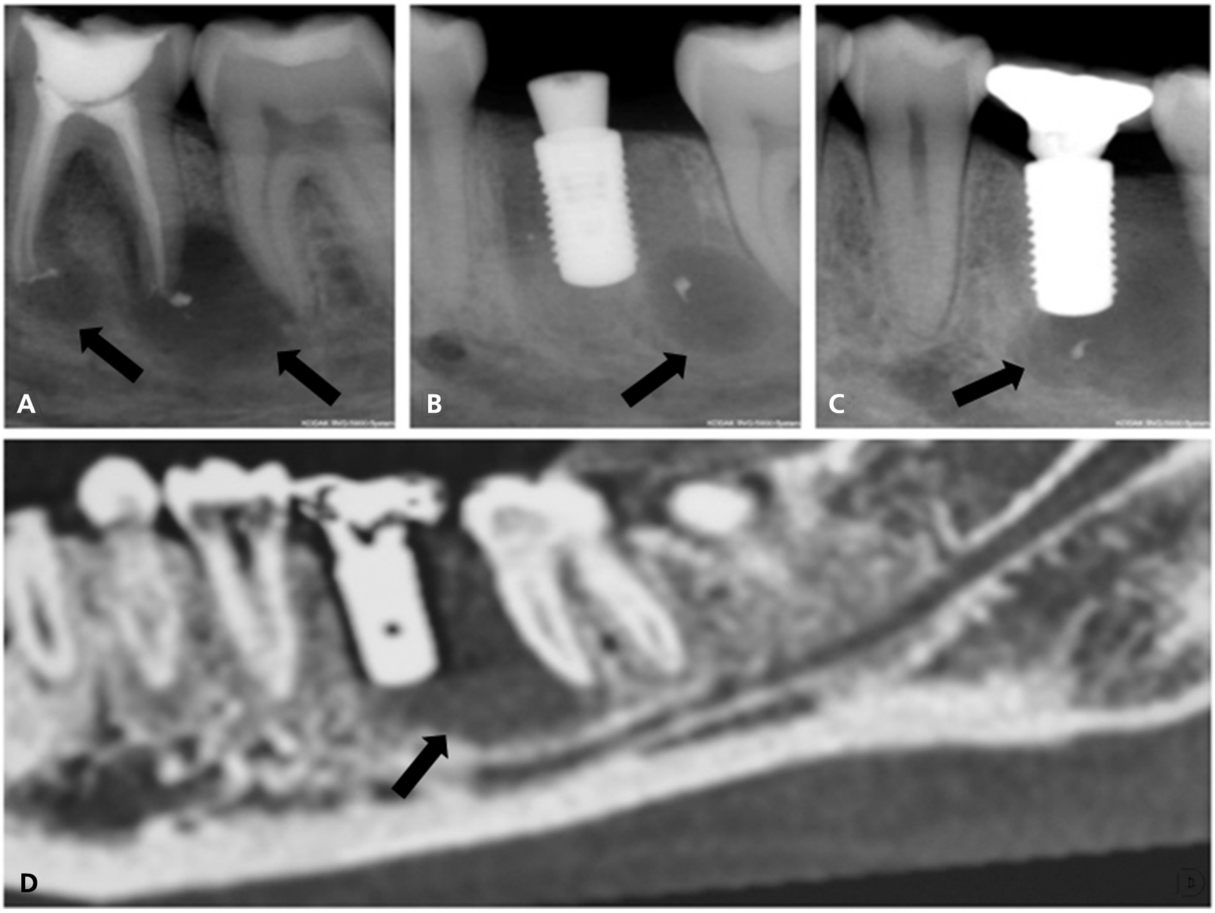

Figure 2. Case 2. Intraoral radiography and cone-beam computed tomographic images showing tooth no. 19 associated with 2 reasonable well-defined areas of bone destruction (arrows) (**A**), implant in the region of tooth no. 19 at 3 months after implant placement in contiguity with bone destructions (arrow) (**B**), and implant in the region of tooth no. 19 at 1 year after implant placement in contiguity with bone destructions (arrows) (**C, D**).

from the crypt, measured using a probe, and sent in toto in fixative for histopathologic examination. The crypts were meticulously curetted, avoiding damage to anatomic structures (that is, the inferior alveolar nerve in case 3). The granulation tissue around the implants was removed using various surgical curettes and the implant surfaces were decontaminated using glycine powder air-polishing (Clinpro Prophy Powder, 3M ESPE). An antibiotic paste (tetracycline) was left on the surface of the implants for 1 minute, then washed with copious saline solution. In case 1, root-end resection for tooth no. 8 was also performed at this stage (Figure 4).

Regeneration procedure

Bony deficiencies were filled using bovine bone material (OsteoBiol, Tecnoss) mixed with autologous bone and finally covered using a collagen membrane (Bio-Gide, Geistlich). Periosteal incision was used to allow a tension-free flap closure, and simple interrupted nonresorbable 5.0 sutures were used to suture the flap in position and to achieve primary closure. Sutures were removed after 14 days. Figures 4 through 6 are intraoperative images of the surgical and regenerative procedures we described.

Histopathologic findings and recall visits

Case 1

The histopathologic report described the lesion as a cyst measuring 7 × 5 millimeters. The patient was followed for 6 years after surgery.

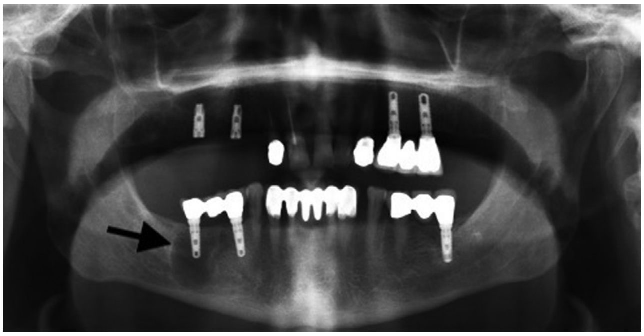

Figure 3. Case 3. Panoramic radiograph showing large poorly defined bone destruction in the mandibular right area (arrow).

Case 2

The histopathologic report described the lesion as a cyst measuring 8 × 5 mm. The patient was followed for 3 years after surgery. No sensory loss occurred.

Case 3

The histopathologic report described the lesion as a cyst measuring 15 × 9 mm. The patient was followed for 10 years after surgery.

During the recall visits, the patients were asymptomatic and clinical examinations revealed no signs of persistent disease. Radiographic examinations at the final recall visits showed complete healing (Figure 7).

DISCUSSION

Our cases were retrograde peri-implantitis associated with residual cysts, which became clinically evident several years after implant placement and its successful long-term management. These cases included immediate (that is, cases 1 and 2) and delayed (that is, case 3) implant placement

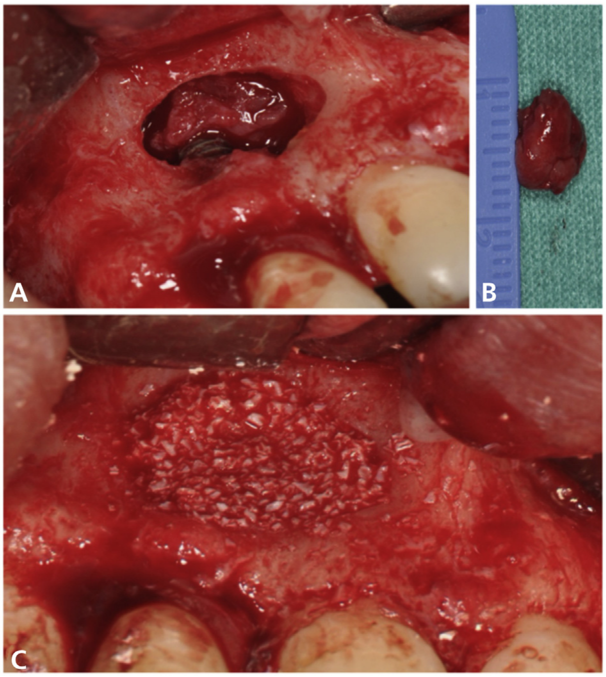

Figure 4. Case 1. Surgical removal of the cyst and bone regeneration procedure. **A.** Preoperative cyst view after buccal bone wall removal, cyst enucleation (**B**), graft condensed in the residual bone defect (**C**).

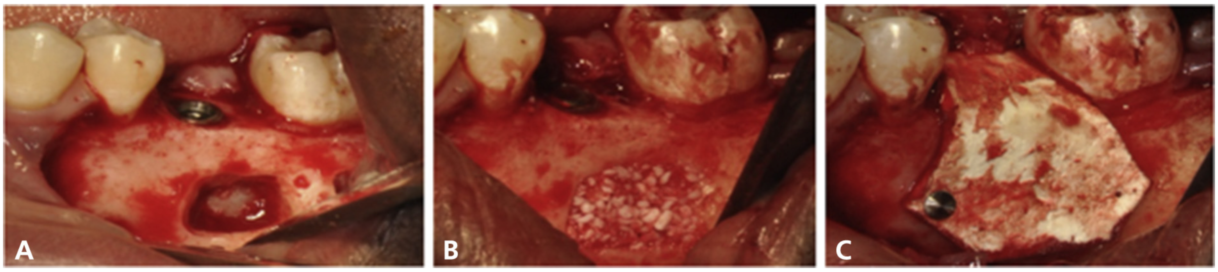

Figure 5. Case 2. Surgical removal of the cyst and bone regeneration procedure. Lesion exposed (**A**), bovine bone material *in situ* (**B**), collagen membrane (**C**).

protocols. Cysts are the most common chronic lesions of the jaws, and their diagnosis is made by means of a combination of radiographic and histopathologic findings,[8] as in our cases. To prevent this subtle complication, the role of local procedures, such as meticulous prophylaxis, socket curettage and debridement, and the use of chlorhexidine, should be reinforced, especially for immediate implant placement to replace teeth associated with periodontal or endodontic infections.[13] The radiographic findings in case 2, after implant placement, suggest inadequate debridement of the periradicular lesion after tooth extraction, as previously extruded root restoration material is evident. Residual cysts are not expected to reoccur after competent enucleation,[8] as in our study, which included long recall periods for all cases.

Bone regenerative procedures were selected in all cases owing to the size and the morphology of the osseous defects around the implants, in particular in case 3. Furthermore, implant surface decontamination was carried out, as the radicular cysts were considered infected owing to the presence of pain and swelling.[8] In the absence of consensus regarding treatment protocols for this infrequent clinical presentation, a published protocol was used to detoxify the exposed implant threads.[14]

The need for long-term recall visits after implant placement, including a regular radiographic evaluation to assess bone loss around the whole fixture, should be reiterated. This would allow for early detection of the different presentations of retrograde peri-implantitis, including cases in which a residual cyst is developing. Furthermore, even if immediate implant placement into extraction sockets with previous pathoses can be successfully obtained in terms of both clinical and esthetic parameters,[15] the possibility of this and other complications should be discussed as part of shared decision making between patients and treatment providers. This is important, considering the expectations of patients regarding shorter treatment periods to fulfill desirable esthetic and functional expectations. The difficulties in the detection and differential diagnosis of intraosseous lesions of the jaws, even when specialized imaging modalities are used, should be restated.[16,17]

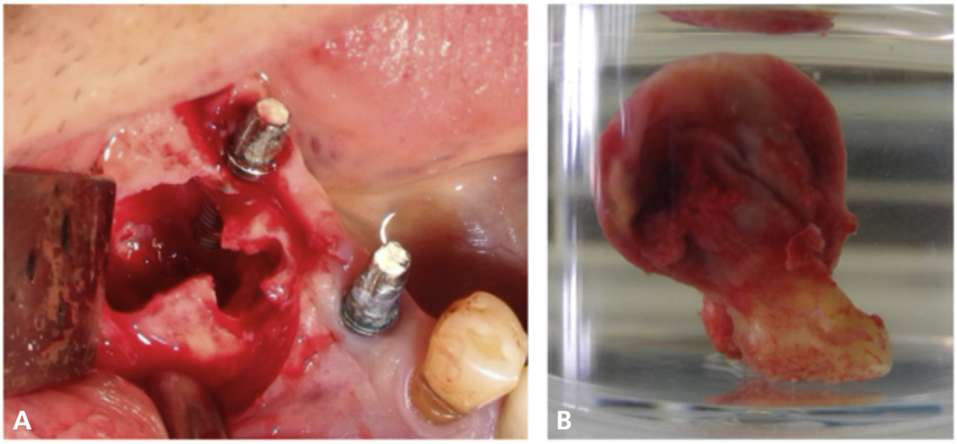

Figure 6. Case 3. Surgical removal of the cyst and bone regeneration procedure. Osseous crypt (**A**) and sample for histopathological assessment (**B**).

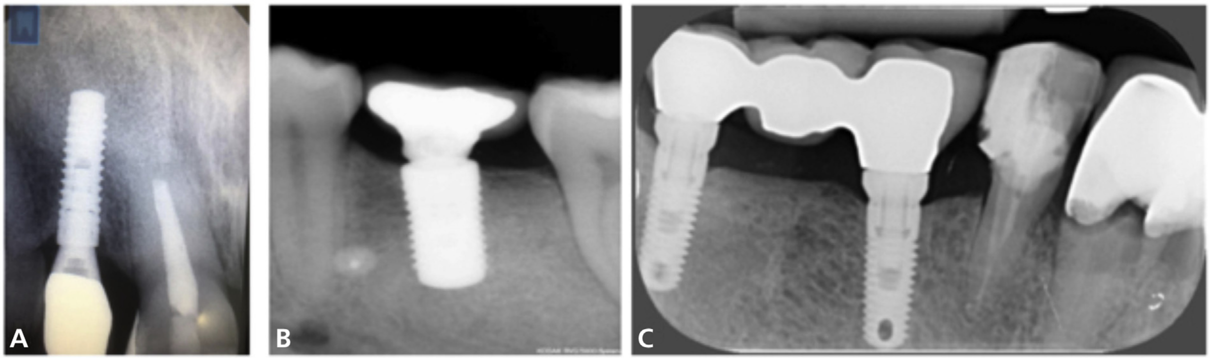

Figure 7. Intraoral radiography images from final recall visits. **A**. Case 1 at 6 years after surgery. **B**. Case 2 at 3 years after surgery. **C**. Case 3 at 10 years after surgery.

CONCLUSIONS

Although the incidence of retrograde peri-implantitis associated with a residual cyst is considered low, this potential complication can occur. Long-term implant retention can be achieved with appropriate surgical and regenerative management. ■

Dr. Roberto Pistilli is a consultant, Azienda Ospedaliera San Camillo Forlanini, Rome, Italy. Address correspondence to Dr. Rossi-Fedele, Adelaide Dental School, University of Adelaide, Level 10, Adelaide Health and Medical Sciences Building, Corner of North Terr and George St, Adelaide, South Australia 5005, Australia, e-mail giampiero.rossi-fedele@adelaide.edu.au.

Dr. Canullo is a practitioner in private practice, Rome, Italy.

Dr. Menini is an assistant professor, Prosthodontic Division, Department of Surgical Sciences, University of Genoa, Genoa, Italy.

Dr. Valeria Pistilli is a practitioner in private practice, Rome, Italy.

Dr. Rossi-Fedele is a professor and head of endodontics, Adelaide Dental School, University of Adelaide, Adelaide, South Australia, Australia.

Dr. Pesce is a research fellow at the Prosthodontic Division, Department of Surgical Sciences, University of Genoa, Genoa, Italy.

Disclosure. None of the authors reported any disclosures.

1. Saito H, Aichelmann-Reidy MB, Oates TW. Advances in implant therapy in North America: improved outcomes and application in the compromised dentition. *Periodontol 2000*. 2020;82(1):225-237.

2. Rossi-Fedele G, Musu D, Cotti E, Doğramacı EJ. Root canal treatment versus single-tooth implant: a systematic review of internet content. *J Endod*. 2016;42(6):846-853.

3. Murray H, Locker D, Kay EJ. Patterns and reasons for tooth extractions in general dental practice in Ontario, Canada. *Community Dent Oral Epidemiol*. 1996;24(3):196-200.

4. Chrysanthakopolous NA. Reasons for extraction of permanent teeth in Greece: a five-year follow-up study. *Int Dent J*. 2011;61(1):19-24.

5. Caldas AF, Marcenes W, Sheiham A. Reasons for tooth extraction in a Brazilian population. *Int Dent J*. 2000;50(5):267-273.

6. Canullo L, Rossi-Fedele G, Camodeca F, Marrucchella G, Doğramacı EJ, Scarano A. Comparative histopathological analysis of granulomatous tissue of endodontic and periodontal origin. *Int J Oral Maxillofac Implant*. 2020;35(3):585-590.

7. Nair PN. On the causes of persistent apical periodontitis: a review. *Int Endod J*. 2006;39(4):249-281.

8. Cawson RA, Odell ED. *Cawson's Essentials of Oral Pathology and Oral Medicine*. 8th ed., St. Louis, MO: Elsevier; 2009:115-119.

9. Lefever D, Van Assche N, Temmerman A, Teughels W, Quirynen M. Aetiology, microbiology and therapy of periapical lesions around oral implants: a retrospective analysis. *J Clin Periodontol*. 2013;40(3):296-302.

10. Marshall G, Canullo L, Logan RM, Rossi-Fedele G. Histopathological and microbiological findings associated with retrograde peri-implantitis of endodontic origin: a systematic and critical review. *Int J Oral Maxillofac Surg*. 2019;48(11):1475-1484.

11. Daubert D, Black RM, Chrepa V, Kotsakis G. Endodontic peri-implant defects: a new disease entity. *J Endod*. 2020;46(3):444-448.

12. Nissen T, Wynn R. The clinical case report: a review of its merits and limitations. *BMC Res Notes*. 2014;7:264.

13. Chrcanovic BR, Martins MD, Wennerberg A. Immediate placement of implants into infected sites: a systematic review. *Clin Implant Dent Relat Res*. 2015;17-(suppl 1):e1-e16.

14. Parma-Benfenati S, Roncati M, Tinti C. Treatment of peri-implantitis: surgical therapeutic approaches based on peri-implantitis defects. *Int J Periodontics Restorative Dent*. 2013;33(5):627-633.

15. Jung RE, Zaugg B, Philipp AO, Truninger TC, Siegenthaler DW, Hammerle CH. A prospective, controlled clinical trial evaluating the clinical radiological and aesthetic outcome after 5 years of immediately placed implants in sockets exhibiting periapical pathology. *Clin Oral Implants Res*. 2013;24(8):839-846.

16. Musu D, Rossi-Fedele G, Campisi G, Cotti E. Ultrasonography in the diagnosis of bone lesions of the jaws: a systematic review. *Oral Surg Oral Med Oral Pathol Oral Radiol*. 2016;122(1):e19-e29.

17. Doğramaci EJ, Rossi-Fedele G, McDonald F. Clinical importance of incidental findings reported on small-volume dental cone beam computed tomography scans focused on impacted maxillary canine teeth. *Oral Surg Oral Med Oral Pathol Oral Radiol*. 2014;118(6):e205-e209.
