# Supplementary material for: A novel histopathological classification of implant periapical lesion: A systematic review and treatment decision tree
Source: PLoS One. 2022 Dec 22;17(12):e0277387. doi: 10.1371/journal.pone.0277387 (PMC9778521; doi:10.1371/journal.pone.0277387)
Supplement: S1 File — (ZIP) [file pone.0277387.s001.zip › support files/Included study/Qu 2013.pdf]

CLINICAL ORAL IMPLANTS RESEARCH

Chunna Qu
Huanxin Meng
Jie Han

Case Report

Implant periapical lesion – a review and a case report with histological evaluation

Authors' affiliations:
Chunna Qu, Huanxin Meng, Jie Han, Department of Periodontology, Peking University School and Hospital of Stomatology, Beijing, China

Corresponding author:
Jie Han
Jie zhongguancun nandajie No.22 Beijing 100081, China
Tel.: 86 10 82195372
Fax: 86 10 62173402
e-mail: han_jie17@sina.com

Key words: complications, dental implant, etiology, failures, implants periapical lesion, treatment

Abstract

Objective: Information of implant periapical lesion(IPL) is insufficient by now. The aim of this review is to analyze the possible causes of IPL and to find out the proper treatment protocol.
Material and methods: A systematic literature search was carried out (up to June 2012) using relevant search terms in PUBMED. And a case of IPL with histological evaluation is reported.
Results: The IPL is accompanied by the radiolucency around the apex of the implant and usually presents symptoms of red, pain, tenderness, swelling, or fistulous tract. It may occur due to infection, overheating, poor bone quality of the implant site, etc. Debridement is considered as the crucial procedure of the treatment, and the prognosis is normally good when assessed by clinical parameters and radiograph.
Conclusion: The causes of IPL seem to be multifactorial. Most of the cases are connected with preexisting infection. Elimination of the infection is the only consensus of the treatment protocols.

Reiser & Nevins (1995) first defined the bone loss limited to the apical segment as 'implant periapical lesion' (IPL), which is also known as 'apical peri-implantitis' or 'retrograde peri-implantitis' (Flanagan 2002) in the literature. A systematic literature search was carried out (up to June 2012) using relevant search terms in PUBMED. Information of IPL is infrequent and can only be collected from some case reports and a few reviews, and about 126 cases of IPLs are reported in the literature. Implant periapical lesion starts at the implant apex, but it exhibits the capacity of spreading coronally, proximally, and facially. (Reiser & Nevins 1995) Although the lesion is rare, it could lead to implant failure. (Quirynen et al. 2005).

The first aim of the review is to summarize the likely etiology of the implant periapical lesion; the second is to find out the proper treatment protocol.

Prevalence

The prevalence of IPLs differs in the literature. Reiser & Nevins (1995) reported an incidence of 0.26% (10/3800). In a retrospective study of 539 implants, Quirynen et al. (2005) reported an incidence of 1.6% in the upper jaw and 2.7% in the lower jaw. Zhou et al. (2012) reported five cases of 2987 implants, indicating a prevalence of 0.17%. Penarrocha-Diago et al. (2009) reported seven cases of 2500 implants, indicating a prevalence of 0.28%.

Classification

The implant periapical lesions can be classified into active and inactive lesions according to the activity of the infection. (Reiser & Nevins 1995) Penarrocha Diago et al. (2006)distinguished the implant periapical lesion into acute and chronic phase.

Etiology

The exact mechanism of bone loss around the implant apex is still not well understood.

The inactive lesion may be caused by periapical scar, which probably comes from the pre-existing scar or the residual bone cavity when the implant is shorter than the implant cavity, and then it is repaired with dense connective tissue instead of bone. (Reiser & Nevins 1995; Waasdrop & Reynolds 2010).

Date:
Accepted 8 June 2013

To cite this article:
Qu C, Meng H, Han J. Implant periapical lesion – a review and a case report with histological evaluation.
Clin. Oral Impl. Res. **25**, 2014, 1099–1104
doi: 10.1111/clr.12232

© 2013 John Wiley & Sons A/S. Published by John Wiley & Sons Ltd

As for the active lesion, many predisposing conditions were discussed in the literature.

Infection

The infection could come from the pre-existing infection of the implant site (Ayangco & Sheridan 2001; Brisman et al. 2001; Flanagan 2002; Park et al. 2004; Quirynen et al. 2005; Ataullah et al. 2006), adjacent teeth(Sussman 1998; Scarano et al. 2000; Chaffee et al. 2001; Oh et al. 2003; Quirynen et al. 2005; Tozum et al. 2006), infected maxillary sinus(Reiser & Nevins 1995), or contamination during the surgery(Rokadiya & Malden 2008; Penarrocha-Diago et al. 2009).

According to these case reports, about half of the lesions were linked with a history of obvious endodontic pathologies, either the implant site or the adjacent teeth. The pathologies became quiescent due to removal of infection source and host response, but implant surgery at this site reactivated the infection and its development was exacerbated. (Sun et al. 2012).

Microorganisms found in IPL resemble the composition of endodontic pathogens. (Park et al. 2004) It was suggested that microorganisms may persist even though the endodontic therapy was considered successful radiographically and clinically. (Brisman et al. 2001) Histological evaluations of periapical tissue after endodontic therapy in cadavers, animals, or humans confirmed that with an radiographically optimal healing, 26–50% of teeth exhibited histological signs of inflammation or persisting microorganisms. (Green et al. 1997; Seltzer 1999) It was also indicated that even after thorough debridement and irrigation of the extraction sockets, infection remained in the bone and led to the initiation of IPLs. (Ayangco & Sheridan 2001; Quirynen et al. 2005) Silva et al. (2010) reported a case of immediate implant insertion after the extraction of infected periodontally compromised teeth, and presumed, most likely, IPL resulted from the deficiency of effective alveolar debridement and decontamination before the implant placement. Contamination during the surgery is also considered as one of the likely etiology of IPLs.(Rokadiya & Malden 2008; Penarrocha-Diago et al. 2009).

Overheating

Insufficient cooling of drill, as well as the use of excessive drilling speed and exertion of excessive force for preparing dense impact bone could lead to the overheating of the bottom of the cavity, which might result in aseptic necrosis of the bone, affect the osseointegration process at the apical area, and sensitize this area to the bacteria. (Reiser & Nevins 1995; Bousdras et al. 2006; Chang et al. 2011; Zhou et al. 2012) The longer the implant was, the higher the risk of bone overheating, especially when the implant length exceeded 12 mm. (Bousdras et al. 2006) In a retrospective study, Balshi et al. (2007) reported 35 patients with 39 IPLs. Totally, 395 implants were inserted in these patients. The mean length of these 39 IPLs was 15.5 mm, so we speculate that these 39 IPLs might be due to insufficient cooling in the deeper osteotomy.

The characteristics of the implant surface

Quirynen et al. (2005) observed that implants with enhanced/modified surface characteristics demonstrated a higher incidence of IPLs compared with the machined titanium surface (8/80 vs. 2/459). The hypothesis is that when coming in contact with a granuloma or endodontic pathology, the machined implants will soon be completely surrounded by granulation tissue before any osteointegration can form, resulting in the loss of the implant, while the modified implants can still integrate at the coronal part before fibrous encapsulation reaches this area because of the accelerated osseointegration process, which representing IPL.(Quirynen et al. 2005).

Poor bone quality of the implant site

The scarcity of osteoprogenitor cells at the surgical site could impede the osseointegration of the implant, and the histochemical analyses show a complete absence of actively secreting osteoblasts around the IPLs. (Piattelli et al. 1998a,b).

Microfracture

Microfracture could result in ischemia, aseptic necrosis, and formation of a bone sequestrum. Overloading or premature loading, excessive tightening caused by self-tapping implant or oversize implant were considered as the reasons of microfracture.(Reiser & Nevins 1995; Piattelli et al. 1998a,b; Quirynen et al. 2005; Bousdras et al. 2006; Chang et al. 2011).

Foreign bodies

Starch particles from rubber gloves were found in a specimen of the IPL, suggesting that the IPL may be provoked by the foreign-body reaction. (Nedir et al. 2007).

Epithelial rests of Malassez

Reiser & Nevins (1995) speculated that the IPL may come from the epithelial rests of Malassez. The maxilla contains far more epithelial; therefore, it is conceivable that dental granuloma has a greater potential of becoming a radicular cyst in the maxilla than it has in mandible.

Penetration of the bone

Silva et al. (2010) reported a case of IPL caused by the penetration of the two-thirds of the implant into the nasal cavity, after 1.5 years of insertion, a large cyst involving the apex, and middle portion of the implant was revealed.

The condition of the patient

Like the periapical lesion, the IPL could be caused by anachoresis, especially when the patient's immune response is impaired. (Rosendahl et al. 2009) Two cases were reported in the medically compromised patients, one was associated with actinomycosis (Sun et al. 2012) and the other was previously diagnosed with human immunodeficiency virus infection (Chan et al. 2011).

Pathology

Histological examination was reported in fifty-one cases of IPLs. Histopathology diagnoses included periapical inflammatory cyst (Silva et al. 2010), aseptic bone necrosis (Piattelli et al. 1998a,b; Scarano et al. 2000), granulation tissue with acute inflammatory infiltrated cells (Piattelli et al. 1998a,b; Balshi et al. 2007; Dahlin et al. 2009; Penarrocha-Diago et al. 2009; Rosendahl et al. 2009; Chan et al. 2011; Sun et al. 2012), fibrous connective tissue containing a dense chronic inflammatory infiltration (Nedir et al. 2007; Chan et al. 2011), or the infiltration with acute and chronic inflammatory cells (Chaffee et al. 2001). Some pathogens were detected by microbial testing; pathogenic bacteria such as Staphylococcus aureus (Rokadiya & Malden 2008), Eikenella corrodens (Chan et al. 2011), and Actinomycetes (Sun et al. 2012) were detected.

Clinical manifestation

According to the case reports, the IPL could occur at different time, varying from 1 week to 11 years after implant placement. All the involved implants were stable, except one (Oh et al. 2003), even though they remained in contact with the bone only with the most coronal four to five threads.(Quirynen et al. 2005).

Red, pain, tenderness, swelling, and fistula tract in the local mucosa of the implant periapical area are the most reported clinical manifestations in the literature. The pain is

© 2013 John Wiley & Sons A/S. Published by John Wiley & Sons Ltd

intense and constant. The acute implant periapical lesion manifests as spontaneous pain (Flanagan 2002; Rokadiya & Malden 2008; Dahlin et al. 2009; Penarrocha-Diago et al. 2009; Rosendahl et al. 2009; Zhou et al. 2012) and dull to percussion (Penarrocha-Diago et al. 2009; Zhou et al. 2012). The gingival inflammation and the increased probing depth are reported only in one case (Sun et al. 2012).

When a full-thickness flap is elevated, a large perforation of the buccal or lingual bone plate could be observed in some cases (Quirynen et al. 2005; Tozum et al. 2006; Quaranta et al. 2012), and the implant is always surrounded by granulation tissue. Some cases (Rokadiya & Malden 2008; Penarrocha-Diago et al. 2009; Zhou et al. 2012) demonstrated suppurative lesions in the apical area without fistula during the surgery, that would probably be due to the interpretation at an early stage, within 1–5 weeks after the implant insertion.

All the cases show the radiolucency around the apex of the implant, while some of the cases are detected radiographically only without the clinical manifestations. (Quirynen et al. 2005; Balshi et al. 2007; Waasdrop & Reynolds 2010).

Also, the IPL could potentially cause devitalization of adjacent teeth (Sussman 1998), maxillary sinus reaction, or acute osteomyelitis (Rokadiya & Malden 2008; Penarrocha-Diago et al. 2009).

Treatment

The inactive lesion presents the radiolucency around the apex of the implant without clinical symptoms. Waasdrop & Reynolds (2010)reported an inactive lesion healed gradually after 10-day course of amoxicillin (500 mg three times daily) with 1-year periodic evaluation. But, most other researchers believe that the inactive lesion is not a true lesion and treatment is unnecessary, unless its diameter increases. Observing and monitoring periodically is enough, especially radiographic assessment. (Reiser & Nevins 1995).

As for the active lesion, elimination of the infection is the only consensus of the treatment protocols. If there is a connection between the lesion and the adjacent tooth which has a recurrent or latent endodontic lesion, sufficient and immediate debridement of the adjacent teeth should be carried out to eliminate the potential infection. (Oh et al. 2003; Park et al. 2004; Tozum et al. 2006) Factors including the mobility of the implant, the size of the implant periapical lesion, the time of the lesion appeared, the implant position, the type of the implant, and the type and quality of the prosthetic rehabilitation should be considered when making treatment plan. If the implant is mobile, or the infection could not be eliminated, the implant must be removed immediately to prevent osteomyelitis (Sussman 1998). Then, a wider-diameter (Park et al. 2004) or same diameter (Oh et al. 2003) implant could be replaced.

The treatment protocols can be classified into two stages: nonsurgical treatment and surgery.

Nonsurgical treatment

Systematic antibiotics are the mostly used nonsurgical treatment. Amoxicillin 500 mg or clindamycin 300 mg, three times per day, for a week is mostly used, with or without metronidazole or local treatment. However, most of the nonsurgical treatment fails to prevent the progression of IPLs. (Bousdras et al. 2006; Nedir et al. 2007; Rokadiya & Malden 2008; Dahlin et al. 2009; Penarrocha-Diago et al. 2009) Therefore,systematic antibiotics are recommended to be prescribed to slow down disease progression until definitive surgical intervention. (Park et al. 2004).

Chang et al. (2011) reported an active lesion, which healing after the medical treatment (prednisolone 5 mg three times daily for 3 days plus augmentin 375 mg and mefenamic acid 250 mg three times daily for 7 days).

Surgery

To eliminate the infection sufficiently, surgery is recommended. Debridement, complete removal of all the granulation tissue and curettage of the bony cavity walls are considered as the crucial procedures of the surgery, while resection of the implant apex and GTR is also carried out in some cases.

The necessity of apex resection was discussed in the literature. The residual biofilm on the enhanced surface of the implant apex (Reiser & Nevins 1995; Dahlin et al. 2009) and the perforated hollow design which was difficult to access (Nedir et al. 2007) were considered as the reasons of the resection. However, in another report (Quirynen et al. 2005), curettage without the resection succeeded in preventing further progression of IPLs with the perforated hollow design implants in the upper jaw.

As for the GBR, about half of the cases were treated by bone grafting and/or absorbable membranes after curettage to prevent fibrous connective tissue invasion into the defect. If the defect size is small (<5 mm), absorbable membrane or bone graft alone is enough; otherwise, absorbable membrane and bone graft are both needed. (Park et al. 2004; Chan et al. 2011) However, Flanagan (2002) and Ayangco & Sheridan (2001) believed that there was no need for grafting or using a membrane.

Irrigation is necessary to remove the additional soft tissue or implant debris. Chlorhexidine and/or saline are mostly used. (Ataullah et al. 2006; Balshi et al. 2007; Zhou et al. 2012) Moreover, after the irrigation, Ataullah et al. (2006) suggested a dabbing with saline-soaked gauze for approximately 5 min, Ayangco & Sheridan (2001) applied tetracycline paste to the zone for 1 min to ensure local disinfection. Flanagan (2002) suggested a paste of calcium hydroxide in water, which has been shown to be a better inhibitor of activity of bacterial species commonly involved in endodontic infection than chlorhexidine.

After the surgery, systemic antibiotic and chlorhexidine gargle are usually prescribed.

Prognosis

Most of the IPLs are successfully treated when assessed by the parameters such as no clinical symptomas, implant stability, and radiological healing, with the follow-up period varying from a minimum of 2 months (Quirynen et al. 2005) to a maximum of 15 years (Balshi et al. 2007).

Radiographically, the bone remodeling process was still ongoing in the area of the apex resection of a 56-year-old patient after 2 years of the surgery. (Bousdras et al. 2006) But, does the radiological healing of the apical lesion mean reosseointegration? There seems to be no definite answers. Jalbout & Tarnow (2001) described a type of healing composed of fibrous soft tissue band, and they suggested that reossointegration may not have been achieved because sufficiently eliminating bacterial endotoxins from the implant surface was difficult. Without reossointegration, the residual implant length would offer less apical bone anchorage for the implant, possibly resulting in less optimal support to the dental prosthesis. Therefore, it is likely that the initial treatment plan and/or superstructure design would need to be modified according to biomechanical principles. (Bousdras et al. 2006).

Prevention

Assessment and treatment planning are necessary; patients' compliance should also be taken into account.

© 2013 John Wiley & Sons A/S. Published by John Wiley & Sons Ltd

As the high incidence of IPL at sites with a history of a periapical granuloma, a more detailed analysis of the radiograph before implant insertion should be part of implant treatment planning routinely, although remaining pathologies are often not detectable on the radiographs.(Park et al. 2004; Quirynen et al. 2005) Quirynen et al. (2005) suggested that the healing time between tooth extraction and implant insertion should be sufficient to prevent the activation of residual bacteria. However, Bell et al. (2011) thought that the placement of implants in sockets affected by chronic periapical pathology could be considered as a safe and viable treatment option, while there was a risk of implant failure when placing implants adjacent to teeth with periapical radiolucencies.

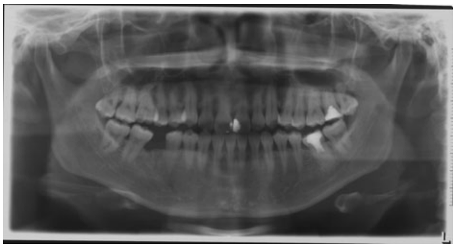

Fig. 1. Orthopantomography (OPG) showed normal bone healing 4 months after extraction of 46.

When implants are placed adjacent to natural teeth with a history of pulpal or periapical disease, a vitality test or quality assessment of their endodontic therapy should be part of implant treatment planning routinely. (Park et al. 2004; Quirynen et al. 2005) Sufficient and immediate debridement of the adjacent teeth should be considered prior to implant placement. (Park et al. 2004) The incidence of retrograde peri-implantitis may be reduced by increasing the distance between the implant and adjacent tooth, and/or the duration from endodontically treated adjacent tooth to implant placement. (Zhou et al. 2009).

When implant inserted immediately after the extraction of infected periodontally compromised teeth, adequate debridement of sockets is recommended. (Silva et al. 2010) During the surgery, more attention should be paid to the aseptic and the minimally traumatic surgical technique, keeping the implant away of saliva, teeth, oral tissues, or the surgeon's gloves and so on. The variations in bone density, sufficient cooling of the drill, and clearing up the bone chips before insertion of implant must be taken care of. (Bousdras et al. 2006).

Conclusion

The implant periapical lesion is confirmed according to the radiolucency around the implant apex. The etiology of IPL seems to be multifactorial or with an unknown origin. Most of the cases are connected with infection. A rapid and exact diagnosis is very important. Once diagnosis of the IPL is confirmed, intervention should be performed as early as possible to limit the extent of disease progression. The criteria for choosing a proper treatment protocol for IPL are still indeterminate. Under sufficient debridement, the surgery should be minimal invasive according to the progression and the size of the lesion. Factors influencing the prognosis of IPLs cannot be determined unless longer follow-up and more cases are reported.

A Case report

A 28-year-old female asked for implant restoration of tooth 46. The patient lost tooth 46 in January 2010 due to caries and residual roots. Normal bone healing was observed 4 months after extraction (Fig. 1). Then, a Straumann dental implant (SLA SP 4.1*10 RN) was placed into the extracted area (Fig. 2). The bone at the implant site was quite hard (Fig. 3), and bone quality class II was recorded. The adjacent teeth were healthy. The healing process was uneventfully. There were no symptoms of pain or discomfort. Clinical examination reported no swelling, no increased probing depth, and no sign of gingival inflammation. No fistulas were observed. The implant was stable with the ISQ = 71/80.

However, an implant periapical lesion was detected via the radiograph taken after abutment connection, 5 months after the implant insertion. A large periapical radiolucency (10 mm*10 mm) was noted on the X-ray,

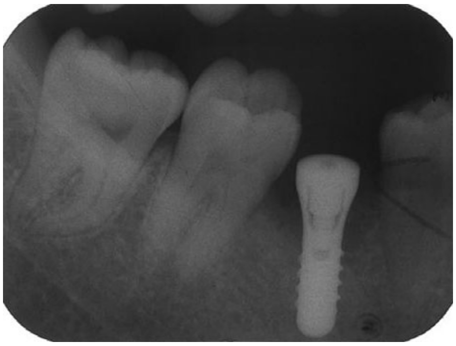

Fig. 2. Periapical radiograph taken on the day of Straumann dental implant (SLA SP 4.1*10 RN) was placed into the extracted area.

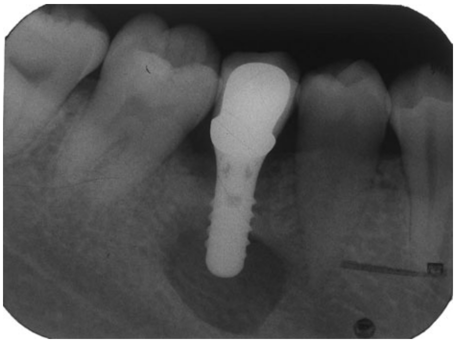

Fig. 4. A large periapical radiolucency (10 mm*10 mm) was noted on the periapical radiograph 5 months after insertion.

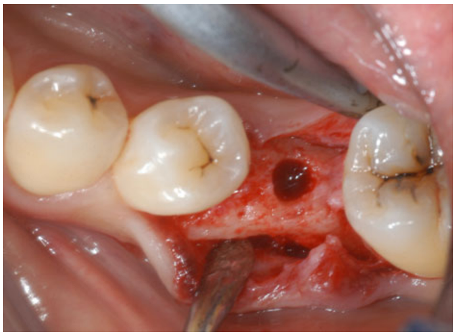

Fig. 3. Photograph taken during the implant insertion surgery.

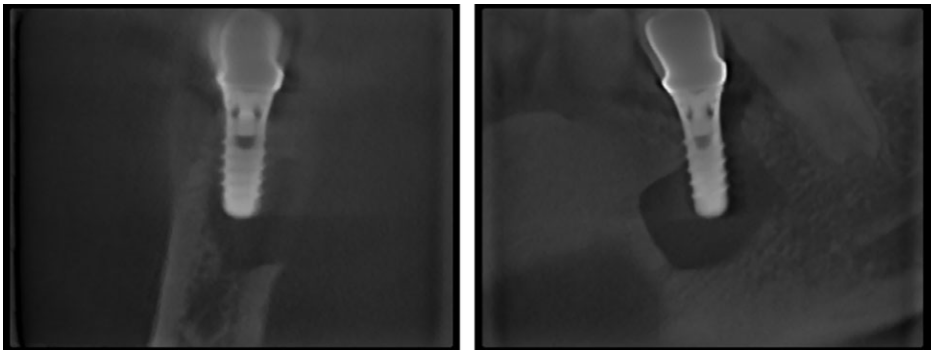

Fig. 5. Cone beam CT (CBCT) clarified a large bony defect showing that apical half of the lingual cortical bone was discontinuous.

© 2013 John Wiley & Sons A/S. Published by John Wiley & Sons Ltd

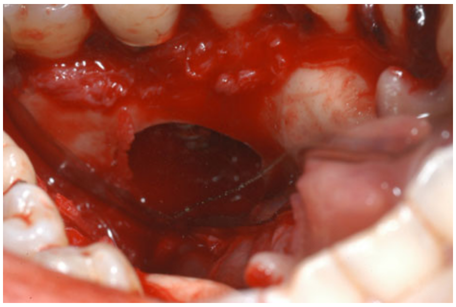

Fig. 6. After removal of the cyst and curettage of the bone cavity, a large cavity (about 15 mm*10 mm*10 mm) was observed.

which was close to the apex of tooth 45 (Fig. 4). Cone beam CT (CBCT) clarified a large bony defect, showing that apical half of the lingual cortical bone was discontinuous (Fig. 5), and the distal part of the apex of tooth 45 was at the border of the radiolucency, while the implant was still stable. A soft cystic mass was found at the apical area of the lingual mucosa when palpation. Light yellow translucent cystic fluid was found by puncture. Neutrophils and a few epithelial cell masses were observed in the smear.

Considering the large extension of bone destruction, even asymptomatic, surgery was indicated. The procedure was carried out under local anesthesia. A lingual flap was elevated to expose the bone of the apical area. Fenestration was found, and a light blue cystic lesion was exposed, whose capsule was integrated. After removal of the cyst and curettage of the bone cavity carefully, a large cavity (about 15 mm*10 mm *10 mm) was observed (Fig. 6), and the distal part of the apex of tooth 45 has already been involved in the lesion. Irrigated the lesion with sufficient sterile saline, then a Bio-Gide membrane was used to cover the lesion without bone substitute grafting and the flap was tightly re-sutured. The systematic antibiotics (amoxicillin and metronidazole) were prescribed for 8 days after the surgery.

The histological examination of the biopsy sample showed inflammatory cyst wall-like lesion, with the infiltration of macrophage and lymphocytes, but the epithelial lining was undetected (Fig. 7).

Six days later, the patient complained the spontaneous pain of the mandible right posterior area, and the tooth 45 was diagnosed as acute pulpitis. After the root canal treatment of tooth 45, the pain disappeared. One month checkup demonstrated no pain or discomfort for the patient. No signs of infection were noted. At 14 months follow-up, the patient remained asymptomatic. CBCT showed increased radiographic bone density, the huge radiolucency around the apical region of the implant disappeared, and lingual bone repaired (Fig. 8). At 22 months follow-up, radiographs revealed complete bone fill and continuous stable bone levels around the previously affected implants (Fig. 9). The implant was stable with no further symptoms.

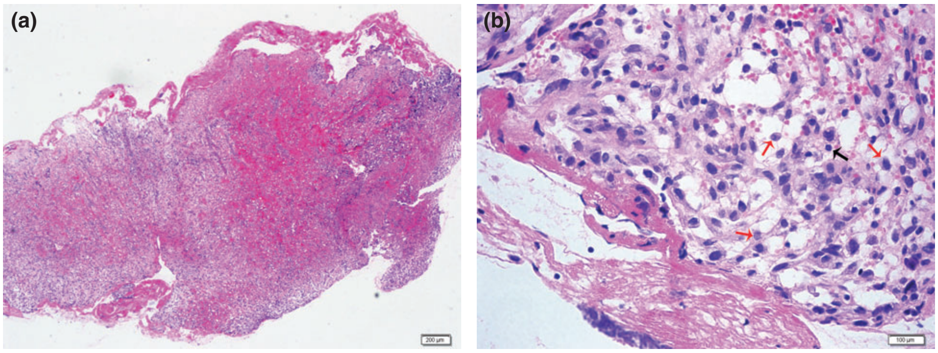

Fig. 7. (a) The histological examination showed inflammatory cystic-like tissue, but the epithelial lining was undetected. (b) Large numbers of macrophage cells (red arrow) and fewer numbers of lymphocytes (black arrow) are involved.

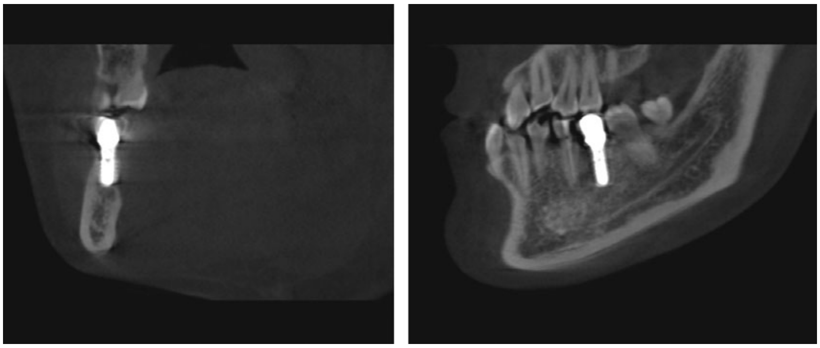

Fig. 8. At 14 months follow-up, CBCT showed increased radiographic bone density, the huge radiolucency lesion around the apical region of the implant disappeared and lingual bone repaired.

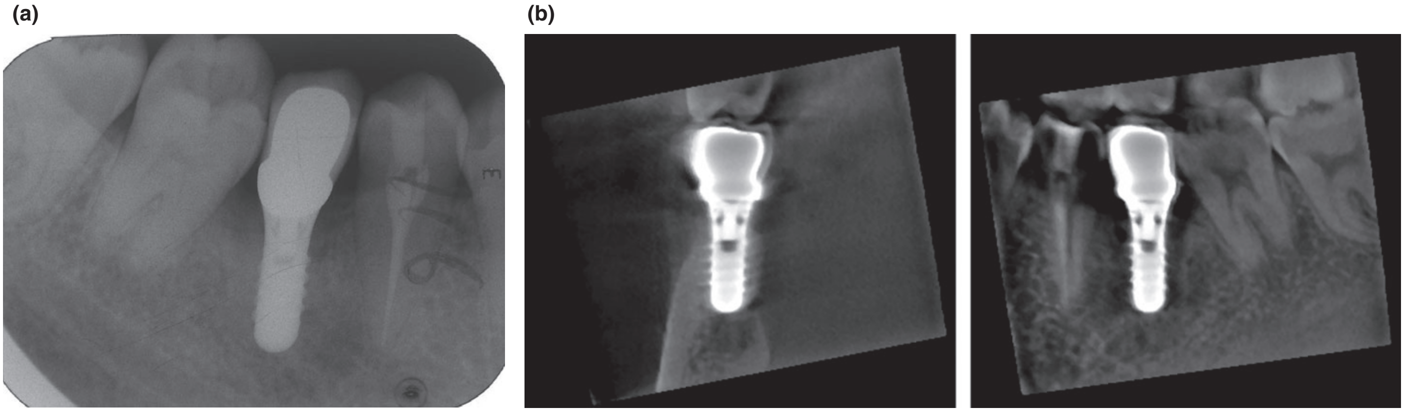

Fig. 9. (a) At 22 months follow-up, radiographs revealed complete bone fill and continuous stable bone levels around the implant. (b) At 22 months follow-up, radiographs revealed complete bone fill and continuous stable bone levels around the implant.

© 2013 John Wiley & Sons A/S. Published by John Wiley & Sons Ltd

Reference

Ataullah, K., Chee, L.F., Peng, L.L. & Lung, H.H. (2006) Management of retrograde peri-implantitis: a clinical case report. *The Journal of Oral Implantology* **32**: 308–312.

Ayangco, L. & Sheridan, P.J. (2001) Development and treatment of retrograde peri-implantitis involving a site with a history of failed endodontic and apicoectomy procedures: a series of reports. *The International Journal of Oral & Maxillofacial Implants* **16**: 412–417.

Balshi, S.F., Wolfinger, G.J. & Balshi, T.J. (2007) A retrospective evaluation of a treatment protocol for dental implant periapical lesions: long-term results of 39 implant apicoectomies. *The International Journal of Oral & Maxillofacial Implants* **22**: 267–272.

Bell, C.L., Diehl, D., Bell, B.M. & Bell, R.E. (2011) The immediate placement of dental implants into extraction sites with periapical lesions: a retrospective chart review. *Journal of Oral and Maxillofacial surgery* **69**: 1623–1627.

Bousdras, V., Aghabeigi, B., Hopper, C. & Sindet-Pedersen, S. (2006) Management of apical bone loss around a mandibular implant: a case report. *The International Journal of Oral & Maxillofacial Implants* **21**: 439–444.

Brisman, D.L., Brisman, A.S. & Moses, M.S. (2001) Implant failures associated with asymptomatic endodontically treated teeth. *Journal of the American Dental Association* **132**: 191–195.

Chaffee, N.R., Lowden, K., Tiffee, J.C. & Cooper, L.F. (2001) Periapical abscess formation and resolution adjacent to dental implants: a clinical report. *The Journal of Prosthetic Dentistry* **85**: 109–112.

Chan, H.L., Wang, H.L., Bashutski, J.D., Edwards, P.C., Fu, J.H. & Oh, T.J. (2011) Retrograde peri-implantitis: a case report introducing an approach to its management. *Journal of Periodontology* **82**: 1080–1088.

Chang, L.C., Hsu, C.S. & Lee, Y.L. (2011) Successful medical treatment of an implant periapical lesion: a case report. *Chang Gung Medical Journal* **34**: 109–114.

Dahlin, C., Nikfarid, H., Alsen, B. & Kashani, H. (2009) Apical peri-implantitis: possible predisposing factors, case reports, and surgical treatment suggestions. *Clinical Implant Dentistry and Related Research* **11**: 222–227.

Flanagan, D. (2002) Apical (retrograde) peri-implantitis: a case report of an active lesion. *Journal of Oral Implantology* **28**: 92–96.

Green, T.L., Walton, R.E., Taylor, J.K. & Merrell, P. (1997) Radiographic and histologic periapical findings of root canal treated teeth in cadaver. *Oral Surgery, Oral Medicine, Oral Pathology, Oral Radiology, and Endodontics* **83**: 707–711.

Jalbout, Z.N. & Tarnow, D.P. (2001) The implant periapical lesion: four case reports and review of the literature. *Practical Procedures & Aesthetic Dentistry* **13**: 107–112. quiz 114.

Nedir, R., Bischof, M., Pujol, O., Houriet, R., Samson, J. & Lombardi, T. (2007) Starch-induced implant periapical lesion: a case report. *The International Journal of Oral & Maxillofacial Implants* **22**: 1001–1006.

Oh, T.-J., Yoon, J. & Wang, H.-L. (2003) Management of the implant periapical lesion: a case report. *Implant Dentistry* **12**: 41–46.

Park, S.H., Sorensen, W.P. & Wang, H.L. (2004) Management and prevention of retrograde peri-implant infection from retained root tips: two case reports. *The International Journal of Periodontics & Restorative Dentistry* **24**: 422–433.

Penarrocha Diago, M., Boronat Lopez, A. & Lamas Pelayo, J. (2006) Update in dental implant periapical surgery. *Medicina oral, patología oral y cirugía bucal* **11**: E429–E432.

Penarrocha-Diago, M., Boronat-Lopez, A. & Garcia-Mira, B. (2009) Inflammatory implant periapical lesion: etiology, diagnosis, and treatment–presentation of 7 cases. *Journal of Oral and Maxillofacial Surgery* **67**: 168–173.

Piattelli, A., Scarano, A., Balleri, P. & Favero, G.A. (1998a) Clinical and histologic evaluation of an active 'implant periapical lesion': a case report. *The International Journal of Oral & Maxillofacial Implants* **13**: 713–716.

Piattelli, A., Scarano, A., Piattelli, M. & Podda, G. (1998b) Implant periapical lesions: clinical, histologic, and histochemical aspects. A case report. *The International Journal of Periodontics & Restorative Dentistry* **18**: 181–187.

Quaranta, A., Andreana, S., Pompa, G. & Procaccini, M. (2012) Active implant peri-apical lesion: a case report treated via guided bone regeneration with a 5-year clinical and radiographic follow-up. *The Journal of Oral Implantology* [Epub ahead of print].

Quirynen, M., Vogels, R., Alsaadi, G., Naert, I., Jacobs, R. & van Steenberghe, D. (2005) Predisposing conditions for retrograde peri-implantitis, and treatment suggestions. *Clinical Oral Implants Research* **16**: 599–608.

Reiser, G.M. & Nevins, M. (1995) The implant periapical lesion: etiology, prevention, and treatment. *Compendium of Continuing Education in Dentistry* **16**: 768. 770, 772 passim.

Rokadiya, S. & Malden, N.J. (2008) An implant periapical lesion leading to acute osteomyelitis with isolation of staphylococcus aureus. *British Dental Journal* **205**: 489–491.

Rosendahl, K., Dahlberg, G., Kisch, J. & Nilner, K. (2009) Implant periapical lesion. A case series report. *Swedish Dental Journal* **33**: 49–58.

Scarano, A., Di Domizio, P., Petrone, G., Iezzi, G. & Piattelli, A. (2000) Implant periapical lesion: a clinical and histologic case report. *The Journal of Oral Implantology* **26**: 109–113.

Seltzer, S. (1999) Long-term radiographic and histological observations of endodontically treated teeth. *Journal of Endodontics* **25**: 818–822.

Silva, G.C., Oliveira, D.R., Vieira, T.C., Magalhaes, C.S. & Moreira, A.N. (2010) Unusual presentation of active implant periapical lesions: a report of two cases. *Journal of Oral Science* **52**: 491–494.

Sun, C.X., Henkin, J.M., Ririe, C. & Javadi, E. (2012) Implant failure associated with actinomycosis in a medically compromised patient. *The Journal of Oral Implantology* **39**: 206–209.

Sussman, H.I. (1998) Periapical implant pathology. *The Journal of Oral Implantology* **24**: 133–138.

Tozum, T.F., Sencimen, M., Ortakoglu, K., Ozdemir, A., Aydin, O.C. & Keles, M. (2006) Diagnosis and treatment of a large periapical implant lesion associated with adjacent natural tooth: a case report. *Oral Surgery, Oral Medicine, Oral Pathology, Oral Radiology, and Endodontics* **101**: e132–e138.

Waasdrop, J. & Reynolds, M. (2010) Nonsurgical treatment of retrograde peri-implantitis: a case report. *The International Journal of Oral & Maxillofacial Implants* **25**: 831–833.

Zhou, Y., Cheng, Z., Wu, M., Hong, Z. & Gu, Z. (2012) Trepanation and curettage treatment for acute implant periapical lesions. *International Journal of Oral and Maxillofacial Surgery* **41**: 171–175.

Zhou, W., Han, C., Li, D., Li, Y., Song, Y. & Zhao, Y. (2009) Endodontic treatment of teeth induces retrograde peri-implantitis. *Clinical Oral Implants Research* **20**: 1326–1332.

 © 2013 John Wiley & Sons A/S. Published by John Wiley & Sons Ltd
