# Supplementary material for: A novel histopathological classification of implant periapical lesion: A systematic review and treatment decision tree
Source: PLoS One. 2022 Dec 22;17(12):e0277387. doi: 10.1371/journal.pone.0277387 (PMC9778521; doi:10.1371/journal.pone.0277387)
Supplement: S1 File — (ZIP) [file pone.0277387.s001.zip › support files/Included study/Scarano 2000.pdf]

◄ R
film

Implant Periapical Lesion: A Clinical and Histologic Case Report

Antonio Scarano, DDS
Pietro Di Domizio, MD, DDS
Giovanna Petrone, DDS
Giovanna Iezzi, DDS
Adriano Piattelli, MD, DDS

KEY WORDS

Bone necrosis
Implant failure
Titanium implant

A new pathologic entity called implant periapical lesion has been recently described. This lesion could be produced by contamination of the implant surface, overheating of bone, overloading of the implant, presence of a pre-existing bone pathology, presence of residual root fragments and foreign bodies in bone, implant placement in an infected maxillary sinus, implant placement in a poor bone quality site, or lack of biocompatibility. A 49-year-old female patient underwent the placement of a screw-shaped titanium dental implant in the premolar region of the right mandible. Six months after implant insertion, the patient presented with a persistent pain resistant to analgesics. No fistula was present at a clinical intraoral examination. A periapical x-ray showed the presence of a radiolucency at the apical portion of the implant; this image was confirmed by a CT Scan. The implant was removed. After implant removal, the pain disappeared completely. The specimen was processed to obtain thin ground sections. The histologic examination showed the presence of necrotic bone in the external and apical portion of the antirotational hole of the implant. The etiology of the implant failure in this instance could be related, probably, to an implant contamination of the apical portion of the implant.

Antonio Scarano, DDS, *is a Research Fellow, Dental School, University of Chieti, Via F. Sciucchi 63, 66100 Chieti, Italy.*

Pietro Di Domizio, MD, DDS *is in private practice in Pescara, Italy.*

Giovanna Petrone, DDS, *and* ***Giovanna Iezzi, DDS,*** *are Research Fellows, Dental School, University of Chieti, Chieti, Italy.*

Adriano Piattelli, MD, DDS, *is Professor of Oral Medicine and Pathology, Dental School, Chieti, Italy, and Honorary Senior Lecturer, Eastman Dental Institute for Oral Health Care Sciences, London, UK.*

Introduction

Dental implant failures can be divided into biological, mechanical, iatrogenic, and functional.[1–4] Biological failure can be defined as the inadequacy of the host tissue to establish or maintain osseointegration.[5] Biological failures may be divided into early or primary (before bridge insertion) and late or secondary (after prosthetic rehabilitation).[1–5]

We have little information on the etiopathogenesis of early failures, and they should be considered as the result of a lack of osteogenic response due to endogenous and/or exogenous factors.[2] Biologically related early losses have been calculated on a sample of 16,935 Branemark implants and have been found to be 3.6%.[5] Many of the early failures can probably be explained by improper surgical technique.[2]

Mellonig *et al*[6] categorize implant failures as infectious failure (peri-implantitis) and traumatic failure (retrograde peri-implantitis). Most early failures are characterized by infectious signs (pus, wound dehiscence, fistula, swelling), periapical bone rarefaction, dense inflammatory cell infiltrate, necrotic bone, epithelial proliferation, and presence of

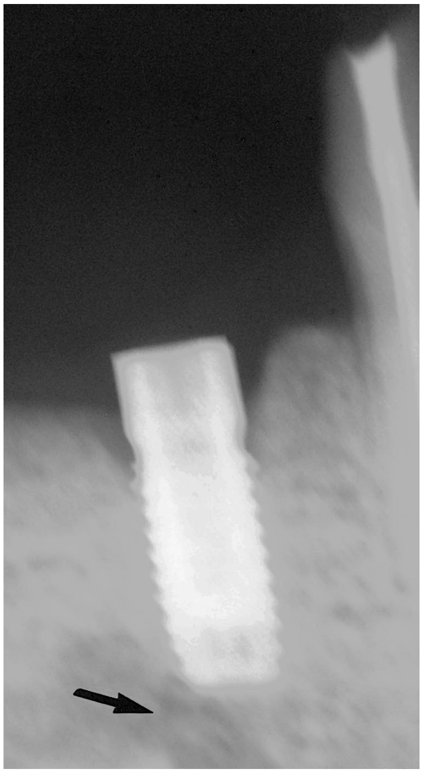

Figure 1. Periapical radiography. A periapical radiolucency is present at the apex of the implant (arrow).

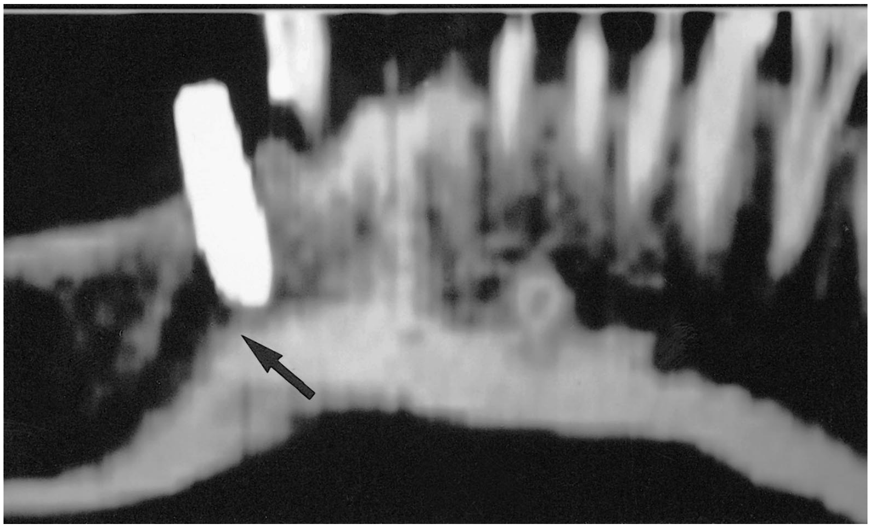

Figure 2. CT scan. A radiolucent lesion is present in a periapical location (arrow).

bacteria.[5] Peri-implant apical radiolucencies have been reported with a prevalence of 0.26%.[5] These lesions are found usually around long implants placed in dense bone and have been called implant periapical lesions.[5,7–10] Radiographically, the coronal portion of the implant is supported by normal bone in intimate contact with a stable implant.[5] The etiology of these lesions is unknown but seems to be multifaceted.[5]

Bone overheating, absence of primary implant stability, reduced healing ability of the host, implant overloading, implant contamination during production or insertion, pre-existing bone infections, residual root particles and foreign bodies, and placement of an implant in an infected maxillary sinus have been implicated in the pathogenesis of this lesion.[3,5,7–10] Also, the possibility of transmitting a periapical infection from a tooth to a recently inserted implant must be kept in mind.[2] The aim of the present case report was to evaluate the clinical and microscopic aspects of a mandibular implant periapical lesion.

Case Report

A 49-year-old female patient underwent the placement of a screw-shaped titanium dental implant in the premolar region of the right mandible. No preexisting pathology of mandibular bone was present. Six months after implant insertion, the patient presented with a dull, persistent pain that tended to increase in severity and was resistant to analgesics. No fistula was present at a clinical intraoral examination. A periapical x-ray showed the presence of a radiolucency at the apical portion of the implant (Fig 1); also a CT scan confirmed the presence of the radiolucent image (Fig 2). Due to the pain persistence, the implant was removed. After implant removal, the pain disappeared completely.

The specimen was immediately fixed in 10% buffered formalin and processed to obtain thin ground sections with the Precise 1 Automated System (Assing, Rome, Italy).[11] The specimen was dehydrated in an ascending series of alcohols and embedded in a glycolmethacrylate resin (Technovit 7200 VLC, Kulzer, Wehrheim, Germany). After polymerization, the specimen was sectioned with a high-precision diamond disk at a thickness of about 150 μm and ground down to about 30 μm. After polishing, the slides were stained with acid fuchsin and toluidine blue and were observed under normal light in the Leitz Laborlux microscope (Leitz, Wetzlar, Germany).

Results

At low power magnification, it was possible to observe that bone and non mineralized tissues were present only in the most apical portion of the implant (Fig 3). In the bone, it was possible to observe the presence of lacunae empty of osteocytes (Fig 4). At higher magnification, demineralizing bone was present in some areas. In the most external portion of the apical fenestration, necrotic and almost completely demineralized bone was present; some multinucleated cells were observed near the titanium surface (Fig 5). Rarely, lymphocytes and granulocytes were present in the tissues surrounding the most apical portion of the implant. No evidence of bacteria was found.

Discussion

The complications of dental implants may be classified in the following ways[12]:

(1) compromised successful implant—presence of inflammation, hyperplasia, and fistula formation near a

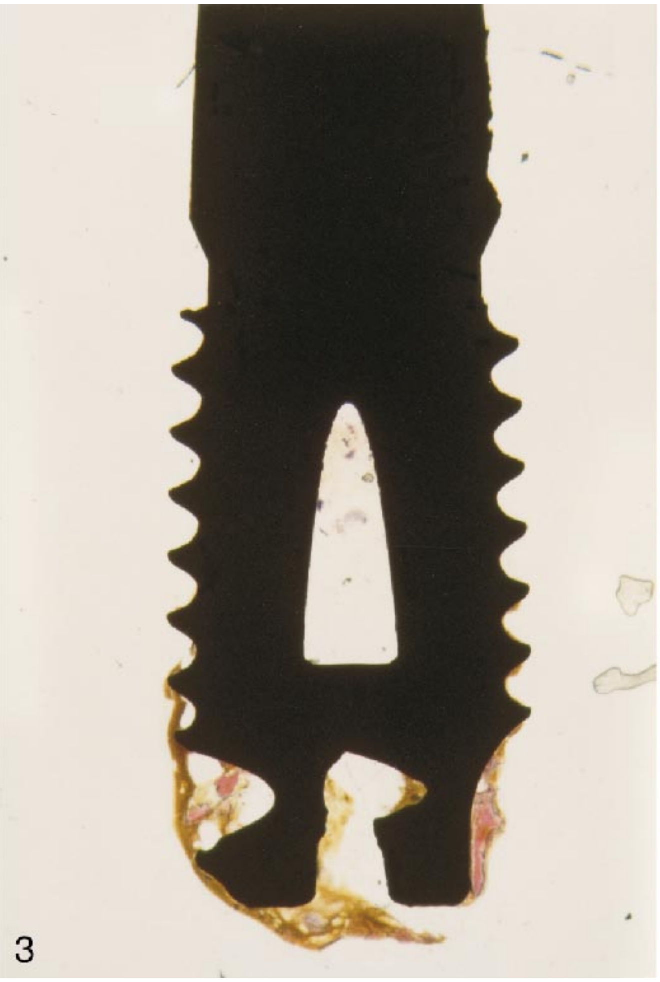

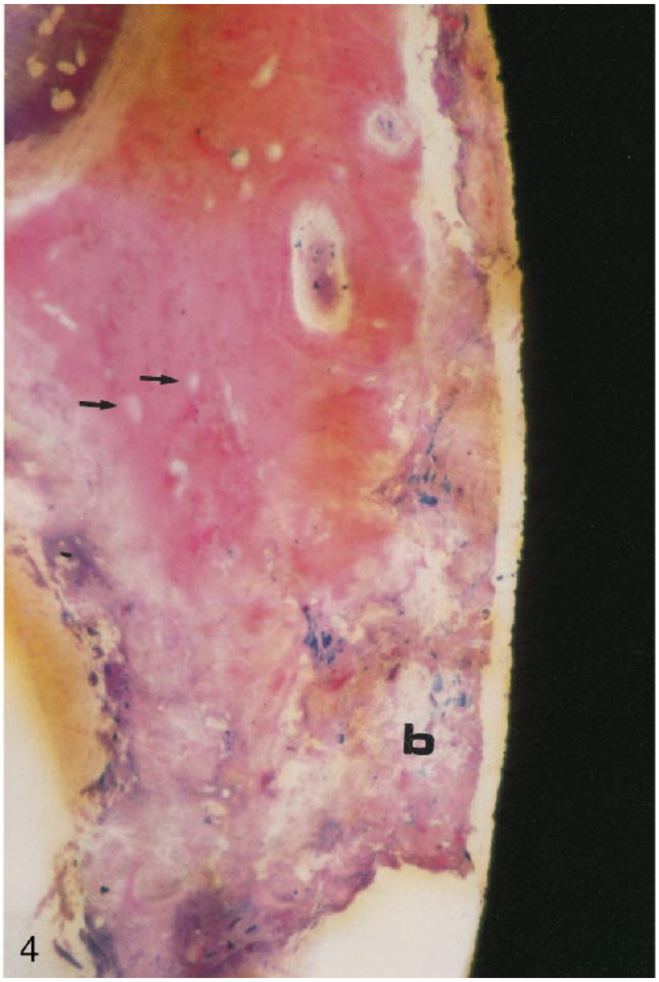

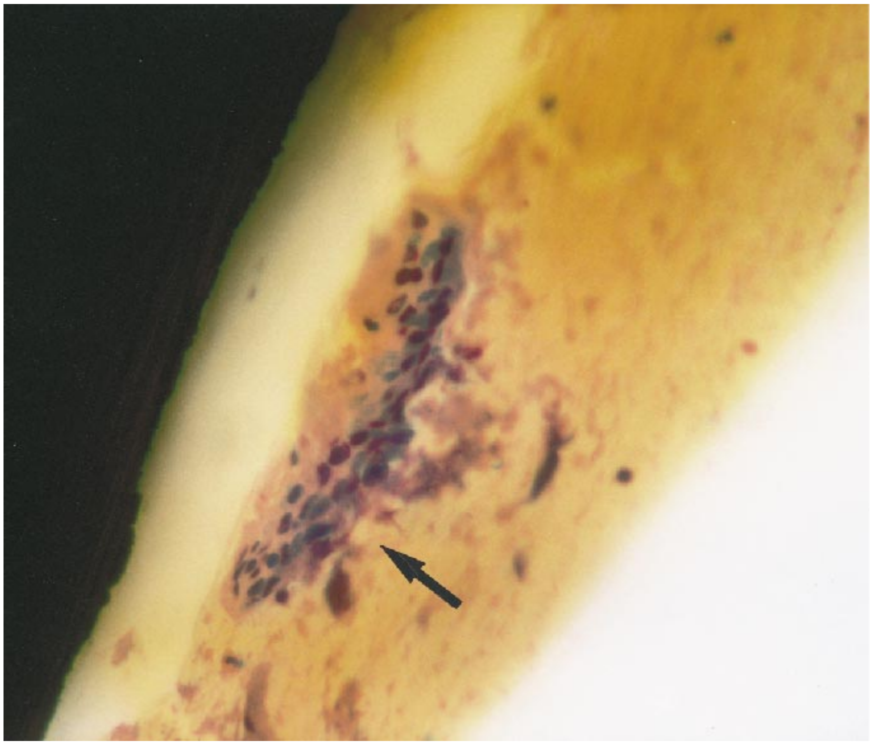

↑

FIGURE 3. Tissue is present in the most apical portion of the implant. Toluidine blue and acid fuchsin; ×12.

FIGURE 4. Empty osteocyte lacunae (arrows) and demineralizing bone (b) are present. Toluidine blue and acid fuchsin; ×50.

successfully osseointegrated implant;

(2) failing implant—progressive bone loss in a functional implant;
(3) failed implant—infection around a compromised implant.

The difference between failing and failed implants could be important be-

←

FIGURE 5. Multinucleated giant cell (arrow) near the implant surface. Toluidine blue and acid fuchsin; ×100.

cause a cause-related therapy could be attempted if a failing implant and its causes could be identified.[1] Complications can occur at any stage[13] in implant dentistry.

Mobility, marginal swelling and redness, bleeding and/or suppuration on probing, increased probing depth, peri-implant radiolucencies, and alveolar bone height loss characterize implant failures.[14] The loss of osseointegration is clinically manifested by a peri-implant radiolucency and implant mobility.[1] The loss of anchorage can be the result of surgical trauma, contamination, or overload.[13] Failure to osseointegrate may be caused by overinstrumentation of the bone producing inadequate implant immobilization or to inadequate implant length.[15] Implant periapical lesions may be active or inactive.[7] The latter may be considered similar to the periapical scar, shows no clinical symptoms, and may result from a residual bone cavity created by placing shorter implants than the implant site, from a heat-induced aseptic bone necrosis, or from an implant apex placed near an existing scar; the active or infected lesion, on the other hand, often tends to increase in size, be symptomatic, and result in fistula formation.[1,3,16,17]

It has been suggested that implant periapical lesions arise from a contaminated implant placed in a site with the presence of necrotic bone.[7] The remaining natural teeth can act as a reservoir of bacteria with involvement of the peri-implant tissues.[18] According to Sussman,[19] two main pathways of periapical implant pathology exist, namely, (1) implant to tooth, when the insertion of an implant produces a tooth devitalization; and (2) tooth to implant, when a periapical lesion from a nearby tooth encroaches upon the implant and contaminates it.

In our patient, the clinical and histologic features could suggest the following etiopathologic hypotheses.

(1) *Overloading of the implant.* The implant had not been loaded.

(2)*Excessive tightening of the implant with compression of the bone chips.* An excessive in-depth positioning of the implant could have caused a compression of the bone chips produced during the bone site preparation with subsequent ischemia, necrosis, and formation of a bone sequestrum. No compressed bone chips were, however, present in the apical portion of the implant.

(3) *Bone overheating during surgery.* Some of the observed histologic features could point to the occurrence of bone overheating during implant placement.

(4) *Fenestration of the vestibular bone.* A fenestration of the vestibular bone was not present clinically; if the cortical bone was thinner than 0.5 mm, bone remodeling could have produced a cortical bone dehiscence with infection of the soft tissues.

(5) *Presence of pre-existing bone pathology.* No periapical bone pathology was present before implant placement.

(6) *Contamination of the implant surface.* The presence of multinucleated cells near the implant surface could point to a contamination of the apical part of the implant.

(7) *Poor quality of the bone site.* The scarcity of osteoprogenitor cells due to poor bone quality at the surgical site can have had, most probably, a negative influence on the formation of mineralized tissues around the implant.

In conclusion, the most probable cause of the occurrence of the periapical pathosis in our patient was a contamination of the apical portion of the implant.

Treatment of an implant periapical lesion can be difficult.[20] Thorough curettage of the infected site with complete removal of all granulation tissues must be obtained. In some instances, resection of infected implant apices may be realized to facilitate adequate access to attain a complete debridement of the affected tissues.[5] In some cases, an extraoral surgical approach may be necessary.[5]

ACKNOWLEDGMENTS

This work was partially supported by the National Research Council (CNR), Rome, Italy, and by the Ministry of University, Research, Science, and Technology (MURST), Rome, Italy.

REFERENCES

1. Esposito M, Hirsch JM, Lekholm U, Thomsen P. Biological factors contributing to failures of osseointegrated oral implants. I: success criteria and epidemiology. *Eur J Oral Sci.* 1998;106:527–551.
2. Esposito M, Hirsch JM, Lekholm U, Thomsen P. Biological factors contributing to failures of osseointegrated oral implants. II: etiopathogenesis. *Eur J Oral Sci.* 1998;106:721–764.
3. Esposito M. *On Biological Failures of Osseointegrated Oral Implants* [dissertation]. Gothenburg: University; 1999.
4. El Askary AS, Meffert RM, Griffin T. Why do implants fail? Part I. *Implant Dent.* 1999;8:173–185.
5. Esposito M, Hirsch J, Lekholm U, Thomsen P. Differential diagnosis and treatment strategies for biologic complications and failing oral implants: a review of the literature. *Int J Oral Maxillofac Implants.* 1999;14:473–490.
6. Mellonig JT, Griffiths G, Mathys E, Spitznagel J. Treatment of the failing implant: case reports. *Int J Periodont Rest Dent.* 1995;15:385–395.
7. Reiser GM, Nevins M. The implant periapical lesion: etiology, prevention and treatment. *Compendium.* 1995;16:768–777.
8. Piattelli A, Scarano M, Piattelli M. Abscess formation around the apex of a maxillary root form implant: clinical and microscopical aspects. A case report. *J Periodontol.* 1995;66:899–903.
9. Piattelli A, Scarano A, Balleri P, Favero GA. Clinical and histological evaluation of an active ''implant periapical lesion.'' A case report. *Int J Oral Maxillofac Implants.* 1998;13:713–716.
10. Piattelli A, Scarano A, Piattelli M, Podda G. ''Implant periapical lesion.'' Clinical, histological and histochemical aspects. A case report. *Int J Periodont Res Dent.* 1998;18:181–187.
11. Piattelli A, Scarano A, Quaranta M. High-precision, cost-effective system for producing thin sections of oral

tissues containing dental implants. *Biomaterials.* 1997;18:577–579.
12. Newman MG, Flemmig TF. Bacteria–host interaction. In: Worthington P, Branemark PI, eds. *Advanced Osseointegration Surgery.* Berlin: Quintessence; 1992 [As reported by Spiekerman H. *Implantology.* Stuttgart: Thieme; 1995; 321.]
13. Tolman DE, Laney WR. Tissue-integrated prosthesis complications. *Int J Oral Maxillofac Implants.* 1992;7:477–484.
14. Rosenberg ES, Torosian JP, Slots J. Microbial differences in 2 clinically distinct types of failures of osseointegrated implants. *Clin Oral Implant Res.* 1991;2:135–144.
15. Zarb GA, Schnitt A. The longitudinal clinical effectiveness of osseointegrated implants: the Toronto study. Part I: surgical results. *J Prosthet Dent.* 1990;63:451–457.
16. Esposito M, Thomsen P, Molne J, Gretzer C, Ericson LE, Lekholm U. Immunohistochemistry of soft tissues surrounding late failures of Branemark implants. *Clin Oral Implant Res.* 1997;8:352–366.
17. Esposito M, Thomsen P, Ericson LE, Lekholm U. Histopathologic observations on early implant failures. *Int J Oral Maxillofac Implants.* 1999;14:798–810.
18. Quirynen M, Listgarten MA. The distribution of bacterial morphotypes around natural teeth and titanium implants ad modum Branemark. *Clin Oral Implant Res.* 1990;1:8–12.
19. Sussman HI. Periapical implant pathology. *J Oral Implantol.* 1998;24:133–138.
20. Balshi TJ, Pappas CE, Wolfinger GJ, Hernandez RE. Management of an abscess around the apex of a mandibular root form implant: clinical report. *Implant Dent.* 1994;3:81–85. ■

J Oral Implantol
2000;26:113

COMMENTARY

Implant Periapical Lesion: A Clinical and Histologic Case Report

A. Norman Cranin, DDS

This is an interesting report, which describes a not unknown phenomenon. The references report on similar findings and, as recently as 1998, this *Journal* published a paper on the subject by Sussman.

Dr Piattelli and colleagues call this a "new" pathology, which, of course, it is not. Their own paper cites cases that go back 7 years, and I recall treating an implant periapical lesion as early as 1988.

None of the etiologies tentatively offered have been verified, and although significant authorities have been referred to, somehow not all of them seem to be convincing. It is understandable that any of the suggested causes might be responsible for total implant failure but not for the singularly discreet apical lesion. Conceivably, residual root particles or foreign bodies could be considered, but if so, they'd be found on biopsy.

From a pragmatic point of view, what appears to be the most logical reason for the etiology of root-form periapical lesions is the accidental implantation of gingival epithelial cells. These cells would serve as a free graft and proliferate in the apical region, thus causing the lesions (Fig 1). The cause could be an improper incision or retraction, allowing some tissue to remain in the path of the implant drills, the recently renewed flapless approach described by Hahn and others, or the use of mini-implants (*eg,* Crête mince, Dentatus, etc), which often simply pierce the overlying gingivae en route to their bony host sites. If the reader will refer to Fig 4 in the article, at the very lowest portion of the micrograph (at 6 o'clock) there appears to be a cluster of epithelial cells.

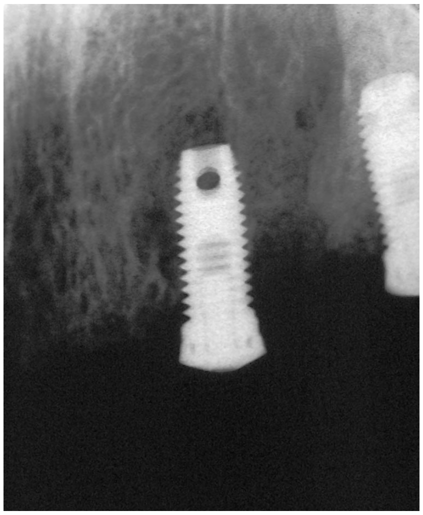

FIGURE 1. Cells proliferate in the apical region and cause lesions.

In regard to treatment, I have little doubt that the particular implant, which is described in this article, required removal. There are alternatives, however, to this approach. In the past, on one occasion, I was able to offer almost instant relief by simply fenestrating to the implant apex at the painful site. On another occasion, an apical exploration, curettage, and bone graft solved the problem.

I congratulate Dr Piattelli and his coauthors for this provocative presentation and hope that it will stimulate additional considerations from our readers.
