# Supplementary material for: A novel histopathological classification of implant periapical lesion: A systematic review and treatment decision tree
Source: PLoS One. 2022 Dec 22;17(12):e0277387. doi: 10.1371/journal.pone.0277387 (PMC9778521; doi:10.1371/journal.pone.0277387)
Supplement: S1 File — (ZIP) [file pone.0277387.s001.zip › support files/Included study/Silva 2010.pdf]

Journal of Oral Science, Vol. 52, No. 3, 491-494, 2010

Case Report

Unusual presentation of active implant periapical lesions: a report of two cases

Guilherme C. Silva[1)], Davidson R. F. Oliveira[2)], Tainah C. Vieira[1)], Cláudia S. Magalhães[1)] and Allyson N. Moreira[1)]

[1)]Department of Restorative Dentistry, School of Dentistry, Federal University of Minas Gerais, Belo Horizonte, MG, Brazil
[2)]Implant Dentistry Graduate Program, Department of Dentistry, Faculty of Administrative Studies (FEAD), Belo Horizonte, MG, Brazil

(Received 17 October 2009 and accepted 23 April 2010)

Abstract: Active implant periapical lesion (IPL) is a rare lesion which has been reported as one of the causes of dental implant failures. Usually, an affected implant shows radiolucency in the apical area, while remaining clinically stable. IPL is often accompanied by symptoms of pain, swelling, tenderness, and fistulation. In this paper, we describe two cases of IPL with very unusual findings which led to implant failure. A large IPL associated with an inflammatory cyst in the anterior maxilla, and a mandibular IPL resulting in an extra-oral fistula are presented. The etiology and treatment approaches for IPL are discussed. (J Oral Sci 52, 491-494, 2010)

Keywords: implant periapical lesion; implant failure; retrograde peri-implantitis; periapical cyst; cutaneous fistula.

Introduction

Active implant periapical lesion (IPL), or retrograde peri-implantitis, has been described as a radiolucent lesion that involves the apex of a clinically stable dental implant, while normal bone is often seen around its coronal portion (1-3). It is a rare event, occurring approximately in 0.3% to 1.8% of placed implants (1,2). It is normally accompanied by symptoms of pain, swelling, tenderness, and fistulation, and progression may cause implant failure (1-4). The etiology of IPL is multifactorial and can be related to bone overheating, bacterial contamination, implant overloading, excessive implant tightening, poor bone quality, presence of a pre-existing lesion, apical periodontitis on a tooth adjacent to the implant, or exogenous contamination of the surgical site or of the implant surface (1-7). The diagnosis is strictly clinical, based on the clinical signs and symptoms and radiographic findings (3). Treatment approaches involve implant removal, apical resection, or lesion curettage followed (or not) by guided tissue regeneration (1-3,5,8).

The aim of this paper is to describe two cases of implant periapical lesions with very unusual presentation which caused implant failure. The possible etiology and treatment approaches of IPL are discussed.

Correspondence to Dr. Guilherme Carvalho Silva, Av Afonso Pena 4334, Belo Horizonte, MG, 30130-009, Brazil
Tel: +55-31-32250966
Fax: +55-31-32277970
E-mail: guilhermeccs@ufmg.br

Case Report

Case 1

A 45-year-old Caucasian man without relevant past medical history presented with a painless swelling in the alveolar mucosa above a single implant-supported prosthesis in the area of the maxillary left central incisor. The implant had been in function for 1 year. The patient also complained of nasal obstruction and respiratory difficulty. The tooth was lost 2 years before due to root fracture. After a 4-month healing period, an external hexagon implant (Osseotite, Biomet 3i, Palm Beach Gardens, FL, USA) was placed according to the manufacturer's instructions under profuse sterile saline irrigation in a non-infected dense bone site. After a submerged healing period of 6 months, the implant was uncovered and

restored with a metal-ceramic crown cemented on a personalized castable abutment (Gold UCLA, Biomet 3i). At the time of examination, no clinical mobility or increased probing depth was observed. Cone-beam computed tomography showed a large and well-circumscribed radiolucent area involving the apex and middle portion of the implant, penetrating into the nasal cavity (Fig. 1). Under local anesthesia and intravenous sedation, a mucoperiosteal flap was raised and a large cystic lesion could be seen involving the implant apex (Figs. 2A, 2B). The lesion and implant were completely removed (Fig. 3), and histological examination confirmed the diagnosis of periapical inflammatory cyst. Healing was uneventful. Three months later, the patient underwent autologous bone graft augmentation to prepare the site for another implant placement.

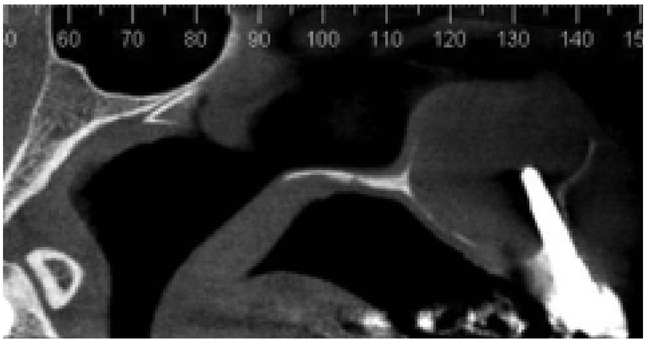

Fig. 1 Sagittal scan of cone-beam computed tomography (CT) showing a cystic area involving the implant apex.

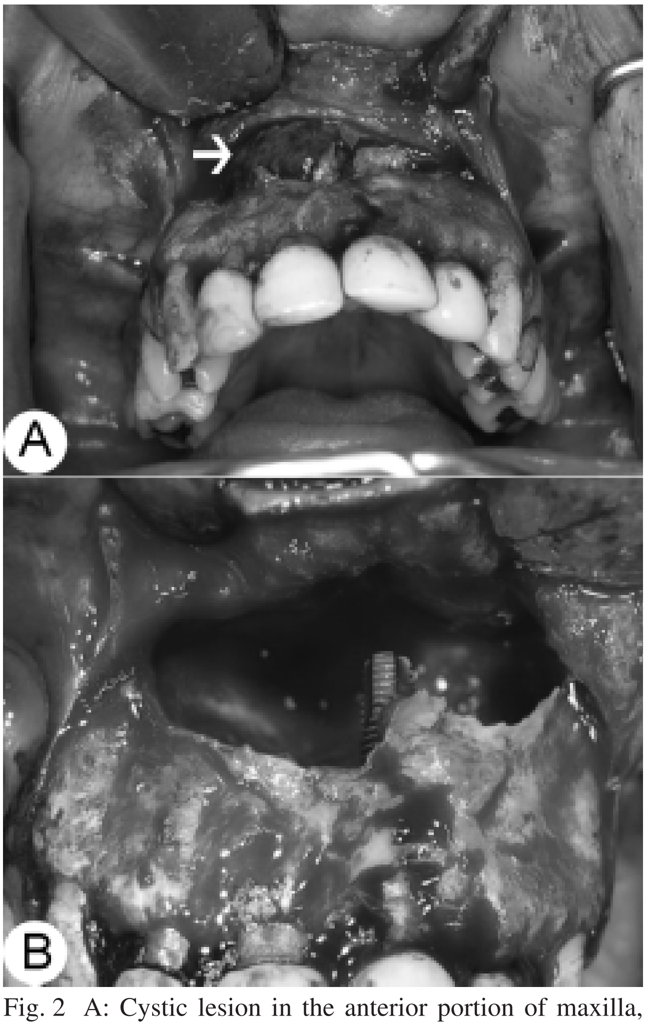

Fig. 2 A: Cystic lesion in the anterior portion of maxilla, penetrating into the nasal cavity (arrow). B: Implant apex inside the nasal cavity after cyst removal.

Case 2

A 38-year-old woman without any relevant past medical history attended our clinic for implant-retained (overdenture) prosthetic rehabilitation. Two immediate external hexagon implants (Osseotite, Biomet 3i) were placed according to the manufacturer's instructions under profuse sterile saline irrigation in the anterior mandibular area 4 months earlier. The immediate placed implants were stable due to high insertion torque in dense bone. Extraction of the anterior mandibular teeth had been carried out due to advanced periodontal disease. The patient had no complaints during the submerged healing time. A panoramic radiograph showed radiolucencies involving the apex of both implants (Fig. 4A). Healing abutments were connected to the implants when no clinical mobility and increased probing depth were detected, but the patient was warned about the poor prognosis of the implants. The patient returned 3 months later, reporting that one implant had spontaneously exfoliated, and a chronic suppurating fistula had emerged in the submental region for 1 month (Fig. 4B). A new panoramic and periapical radiograph showed a larger radiolucency around the remaining implant

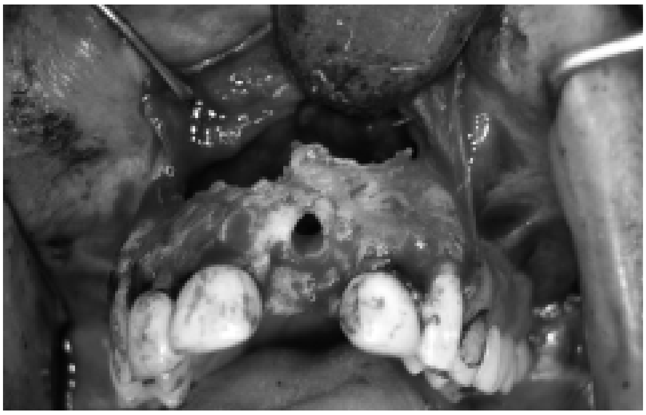

Fig. 3 Cavity after implant removal. Note healthy coronal bone.

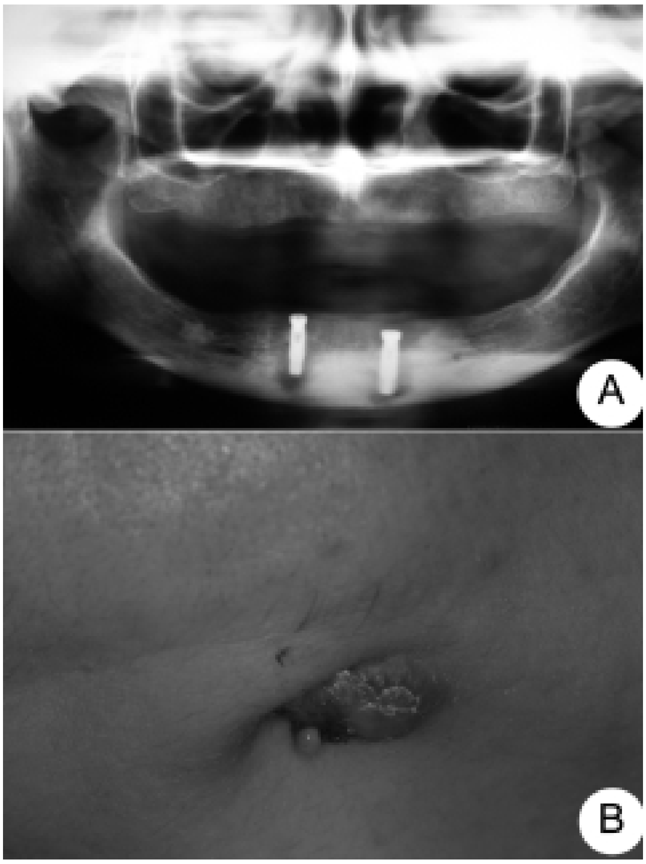

Fig. 4 A: Initial panoramic radiograph showing radiolucencies involving the apex of the two implants. B: Suppurating cutaneous fistula in the submental region.

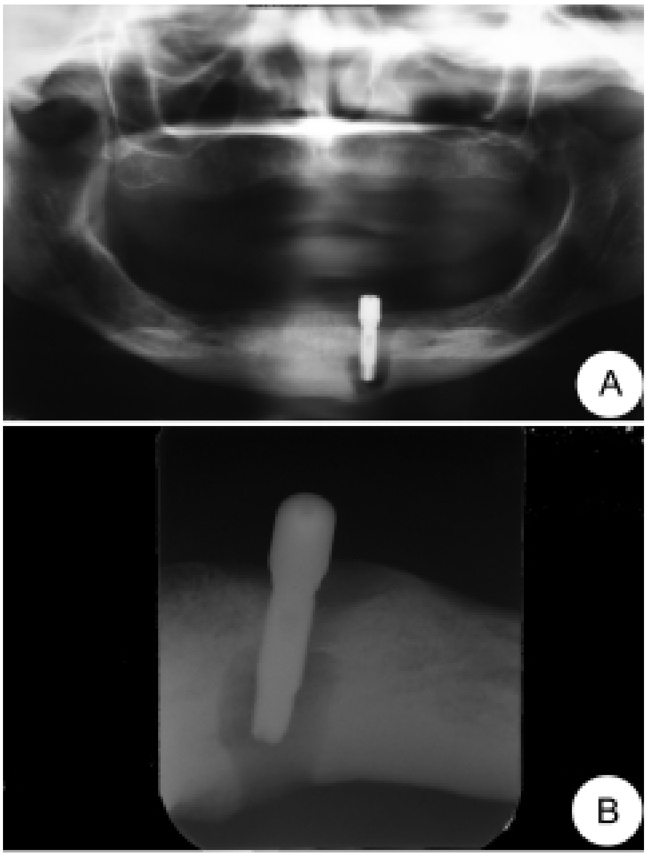

Fig. 5 A: Panoramic radiograph showing radiolucency involving the implant apex, reaching the mandibular base. D: Periapical radiograph.

compared with the previous image (Figs. 5A, 5B). Under local anesthesia, the implant was removed and the site was carefully curetted under profuse sterile irrigation. Healing was uneventful. After 4 months, two external hexagon implants (Osseotite, Biomet 3i) were placed in the region of the mandibular canines, in completely healed bone, and after a submerged healing period of 4 months, a ball-retained overdenture was delivered to the patient.

Discussion

Complications and failures in dental implant treatment may occur at any stage of the treatment. Active implant periapical lesion has been reported as one of the early causes of dental implant failures (1-3). Usually, IPL diagnosis is made based on the clinical and radiographic observations (3), as performed in case 2. However, histological analysis of any material retrieved from surgery is recommended when an acute inflammatory infiltrate is expected (3), as in case 1. Active IPL must be distinguished from the inactive form. Radiographically, the inactive implant periapical lesion appears similar to the active form, but it shows no clinical symptoms and requires only clinical and radiographic follow-up. The lesion may result from heat-induced aseptic bone necrosis, when placing implants shorter than the site prepared, or placing them in a pre-existing bone scar (1). The etiopathogenesis of active IPL remains controversial, and it is believed to have a multi-factorial origin (3-5). Reported cases suggest that IPL can result from bacterial contamination (1), presence of a pre-existing endodontic or cystic lesion (2,7), bone overheating (4), excessive implant tightening (4), poor bone quality (4), apical periodontitis on a tooth adjacent to the implant (5), or exogenous contamination of the surgical site or implant surface (4,6). In case 1, clinical aspects, treatment history, image analysis and histological examination suggested that the periapical cyst was caused by implant penetration into the nasal cavity. The implant was placed in a healed non-infected site adjacent to healthy teeth, was stable during 1 year of function, and showed no increased probing depth. Then, the most likely cause of the lesion is the implant extension into the nasal cavity. However, clinical studies demonstrated that the insertion of implants some

millimeters into the sinus or nasal cavity is well tolerated, without reports of cyst development (9). In case 1, it was found that about two thirds of the implant was penetrating into the nasal cavity. In case 2, the implants were inserted immediately after the extraction of infected periodontally compromised teeth. Although the implants were stable and showed normal bone in the cervical portion 4 months after surgical placement, the lesions rapidly increased, leading to an extra-oral fistula and implant failure. These characteristics suggest that the reported implant periapical lesions were related to pre-existing bacterial contamination. Most likely, effective alveolar debridement and decontamination were not carried out before the implant placement. The immediate placement of implants into infected sockets may not necessarily be contraindicated if appropriate clinical procedures such as careful debridement and cleaning are performed. A recent study showed favorable results when implants were inserted into debrided infected sockets (10). However, the cases must be carefully selected and active suppurating sites must be avoided.

Some therapies have been reported to treat IPL, including lesion excision or debridement, with or without bone grafting (2,3), antibiotics (2,6), implant apicoectomy (5,6,8), and implant removal (4,7). When possible, the treatment should preserve a stable implant. A study which analyzed a treatment protocol consisting of lesion debridement, implant apicoectomy and administration of topical and systemic antibiotics showed 97.4% of success after 4.5 years in 39 cases of IPL (8). One serial case study reported favorable initial results when periapical surgery with curettage and chlorhexidine irrigation was performed in rapidly diagnosed IPL (3). Some factors should be taken into consideration to determine appropriate treatment, including size of the lesion, implant stability, bone anchorage, peri-implant probing depth, the status of adjacent teeth, implant position, and the type and quality of the prosthetic rehabilitation (6). Because of the large extension of bony destruction caused by the actively progressing implant lesions, a more conservative treatment option with preservation of the implant could not be performed in the presented cases. Early diagnosis is desired to treat the lesion and prevent the need for implant extraction (3).

To prevent the occurrence of IPL, some basic surgical recommendations must be followed, such as stringent aseptic measures, careful anatomical and surgical preplanning and adequate debridement of sockets in immediate implant placement.

References

1. Reiser GM, Nevins M (1995) The implant periapical lesion: etiology, prevention and treatment. Compend Contin Educ Dent 16, 768, 770, 772.
2. Quirynen M, Vogels R, Alsaadi G, Naert I, Jacobs R, van Steenberghe D (2005) Predisposing conditions for retrograde peri-implantitis, and treatment suggestions. Clin Oral Implants Res 16, 599-608.
3. Peñarrocha-Diago M, Boronat-Lopez A, García-Mira B (2009) Inflammatory implant periapical lesion: etiology, diagnosis, and treatment–presentation of 7 cases. J Oral Maxillofac Surg 67, 168-173.
4. Piattelli A, Scarano A, Piattelli M, Podda G (1998) Implant periapical lesions: clinical, histologic, and histochemical aspects. A case report. Int J Periodontics Restorative Dent 18, 181-187.
5. Rosendahl K, Dahlberg G, Kisch J, Nilner K (2009) Implant periapical lesion. A case series report. Swed Dent J 33, 49-58.
6. Nedir R, Bischof M, Pujol O, Houriet R, Samson J, Lombardi T (2007) Starch-induced implant periapical lesion: a case report. Int J Oral Maxillofac Implants 22, 1001-1006.
7. Casado PL, Donner M, Pascarelli B, Derocy C, Duarte ME, Barboza EP (2008) Immediate dental implant failure associated with nasopalatine duct cyst. Implant Dent 17, 169-175.
8. Balshi SF, Wolfinger GJ, Balshi TJ (2007) A retrospective evaluation of a treatment protocol for dental implant periapical lesions: long-term results of 39 implant apicoectomies. Int J Oral Maxillofac Implants 22, 267-272.
9. Jung JH, Choi BH, Jeong SM, Li J, Lee SH, Lee HJ (2007) A retrospective study of the effects on sinus complications of exposing dental implants to the maxillary sinus cavity. Oral Surg Oral Med Oral Pathol Oral Radiol Endod 103, 623-625.
10. Casap N, Zeltser C, Wexler A, Tarazi E, Zeltser R (2007) Immediate placement of dental implants into debrided infected dentoalveolar sockets. J Oral Maxillofac Surg 65, 384-392.
