# Supplementary material for: A novel histopathological classification of implant periapical lesion: A systematic review and treatment decision tree
Source: PLoS One. 2022 Dec 22;17(12):e0277387. doi: 10.1371/journal.pone.0277387 (PMC9778521; doi:10.1371/journal.pone.0277387)
Supplement: S1 File — (ZIP) [file pone.0277387.s001.zip › support files/Included study/Sivolella 2013.pdf]

MINERVA STOMATOL 2013;62:235-9

# Nasopalatine duct cyst as a complication of dental implant placement: a case report

S. SIVOLELLA [1], M. VALENTE, [2], E. GASPARINI [3], E. STELLINI [4]

[1]*Institute of Clinical Dentistry*
*University of Padua, Padua, Italy*
[2]*Section of Special Pathology*
*Department of Cardiological Thoracic and Vascular Sciences*
*University of Padua, Padua, Italy*
[3]*Institute of Clinical Dentistry*
*Azienda Ospedaliera/*
*University of Padua, Padua, Italy*
[4]*Department of Neurosciences*
*Neurological, Psychiatric, Sensorial Reconstructive and Rehabilitative Sciences*
*University of Padua, Padua, Italy*

**This report demonstrates the association between the development of a nasopalatine duct cyst and implant surgery, involving 2 implants positioned 4 years after teeth extraction at a site unaffected by any prior local endodontic disease or radiolucency. The cyst was removed and the residual void was filled with deproteinized bovine bone. Two-year follow-up showed no cyst recurrence, the normal anatomy was partly restored, and one of the implants showed clinical signs of re-osseointegration.**

**Key words:** Dental implants - Cysts - Osseointegration.

Nasopalatine duct cysts (NPDC) are believed to result from spontaneous degeneration and proliferation of remnants of the nasopalatine duct or mucus cells in the incisive canal.[1] Dental implant surgery is reportedly one of the possible irritative and traumatic causes of this condition.[2, 3]

## Case report

A 70-year-old female was referred with tumefaction of the upper anterior buccal arch, where two dental implants had been placed at the sites of the upper right central and lateral incisors, each supporting a single crown. The implants seemed stable. They had been inserted five years earlier, approximately 4 years after the teeth had been extracted.

An orthopantomogram obtained prior to placing the implants showed no signs of radiolucency (Figure 1). A new orthopantomogram and CT scan (Figure 2) revealed a large, roundish radiolucent lesion with clear margins in the anterior maxilla region. The lesion involved the two implants, the nasal floor, the buccal alveolar wall and the nasopalatine duct, and reached the apex of the upper left central incisor, which was still vital. The size of the lesion along its greater axis was approximately 2 cm.

The cyst was accurately isolated and removed. The nasopalatine nerve was isolated and perineural soft tissue of unclear origin was removed. The implants were not displaced or damaged, their surface was cleansed with sterile physiological solution, and the cavity was filled with a bone substitute (Bio-Oss®, Geistlich Pharm, AG Wolhusen, Switzerland), covering the buccal side of the implants, and a collagen membrane (BioGide® Geistlich) was applied. Microscopic examination showed that the cyst was covered with a layer of epithelium comprising three epithelial cell types, *i.e.*, ciliated columnar (respiratory), cuboidal, and non-keratinised stratified squa-

Corresponding author: S. Sivolella, DDS, University of Padua, Institute of Clinical Dentistry, via Venezia 90, 35138 Padua, Italy. E-mail: stefano.sivolella@libero.it

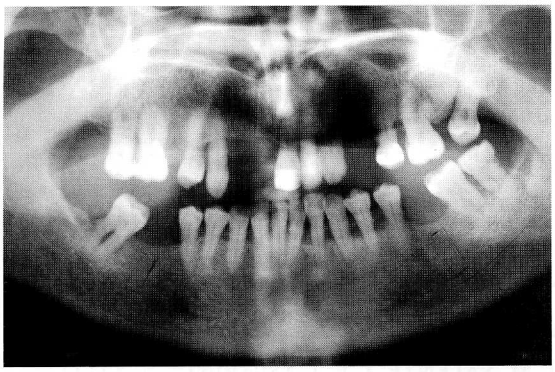

Figure 1.—Preimplant orthopantomogram: the upper right central and lateral incisors are missing and there is no evidence of radiolucent lesions.

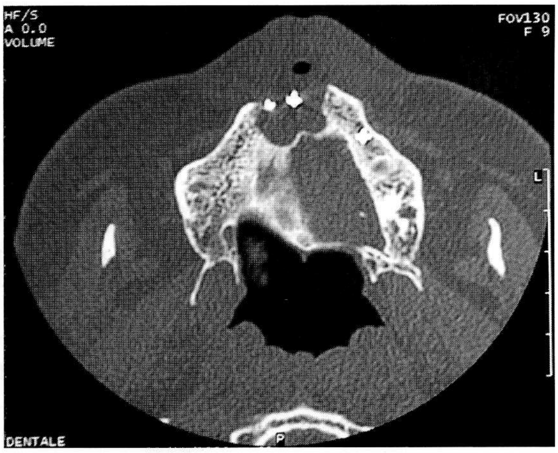


Figure 2.—Preoperative CT. Axial image of the region corresponding to the upper right central and lateral incisors.

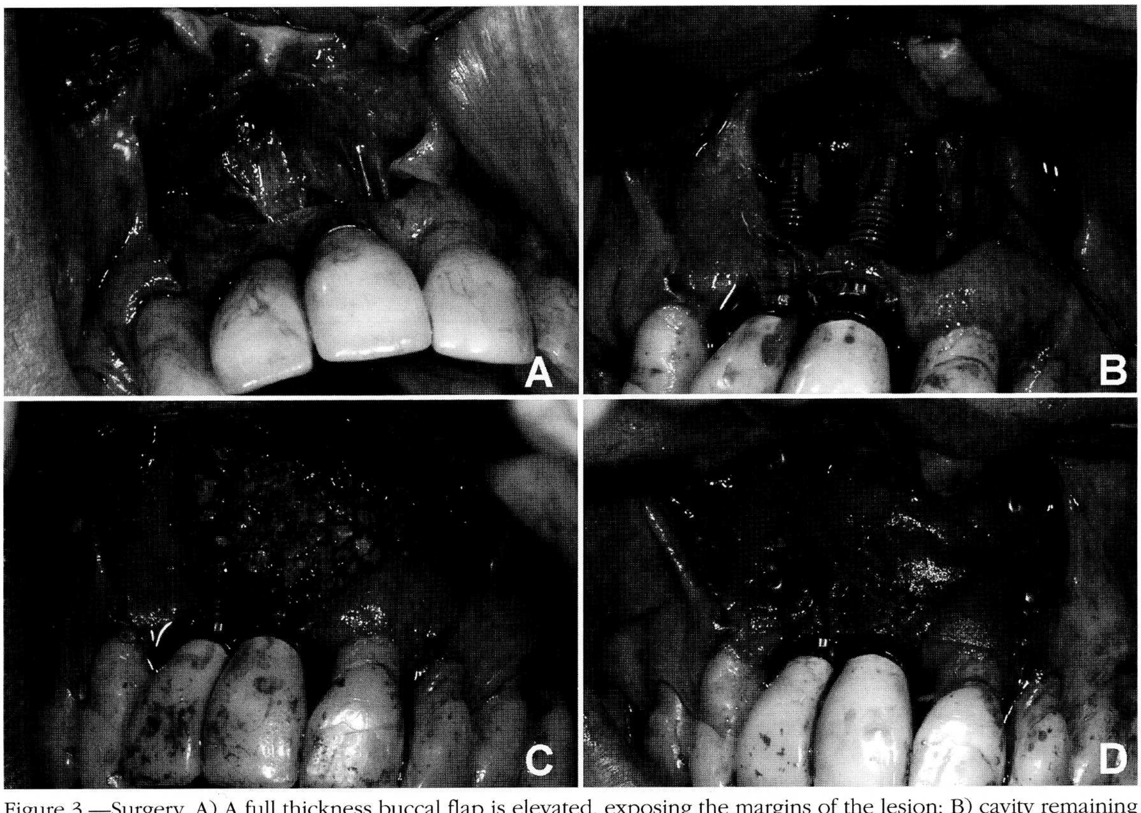


Figure 3.—Surgery. A) A full thickness buccal flap is elevated, exposing the margins of the lesion; B) cavity remaining after removing the cyst and isolating the nasopalatine nerve; C) BioOss® graft; D) BioGide® membrane fixed with 4 titanium pins (Frios-membrane pins, Friadent, Mannheim, Germany).

mous epithelium. The cyst wall exhibited a prominent component of blood vessels and peripheral nerves, and there was a diffuse lymphocytic infiltrate. These histological, clinical and radiological features prompted the diagnosis of NPDC. A month after surgery, the implant at the site of the upper right central incisor was lost and there was evidence of mild wound dehiscence and loss of part of the bone graft.

Twelve months after surgery, the implant at the

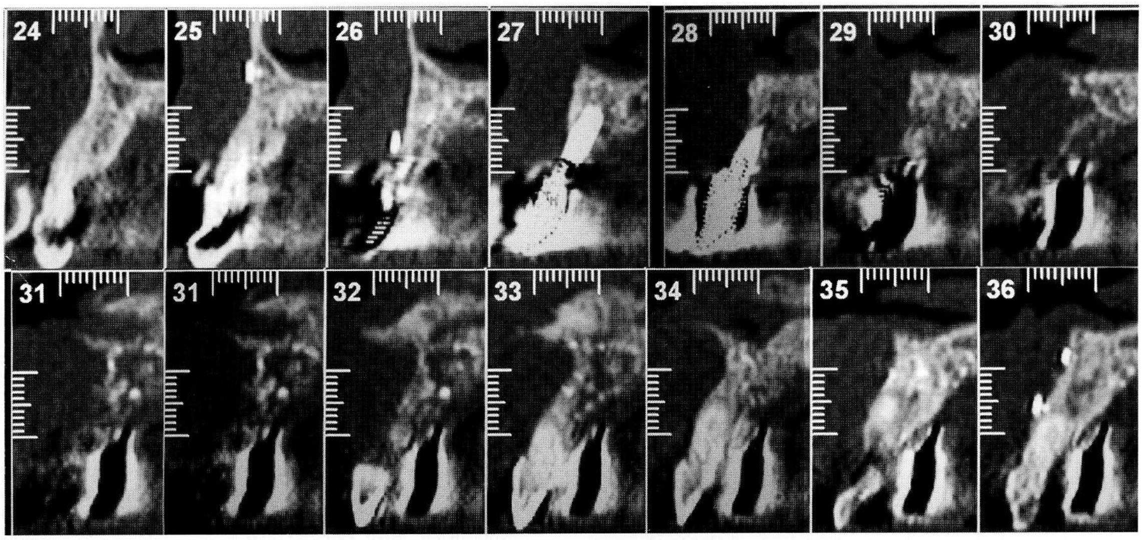


Figure 4.—Radiographic follow-up 12 months after surgery: CT, paraxial views.

site of the lateral incisor was stable. Follow-up CT scan at one year (Figure 3) revealed a radio-opaque material up against the remaining implant, on the buccal and palatal sides. There was evidence of poorly-organised radiolucent areas in the area of the central incisor. The structure of the bone appeared to return to normal towards the left central incisor and the floor of the nasal cavity. There were no radiological signs of any recurrent cystic disease. At two-year follow-up, there was no evidence of any sequelae or recurrence and the implant remained stable.

## Discussion

Casado *et al.*,[2] described a case of NPDC secondary to the insertion of an implant immediately after the extraction of a tooth, in an alveolus contaminated by prior endodontic infectious disease. McCrea [3] published a case of NPDC as a late complication of successful dental implant placement, but it is impossible to say whether the NDPC related to the implant surgery due to the lack of any clinical or radiological documentation to demonstrate that the patient had no endodontic or periodontal disease at the time of the implant's placement. In our case, the two implants had been inserted 4 years after the natural teeth had been extracted (due to periodontal problems, according to the patient).

The surgical treatment adopted in this case is one of the recommended procedures for treating maxillary cysts, periapical implant lesions and to try to obtain the implant reosseointegration.[1, 2, 4-6]

Renvert *et al.*[6] conducted a systematic review on re-osseointegration on previously contaminated dental implants surfaces. One of the main finding was that the selection of a regenerative procedure might be more important than the selection of an implant surface decontamination, such as mechanical debridement, the use of chemical agents (*e.g.*, chlorhexidine, citric acid, hydrogen peroxide, delmopinol) or laser. Overall the best results for re-osseointegration tended to occur when a combination of both GBR and graft material was used, irrespective of the grafting material.

Several surgical methods have been used in an effort to avoid the risk of lesions affecting dental implants in the anterior maxilla, including removal of the contents of the incisive canal associated with a bone graft or implant placement.[7-10]

## Conclusions

According to Casado *et al.*[2] e McCrea,[3] implant surgery may be correlated to the

development of a NPDC. This event has to be considered when performing implant surgery in the anterior maxilla. Conservative treatment with bone regeneration can be used to restore the oral anatomy and enable the re-osseointegration of the implant(s) involved.

## References


1. Suter VG, Altermatt HJ, Voegelin TC, Bornstein MM. The nasopalatine duct cyst: epidemiology, diagnosis and therapy. Schweiz Monatsschr Zahnmed. 2007;117:824-39.
2. Casado PL, Donner M, Pascarelli B, Derocy C, Duarte ME, Barboza EP. Immediate dental implant failure associated with nasopalatine duct cyst. Implant Dent 2008;17:169-75.
3. McCrea SJ. Nasopalatine duct cyst, a delayed complication to successful dental implant placement: Diagnosis and surgical management. J Oral Implantol 2012 [Epub ahead of print].
4. Peñarrocha DM, Boronat LA, García MB. Inflammatory implant periapical lesion: etiology, diagnosis, and treatment - presentation of 7 cases. J Oral Maxillofac Surg 2009;67:168-73.
5. Peñarrocha DM, Sanchis BJM. Surgical treatment and follow-up of solitary bone cyst of the mandible: a report of seven cases. Br J Oral Maxillofac Surg 2001;39:221-3.
6. Renvert S, Polyzois I, Maguire R. Re-osseointegration on previously contaminated surfaces: a systematic review. Clin Oral Impl Res 2009;20:216-27.
7. Artzi Z, Nemcovsky CE, Bitlitum I, Segal P. Displacement of the incisive foramen in conjunction with implant placement in the anterior maxilla without jeopardizing vitality of nasopalatine nerve and vessels: a novel surgical approach. Clin Oral Implants Res 2000;11:505-10.
8. Scher EL. Use of the incisive canal as a recipient site for root form implants: preliminary clinical reports. Implant Dent. 1994;3:38-41.
9. Peñarrocha DM, Carrillo C, Uribe R, García B. The nasopalatine canal as an anatomic buttress for implant placement in the severely atrophic maxilla: a pilot study. Int J Oral Maxillofac Implants 2009;24:936-42.
10. Rosenquist JB, Nystrom E. Occlusion of the incisal canal with bone chips. A procedure to facilitate insertion of implants in the anterior maxilla. Int J Oral Maxillofac Surg 1992;21:210-1.


*Conflicts of interest.*—The authors certify that there is no conflict of interest with any financial organization regarding the material discussed in the manuscript.

Received on February 21, 2013.
Accepted for publication on June 26, 2013.

# Cisti del dotto naso palatino come complicanza del posizionamento di impianti dentali: un caso clinico

Si ritiene che le cisti del dotto naso-palatino siano il risultato di una degenerazione spontanea e proliferazione dei residui delle cellule del dotto naso-palatino o cellule mucose nel canale incisivo [1]. È stato riportato che la chirurgia implantare dentale sia una delle possibili cause irritative e traumatiche di questa condizione [2, 3].

### Caso clinico

Una donna di 70 anni di età è stata valutata per una tumefazione dell'arcata anteriore superiore vestibolare, dove due impianti dentali, ciascuno supportante una corona singola, erano stati posizionati nei siti degli incisivi superiori centrali e laterali di destra. Gli impianti apparivano stabili. Essi erano stati inseriti cinque anni prima, circa 4 anni dopo che i denti erano stati estratti.

Un'ortopantomografia ottenuta prima del posizionamento degli impianti non dimostrava alcun segno di radiotrasparenza (Figura 1). Una nuova ortopantomografia e tomografia computerizzata (Figura 2) rivelavano un'ampia lesione radiotrasparente rotondeggiante, con margini netti, a livello del mascellare anteriore superiore. La lesione coinvolgeva i due impianti, il pavimento del naso, la parte alveolare vestibolare ed il dotto nasopalatino, e raggiungeva l'apice dell'incisivo centrale superiore sinistro, che era ancora vitale. La dimensione della lesione lungo l'asse maggiore era circa 2 cm.

La cisti è stata accuratamente isolate e rimossa. Il nervo nasopalatino stato isolato ed il tessuto molle perineurale di origine incerta rimosso. Gli impianti non sono stati spostati o danneggiati, e la loro superficie è stata pulita con soluzione fisiologica sterile, e la cavità riempita con un sostituto osseo (Bio-Oss®, Geistlich Pharm, AG Wolhusen, Switzerland), che copriva gli impianti sul versante vestibolare, e una membrana collagene è stata poi applicata (Bio-Gide® Geistlich) (Figura 3). L'esame microscopico dimostrava che la cisti era coperta da uno strato di epitelio comprendente tre tipi cellulari, ovvero colonnare ciliato (respiratorio), cuboidale e epitelio stratificato non cheratinizzato. La parete cistica mostrava una componete prominente di vasi sanguigni e nervi periferici, e c'era un diffuso infiltrato linfocitario. Queste caratteristiche istologiche, cliniche e radiologiche hanno portato alla diagnosi di cisti del dotto nasopalatino. Un mese dopo la chirurgia, l'impianto nella sede dell'incisivo centrale superiore era perso, e c'era evidenza di una piccola deiescenza della mucosa e perdita di parte dell'innesto osseo.

Dodici mesi dopo la chirurgia, l'impianto nella sede dell'incisivo laterale era stabile. La TC di con-

trollo a un anno (Figura 4) rivelava materiale radioopaco a ridosso dell'impianto residuo, sui versanti vestibolare e palatale. C'era evidenza di aree radiotrasparenti poco organizzate nell'area dell'incisivo centrale. La struttura dell'osso aveva un aspetto tornato alla normalità a ridosso dell'incisivo superiore centrale sinistro e della cavità nasale. Non c'era alcun segno radiologico di recidiva della patologia cistica. Al follow-up a due anni, non c'era evidenza di alcuna sequela né recidiva e l'impianto rimaneva stabile.

## Discussione

Casado *et al.*[2] hanno descritto un caso di cisti del dotto nasopalatino secondario all'inserzione di un impianto immediatamente dopo l'estrazione del dente, in un alveolo contaminato da una lesione endodontica precedente. McCrea [3] ha pubblicato un caso di cisti del dotto nasopalatino come complicanza tardiva di un impianto posizionato con successo, ma non è possibile affermare se la cisti fosse in relazione alla chirurgia implantare, a causa della mancanza di alcuna documentazione clinica o radiologica che dimostrasse che il paziente non aveva alcuna patologia endodontica o parodontale al tempo dell'inserimento dell'impianto. Nel nostro caso, i due impianti erano stati inseriti 4 anni dopo che i denti naturali erano stati estratti (a causa di problemi parodontali, secondo la paziente).

Il trattamento chirurgico adottato in questo caso è una delle procedure raccomandate per trattare le cisti mascellari, le lesioni apicali peri-implantari e per cercare di ottenere la re-osteointegrazione implantare [1, 2, 4-6].

Renvert et al[6] hanno condotto una revisione sistematica sulla re-osteointegrazione su superfici implantari precedentemente contaminate. Uno dei principali risultati è stato che la selezione di una procedura rigenerativa possa essere più importante della selezione di un metodo di decontaminazione della superficie implantare, come la detersione meccanica o l'utilizzo di agenti chimici (es. clorexidina, acido citrico, acqua ossigenata, delmopinolo), o il laser. In generale, i migliori risultati per la re-osteintegrazione si sono verificati quando una combinazione di GBR e materiale da innesto era utilizzata, a prescindere dal tipo di materiale utilizzato.

Molti metodi chirurgici sono stati utilizzati per cercare di evitare il rischio di lesioni a carico degli impianti dentali nel mascellare superiore anteriore, inclusi la rimozione del contenuto del canale incisivo associato a innesto osseo o al posizionamento di un impianto [7-10].

In accordo con Casado *et al.*[2] e McCrea [3], la procedura implantologica può costituire una causa precipitante lo sviluppo di cisti del dotto naso palatino. Questa possibilità deve essere considerata nelle procedure implantologiche del settore anteriore mascellare. Il trattamento conservativo con rigenerazione ossea può essere applicato per ristabilire l'anatomia orale e favorire la re-osteointegrazione degli impianti coinvolti [6].

## Riassunto

Questo caso dimostra l'associazione tra lo sviluppo di una cisti del dotto naso palatino e la chirurgia implantare, coinvolgente 2 impianti posizionati 4 anni dopo le estrazioni dentarie in un sito non affetto da alcuna precedente patologia endodontica locale o radiotrasparenza. La cisti è stata rimossa e la cavità residua riempita con osso bovino deproteinizzato. Al controllo a due anni non ci sono stati segni di recidiva, la normale anatomia era parzialmente recuperata, ed uno degli impianti mostrava segni clinici di re- osteointegrazione.

PAROLE CHIAVE: Impianto dentale - Cisti - Osteointegrazione.
