# Supplementary material for: A novel histopathological classification of implant periapical lesion: A systematic review and treatment decision tree
Source: PLoS One. 2022 Dec 22;17(12):e0277387. doi: 10.1371/journal.pone.0277387 (PMC9778521; doi:10.1371/journal.pone.0277387)
Supplement: S1 File — (ZIP) [file pone.0277387.s001.zip › support files/Included study/Sun 2013.pdf]

Implant Failure Associated With Actinomycosis in a Medically Compromised Patient

Chun-Xiao Sun, DMD, PhD, MS, MSD*
Jeffrey M. Henkin, DDS, MS
Craig Ririe, DDS, MS
Elham Javadi, DDS

Oral actinomycosis is not a common disease, but it can cause massive destruction. This article reports a case of implant failure associated with actinomycosis. A 55-year-old Caucasian male patient had tooth #20 extracted years ago and an implant placed 3 years ago. The #20 implant area developed an abscess about 1½ years after implant placement. Radiographic findings revealed a large radiolucency on the mesial aspect of the #20 implant. The implant was surgically removed and the lesion thoroughly debrided. The patient experienced severe pain when the apical soft tissue was curreted following implant removal. A periapical radiograph revealed that the lesion approached the mental foramen. A short course of antibiotics was prescribed. Histological observation found sulfur granules, which were found to be actinomycotic colonies. Peri-implant actinomycosis was diagnosed. No recurrence had occurred at the 1-year follow-up.

Key Words: dental implant, implant failure, actinomycosis, case report

INTRODUCTION

Actinomycosis is a chronic infectious disease characterized by abscess formation, tissue fibrosis, and draining sinuses.[1] It is caused by gram-positive *Actinomyces* species, which have the morphological appearance of a fungus and occurs rarely in humans but more frequently in cattle as a disease called lumpy jaw.[2] The intertwining bacterial filamentous colonies are called "sulfur granules" because they appear as yellowish specks. The colonies are made of radiating filaments and have a starburst appearance. Because of this appearance, these colonies are often referred to as "ray fungus."[2]

Actinomycosis is considered to be of low pathogenicity, causing disease only at the site of a previous tissue injury, typically following dental treatment.[3] It has been reported that men 20 to 60 years of age are more susceptible to this infection. The occurrence of this infection is 4 times more prevalent in men than in women.[4] Actinomycosis could involve cervicofacial, pulmonary or pulmothoracic, or abdominal-pelvic regions.[5] Fifty-five percent of actinomycosis infections occur in the cervicofacial region.[1]

Although *Actinomyces* is part of the normal oral bacterial flora, cervicofacial actinomycosis is not a common infection. Predisposing factors include both systemic and local factors. Systemic factors are immunoincompetence, malignancy, or diabetes. Local factors could be tissue or bone trauma, poor oral hygiene, and deep caries.[1] Most cervicofacial actinomycoses are of odontogenic origin. They may result from dental treatment, trauma, or poor oral hygiene,[1] although non-odontogenic origin cases have been reported recently.[5]

The most common pathogenic species isolated from humans is *Actinomyces israelii*.[6,7] *A israelii* has been isolated from tonsils, dental plaque, periodontal pockets, and carious lesions. It also has been detected in stable orthodontic mini-implants but rarely found in failed implants.[8] *Actinomyces odontolyticus* was the far most prominent *Actinomyces*

Department of Periodontics, Loma Linda University School of Dentistry, Loma Linda, Calif.
* *Corresponding author,* e-mail: csun1@llu.edu
DOI: 10.1563/AAID-JOI-D-11-00028

species found in failed dental implants, which were present in 84% of *Actinomyces*-positive failed implants.[9]

Caries-induced infected root canals are believed to be the primary port of bacterial entry in periapical actinomycosis, one form of cervicofacial actinomycosis.[10] There are several case reports of periapical actinomycosis,[10–13] but no report has been documented for peri-implant actinomycosis. This article reports a case of dental implant failure due to actinomycosis and discusses its possible causes.

Case Report

In October 2009, a 55-year-old Caucasian male patient was referred back from a dental student for evaluation of implant #20, which had been placed about 2½ years previously. The patient's medical history included severe pneumonia, which had been treated with lung surgery and blood transfusion; hepatitis C, which was being treated with Pegasys and ribavirin; and herpes genitalis. No other significant medical condition was reported.

The patient's dental history included extraction of tooth 20 years ago and root canal treatment on tooth #19 in Mexico in early 2005. The patient came to our school for construction of a fixed partial denture on teeth #19–#21 about 4 months later. A dental student started to prepare these teeth and noticed not enough tooth structure was left for crown retention on the distal of tooth #19. The patient complained of pain, and the tooth was sensitive to percussion and biting after 3 weeks. A periapical lesion was noticed on the radiograph. After endodontic consultation and restorability evaluation, the tooth was deemed to have poor prognosis. The patient elected to have the tooth extracted and have an implant placed. The healing after the extraction of tooth #19 was uneventful. Tooth #18 developed sensitivity to cold after an MO amalgam replacement and required subsequent root canal treatment.

Prior to the implant placement, a panoramic radiograph and cone beam radiograph (data not shown) were taken to evaluate the bony condition, and no obvious lesions were noticed. Implants #19 and #20 (Nobel Biocare Replacement Select Straight 5 $\times$ 11.5 mm and 4.3 $\times$ 13 mm, respectively; Nobel Biocare, Zurich, Switzerland) were placed uneventfully in 2007 by a resident (Figure 1a). Implant crowns were placed 6 months later by a dental student.

At the following visit, about 1 month later, the dental student noticed an abscess on the marginal gingiva of implant #20 and sent the patient to a prosthodontist for an evaluation. A periapical radiograph was taken, and no obvious lesion was noticed. Chlorhexidine mouth rinse (Peridex, 3M ESPE, St Paul, Minn) was prescribed, and the patient was instructed to come back for reevaluation in 2 weeks. However, the patient was not reevaluated for about 2 years until the patient felt pain in the lower left implant area. At this time, a large radiolucent lesion was noticed on the mesial aspect of implant #20 on a periapical radiograph (Figure 1b). The patient was referred back for evaluation. Upon examination, in addition to that large radiolucent lesion, a peri-implant abscess on the buccal of both #19 and #20 implants was noted. Deep probing depth, bleeding, and suppuration were found upon the probing of implant #20. The patient was informed of the findings, and the decision was made to remove implant #20 and biopsy the lesion. The patient was prescribed amoxicillin (Amoximed, Mediate Pharmaceuticals Pvt Ltd, Karachi, Pakistan) and instructed to start 2 days prior to implant removal. The implant was removed with tooth extraction forceps without difficulty. The socket was debrided thoroughly, finding a considerable amount of granulomatous tissues. These tissues were collected and sent to a private institute for pathological examination. The lesion was found to be in close proximity to the mental foramen (Figure 2). The patient reported severe pain on curetting the most apical soft tissue. A fenestration defect of approximately 3 $\times$ 3 mm was present on the buccal of tooth #21. There was a large bony dehiscence defect on #20 buccal as well. The socket was treated with tetracycline solution for 3 minutes and allowed to fill with a blood clot. The patient was advised to take amoxicillin for another 7 days and rinse with Peridex. Verbal and written postoperative instructions were given to the patient.

The pathology report found 2 sulfur granules demonstrating granular and fibrillar basophilic to amphophilic bacterial colonies associated with peripheral purulent exudates (neutrophils; Figure 3a). Granules were composed of intertwined branching bacterial filaments, which were surround-

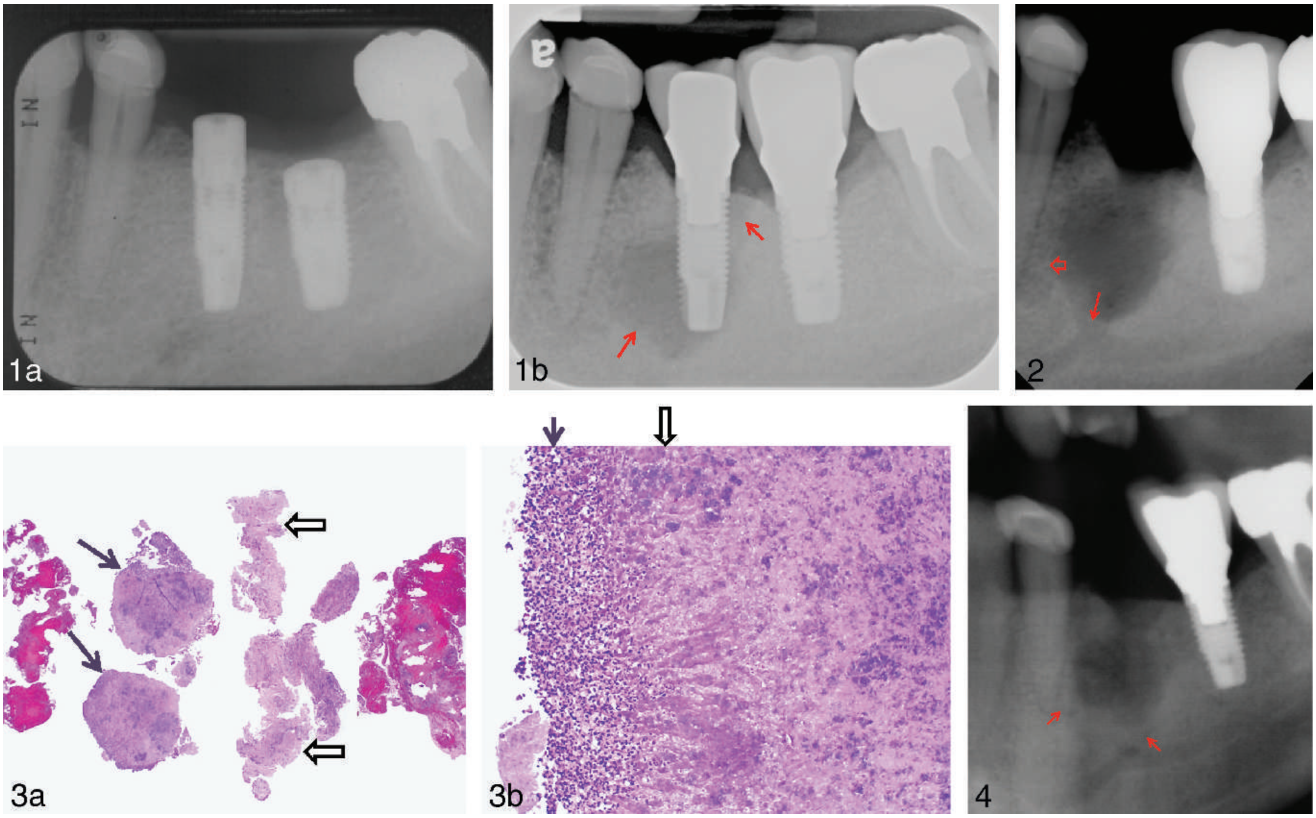

Figures 1–4. Figure 1. Preoperative radiographic finding. (a) Immediately after implant placement. No obvious radiographic lesion was noticed around implants #19 and #20. (b) Two years after implant restoration. A large radiolucent lesion was noticed on the mesial of #20 implant (arrow), which had been extended to the distal of #20 implant (arrow). **Figure 2.** Postoperative radiographic finding at implant removal. The communication with inferior alveolar nerve (arrow), the defect extended into buccal of #21 (open wide arrow). **Figure 3.** Pathological findings. (a) Two sulfur granules (bacterial colonies; arrows) and chronically inflamed tissues (open arrows) were observed under microscope (×10). (b) Bacterial colony consisted of granular basophilic and amphophilic material, which was surrounded by radiating extracellular matrix (open arrow) and surrounding neutrophils (pus; arrow; ×40). **Figure 4.** One-year postoperative radiographic finding. The #20 site is still healing, and there is noticeable bone fill on the #21 distal area and coronal of mental foramen (arrows).

ed by a radiating clubbed extracellular matrix (Figure 3b). The typical ray fungus appearance was obvious. At the periphery of the granule masses, purulence was still present in some areas. Abundant numbers of polymorphonuclear leukocytes were observed. The final diagnosis of actinomycosis was made in consideration of clinical, radiographic, and pathological findings.

The patient was evaluated at 1 week, 1 month, 6 months, and 1 year postoperatively. Healing was normal, and no adverse reaction or recurrence noticed (Figure 4).

Discussion

Periapical actinomycosis is believed to be rare. Only 94 cases of periapical actinomycoses have been reported, which accounts for an incidence of 1.8%–4.4%.[11–13] In some cases, the infection may occur in combination with a radicular cyst.[14] Moreover, the prognosis of periapical actinomycosis is believed to be better than other cervicofacial infection, with fewer complications or recurrence.[12] There are 2 possible explanations for this case. First, although the reason for missing tooth #20 is unknown, we can speculate that it was due to decay because the patient's periodontal condition was very stable and periodontal bone loss was minimal. The tooth might have been infected with *Actinomyces* prior to its extraction. Because *Actinomyces* are slow-growing bacteria, it may be latent for many years before it became symptomatic. In this case, tooth #20 might have been extracted before any symptom developed and the infection became quiescent

due to removal of infection source and host response. But implant surgery at this site reactivated the infection and exacerbated its development. Another possibility is that the implant was contaminated by *Actinomyces* during placement. The implant brought the bacteria into the osteotomy site, and disease developed shortly after implant surgery. In either situation, this is the first case report regarding actinomycosis-caused dental implant failure.

The patient's impaired immune response may have contributed to the disease. The patient had severe pneumonia that required surgical intervention, has hepatitis C being treated with a combination of medications, and had herpes genitalis. All of these indicate that this patient might be immunodeficient and is very susceptible to various infections.

Classical signs and symptoms of infection, such as redness, swelling, fever, and pain, may not be noticeable with actinomycosis. *Actinomyces* are sensitive to a number of antibiotics, and penicillin is the drug of choice.[1] Most clinicians recommend extended antibiotic treatment, usually from 2 to 12 months in duration. In certain periapical lesions, short-term antibiotic administration may be adequate.[1,2,3,7] Under most circumstances, surgical excision or debridement is desired also, especially when a neoplasm is suspected. Long-term follow-up is needed as the recurrence of infection has been reported.

Acknowledgment

We, the authors, graciously thank Dr Lee J. Slater, Scripps Oral Pathology Service, for providing histological photographs. The authors do not have any conflict of interest for this case report.

References

1. Smego RA Jr, Foglia G. Actinomycosis. *Clin Infect Dis*. 1998;26:1255–1261.
2. Nair PNR. On the causes of persistent apical periodontitis: a review. *Int Endod J*. 2006;39:249–281.
3. Jeansonne BG. Periapical actinomycosis: a review. *Quintessence Int*. 2005;36:149–153.
4. Pulverer G, Schutt-Gerowitt H. Human cervicofacial actinomycoses: microbiologic data for 1997 cases. *Clin Infect Dis*. 2003;37:490–497.
5. Lancella A, Abbate G, Foscolo AM, Dosdegani R. Two unusual presentations of cervicofacial actinomycosis and review of the literature. *Acta Otorhinolaryngol Ital*. 2008;28:89–93.
6. Palonta F, Preti G, Vione N, Cavalot AL. Actinomycosis of the masseter muscle: report of a case and review of the literature. *J Craniofac Surg*. 2003;14:915–918.
7. Oostman O, Smego RA. Cervicofacial actinomycosis: diagnosis and management. *Curr Infect Dis Rep*. 2005;7:170–174.
8. Apel S, Apel C, Morea C, Tortamano A, Dominguez GC, Conrads G. Microflora associated with successful and failed orthodontic mini-implants. *Clin Oral Implants Res*. 2009;20:1186–1190.
9. Sarkonen N, Könönen E, Eerola E, Könönen M, Jousimies-Somer H, Laine P. Characterization of *Actinomyces* species isolated from failed dental implant fixtures. *Anaerobe*. 2005;11:231–237.
10. Ricucci D, Siqueira JF Jr. Apical actinomycosis as a continuum of intraradicular and extraradicular infection: case report and critical review on its involvement with treatment failure. *J Endod*. 2008;34:1124–1129.
11. Sakellariou PL. Periapical actinomycosis: report of a case and review of the literature. *Endod Dent Traumatol*. 1996;12:151–154.
12. Hirshberg A, Tsesis I, Metzger Z, Kaplan I. Periapical actinomycosis: a clinicopathologic study. *Oral Surg Oral Med Oral Pathol Oral Radiol Endod*. 2003;95:614–620.
13. Nair PNR, Schroeder HE. Periapical actinomycosis. *J Endod*. 1984;10:567–570.
14. Tseng SK, Tsai YL, Li UM, Jeng JH. Radicular cyst with actinomycotic infection in an upper anterior tooth. *J Formos Med Assoc*. 2009;108:808–813.
