# Supplementary material for: A novel histopathological classification of implant periapical lesion: A systematic review and treatment decision tree
Source: PLoS One. 2022 Dec 22;17(12):e0277387. doi: 10.1371/journal.pone.0277387 (PMC9778521; doi:10.1371/journal.pone.0277387)
Supplement: S1 File — (ZIP) [file pone.0277387.s001.zip › support files/Included study/Thompson-Sloan 2012.pdf]

Management of Retrograde Peri-Implantitis by Apical Resection and Guided Bone Regeneration in Adjacent Maxillary Implants

Tamika N. Thompson-Sloan,* Shilpa Kolhatkar,* and Monish Bhola*

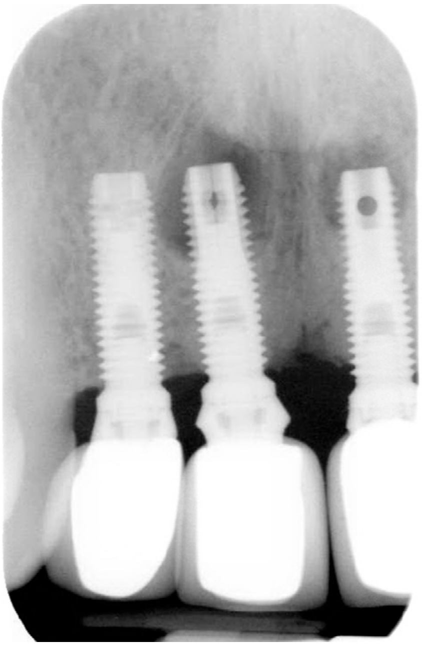

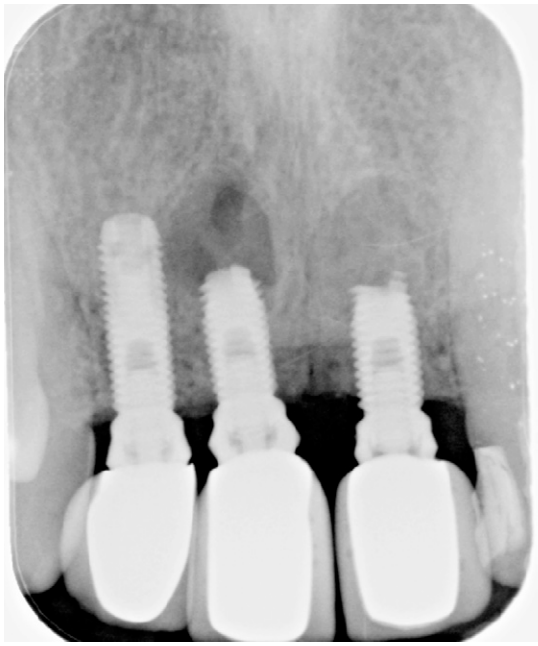

Introduction: Retrograde peri-implantitis (RPI) is defined as a clinically symptomatic periapical lesion that develops shortly after implant insertion while the coronal portion of the implant sustains a normal bone-to-implant interface. The etiology of RPI is unclear and may be attributed to several causes. Regardless of etiology, the management of RPI has been attempted by either resective or regenerative techniques.

Case Presentation: A 61-year-old male was screened and evaluated for three maxillary anterior implants placed 10 years previously. Evaluation included a thorough periodontal and dental exam, radiographs, and cone-beam computed tomography. Probing depths around all implants ranged from 2 to 4 mm with no bleeding on probing/mobility. The apices of implants #8 and #9 exhibited radiolucencies, and a draining fistula was associated with implant #8. Treatment consisted of sectioning and removal of the affected portion of the implants and collection of a specimen for histopathologic examination. Demineralized bone matrix putty was placed in the residual bony defects and covered with a collagen barrier. At 1 year, radiographs revealed both lesions around implants #8 and #9 appeared to be resolving. The lesion associated with implant #9 had a more remarkable radiographic change, because it was smaller than the lesion around implant #8. Both implants continued to function with no recurrence of fistula formation. Histopathologic analysis was consistent with the diagnosis of a periapical granuloma.

Conclusion: Resection of the apical portion of implants is a viable treatment modality in the management of RPI. *Clin Adv Periodontics* 2012;2:250-255.

Key Words: Bone regeneration; dental implants; infection; peri-implantitis.

* *Department of Periodontology and Dental Hygiene, University of Detroit Mercy, Detroit, MI.*

Submitted November 29, 2011; accepted for publication January 24, 2012

doi: 10.1902/cap.2012.110106

Background

An entity separate from peri-implantitis was reported in the literature first by McAllister et al.[1] as retrograde peri-implantitis (RPI). It is defined as a "clinically symptomatic periapical lesion that develops within the first few months after implant insertion while the coronal portion of the implant sustains a normal bone to implant interface."[1] The

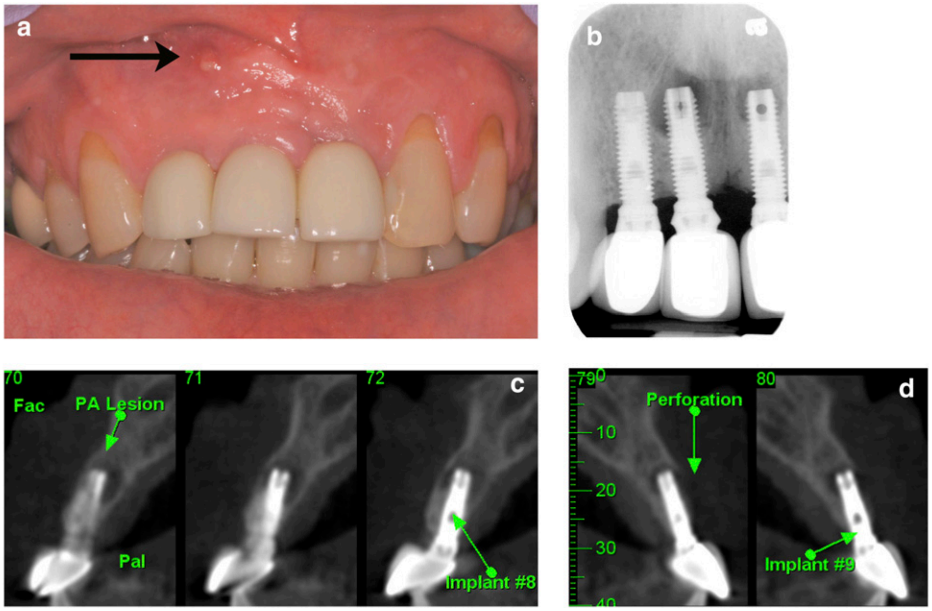

FIGURE 1 Initial clinical presentation of three implants placed in 2001. The patient gave a history of multiple episodes of fistula formation, which were managed using only oral antibiotics. **1a** A clinical exam revealed probing depths around all implants ranging from 2 to 4 mm with no bleeding on probing and no mobility and the presence of a fistula apical to implant #8 (black arrow). **1b** A periapical radiograph revealed the presence of radiolucencies associated with the apices of implants #8 and #9 and marginal discrepancy of the implant-supported crowns. **1c** The buccofacio (Fac)-palatal (Pal) extent of osseous destruction was assessed using CBCT. Implant #8 was in close proximity to the nasopalatine canal with perforation of the palatal cortical plate. The periapical lesion (green arrow) completely enveloped the apical portion of the implant and extended approximately half the length of the implant on the palatal aspect. **1d** Implant #9 showed less osseous destruction in the form of a smaller palatal and apical lesion with a perforation on the labial aspect (green arrows).

incidence of RPI based on a handful of studies ranges from <1%[2] to 9.9%.[3,4] Additional reports have described the presence of RPI in one or two cases.[5,6]

The etiology of RPI[6,7] is unclear and may be attributed to several causes, including implant surface contamination, overheating during preparation or placement, residual bacteria in the implant site, presence of adjacent endodontic lesions, and drainage of inflammation from marrow spaces. Additional causes may be violation of minimal distance from adjacent teeth, residual root particles or foreign bodies, surgical drilling beyond the length of the implant, fenestration of vestibular bone, bone compression, poor bone quality, premature loading, development of osteomyelitis, technique of the implant system used, and bone loss as a result of a flap procedure.[7]

A search of the literature reveals a paucity of information about the etiology of RPI. Regardless of etiology, the management of RPI has been attempted by either regenerative[1,8-13] or resective[10,14,15] techniques. Isolated reports have described the use of debridement[16] or antibiotics alone[17] for its management. Reports citing the success of both approaches are available.[1,15] In a retrospective study by Quirynen et al.,[3] 10 (seven maxillary and three mandibular) of 539 implants were treated for RPI using regenerative techniques. One of the mandibular implants was lost after an 18-month follow-up. In another retrospective study, Balshi et al.[4] treated 39 of 395 implants for RPI using resective techniques and followed these cases for 4 to 15 years. Of the 39 implants, 38 were successfully treated with this technique.

In this case report, the management of RPI involving two of three implants placed in the anterior maxilla using an apical resective approach with guided bone regeneration (GBR) is described.

Clinical Presentation

A 61-year-old male was referred to the graduate periodontics clinic of the University of Detroit Mercy, School of

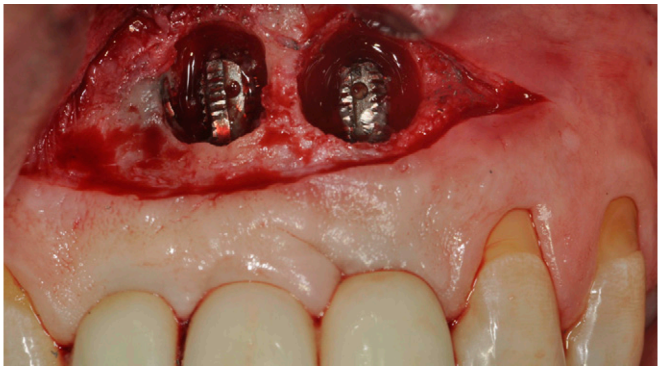

FIGURE 2 Access to the implant apices was enlarged, and the apical one third of both implant fixtures were subsequently resected.

Dentistry (UDMSOD), Detroit, Michigan, on October 23, 2009, with a chief complaint of, "I have three implants that I am concerned about and would like an evaluation." The patient had been treated multiple times with antibiotics with no permanent resolution of the fistula associated with implant #8.

Evaluation at UDMSOD included a thorough periodontal (Fig. 1a) and dental exam, radiographs (Fig. 1b), and cone-beam computed tomography (CBCT) (Figs. 1c and 1d). A thorough occlusal analysis was conducted, and no occlusal discrepancies were detected.

After careful discussion of all treatment options, the decision to resect the apical portion of the implants and perform regenerative therapy was made because of the extensive nature of the lesion (erosion of buccal and palatal cortical plates as detected by CBCT) and the fact that we could not access the apical and palatal areas for debridement.

Case Management

On the day of the surgery, written informed consent was obtained. A full-thickness flap was reflected from both the buccal and palatal aspects to ensure proper access to the underlying implant-associated lesions (Video 1). The

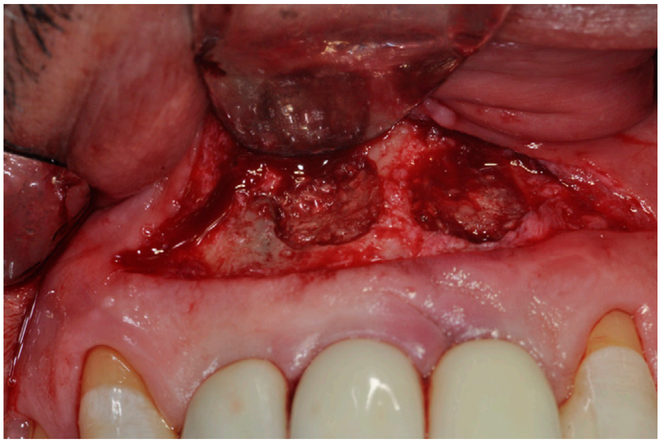

FIGURE 3 Placement of initial increment of demineralized bone matrix putty.

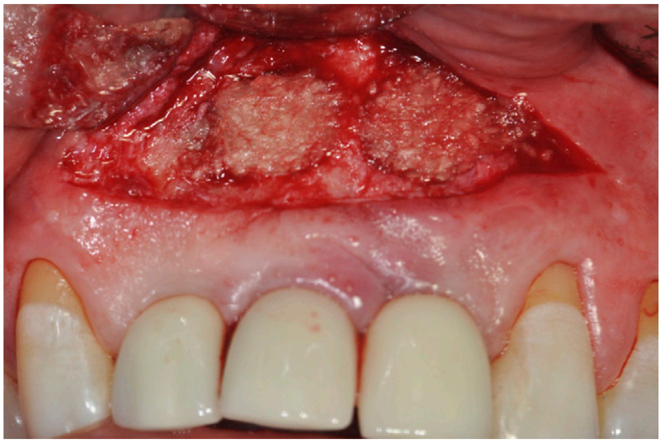

FIGURE 4 Completion of bone graft augmentation.

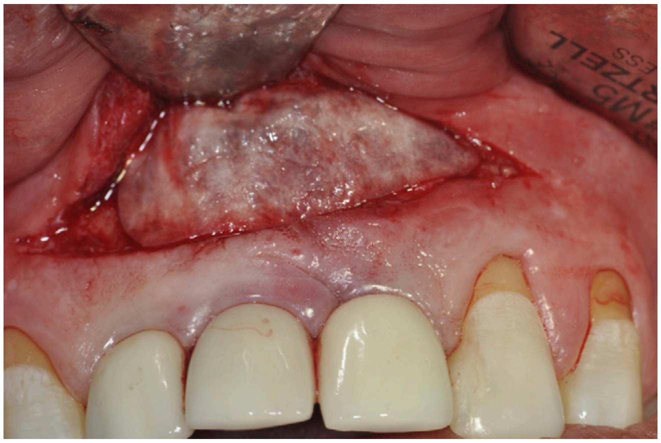

FIGURE 5 Placement of resorbable collagen barrier that was trimmed to cover both implant apices.

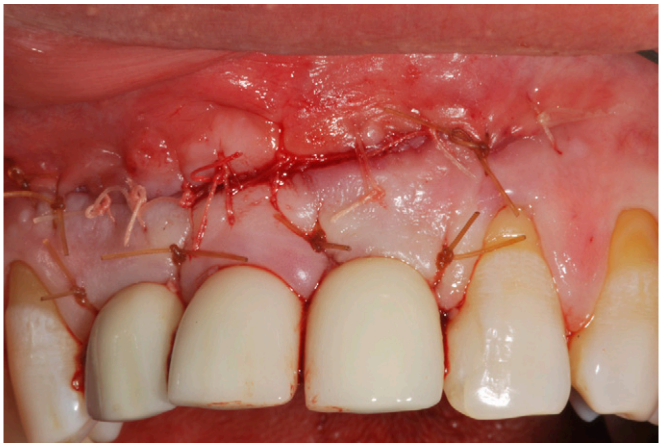

FIGURE 6 Surgical wound closure using interrupted sutures.

preexisting fenestrations were enlarged with a round carbide bur[†] (Fig. 2), biopsy samples were obtained, and the affected one third of the implants were amputated with burs.[‡] Care was taken to reduce generation of heat by using copious irrigation and very light pressure. A particular concern was the proximity of implants #7 and #8, and, therefore special care was taken to avoid gouging the bony walls separating them. The residual bony cavities were treated by bathing in 300 mg/2 mL clindamycin solution[§] for 3 minutes, using a collagen barrier[||] on the palatal aspect of implant #8, demineralized bone matrix putty[¶] (Figs. 3 and 4), and another collagen barrier[#] on the facial aspect (Fig. 5). Surgical wound closure was completed (Fig. 6), and a postoperative radiograph was taken (Fig. 7). The patient was prescribed postoperative medications (azithromycin,[**] 250 mg 2 tablets stat, 1 tablet daily until gone; chlorhexidine gluconate,[††] 0.12% twice daily; Ibuprofen,[‡‡] 600 mg, every 6 to 8 hours as needed for pain) and given postoperative instructions to refrain lifting the upper lip, brushing the surgical area, or incising food using the anterior teeth.

Clinical Outcomes

The patient was recalled for postoperative visits at 1, 2, and 4 weeks. The histopathologic analysis was consistent with the diagnosis of a periapical granuloma (Fig. 8).

At the 6-month postoperative visit, soft-tissue healing was unremarkable, and the periapical radiograph displayed increasing radiodensity around both implants. At 1 year, radiographs revealed both lesions around implants #8 and #9 appeared to be resolving. The lesion associated with implant #9 had a more remarkable radiographic change, because it was smaller than the lesion around implant #8. Both implants continued to function with no recurrence of fistula formation. The crestal bone level remained stable, and no changes in probing depth or mobility were noted (Fig. 9).

Discussion

Management of RPI has been attempted using techniques such as debridement alone[16]or in combination with osseous grafts and/or barriers. Use of antibiotics alone or with

† Benco, Pittston, PA.
‡ SS White, Lakewood, NJ.
§ Hospira, Lake Forest, IL.
|| BioMend Extend, Zimmer Dental, Carlsbad, CA.
¶ Accell, Keystone Dental, Burlington, MA.
# DynaMatrix, Keystone Dental.
** Zithromax, Pfizer, New York, NY.
†† Peridex, 3M ESPE, St. Paul, MN.
‡‡ Janssen Pharmaceuticals, Titusville, NJ.

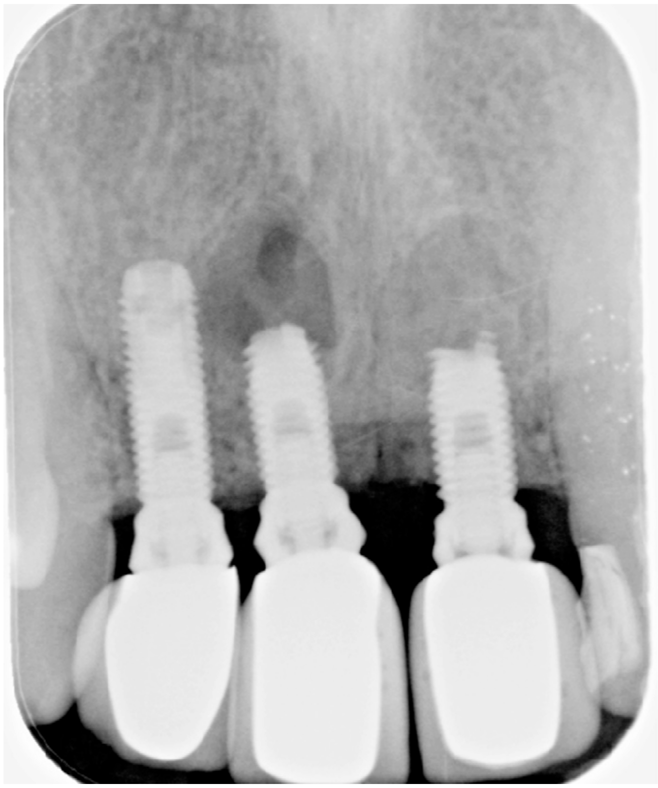

FIGURE 7 Postoperative radiograph taken immediately after completion of resection and placement of bone graft.

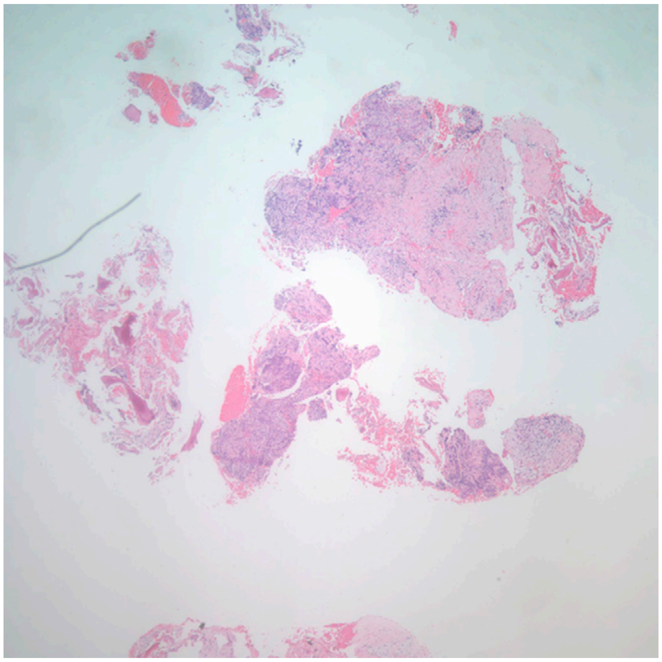

FIGURE 8 Histopathologic analysis using hematoxylin and eosin stain revealed predominately fibrovascular connective tissue and granulation tissue. A dense infiltrate of both acute and chronic inflammatory cells was seen embedded in the soft tissue. Vital lamellar bone and extravasated erythrocytes were located at the periphery of the histologic specimen, which was consistent with the diagnosis of a periapical granuloma. Original magnification ×4.

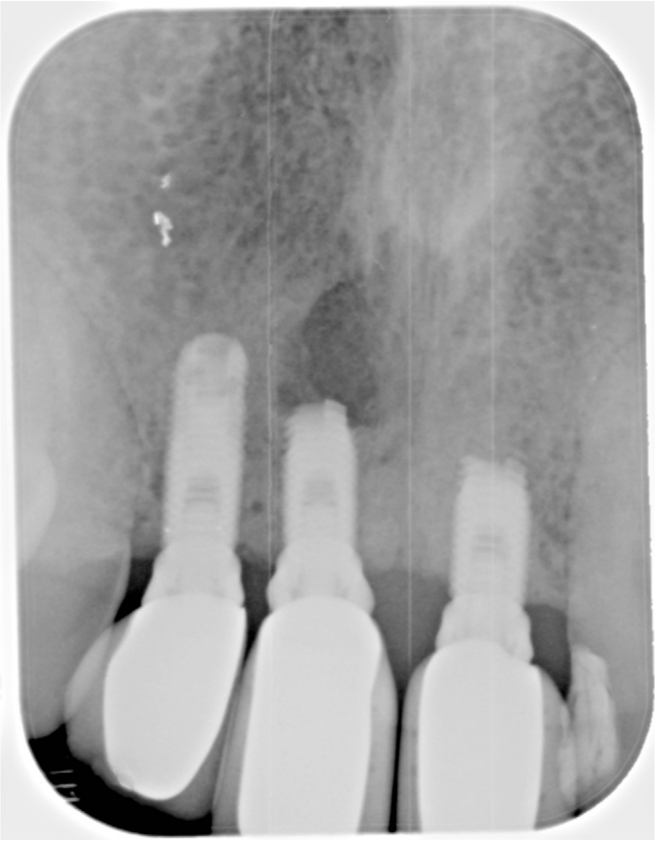

FIGURE 9 One-year postoperative periapical demonstrating increased radiodensity and maturation of bone.

additional regenerative materials has also been documented.[8-10,14] Reports in the literature on the management of these lesions by antibiotics alone have had poor outcomes.[9,14]

The other surgical option, which also focuses on retention of the implant, is resection.[4,10,14,15] Resective techniques follow a similar protocol, with the primary difference being the resection of the infected apical portion of the implant. Balshi et al.[4] treated 39 cases with resective therapy and used bovine bone to graft the defects. A collagen membrane was placed over the larger defects. The follow-up time averaged 4.5 years, with 15 years being the longest follow-up. All but one of the 39 implants treated with this technique was successfully retained. The protocol of Dahlin et al.[15] after resection consisted of irrigation with saline and postoperative systemic antibiotics. The 3-year follow-up of the two cases, with two implants total, revealed complete bone fill into the resected area. The bone levels around the implants were stable, and the patients remained asymptomatic.

In our case report, after assimilating all diagnostic information, the following treatment options were discussed with the patient: 1) implant resection and GBR; 2) implant surface decontamination and GBR; and 3) removal of implants, GBR, and placement of new implants in 6 months. Our clinical approach was similar to the above reports.[4,15] A full-thickness buccal and palatal flap was elevated because of the extensive nature of the lesion. Similar to the protocol of Balshi et al.,[4] the size of the buccal fenestration

had to be increased to provide access to the apical lesion. Although only two thirds of the implants threads were covered with bone, both implants were immobile. The decision to surgically access the implant apices and use resective techniques was based primarily on the fact that complete debridement of the infected implant portions would be impossible given the extent of the lesions.

A combination of resective treatment, antibiotic decontamination of the surgical sites, and regenerative therapy is a viable treatment modality for the management of RPI. This case was followed for 1 year, and both lesions in the areas of implants #8 and #9 appeared to be resolving with no clinical symptoms. The lesion of implant #9 had a more remarkable radiographic change compared to the size and extent of the lesion on implant #8. An additional regenerative procedure may be indicated at this site in the future. Both implants continued to function with no recurrence of fistula formation. ■

Summary

**Why is this case new information?**	■ We highlight the fact that a combination of resective and regenerative therapy is a viable treatment modality for RPI cases that range in small to large areas of bone destruction. This combination of treatment can help salvage implants afflicted with RPI in the anterior areas of the mouth without compromising the coronal peri-implant esthetics.
**What are the keys to successful management of this case?**	■ We achieved a favorable outcome in this case report because we were able to remove the affected apical portion of the implant and access and remove the surrounding pathology. The residual defects in bone were augmented with a bone graft.
**What are the primary limitations to success in this case?**	■ For implant #8, the bucco-palatal extent of osseous destruction was significant, with perforation of the palatal cortical plate. The lesion completely enveloped the apical portion of the implant and extended approximately half the length of the implant on the palatal aspect. Implant #9 showed less osseous destruction in the form of a smaller palatal and apical lesion.<br>■ At the 12-month postoperative visit, radiographically, the apex of implant #8 appeared less radiodense compared to implant #9. This was attributed to the size and extent of the lesion around implant #8 along with the bicortical perforation.

Acknowledgments

The authors acknowledge Mr. Eric Jacobs, Media Specialist, School of Dentistry, University of Detroit Mercy, Detroit, Michigan, for his assistance with videotaping and photography in this case report. Drs. Thompson-Sloan and Kolhatkar report no conflict of interest related to this case report. Dr. Bhola is a consultant for Keystone Dental, Burlington, Massachusetts, and Zimmer Dental, Carlsbad, California.

CORRESPONDENCE:
Dr. Tamika N. Thompson-Sloan, 2700 Martin Luther King Jr. Blvd., Detroit, MI 48208. E-mail: thompson_tamika@yahoo.com.

References

1. McAllister BS, Masters D, Meffert RM. Treatment of implants demonstrating periapical radiolucencies. *Pract Periodontics Aesthet Dent* 1992;4:37-41.
2. Reiser GM, Nevins M. The implant periapical lesion: Etiology, prevention, and treatment. *Compend Contin Educ Dent* 1995;16: 768, 770, 772 passim.
3. Quirynen M, Vogels R, Alsaadi G, Naert I, Jacobs R, van Steenberghe D. Predisposing conditions for retrograde peri-implantitis, and treatment suggestions. *Clin Oral Implants Res* 2005;16:599-608.
4. Balshi SF, Wolfinger GJ, Balshi TJ. A retrospective evaluation of a treatment protocol for dental implant periapical lesions: Long-term results of 39 implant apicoectomies. *Int J Oral Maxillofac Implants* 2007;22:267-272.
5. Piattelli A, Scarano A, Piattelli M, Podda G. Implant periapical lesions: Clinical, histologic, and histochemical aspects. A case report. *Int J Periodontics Restorative Dent* 1998;18:181-187.
6. Scarano A, Di Domizio P, Petrone G, Iezzi G, Piattelli A. Implant periapical lesion: A clinical and histologic case report. *J Oral Implantol* 2000;26:109-113.
7. Chan HL, Wang HL, Bashutski JD, Edwards PC, Fu JH, Oh TJ. Retrograde peri-implantitis: A case report introducing an approach to its management. *J Periodontol* 2011;82:1080-1088.
8. Bretz WA, Matuck AN, de Oliveira G, Moretti AJ, Bretz WA. Treatment of retrograde peri-implantitis: Clinical report. *Implant Dent* 1997;6:287-290.
9. Brisman DL, Brisman AS, Moses MS. Implant failures associated with asymptomatic endodontically treated teeth. *J Am Dent Assoc* 2001; 132:191-195.
10. Jalbout ZN, Tarnow DP. The implant periapical lesion: Four case reports and review of the literature. *Pract Proced Aesthet Dent* 2001; 13:107-112, quiz 114.
11. Ataullah K, Chee LF, Peng LL, Lung HH. Management of retrograde peri-implantitis: A clinical case report. *J Oral Implantol* 2006;32:308-312.
12. Tözüm TF, Sençimen M, Ortakoğlu K, Ozdemir A, Aydin OC, Keleş M. Diagnosis and treatment of a large periapical implant lesion associated with adjacent natural tooth: A case report. *Oral Surg Oral Med Oral Pathol Oral Radiol Endod* 2006;101:e132-e138.
13. Peñarrocha-Diago M, Boronat-Lopez A, García-Mira B. Inflammatory implant periapical lesion: Etiology, diagnosis, and treatment — Presentation of 7 cases. *J Oral Maxillofac Surg* 2009;67:168-173.
14. Shaffer MD, Juruaz DA, Haggerty PC. The effect of periradicular endodontic pathosis on the apical region of adjacent implants. *Oral Surg Oral Med Oral Pathol Oral Radiol Endod* 1998;86:578-581.
15. Dahlin C, Nikfarid H, Alsén B, Kashani H. Apical peri-implantitis: Possible predisposing factors, case reports, and surgical treatment suggestions. *Clin Implant Dent Relat Res* 2009;11:222-227.
16. Ayangco L, Sheridan PJ. Development and treatment of retrograde peri-implantitis involving a site with a history of failed endodontic and apicoectomy procedures: A series of reports. *Int J Oral Maxillofac Implants* 2001;16:412-417.
17. Waasdrop J, Reynolds M. Nonsurgical treatment of retrograde peri-implantitis: A case report. *Int J Oral Maxillofac Implants* 2010;25:831-833.

○ indicates key references.
