# Supplementary material for: A novel histopathological classification of implant periapical lesion: A systematic review and treatment decision tree
Source: PLoS One. 2022 Dec 22;17(12):e0277387. doi: 10.1371/journal.pone.0277387 (PMC9778521; doi:10.1371/journal.pone.0277387)
Supplement: S1 File — (ZIP) [file pone.0277387.s001.zip › support files/Included study/Troiano 2020.pdf]

dentistry journal

MDPI

Case Report

Salvage of Dental Implant Located in Mandibular Odontogenic Cyst. A Conservative Surgical Treatment Proposal

Antonio Troiano [1], Giorgio Lo Giudice [1,*], Roberto De Luca [1], Fabrizio Lo Giudice [2], Salvatore D'Amato [3], Gianpaolo Tartaro [3,†] and Giuseppe Colella [3,†]

1 Maxillofacial Surgery Unit, Department of Neurosciences, Reproductive and Odontostomatological Sciences, University of Naples "Federico II", 80138 Naples, Italy; antoniotroiano85@gmail.com (A.T.); robertodeluca89@yahoo.it (R.D.L.)
2 Department of Biomedical and Dental Sciences and Morphofunctional Imaging, Messina University, 98122 Messina, Italy; fabrizio.logiudice@hotmail.it
3 Multidisciplinary Department of Medical-Surgical and Dental Specialities, Oral and Maxillofacial Surgery Unit, AOU University of Campania "Luigi Vanvitelli", 80138 Naples, Italy; salvatore.damato@unicampania.it (S.D.); gianpaolo.tartaro@unicampania.it (G.T.); giuseppe.colella@unicampania.it (G.C.)
* Correspondence: giorgio.logiudice@gmail.com; Tel.: +39-3472507395
† These senior authors contributed equally to this study.

Received: 24 April 2020; Accepted: 8 May 2020; Published: 11 May 2020

check for updates

Abstract: The aim of this case report was to evaluate the use of Partsch I cystotomy in order to preserve a dental implant located in an odontogenic cyst extended from 3.2 to 4.4. A 50 year-old woman showed a circular, well-defined unilocular radiolucent area, Ø2.5 cm, in the right mandibular region with an oral implant intruding inside it. The overdenture in the mandibular right site showed no clinical mobility. The authors decided to perform a surgical treatment aimed to preserve the implant. The patient underwent Partsch I surgery followed by iodoform gauze insertion replaced weekly for one month, revision of the previous orthograde endodontic treatments, and an acrylic resin obturator prosthesis application for the following two months. The twelve month follow-up showed no clinical mobility of the right lateral mandibular implant prostheses. Radiographical analysis revealed cystic lesion healing and perimplant bone regeneration. This report highlights the opportunity to apply cystotomy when the cyst involves a dental implant and undermines its stability. This possibility is offered by the peculiar clinical scenario where the implant was stabilized by the presence of a previous prosthetic fixation. Our study led to the application of an operative protocol that allowed for the preservation of the implant.

Keywords: cyst; dental implant; impacted implant; marsupialization; Partsch

1. Introduction

Odontogenic cysts are the most frequent jawbone benign neoformations of inflammatory origin. These endo-osseous cavitations have prevalence between the third and fifth decade and are more frequent in females and seldom surpass 1 cm in diameter [1,2]. The WHO classification based on histology categorizes jawbone cysts into several categories:

- Odontogenic cysts of inflammatory origin (radicular cyst, inflammatory collateral cysts).
- Odontogenic and non-odontogenic developmental cysts (dentigerous cyst, odontogenic keratocyst, lateral periodontal cyst and botryoid odontogenic cyst, gingival cysts, glandular odontogenic cyst, calcifying odontogenic cyst, orthokeratinized odontogenic cyst, nasopalatine duct cyst) [3].

Several treatment choices are available for cysts in maxillary bones: endodontic treatment, surgical-endodontic management performing apicectomy and retrograde root canal filling, extraction, enucleation (Partsch II), marsupialization (Partsch I), or a combination of the two (Partsch I followed by Partsch II). The surgical golden standard is Partsch II, but a conservative treatment can be planned to prevent surgical risks. When the Partsch I approach is performed, the surgeon must guarantee the oral breach is open until the cyst dimension shrinks and prevent any food or debris from going inside by the means of an iodine gauze insertion into the cavity and/or obturators. Moreover, the patient must be instructed on oral hygiene to avoid any complication. A two-stage approach is also a possible management strategy: first Partsch I, then Partsch II as soon as the cyst dimension allows it [4–6].

The relation between dental implants and cysts has been analyzed in the literature by researchers. The cases focused on radiolucent lesions related to implant periapical lesions or derived from epithelium inclusion caused by sinus surgery and implant placement near the nasopalatine duct [7–10]. Other studies have considered surgical timing, outcome, and implant insertion in infected sites [11–18]. The common treatment plan in implant failure usually follows three steps: explantation of the implant, bone regeneration, and reimplantation. Dental implant explantation is due to mechanical problems such as a lack of primary stability, overload, biological reasons like peri-implantitis and bone resorption, or when adjacent to malignant neoplasms. Nevertheless, the authors have never seen described in the literature any guidelines of implant explantation when there are adjacent or impacting cystic lesions [19]. The aim of this case report was to evaluate the use of the Partsch I surgical technique in order to preserve a dental implant impacted in an odontogenic cyst extended from 3.2 to 4.4. This choice was made considering the stability of the fixture and represents a rare occasion of implant saving and achieved osseointegration.

2. Case Report

The study was approved by the internal ethical committee of the University (AOU-Second University-Naples (SUN), prot. 3733, 16 May 2015). The patient signed the informed consent for the procedures.

A 50 year-old woman was referred to the Maxillo-Facial Surgery Department of the University of Campania "Luigi Vanvitelli" with painless facial swelling in the mental region. No comorbidities were referred. Intraoral inspection revealed a bony expansion in the right vestibular mandibular region. Presence of sinus with purulent drainage in vestibular mental region was observed; submental lymph nodes were palpable. Bilateral mandibular implant-supported prostheses were present and no clinical mobility of overdenture was shown.

Orthopantomography was performed, followed by Cone Beam Computed Tomography scan that showed a large, circular, well-defined unilocular radiolucent area, starting from the left mandibular lateral incisor and extending to the right mandibular first premolar, Ø 2.5 cm. The cystic lesion enclosed in region 4.4 an implant fixture; no signs of root resorption were detected in the adjacent teeth.

In the mandibular region, the radiological analysis showed:

- Implant-supported prostheses from 3.4 to 3.6 and from 4.4 to 4.6.
- Prosthetic rehabilitation on dental support from 3.3 to 4.3. Metallic endocanalar posts in 3.3, 4.2, and 4.3.
- Endodontic treatment on 3.1, 3.2, 3.3, 4.1, 4.2, and 4.3.

The endodontic treatment of 4.1, 4.2, and 4.3 did not reach the radiological tooth apex (Figure 1).

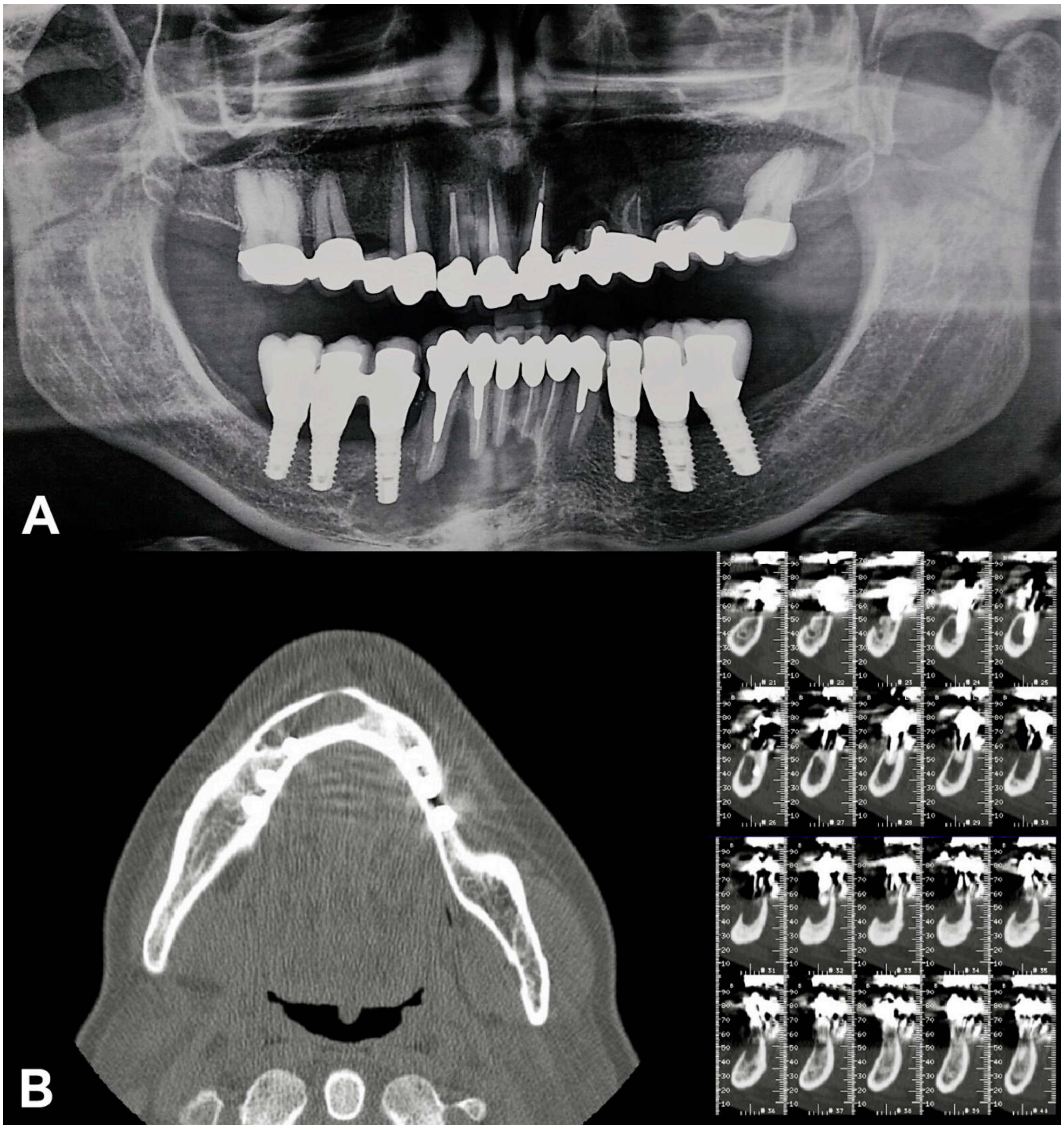

Figure 1. Pre-surgical radiographical examinations. (**A**) Orthopantomography; (**B**) Cone Beam Computed Tomography.

Based on the clinical and radiological signs, the preliminary diagnosis was odontogenic cysts of inflammatory origin. The patient signed the informed consent for the procedures and the publication of this paper. Partsch I surgery was performed, then an orthograde endodontic retreatment of 3.1, 3.2, 3.3, 4.1, 4.2, and 4.3 shortly after surgery, during the healing period (Figure 2A). The marsupialization of the cyst was achieved using piezosurgery and a tissue sample was taken for histopathological analysis (Figure 3).

An iodine gauze was inserted to the decompression socket of the right lateral incisor to relieve the endocystic pressure (Figure 2B). An antibiotic prophylaxis treatment was performed with twice a day doses of Amoxicillin 875 mg and Clavulanic acid 125 mg in pre surgical time (three days) and the post-operative phase (seven days) for one week. The iodine gauze was replaced weekly for one month during postoperative follow-ups (Figure 2C). An acrylic resin obturator prosthesis was then inserted to maintain the surgical opening and to improve hygiene for two months (Figure 2D–F).

Histopathologic examination on the sample confirmed our initial diagnosis as an odontogenic inflammatory cyst (Figure 3).

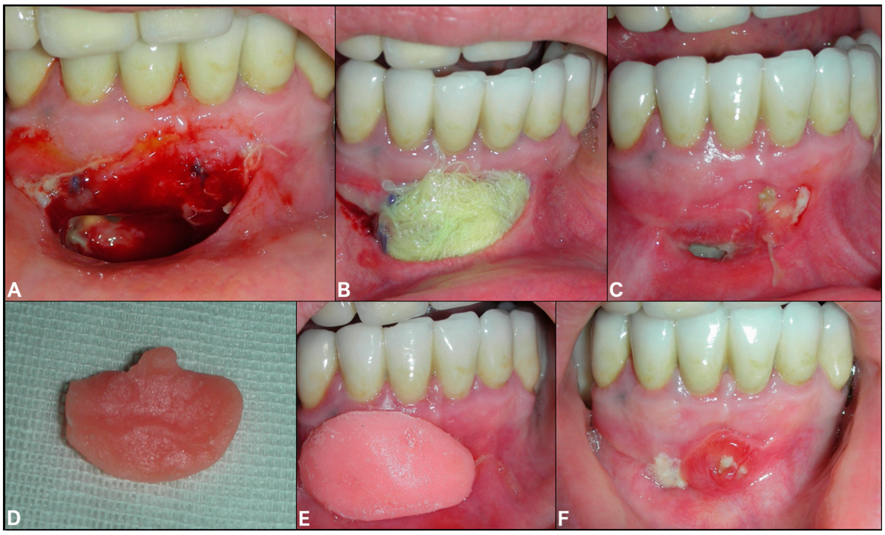

Figure 2. (**A**) Intraoral intraoperative view; (**B**) Intraoperative iodine gauze insertion; (**C**) 1 month follow-up; (**D**) Gingival view of the acrylic resin obturator; (**E**) Intraoral view of the obturator placement; (**F**) Three month follow-up.

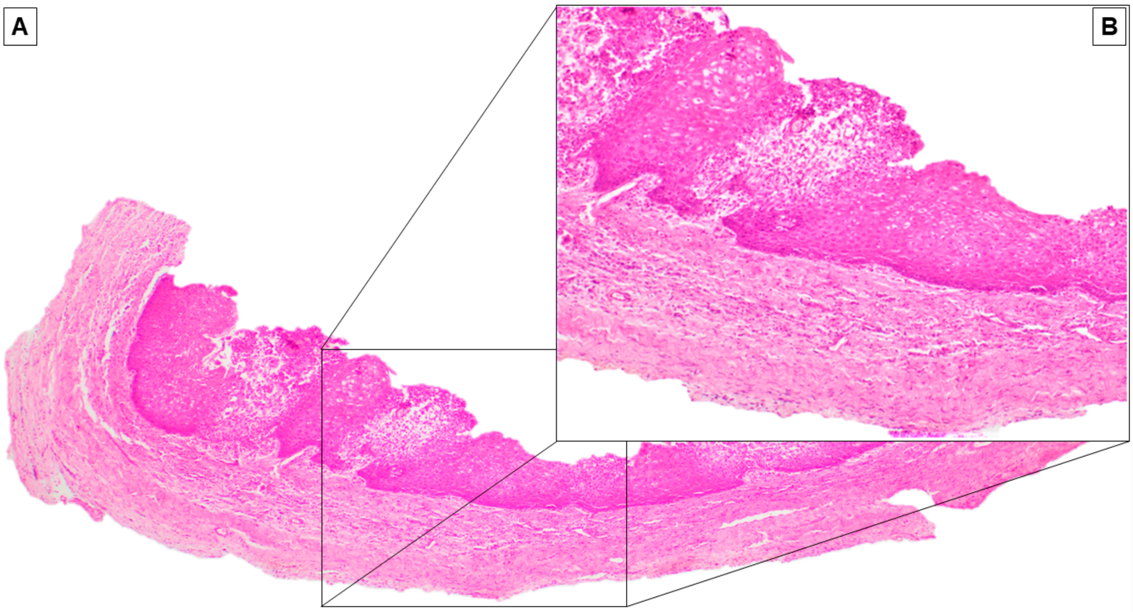

Figure 3. (**A**) On histologic sections, the cystic wall was covered by a stratified non-keratinizing squamous epithelium (Hematoxylin and Eosin, original magnification 40×). (**B**) At higher magnification, the epithelium appeared hyperplastic, with acanthosis, vacuolization of the cheratinocytes, and focal granulocyte exocytosis (Hematoxylin and Eosin, original magnification 100×).

At the five month follow-up, intraoral examination in the mandibular region showed no signs of inflammation and fistula in vestibular mucosa or mobility of the right dental implant prostheses. Radiographical analysis showed an increase in the bone density of the cystic area, new bone formation around the implant, and an evident decrease in periapical teeth radiolucency (Figure 4).

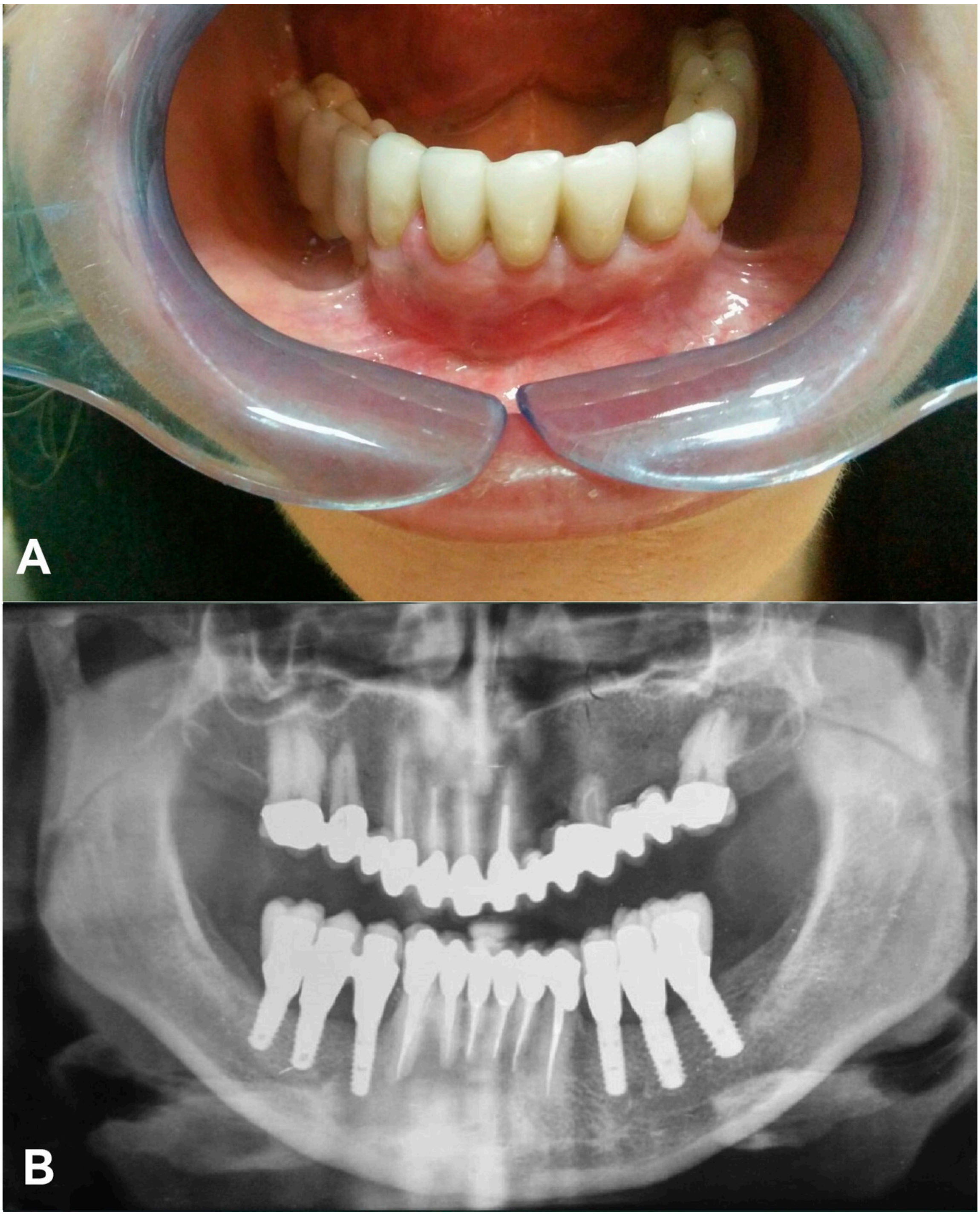

Figure 4. Five month follow-up. (**A**) Intraoral view; (**B**) Orthopantomography.

The twelve month follow-up confirmed the clinical and radiological stability of the treatment over time, bone regeneration in the cystic area, and a renewed bone relation with a 4.4 fixture (Figure 5).

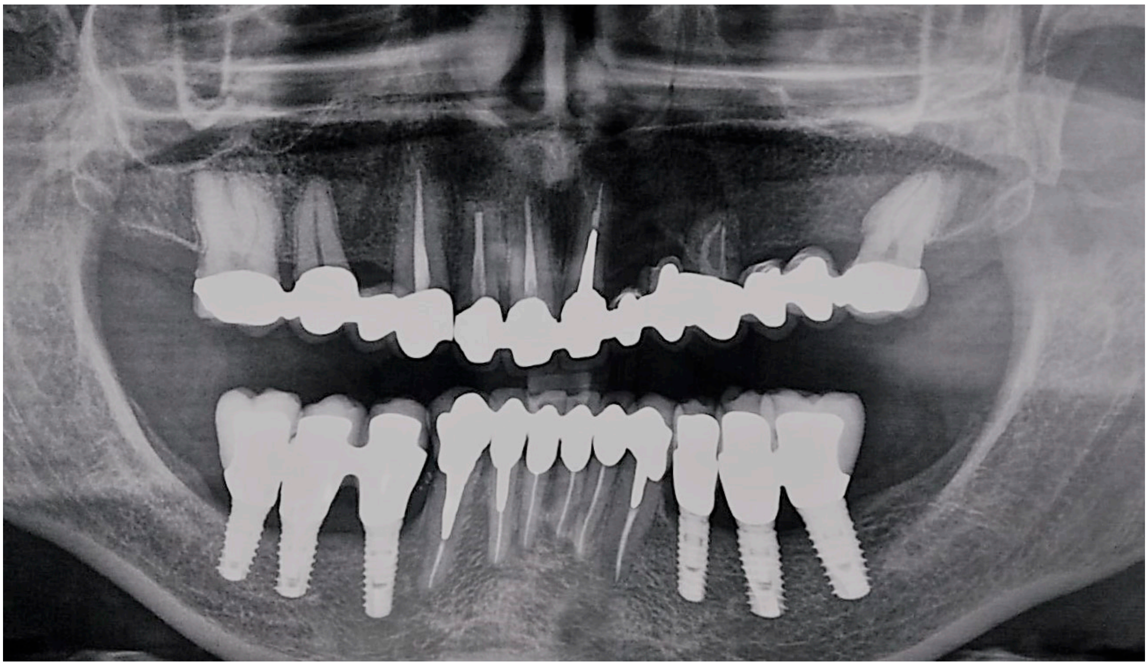

Figure 5. Twelve month follow-up: Orthopantomography.

3. Discussion

In a literature analysis of the PubMed database (key words: Marsupialization OR Partsch AND implant; Cyst AND dental implant; Cyst AND endosseus implant; Cyst AND impacted implant; Partsch AND dental implant), only a few case reports about implants impacted in a maxillary cyst can be found. Park et al., in an infected postoperative maxillary cyst, performed cystectomy, implant conservation, decontamination, and bone grafting to fill the defect [20]. Other authors have reported cases of nasopalatine duct cysts impacted in dental implants. These kinds of lesions probably developed from the inclusion of epithelial remnants of the nasopalatine duct. The therapeutic planning foresaw different approaches: implant and cyst removal, cystectomy, and the resection of the apical portion of the implant inside the cyst cavity [10,21]. Sukegawa et al. decided to perform a cystectomy, but did not perform any implant extractions due to implant stability and no report of perimplant bone resorption [22].

The patient was referred to our clinic with a painless buccal lateral expansion and vestibular purulent sinus discharge. The radiological exam showed that the lesion was associated with a dental implant; peri-implant extensive bone resorption was observed. The endodontic treatments were incomplete. Considering the radiological result, since radicular cysts usually present as unilocular, radiolucent lesions without prominent clinical symptoms, differential diagnosis should include the odontogenic keratocyst, unilocular ameloblastoma, and odontogenic fibromyxoma [2]. Our case was diagnosed as a radicular cyst for the following reasons: presence of large and painless radiolucent lesion in relation to teeth roots previously endodontically treated, size larger than 2 cm, and histological diagnosis confirmation of cystic epithelial lining [23]. The relation between dental implants and cysts has been analyzed in the literature both preliminarily on implant insertion and late cyst development in the fixture insertion area. Researchers have also focused on post-cystectomy surgical timing and implant insertion in post-extractive infected sites [11–18]. Furthermore, studies have been made on implant periapical lesion and radiolucent lesions that could derive from epithelium inclusion in particular situations such as dental implant fixation following sinus augmentation [7–10]. The authors wanted to find a treatment that allowed the patient to preserve the implant itself, avoiding subsequent surgical procedures. The first step was the marsupialization of the cyst, usually reserved for patients with deciduous or mixed dentition or cysts >5 cm, removing the inflammation stimuli coming from

the root. The decompression of the cyst would allow the bone to regenerate due to the increase in the osteoblastic activity [24]. During aftercare, we performed an orthograde retreatment of the previous endodontics in order to avoid disease recurrence. The Partsch I technique was chosen in order to guarantee the preservation of the impacted but stable implant, to simplify the endodontic retreatment, and to minimize the inflammatory phase characterized by the purulent drainage. Various assessment methods of implant stability are described in the literature: tapping the implant with metal instruments and analyzing the resulting sound, the application of reverse torque over the implant, Periotest or resonance frequency value (RFA) devices [25,26]. In our case, implant stability was achieved thanks to the block performed by the rigid prosthetic connection between the affected dental implant and the sound adjacent implants. Considering that, in order to perform any analytic analysis on the implant itself, the overdenture should have been detached, the authors decided not to remove it and to perform the treatment protocol directly. Partsch I cystotomy or marsupialization consists of uniting the cyst lining to the oral mucosa whether with intraoral devices or simply with an iodine gauze inserted in the socket [27–29]. This method has fewer complications than Partsch II enucleation regarding the preservation of anatomical structures, but requires a prolonged treatment period and there is a possibility of leaving pathologic tissue in situ. Ameloblastoma, squamous cell carcinoma, or mucoepidermoid carcinomas have, in fact, been reported to form from the cells in the lining of the cyst [30,31]. Marsupialization, which is a more conservative option, was the optimal treatment since clinical and radiological analysis showed a positive lesion and considering the goal of subsequent implant osseointegration [17,32]. Histological analysis performed during surgery confirmed our hypothesis excluding any malignant neoplasms. Karamanis et al. underlined that in a tissue with an inflammation history, the possible existence of adhesions may complicate the procedure and make it less predictable. However, they raised concerns about the possible defect size following enucleation and possible need for bone grafting [11]. Some authors have recommended titanium brushing to debride and mechanically decontaminate implant surfaces and suggested the use of antibiotics and chemical decontamination, although the literature seems inconclusive on this operative protocol [20,33–35]. The authors also decided not to follow through these procedures since the cyst wall seemed intact and presumably absolved a barrier function between implants and cyst cavity, and the surgical access did not allow it. At the twelve month follow-up, all clinical and radiographic findings showed healing and new bone formation.

4. Conclusions

This report highlights the opportunity to apply a conservative procedure, marsupialization, when the cyst involves a dental implant, thus avoiding the extraction of the implant itself and the long treatment plan required to allow the insertion of a new fixture in the same position.

Author Contributions: Investigation, R.D.L.; Formal analysis, F.L.G.; Project administration, S.D.; Resources, G.C.; Supervision, G.C. and G.T.; Writing—original draft, A.T.; Writing—review & editing, G.L.G. All authors have read and agreed to the published version of the manuscript.

Funding: This research received no external funding.

Conflicts of Interest: The authors declare no conflict of interest.

References

1. Johnson, N.; Gannon, O.; Savage, N.W.; Batstone, M.D. Frequency of odontogenic cysts and tumors: A systematic review. *J. Investig. Clin. Dent.* **2013**, *5*, 9–14. [CrossRef] [PubMed]
2. Jansson, L.; Ehnevid, H.; Lindskog, S.; Blomlöf, L. Development of periapical lesions. *Swed. Dent. J.* **1993**, *17*, 85–93. [PubMed]
3. Wright, J.M.; Vered, M. Update from the 4th Edition of the World Health Organization Classification of Head and Neck Tumours: Odontogenic and Maxillofacial Bone Tumors. *Head Neck Pathol.* **2017**, *11*, 68–77. [CrossRef] [PubMed]

4. Manor, E.; Kachko, L.; Puterman, M.B.; Szabo, G.; Bodner, L. Cystic Lesions of the Jaws—A Clinicopathological Study of 322 Cases and Review of the Literature. *Int. J. Med. Sci.* **2011**, *9*, 20–26. [CrossRef] [PubMed]
5. Giudice, R.L.; Puleio, F.; Rizzo, D.; Alibrandi, A.; Giudice, G.L.; Centofanti, A.; Fiorillo, L.; Di Mauro, D.; Nicita, F. Comparative Investigation of Cutting Devices on Bone Blocks: An SEM Morphological Analysis. *Appl. Sci.* **2019**, *9*, 351. [CrossRef]
6. Castro-Núñez, J.; Information, P.E.K.F.C. Decompression of Odontogenic Cystic Lesions: Past, Present, and Future. *J. Oral Maxillofac. Surg.* **2016**, *74*, 104.e1–104.e9. [CrossRef] [PubMed]
7. Piattelli, A.; Scarano, A.; Piattelli, M.; Podda, G. Implant periapical lesions: Clinical, histologic, and histochemical aspects. A case report. *Int. J. Periodontics Restor. Dent.* **1998**, *18*, 181–187.
8. Oh, T.-J.; Yoon, J.; Wang, H.-L. Management of the Implant Periapical Lesion: A Case Report. *Implant Dent.* **2003**, *12*, 41–46. [CrossRef]
9. Blaya-Tárraga, J.-A.; Cervera-Ballester, J.; Peñarrocha-Oltra, D.; Peñarrocha-Diago, M. Periapical implant lesion: A systematic review. *Medicina Oral Patología Oral Y Cirugia Bucal* **2017**, *22*, e737–e749. [CrossRef]
10. Casado, P.; Donner, M.; Pascarelli, B.; Derocy, C.; Duarte, M.E.L.; Barboza, E.P. Immediate Dental Implant Failure Associated With Nasopalatine Duct Cyst. *Implant Dent.* **2008**, *17*, 169–175. [CrossRef]
11. Karamanis, S.; Kitharas, T.; Tsoukalas, D.; Parissis, N. Implant Placement After Marsupialization of a Dentigerous Cyst. *J. Oral Implant.* **2006**, *32*, 313–316. [CrossRef] [PubMed]
12. Aboulhosn, M.; Noujeim, Z.; Nader, N.; Berbéri, A. Decompression and Enucleation of a Mandibular Radicular Cyst, Followed by Bone Regeneration and Implant-Supported Dental Restoration. *Case Rep. Dent.* **2019**, *2019*, 1–8. [CrossRef] [PubMed]
13. Aoki, N.; Ise, K.; Inoue, A.; Kosugi, Y.; Koyama, C.; Iida, M.; Baba, J.; Iwai, T.; Mitsudo, K. Multidisciplinary approach for treatment of a dentigerous cyst – marsupialization, orthodontic treatment, and implant placement: A case report. *J. Med. Case Rep.* **2018**, *12*, 305. [CrossRef] [PubMed]
14. Al-Shamiri, H.M.; Elfaki, S.; Al-Maweri, S.; Alaizari, N.A.; Tarakji, B. Development of Nasopalatine Duct Cyst in Relation to Dental Implant Placement. *N. Am. J. Med. Sci.* **2016**, *8*, 13–16. [CrossRef]
15. Narad, C.; Lingraj, J.; Aulakh, K.K.; Handa, K.; Kotrashetti, S.; Pinto, P. Assessment of primary stability of the implant placed in prepared infected extraction sockets. *J. Oral Biol. Craniofacial Res.* **2016**, *8*, 154–157. [CrossRef]
16. Jofré, J.; Valenzuela, D.; Quintana, P.; Asenjo-Lobos, C. Protocol for Immediate Implant Replacement of Infected Teeth. *Implant Dent.* **2012**, *21*, 287–294. [CrossRef]
17. Crespi, R.; Capparè, P.; Giudice, G.L.; Gastaldi, G.; Gherlone, E. Immediate Implant Placement in Sockets with Asymptomatic Apical Periodontitis. *Clin. Implant Dent. Relat. Res.* **2016**, *19*, 20–27. [CrossRef]
18. Crespi, R.; Capparè, P.; Crespi, G.; Giudice, G.L.; Gastaldi, G.; Gherlone, E. Dental Implants Placed in Periodontally Infected Sites in Humans. *Clin. Implant Dent. Relat. Res.* **2016**, *19*, 131–139. [CrossRef]
19. Solderer, A.; Al-Jazrawi, A.; Sahrmann, P.; Jung, R.E.; Attin, T.; Schmidlin, P. Removal of failed dental implants revisited: Questions and answers. *Clin. Exp. Dent. Res.* **2019**, *5*, 712–724. [CrossRef]
20. Park, W.-B.; Kim, Y.-J.; Han, J.-Y.; Kang, P. Successful Management of Dental Implants in Postoperative Maxillary Cyst: A Case Report With a 13-Year Follow-Up. *J. Oral Implant.* **2020**, *46*, 133–138. [CrossRef]
21. Jimbo, R.; Takahashi, T.; Takeshita, K.; Funaki, K. Nasopalatine duct cyst developed in association with dental implant treatment: A case report and histopathological observation. *J. Oral Maxillofac. Pathol.* **2013**, *17*, 319. [CrossRef] [PubMed]
22. Sukegawa, S.; Kanno, T.; Kawai, H.; Takebe, Y.; Shibata, A.; Takahashi, Y.; Nagatsuka, H.; Furuki, Y. Nasopalatine duct cyst associated with dental implant treatment: A case report. *Oral Maxillofac. Surg. Cases* **2015**, *1*, 38–41. [CrossRef]
23. Contar, C.M.M.; Thomé, C.A.; Pompermayer, A.; Sarot, J.R.; Vinagre, R.O.; Machado, M.A.N. Marsupialization of Dentigerous Cyst: Report of a Case. *J. Maxillofac. Oral Surg.* **2011**, *14*, 4–6. [CrossRef] [PubMed]
24. Hu, X.; Zhao, Y.-F.; Man, Q.-W.; Li, R.-F.; Liu, B.; Zhao, Y.-F. The effects of marsupialization on bone regeneration adjacent to keratocystic odontogenic tumors, and the mechanisms involved. *J. Oral Sci.* **2017**, *59*, 475–481. [CrossRef]
25. Sennerby, L.; Meredith, N. Implant stability measurements using resonance frequency analysis: Biological and biomechanical aspects and clinical implications. *Periodontology* **2008**, *47*, 51–66. [CrossRef]

26. Andreotti, A.M.; Goiato, M.C.; Nobrega, A.S.; Da Silva, E.V.F.; Filho, H.G.; Pellizzer, E.P.; Dos Santos, D.M. Relationship Between Implant Stability Measurements Obtained by Two Different Devices: A Systematic Review. *J. Periodontol.* **2017**, *88*, 281–288. [CrossRef]
27. Lee, S.-T.; Kim, S.-G.; Moon, S.Y.; Oh, J.; You, J.-S.; Kim, J.-S. The effect of decompression as treatment of the cysts in the jaws: Retrospective analysis. *J. Korean Assoc. Oral Maxillofac. Surg.* **2017**, *43*, 83–87. [CrossRef]
28. Moturi, K.; Puvvada, D.; Kotha, P.R. A Novel, Minimally Invasive Technique in the Management of a Large Cyst Involving the Maxilla in a Child: A Case Report. *Cureus* **2018**, *10*. [CrossRef]
29. Castro-Núñez, J. An Innovative Decompression Device to Treat Odontogenic Cysts. *J. Craniofacial Surg.* **2016**, *27*, 1. [CrossRef]
30. Lee, F. Ameloblastoma of the maxilla with probable origin in a residual cyst. *Oral Surg. Oral Med. Oral Pathol.* **1970**, *29*, 799–805. [CrossRef]
31. Van Der Wal, K.G.; De Visscher, J.G.; Eggink, H.F. Squamous cell carcinoma arising in a residual cyst. A case report. *Int. J. Oral Maxillofac. Surg.* **1993**, *22*, 350–352. [CrossRef]
32. Ghandour, L.; Bahmad, H.F.; Bou-Assi, S. Conservative Treatment of Dentigerous Cyst by Marsupialization in a Young Female Patient: A Case Report and Review of the Literature. *Case Rep. Dent.* **2018**, *2018*, 1–6. [CrossRef] [PubMed]
33. Valderrama, P.; Thomas, G.W., Jr. Detoxification of Implant Surfaces Affected by Peri-Implant Disease: An Overview of Surgical Methods. *Int. J. Dent.* **2013**, *2013*, 1–9. [CrossRef]
34. Zabtotsky, M.H.; Diedrich, D.L.; Meffert, R.M. Detoxification of endotoxin-contaminated titanium and hydroxyapatite-coated surfaces utilizing various chemotherapeutic and mechanical modalities. *Implant Dent.* **1992**, *1*, 154–158. [CrossRef]
35. An, Y.-Z.; Lee, J.-H.; Heo, Y.-K.; Lee, J.-S.; Jung, U.-W.; Choi, S.-H. Surgical Treatment of Severe Peri-Implantitis Using a Round Titanium Brush for Implant Surface Decontamination: A Case Report with Clinical Reentry. *J. Oral Implant.* **2017**, *43*, 218–225. [CrossRef] [PubMed]

© 2020 by the authors. Licensee MDPI, Basel, Switzerland. This article is an open access article distributed under the terms and conditions of the Creative Commons Attribution (CC BY) license (http://creativecommons.org/licenses/by/4.0/).
