# Supplementary material for: A novel histopathological classification of implant periapical lesion: A systematic review and treatment decision tree
Source: PLoS One. 2022 Dec 22;17(12):e0277387. doi: 10.1371/journal.pone.0277387 (PMC9778521; doi:10.1371/journal.pone.0277387)
Supplement: S1 File — (ZIP) [file pone.0277387.s001.zip › support files/Included study/Tseng 2005.pdf]

Peri-Implant Pathology Caused by Periapical Lesion of an Adjacent Natural Tooth: A Case Report

Chuen-Chyi Tseng, DDS, MS[1]/Yea-Huey Melody Chen, DDS, MS[2]/Iok-Chao Pang, DDS, MS[3]/
Hans-Peter Weber, DMD, Dr Med Dent[4]

An implant was removed 6 months after restoration because of peri-implant pathosis. The implant had been placed adjacent to the mandibular right second premolar and close to a periapical lesion of the endodontically treated adjacent first premolar. Along with removal of the failed implant and an apicoectomy of the problem tooth, guided bone regeneration was used to restore the alveolar ridge defect at the site of the failed implant. A replacement implant was placed 6 months later and successfully restored after healing. INT J ORAL MAXILLOFAC IMPLANTS 2005;20:632–635

Key words: *dental implants, implant failure, peri-implantitis*

Implant therapy has become an important part of dentistry for completely and partially edentulous patients.[1] Successful implants are not only characterized by achieving osseointegration but also by their placement in a correct position for prosthetic restoration.[2] Therefore, many factors have to be considered when planning implant treatment in the partially edentulous jaw. One of the factors is the pulpal/periapical condition of adjacent teeth. If there is concern about the status of the pulp or the quality of root canal treatment of teeth adjacent to the implant site, appropriate endodontic therapy should be completed before implant placement.[3–8] Placing an implant too close to an apically compromised natural tooth may cause peri-implant pathosis. Potential sources of microbial contamination of the healing implant should be eradicated to reduce the risk of implant failure. If the lesion persists after conventional root canal therapy, apical surgery with retrograde filling or extraction is recommended.[9]

This clinical case report documents the treatment of a failed implant that apparently was affected by the periapical pathology of an adjacent endodontically treated tooth. A new implant was placed after apicoectomy of the adjacent natural tooth and successful guided bone regeneration at the implant site.

CASE REPORT

A 33-year-old woman came to the Dental Department of the Chi Mei Medical Center seeking to have her missing teeth—the mandibular left second premolar and second molar and the mandibular right second premolar and first molar—restored. After thorough clinical and radiographic examination (Fig 1a), a treatment plan was proposed that included initial periodontal therapy, endodontic treatment of the mandibular right first premolar, and orthodontic alignment of the malpositioned teeth, as well as implant placement and the restoration of the missing teeth. The patient consented to the treatment plan. After completion of the periodontal, endodontic, and orthodontic treatment, 4 ITI dental implants (Straumann, Waldenburg, Switzerland) were placed

[1]Deputy Chairman, Dental Department, Chi Mei Medical Center, Tainan and Associate Professor, Dental Department, National Cheng University, Tainan, Taiwan.
[2]Director of Dental Department, Chi Mei Hospital Liouying, Tainan, Taiwan.
[3]Director of Prosthodontic Division, Dental Department, Chi Mei Medical Center, Tainan, Taiwan.
[4]Professor and Chair, Department of Restorative Dentistry and Biomaterials Sciences, Harvard School of Dental Medicine, Boston, Massachusetts.

Correspondence to: Dr Yea-Huey Melody Chen, Dental Department, Chi Mei Hospital Liouying, No. 201 TaiKang Village, Liouying Township, Tainan 736, Taiwan. E-mail: jimtseng@ms15.hinet.net

COPYRIGHT © 2005 BY QUINTESSENCE PUBLISHING CO, INC.
PRINTING OF THIS DOCUMENT IS RESTRICTED TO PERSONAL USE ONLY.
NO PART OF THIS ARTICLE MAY BE REPRODUCED OR TRANSMITTED IN ANY FORM
WITHOUT WRITTEN PERMISSION FROM THE PUBLISHER.

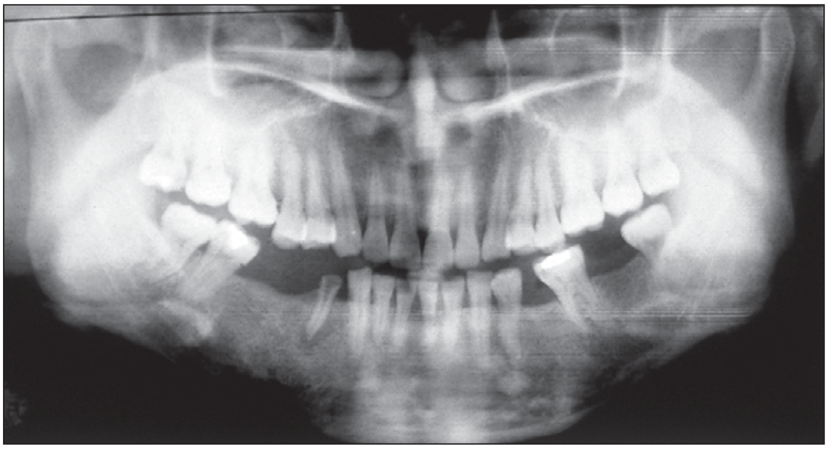

Fig 1a (*Left*) Initial panoramic radiograph.

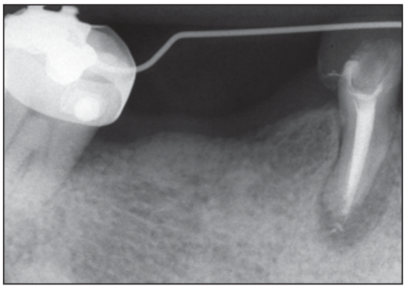

Fig 1b (*Below*) Periapical radiograph after orthodontic treatment and root canal therapy of the mandibular right first premolar.

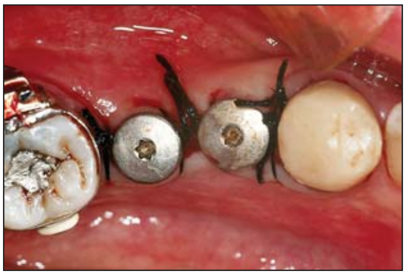

Fig 2a Two implants placed in the areas of the mandibular right second premolar and first molar. The implants were correctly positioned from a restorative viewpoint.

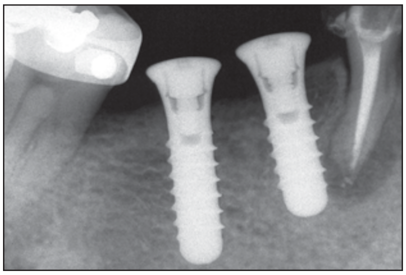

Fig 2b Periapical radiograph immediately after implant placement. Note the proximity of the implant to the first premolar.

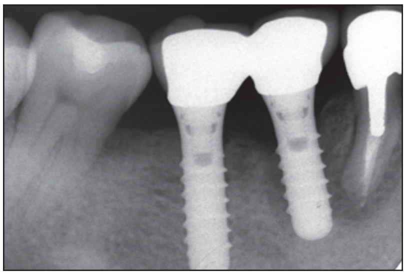

Fig 3 Periapical radiograph after crown placement. Slight radiolucency on the distal aspect of the implant in the mandibular right second premolar area.

in the areas of the missing teeth (Fig 1b). Although the position and alignment of the implants in the mandibular right quadrant appeared optimal (Fig 2a), the implant in the right second premolar area was found to be close to the periapical area of the first premolar when viewed in the postsurgical radiograph (Fig 2b). Postoperative wound healing was uneventful. A solid abutment was connected to the implant with 35 Ncm torque, and a crown was fabricated and cemented without any problems. A postoperative radiograph revealed a small radiolucency on the distal aspect of the implant (Fig 3).

During the 6-month follow-up period, no discomfort was reported by the patient. However, some marginal gingival recession on the buccal aspect of the premolar implant occurred. A periapical radiograph revealed that the radiolucency around the implant had increased in size. Clinically, 9-mm pockets were probed on the buccal and distal aspects of the implant. It was decided to debride the lesion around the implant, perform an apicoectomy and a retrograde filling of the first premolar, and regenerate the lost bone in the area using guided bone regeneration.

However, after flap reflection, it became obvious that the implant had to be removed because of loss of osseointegration (Fig 4a). An apical resection and retrograde filling with mineral trioxide aggregate (Dentsply Friadent Ceramed, Lakewood, CO) were performed on the first premolar (Fig 4b). After implant removal, the large osseous defect and the apical bony cavity were filled with Bio-Oss (Geistlich Pharma, Wolhusen, Switzerland) and covered with an absorbable collagen membrane (Peri-Aid; Collagen Matrix, Franklin Lakes, NJ) (Fig 4c). The curetted apical tissue was sent for pathology diagnosis, and a radicular cyst was subsequently diagnosed. The flaps were closed with Gore-Tex sutures (W. L. Gore & Associates, Newark, DE). Wound healing was uneventful.

Six months later, a new ITI implant was placed in the previously augmented site. A bone specimen of the augmented site was taken for histologic examination, and it showed vital bone tissue (Fig 5). The implant was restored with a new cemented single crown and has remained symptom-free (Figs 6a and 6b). The patient is recalled regularly for evaluation and maintenance care.

COPYRIGHT © 2005 BY QUINTESSENCE PUBLISHING CO, INC.
PRINTING OF THIS DOCUMENT IS RESTRICTED TO PERSONAL USE ONLY.
NO PART OF THIS ARTICLE MAY BE REPRODUCED OR TRANSMITTED IN ANY FORM
WITHOUT WRITTEN PERMISSION FROM THE PUBLISHER.

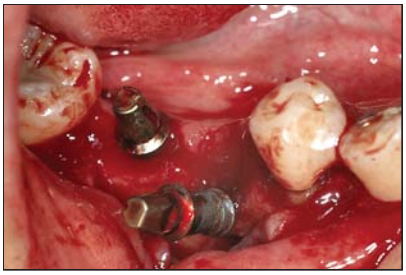

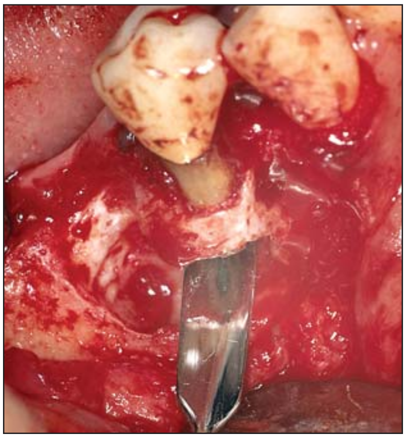

Fig 4a *(Above)* Failed implant after flap elevation.

Fig 4b *(Right)* Apical surgery and retrograde filling with mineral trioxide aggregate on mandibular right first premolar.

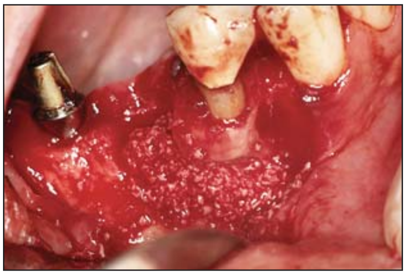

Fig 4c Filling of the osseous defect with Bio-Oss graft (subsequent placement of collagen barrier membrane not shown).

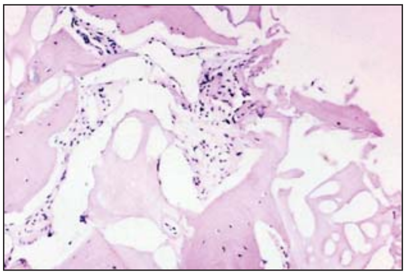

Fig 5 Histologic specimen of bone retrieved during osteotomy of the previously augmented site for new implant placement. The presence of vital bone and remaining graft material is visible (hematoxylin-eosin; original magnification ×100).

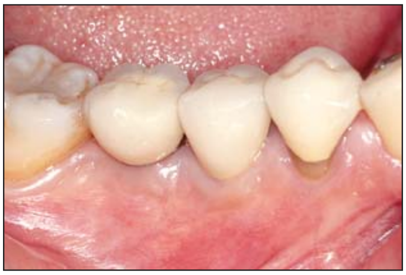

Fig 6a Clinical photograph of the definitive restoration.

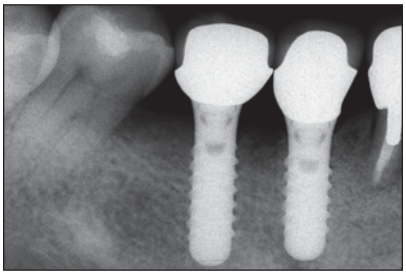

Fig 6b Radiograph of the definitive restoration.

DISCUSSION

Dental implant treatment plays an important role in oral rehabilitation.[1] In recent decades, the concept of restoratively driven implant placement has become well accepted.[2] Consequently, the relationship between implant and natural tooth has to be a major concern when placing implants in the partially edentulous patient. The proximity of the implant to natural tooth roots and their periapical regions must be considered.

Sussman[5] suggested that there are 2 types of periapical implant pathologies: type I, implant to tooth, and type II, tooth to implant. The case described here is a case of type II implant pathology. There is no available literature regarding what distance to allow between an implant and existing diseased periapical tissue. The radiograph obtained after implant placement showed that the implant was very close to the periapical radiolucency of the adjacent endodontically treated first premolar.

The root canal morphology of mandibular premolars has been a challenge in endodontics. The reported incidence of 2 canals ranges from 2.7% to 62.5%.[10,11] In rare cases, 3 canals have been found.[12] In this case, 3 canals were found, indicating the complicated morphology of this root canal system. During the apicoectomy surgery, it was found that the canals had a C-shaped morphology in the apical cross section.

Because of the complex root canal morphology of certain teeth, it is suggested that periapical surgery be performed at the same time as adjacent implant placement if radiographic periapical pathology persists after root canal therapy. Thus, the chance that any remaining peripapical pathosis will compromise successful implant placement can be reduced or eliminated. However, further research evidence is necessary to verify these statements.

The large osseous defect that remained after implant removal was augmented using the concept of guided tissue regeneration with a bone graft and a barrier membrane.[13] The technique of guided bone regeneration using nonresorbable membranes has been used in periodontics and in implant dentistry for more than 10 years. More and more frequently, resorbable membranes have replaced nonresorbable

COPYRIGHT © 2005 BY QUINTESSENCE PUBLISHING CO, INC.
PRINTING OF THIS DOCUMENT IS RESTRICTED TO PERSONAL USE ONLY.
NO PART OF THIS ARTICLE MAY BE REPRODUCED OR TRANSMITTED IN ANY FORM
WITHOUT WRITTEN PERMISSION FROM THE PUBLISHER.

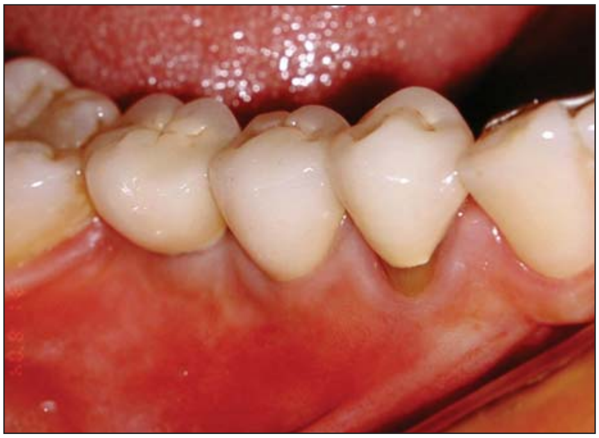

Fig 7a Clinical photograph after 1 year of follow-up.

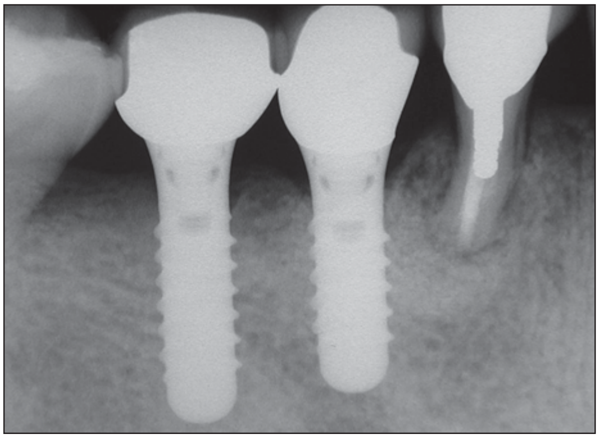

Fig 7b Radiograph obtained after 1 year of follow-up.

ones. The results achieved with resorbable membranes are similar to those previously achieved with nonresorbable ones, and the use of resorbable membranes has simplified the procedure and reduced the number of complications, such as early membrane exposure. In this case, the deficient bone site was augmented using an anorganic bovine bone material (Bio-Oss) combined with a resorbable collagen membrane.[14] The subsequent biopsy demonstrated that the regenerated hard tissue was indeed healthy bone into which the new implant could be placed. The first year of follow-up of the new implant was uneventful (Figs 7a and 7b).

REFERENCES

1. Buser D, Mericske-Stern R, Bernard JP, et al. Long-term evaluation of non-submerged ITI implants. Part I: 8-year life table analysis of prospected multi-center study with 2359 implants. Clin Oral Implants Res 1997;8:161–172.
2. Bahat O, Fontanesi RV, Preston J. Reconstruction of the hard and soft tissues for optimal placement of osseointegrated implants. Int J Periodontics Restorative Dent 1993;13:255–275.
3. Sussman HI, Moss SS. Localized osteomyelitis secondary to endodontic-implant pathosis. A case report. J Periodontol 1993;64:306–310.
4. Sussman HI. Implant pathology associated with loss of periapical seal of adjacent tooth: Clinical report. Implant Dent 1997;6:33–37.
5. Sussman HI. Periapical implant pathology. J Oral Implantol 1998;24:133–138.
6. Shaffer MD, Juruaz DA, Haggerty PC. The effect of periradicular endodontic pathosis on the apical region of adjacent implants. Oral Surg Oral Med Oral Pathol Oral Radiol Endod 1998;86:578–581.
7. Scarano A, Domizio PD, Petrone G, Iezzi G, Piattelli A. Implant periapical lesion: A clinical and histologic case report. J Oral Implantol 2000;26:109–113.
8. Chaffee NR, Lowden K, Tiffee JC, Cooper LF. Periapical abscess formation and resolution adjacent to dental implants: a clinical report. J Prosthet Dent 2001;85:109–112.
9. Misch CE, Reinhardt R (eds). Contemporary Implant Dentistry. St. Louis: Mosby-Year Book, 1993:184.
10. Green D. Double canals in single roots. Oral Surg Oral Med Oral Pathol 1973;35:689–696.
11. Zillich R, Dowson J. Root canal morphology of mandibular first and second premolars. Oral Surg Oral Med Oral Pathol 1973;36:738–744.
12. Vertucci FJ. Root canal anatomy of the human permanent teeth. Oral Surg Oral Med Oral Pathol 1984;58:589–599.
13. Buser D, Dahlin C, Schenk RK. Guided Bone Regeneration in Implant Dentistry. Chicago: Quintessence, 1994:189–264.
14. Zitzmann NU, Naef R, Scharer P. Resorbable versus nonresorbable membranes in combination with Bio-Oss for guided bone regeneration. Int J Oral Maxillofac Implants 1997;12:844–852.

COPYRIGHT © 2005 BY QUINTESSENCE PUBLISHING CO, INC.
PRINTING OF THIS DOCUMENT IS RESTRICTED TO PERSONAL USE ONLY.
NO PART OF THIS ARTICLE MAY BE REPRODUCED OR TRANSMITTED IN ANY FORM
WITHOUT WRITTEN PERMISSION FROM THE PUBLISHER.
