# Supplementary material for: A novel histopathological classification of implant periapical lesion: A systematic review and treatment decision tree
Source: PLoS One. 2022 Dec 22;17(12):e0277387. doi: 10.1371/journal.pone.0277387 (PMC9778521; doi:10.1371/journal.pone.0277387)
Supplement: S1 File — (ZIP) [file pone.0277387.s001.zip › support files/Table in Excel and Word/Table 1.docx]

Table 1. The present classification systems regarding the IPL.

| Author | Category | | Definition |
| --- | --- | --- | --- |
| Reiser & Nevins | Inactive (non- infected) | | Apical scar, overdrilling |
|  | Active（infected） | | Residual infection or contaminated implant |
| Sussman | Implant to Tooth | | Osteotomy preparation causes adjacent tooth pulp devitalization |
|  | Tooth to Implant | | Adjacent tooth periapical pathology or previously existing apical lesion |
| Sarmast et al | Class1, 2 same as Sussman | | Same as Sussman |
|  | Improper placement or angulation of the implant | | Implants that are placed too far labially or lingually/palatially |
|  | Residual infection | | Residual bacteria/viruses and/or necrotic bone/subclinical infection or placement into an infected or inflamed sinus |
| Penarrocha-Diago et al. | Inactive | Asymptomatic | Apical scar caused by overpreparation or by bone necroses due to overheating |
|  | Active | Acute non-suppurated | Acute, spontaneous, continuous pain  Mucosa can be swelled and reddish  No peri-implant alterations |
|  |  | Acute suppurated | Implant periapical radiolucency  others same as non-suppurated |
|  |  | subacute or suppurated-fistulized | Dull pain；Periapical radiolucent area  Possible fistulous tract or abscess or implant mobility |
| Kadkhodazadeh & Amid | Primary periodontal lesions (P-class) | | P1: apical peri-implantitis |
|  |  |  | P2: marginal peri-implantitis |
|  |  |  | P3: marginal and apical peri-implantitis |
|  | Primary Implant complications (I-class) | | I1: apical periodontitis |
|  |  |  | I2: marginal periodontitis |
|  |  |  | I3: marginal and apical periodontitis |
|  | Periodontal and peri-implant lesions | | S1: apical lesions |
|  |  |  | S2: marginal lesions |
|  |  |  | S3: marginal and apical lesions |
|  | Traumatic lesions with an iatrogenic origin | | T0: Non symptomatic |
|  |  |  | T1: symptomatic lesions |
| Shah et al. | Mild | | <25% of the implant length from apex |
|  | Moderate | | 25–50% of the implant length from apex |
|  | Advanced | | >50% of the implant length from apex |
